# A revision of Passiflora L. subgenus Decaloba (DC.) Rchb. supersection Cieca (Medik.) J. M. MacDougal & Feuillet (Passifloraceae)

**DOI:** 10.3897/phytokeys.43.7804

**Published:** 2014-11-06

**Authors:** Kristen Porter-Utley

**Affiliations:** 1 Department of Biology, Keene State College, 229 Main Street, Mail Stop 2001, Keene, New Hampshire 03435-2001, United States of America

**Keywords:** *Passiflora*, *Cieca*, morphology, ITS, species complex, cladistics

## Abstract

Passiflora subgenus Decaloba supersection Cieca is a monophyletic group of herbaceous to woody climbers found in subtropical and tropical regions of the world. The 19 species recognized here are primarily distributed in the southern United States, Mexico, Central America, South America, and the Caribbean. Two species, *Passiflora suberosa* and *Passiflora pallida*, are also naturalized in various regions of the Old World. The species of the supersection are recognized by their small, apetalous, usually greenish flowers with the filaments of the corona mostly in two series. The plants commonly lack c-glycosylflavones but possess flavonol 3-O-glycosides. The supersection contains two problematic species complexes, *Passiflora suberosa* and *Passiflora coriacea*. Phylogenetic relationships within supersection *Cieca* are investigated by means of phenetic and cladistic analyses of morphological and molecular (ITS 1 & 2) characters. The morphological and molecular data sets were analyzed separately because of incongruity due to taxon sampling and the complicated evolutionary history of entities within the *Passiflora suberosa* complex. All analyses confirm the monophyly of the supersection. They also show that the *Passiflora suberosa* complex is a non-monophyletic group of cryptic species, and inter-taxic hybridization and polyploidy have contributed to the confusing and complex pattern of variation evident within the group. Four taxa that were formerly included in this complex are recognized: *Passiflora pallida*, Passiflora suberosa subsp. suberosa, Passiflora suberosa subsp. litoralis, and *Passiflora tridactylites*. On the basis of molecular and morphological data, three species from the *Passiflora coriacea* complex are recognized: *Passiflora coriacea*, *Passiflora sexocellata*, and *Passiflora megacoriacea*. A key, detailed descriptions, distribution maps, and illustrations are included in the revision. Pollination, dispersal, and herbivory of the group are reviewed. The distribution and ecology of the species within the supersection are also discussed.

## Introduction

Passiflora L. subgenus Decaloba (DC.) Rchb. supersection Cieca (Medik.) J. M. MacDougal & Feuillet is a monophyletic group of herbaceous to woody climbers found in subtropical and tropical regions of the world from latitude 34°N to latitude 34°S. The 19 species recognized here are primarily distributed in the southern United States, Mexico, Central America, South America, and the Caribbean. Two species, *Passiflora suberosa* L. and *Passiflora pallida* L., also occur in various regions of the Old World, likely as a result of naturalization.

Friedrich Medikus (Medikus 1787) was the first to recognize this group, and he proposed the generic name *Cieca* for the apetalous species of Passifloraceae. Since Medikus’ time several monographers have also acknowledged the phenetic cohesiveness of these species and placed them (or most of them) in their own genus or section. However, the species with tubular, flowers were often excluded and other species of uncertain relationship included. John MacDougal, as part of his revision of Passiflora subgenus Decaloba section Pseudodysosmia (Harms) Killip [=supersection *Bryonioides* (Harms) J.M.MacDougal & Feuillet] (Passifloraceae), was the first to include the tubular-flowered species in supersection *Cieca* and also to transfer various vegetatively divergent species out of the group (MacDougal 1983).

Five subgenera of *Passiflora* are currently recognized: *Passiflora*, *Deidamioides* (Harms) Killip, *Astrophea* (DC.) Mast., *Decaloba* (DC.) Rchb, and *Tetrapathea* (DC.) P.S. Green (Feuillet and MacDougal 2003, Krosnick et al. 2009). Supersection *Cieca* belongs within subgenus *Decaloba* on the basis of having small (<4 cm in diameter) flowers with the corona in a few series (two to three), and a plicate, membranous operculum. The base chromosome number of species in supersection *Cieca* and most species in subgenus *Decaloba* is six (n = 6); one count in a basal lineage of the subgenus is n = 9 (Snow and MacDougal 1993). The species of supersection *Cieca* are easily recognized by their small, apetalous, usually greenish flowers with the filaments of the corona mostly in two series. In addition, the flowers lack bracts or possess only one or two bracts, and the plants commonly lack c-glycosylflavones but possess flavonol 3-O-glycosides.

Several factors enhance the biological significance of Passiflora supersection Cieca. Records of pollination are rare in the supersection, but the species exhibit three pollination syndromes: melittophily (pollination by bees), sphecophily (pollination by wasps), and ornithophily (pollination by birds) (Gilbert 1991; Koschnitzke and Sazima 1997; Lindberg 1998; MacDougal 1992). The species of the supersection are also utilized as larval hosts by most genera of the subfamily Heliconiinae (see section on herbivory) (Benson et al. 1975; Spencer 1988). Four of the 19 species within supersection *Cieca* are listed as endangered or threatened in the 1997 IUCN Red List of Threatened Plants. One species, *Passiflora clypeophylla* Mast., may be extinct and is represented by only a single herbarium specimen. The status of another species, *Passiflora macfadyenii* C.D. Adams, is uncertain because, despite several searches, it has not been found in its native habitat in Jamaica since 1998.

Supersection *Cieca* contains two problematic species complexes, *Passiflora suberosa* and *Passiflora coriacea* Juss. Ever since Linnaeus first described *Passiflora suberosa* in his *Species Plantarum*, taxonomists have disagreed about the circumscription of this widespread species and, as a result, many synonyms exist for it (Linnaeus 1753; The Herbarium of the Royal Botanic Gardens 1996). My analysis of the herbarium specimens of *Passiflora suberosa* *s. l.* indicate that this variable species has served as a “disposal depot” for at least four entities (*Passiflora pallida*, Passiflora suberosa L. subsp. suberosa L., Passiflora suberosa L. subsp. litoralis (Kunth) K.Port.-Utl. ex. M.A.M.Azevedo, Baumgratz & Gonç.-Estev., and *Passiflora tridactylites* Hook.f.) that cannot be assigned to any of the other members of the supersection. Molecular and morphological phylogenetic analyses show that the complex is a non-monophyletic group of cryptic species, a situation not unusual in plants (Rieseberg and Brouillet 1994). *Passiflora coriacea* Juss., as traditionally circumscribed, is another “species” that exhibits marked morphological variation over its distribution from eastern Mexico to northern South America, and evidence presented in this study indicates that it comprises three distinct entities (*Passiflora coriacea*, *Passiflora megacoriacea* K.Port.-Utl., and *Passiflora sexocellata* Schltdl.).

## Taxonomic history

The genus *Passiflora* (Passifloraceae Juss. ex Roussel; tribe Passifloreae DC.) is a large and diverse group of approximately 500 species of vines, lianas, and trees (Feuillet and MacDougal 1999; Feuillet and MacDougal 2003; Killip 1938; Wilde 1971). The geographical distribution of *Passiflora* is primarily restricted to New World tropical, subtropical, and occasionally temperate areas, but approximately 20 species are found in Southeast Asia, Oceania, and Australia. The genus *Passiflora* contains five subgenera: *Passiflora*, *Deidamioides* (Harms) Killip, *Astrophea* (DC.) Mast., and *Decaloba* (DC.) Rchb., and *Tetrapathea* (DC.) P.S.Green (Feuillet and MacDougal 2003, Krosnick et al. 2009). The two largest subgenera in the genus are *Passiflora* (~250 species) and *Decaloba* (~230 species). Supersection *Cieca*, one of eight supersections in subgenus *Decaloba*, is the fourth largest supersection in the subgenus (Feuillet and MacDougal 2003). Nineteen species are recognized in the supersection, and of those, two are newly described in this revision. Traditionally, the tubular-flowered members of the group have been separated from those that possess dish-shaped flowers; the tubular-flowered species often have been placed in segregate sections and genera.

From 1570–1577 Francisco Hernández, the personal physician of King Philip II of Spain, traveled in the Americas in search of new medicines. Hernández spent his time in Mexico and enlisted native guides, artists, herbalists, and physicians to teach him about the *materia medica*, resulting in the earliest treatment of Mexico’s natural history. However, it was not until 1651 that his manuscript was published in *Rerum medicarum Novæ Hispaniæ thesaurus seu plantarum animalium mineralium Mexicanorum historia*. In it was the first description of a plant from supersection *Cieca*, *Passiflora sexocellata* Schltdl. Hernández gave the Aztec name for it, *Tzinacanatlapatli*, followed by a brief description and illustration of the plant (Hernández 1651).

Charles Plumier (1693), in his *Description des plantes de l’Amérique*, described and illustrated four more “species” of supersection *Cieca*: “Clematitis indica, folio hederaceo major, fructu olivae formi” (= *Passiflora suberosa*); “Clematitis indica, folio angusto, trifido, fructu olivae formi” (= *Passiflora suberosa*); “Clematitis indica alia, flore minore pallido” (= *Passiflora pallida*); and “Clematitis indica, flore minimo pallido” (= *Passiflora pallida*). His descriptions and illustrations of the members of the *Passiflora suberosa* complex are truly outstanding and indicate that he had an extensive knowledge of the variation of the group in the Caribbean.

In the year 1719, Joseph Pitton de Tournefort created two genera of passionflowers: *Granadilla* and *Murucuia*. One species with fused coronal filaments was placed in the genus *Murucuia* (= *Passiflora murucuja* L.). The remaining 23 species recognized by Tournefort, including the species of supersection *Cieca* described and illustrated by Plumier, were placed in the genus *Granadilla* (Tournefort 1719).

Plumier and Tournefort, along with authors like Robert Morison and Leonard Plukenet, laid the foundation for the work of Carolus Linnaeus (Linnaeus 1745, 1749; Morison 1680; Plukenet 1691, 1696). In his *Dissertatio Botanica de* Passiflora, Linnaeus illustrated the leaves and described 22 species of passionflowers with direct references to earlier synonyms, four of which are members of supersection *Cieca*: “Passiflora foliis indivisis ovatis integerrimis, petiolis biglandulosis” (= *Passiflora pallida*), “Passiflora foliis trilobis peltatis” (= *Passiflora suberosa*), “Passiflora foliis trilobis villosis, floribus opposites” (= *Passiflora pallida*, but considered within *Passiflora hirsuta* by Linnaeus), and “Passiflora foliis trilobis integerrimis, lobis sublanceolatis: intermedio productiore” (= *Passiflora pallida*, but considered within *Passiflora minima* by Linnaeus). Linnaeus included information about the history, nomenclature, distribution, superstitions, and medicinal and economic uses of the plants. In Linnaeus’ (1753) *Species Plantarum*, all of the 22 species from the *Dissertatio* were placed in the genus *Passiflora*, along with two additional species.

In 1782, Friedrich Medikus began to publish essays treating the Passifloraceae (Medikus 1782/1783, 1784). In 1787, as a tribute to Pedro Cieza de León, he created the genus *Cieca* for the apetalous members [*Cieca viridis* (= *Passiflora minima*) and *Cieca nigra* (= *Passiflora suberosa*)] of the group; he did not rename or treat *Passiflora pallida*. He recognized Linnaeus’ genus *Passiflora* but also revived Tournefort’s *Granadilla* and *Murucuia* (Medikus 1787).

The next published monograph was *Decima dissertatio botanica de* Passiflora (Cavanilles 1790) in which a total of 43 species (all placed in the genus *Passiflora*) were described; 32 of the species were illustrated. One new species of supersection *Cieca* [*Passiflora peltata* Cav. (= *Passiflora suberosa* in this revision)] was included, in addition to the four described by Linnaeus. In 1799, Antonio Cavanilles, in his *Icones et Descriptiones Plantarum*, described another species of supersection *Cieca*, the tubular-flowered *Passiflora viridiflora* Cav. (Cavanilles 1799).

In 1805, Antoine Laurent de Jussieu formally described 13 new species of the genus, including *Passiflora coriacea*; he also recognized the genera *Murucuia* and *Tacsonia*. Because of its tubular flowers, he placed the apetalous *Passiflora viridiflora* in the genus *Tacsonia*. In this treatment, Jussieu also discussed in great detail questions of generic delimitation and relationship, and he was the first to suggest that *Passiflora*, *Murucuia*, and *Tacsonia* should be placed together in their own family. However, Jussieu did not use a family name in the official sense (Jussieu 1805a, 1805b). Henri Francois Anne de Roussel, in 1806, was the first to validly publish the family Passifloraceae, with credit to Jussieu. Until recently, the first valid publication of the Passifloraceae was attributed to Karl Sigismund Kunth; however, Roussel’s description of the family was published more than ten years before Kunth’s. Incidentally, Kunth in H.B.K. also published the species *Passiflora tubiflora* but was probably unaware that Cavanilles had already named it *Passiflora viridiflora* (Kunth 1817). In 1826, Curt Polycarp Joachim Sprengel transferred *Passiflora viridiflora* into the genus *Murucuia* (Sprengel 1826). One year later, William Hamilton, in his *Prodromus Plantarum Indiae Occidentalis*, described three new species of *Passiflora*. One of these, *Passiflora lancifolia* Ham., is a red, apetalous, hummingbird-pollinated member of supersection *Cieca* from the Antilles (Hamilton 1825).

In 1822 and 1828, Augustin Pyramus de Candolle subdivided *Passiflora* into eight sections based upon bract and calyx morphology: *Astrophea*, *Polyanthea*, *Tetrapathea*, *Cieca*, *Decaloba*, *Granadilla*, *Tacsonioides*, and *Dysosmia*; he thought that all members of the tribe Passifloreae lacked a corolla. He placed all of the species with dish-shaped flowers that were either ebracteate or possessed small bracts and a five-lobed calyx in section *Cieca*. However, he mistakenly placed individuals that we now know possess five petals and five sepals (a ten-lobed calyx, according to de Candolle) in the section. He did not place *Passiflora lancifolia* in a section because he felt that the species was not sufficiently known. Additionally, he placed *Passiflora viridiflora* in the section *Psilanthus* and accepted Jussieu’s placement of the species in the genus *Tacsonia* (Candolle 1828). In the same year, Heinrich Gottlieb Ludwig Reichenbach (1828) raised section *Decaloba* DC. to the rank of subgenus. Additionally, he placed *Passiflora viridiflora* in the genus *Synactila* Raf.

Max Joseph Roemer (1846) published a monograph of the Passifloraceae and raised de Candolle’s sections to the rank of genera. Thus, most of the apetalous species discussed above were once again placed in their own genus, though Roemer repeated de Candolle’s mistake and also placed petalous species in the genus *Cieca*. He placed *Passiflora lancifolia* in the genus *Decaloba* and recognized *Psilanthus viridiflora* (Roemer 1846). Soon after the publication of Roemer’s monograph, Joseph Dalton Hooker supported de Candolle’s broad and more conservative concept of the genus in his treatment for *Genera plantarum* (Hooker 1867). In the interim, another apetalous species belonging to supersection *Cieca*, *Passiflora tenuiloba* Engelm., was described by George Engelmann (Gray 1850).

In 1871, Maxwell Tylden Masters published a preliminary taxonomic paper on the Passifloraceae in the *Transactions of the Linnaean Society* that would be expanded upon in a comprehensive monograph of the family that appeared a year later in Carolus Martius’ *Flora Brasiliensis* (Masters 1871, 1872). In the 1871 publication, he validly established four subgenera within *Passiflora* based upon various floral characteristics: *Astrophea* (Ohwi) Rchb., *Plectostemma* Mast. (with sects. *Cieca*, *Dysosmia*, and *Decaloba*), *Murucuia* Tourn. ex Mill. (with sects. *Eumurucuia* and *Psilanthus*), and *Granadilla* Mill. Section *Cieca* was put in subgenus *Plectostemma* and consisted of the apetalous members of *Passiflora* that lacked bracts and possessed dish-shaped flowers; he also mistakenly placed some petalous species in the group. In addition, many of the species that previous authors recognized as distinct from *Passiflora suberosa* were reduced to varieties (see discussions of *Passiflora pallida* and *Passiflora suberosa* for details). Masters also placed *Passiflora lancifolia* and *Passiflora viridiflora* in an unnamed section, along with other tubular-flowered species. In the 1872 monograph, he maintained section *Cieca* as described above, but he recognized not only varieties of *Passiflora suberosa* but also subvarieties. In addition, Masters placed *Passiflora lancifolia* and *Passiflora viridiflora* (and the associated species from the 1871 paper) in section *Psilanthus* Hook.f. He also (Masters 1871) put all of these in his subg. *Plectostemma* and appeared to be unaware that Reichenbach (1828) had already elevated *Decaloba* to the rank of subgenus. John Mochrie MacDougal (1983) pointed out that the type species of these two subgenera, subg. *Plectostemma* Mast. and subg. *Decaloba* (DC.) Rchb., are so closely related that for all practical purposes *Decaloba* shoud be used instead of *Plectostemma*. Jose JeronimoTriana and Jules Émile Planchon (1873), in their monograph of the Colombian Passifloraceae adopted Masters’ 1872 classification with one modification. They reduced the genus *Tacsonia* to a subgenus within *Passiflora*. In 1887 and 1891, Masters described two additional species of supersection *Cieca* that are endemic to Guatemala, *Passiflora trinifolia* Mast. and *Passiflora clypeophylla* Mast. (Masters 1887, 1891). In 1890, Martin Sessé y Lacasta and José Mariano Mociño, in *Plantae Novae Hispaniae*, post humously described *Passiflora obtusifolia* Sessé & Moc. (here placed in supersection *Cieca*). There is also an illustration of that species in *Icones Florae Mexicanae* (McVaugh 1977, 1980, 1982; Sessé and Mociño 1887–1890, 1894). However, no later author until Ellsworth Killip (1938) mentioned *Passiflora obtusifolia*.

Hermann Harms, in his *Die Natürlichen Pflanzenfamilien* (1893, 1897, 1925), revised the generic and infrageneric classification of the family. Harms, instead of dividing the genus *Passiflora* into subgenera, separated it into 21 sections; the sections were often divided into subsections or series. He also recognized the New World genera *Dilkea*, *Mitostemma*, and *Tetrastylis*. He defined the members of section *Cieca* as possessing small, whitish or greenish, bowl-shaped flowers without petals. He mistakenly thought that *Passiflora inamoena* A.Gray (= *Passiflora bryonioides* Kunth) lacked petals and therefore included it in section *Cieca*. He placed the apetalous *Passiflora gracilis* J.F.Jacq. ex Link in the section. However, MacDougal (1994) determined (based upon morphological evidence) that it is more closely related to species in supersection *Bryonioides* (Harms) Feuillet & MacDougal than to members of supersection *Cieca*. Harms did not indicate where *Passiflora lancifolia* belonged, but he placed *Passiflora viridiflora* by itself in section *Chloropathanthus* Harms (Harms 1893, 1897, 1925).

In 1938, Killip published a revision of the American Passifloraceae. Killip’s revision, by his own admission, closely approximated that of Harms. The most important differences were the raising of Harms’ sections to subgenera and the regrouping of the species placed by Harms in sections *Decaloba* and *Cieca*. Killip defined section *Cieca* as those members of subgenus *Plectostemma* (= subgenus *Decaloba*) that possess petiolar glands, reticulate seed coats, and bracts that are scattered along the peduncle and more than 1 mm long; he considered the lack of bracts in many of the species of the section to be the result of deciduousness. He placed in the group the apetalous species of *Cieca* Medik., the “Bryonioideae” of Harms, and several other species of uncertain relationship. However, in comparison with other genera in the Passifloraceae, the character states that he used to define the section are plesiomorphic, and the members of his section *Cieca* are now considered an artificial assemblage (MacDougal 1994). He placed *Passiflora viridiflora* and *Passiflora lancifolia* in subgenus *Chloropathanthus* Harms, based upon the lack of a plicate operculum, even though all of the other characters that he used to define this subgenus are the same as those that he used to designate section *Cieca*.

In 1967, another tubular-flowered species of supersection *Cieca* was described, *Passiflora macfadyenii*, a Jamaican endemic (Proctor 1967). MacDougal described four additional species in supersection *Cieca*: *Passiflora eglandulosa* J.M.MacDougal, *Passiflora juliana* J.M.MacDougal, *Passiflora xiikzodz* J.M.MacDougal, and *Passiflora mcvaughiana* J.M.MacDougal (MacDougal 1988, 1992, 2001).

The most recent revision of the genus *Passiflora*, *A new infrageneric classification of Passiflora* (Feuillet and MacDougal 2003), was presented by C. Feuillet and J. M. MacDougal at the International Botanical Congress (St. Louis) in August of 1999 (Feuillet and MacDougal 1999). Feuillet and MacDougal proposed that only four subgenera are sufficient to reflect the most basic phylogenetic relationships within *Passiflora*: *Astrophea*, *Deidamioides*, *Decaloba*, and *Passiflora*. These subgenera are further separated into supersections, sections, and series, and they recognized Medikus’ *Cieca* as a supersection (Feuillet and MacDougal 2003).

## Methods

### Morphological data set

The morphological investigation of supersection *Cieca* is based upon the careful study of over 4,200 dried specimens from 44 herbaria, supplemented with observations from plants preserved in ethyl alcohol and living plants in the field and greenhouse. Of the 19 species in the supersection, 13 (all except *Passiflora clypeophylla*, *Passiflora eglandulosa*, *Passiflora macfadyenii*, *Passiflora trinifolia*, *Passiflora megacoriacea*, and *Passiflora tridactylites*) were collected during field work in Jamaica, Haiti, and Mexico, or donated to the *Passiflora* greenhouse collection at the University of Florida; several correspondents and colleagues contributed living material during the course of this study. Vouchers are deposited at CICY and FLAS.

**Figure 1.**
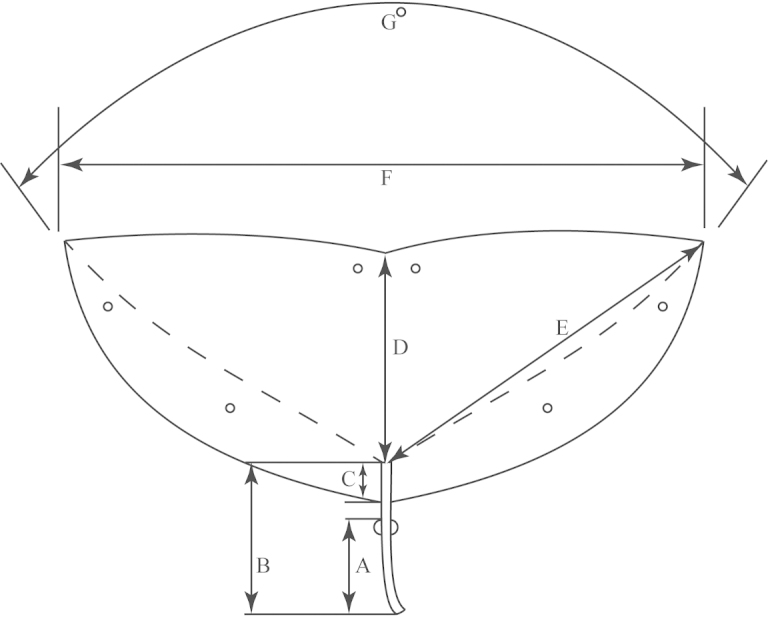
Outline of a bilobed, peltate leaf (typical of e.g., *Passiflora coriacea*) demonstrating method of measurement. **a** Distance from petiolar base to nectary **b** Length of petiole **c** Degree peltate (distance from point of petiolar insertion to leaf base) d. Length of central vein. c+d = leaf length **e** Length of lateral vein **f** Width of leaf **g** Angle between primary lateral veins.

**Figure 2.**
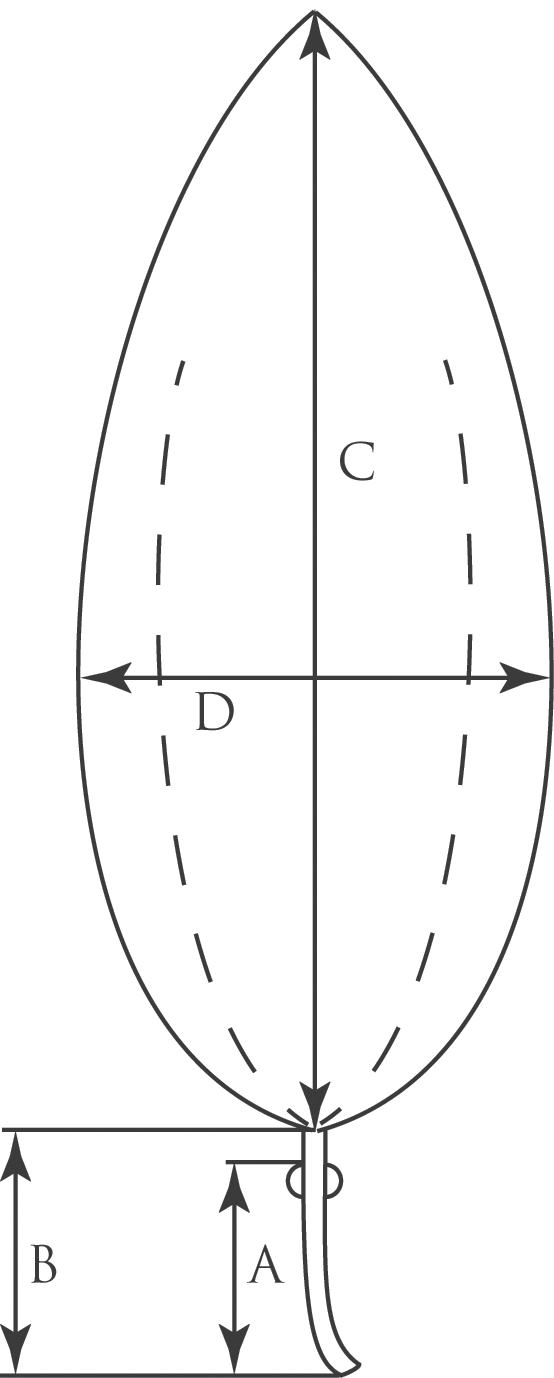
Outline of an unlobed, entire leaf (typical of *Passiflora pallida*) demonstrating method of measurement. **a** Distance from petiolar base to nectary **b** Length of petiole **c** Length of central vein/length of leaf **d** Width of leaf.

**Figure 3.**
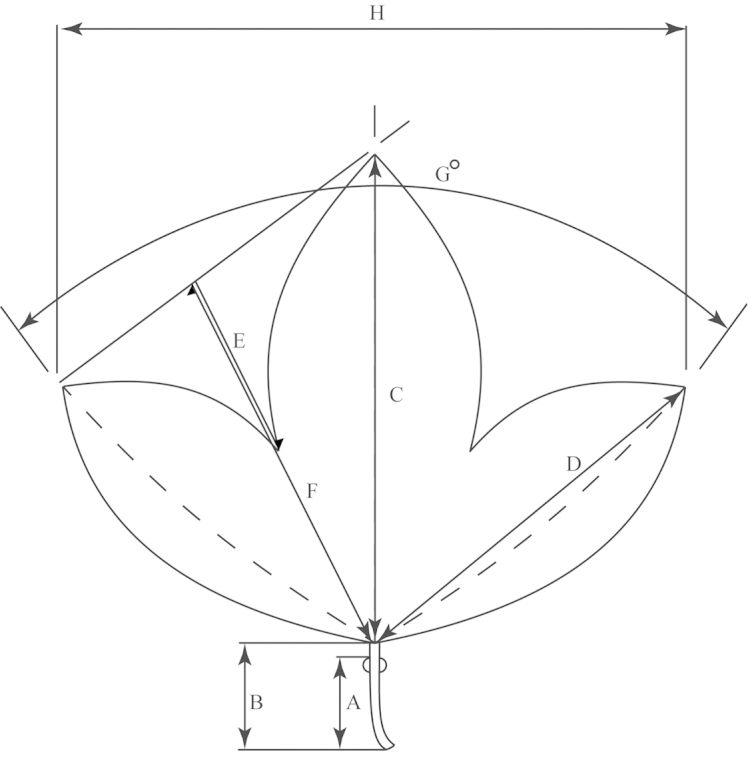
Outline of a trilobed leaf (typical of e.g., *Passiflora suberosa*) demonstrating method of measurement. **a** Distance from petiolar base to nectary **b** Length of petiole **c** Length of central vein/leaf length **d** Length of lateral vein **e** Distance from outline of leaf to margin of sinus **f** Distance from outline of leaf to leaf base measured across deepest part of sinus **g** Angle between primary lateral veins **h** Width of leaf.

**Figure 4.**
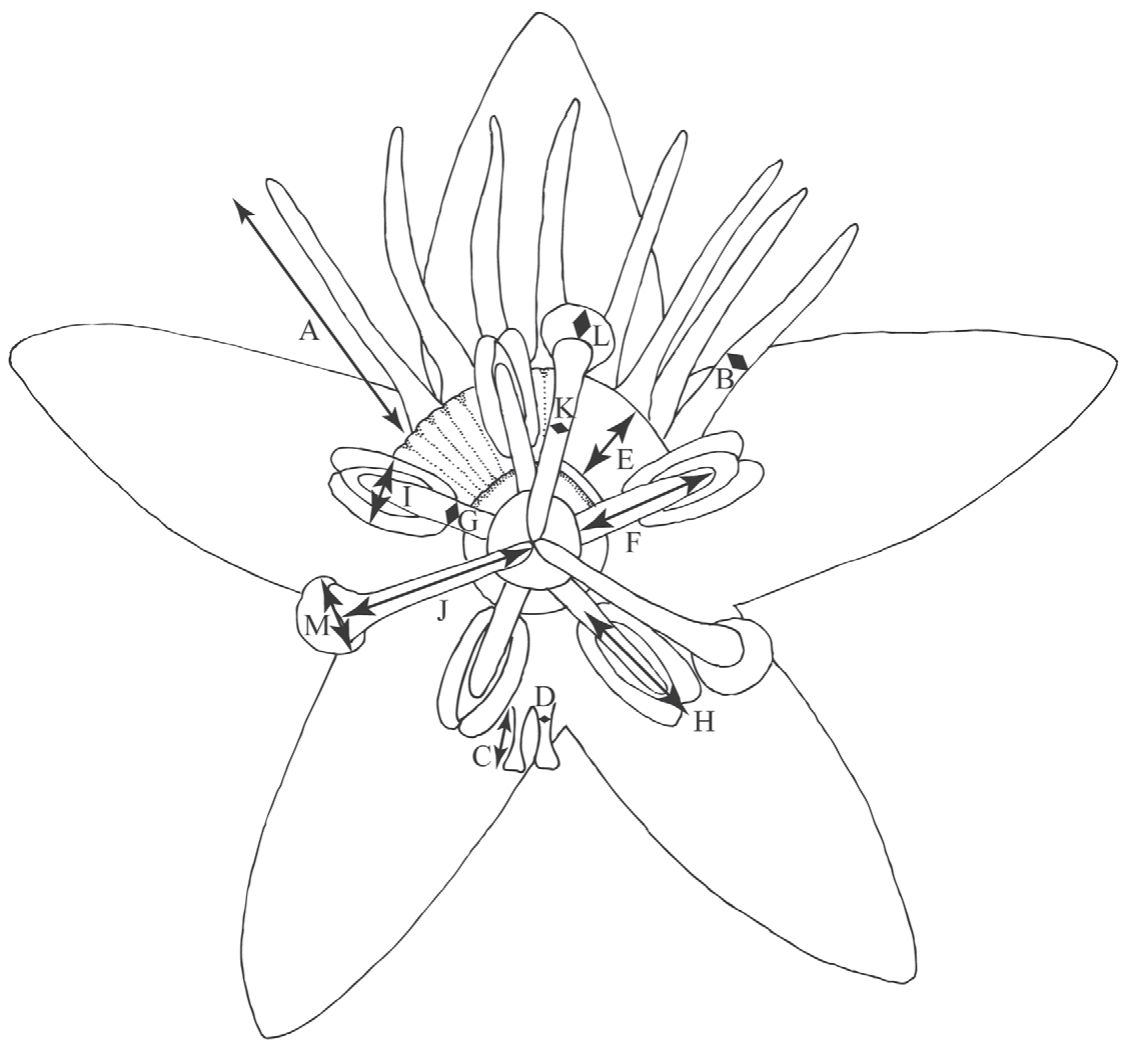
View of flower of *Passiflora coriacea* from the top demonstrating method of measurement. **a** Length of outer coronal filament **b** Width of outer coronal filament **c** Length of inner coronal filament **d** Width of inner coronal filament **e** Nectary diameter **f** Length of staminal filament **g** Width of staminal filament **h** Length of anther **i** Width of anther **j** Length of style **k** Width of style **l** Length of stigma **m** Width of stigma.

**Figure 5.**
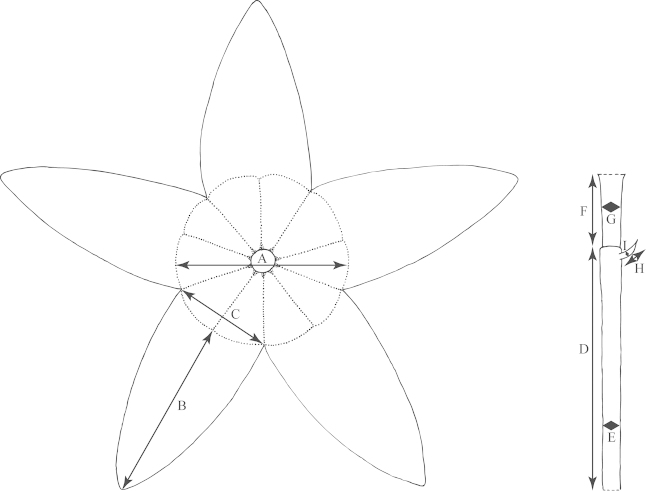
Shown to the left is a view of the flower of *Passiflora coriacea* from the bottom demonstrating method of measurement. Shown to the right is a flower stipe and pedicel of *Passiflora coriacea* demonstrating method of measurement. **a** Width of hypanthium **b** Length of sepal **c** Width of sepal **d** Length of pedicel **e** Width of pedicel **f** Length of stipe **g** Width of stipe **h** Length of bract **i** Width of bract.

**Figure 6.**
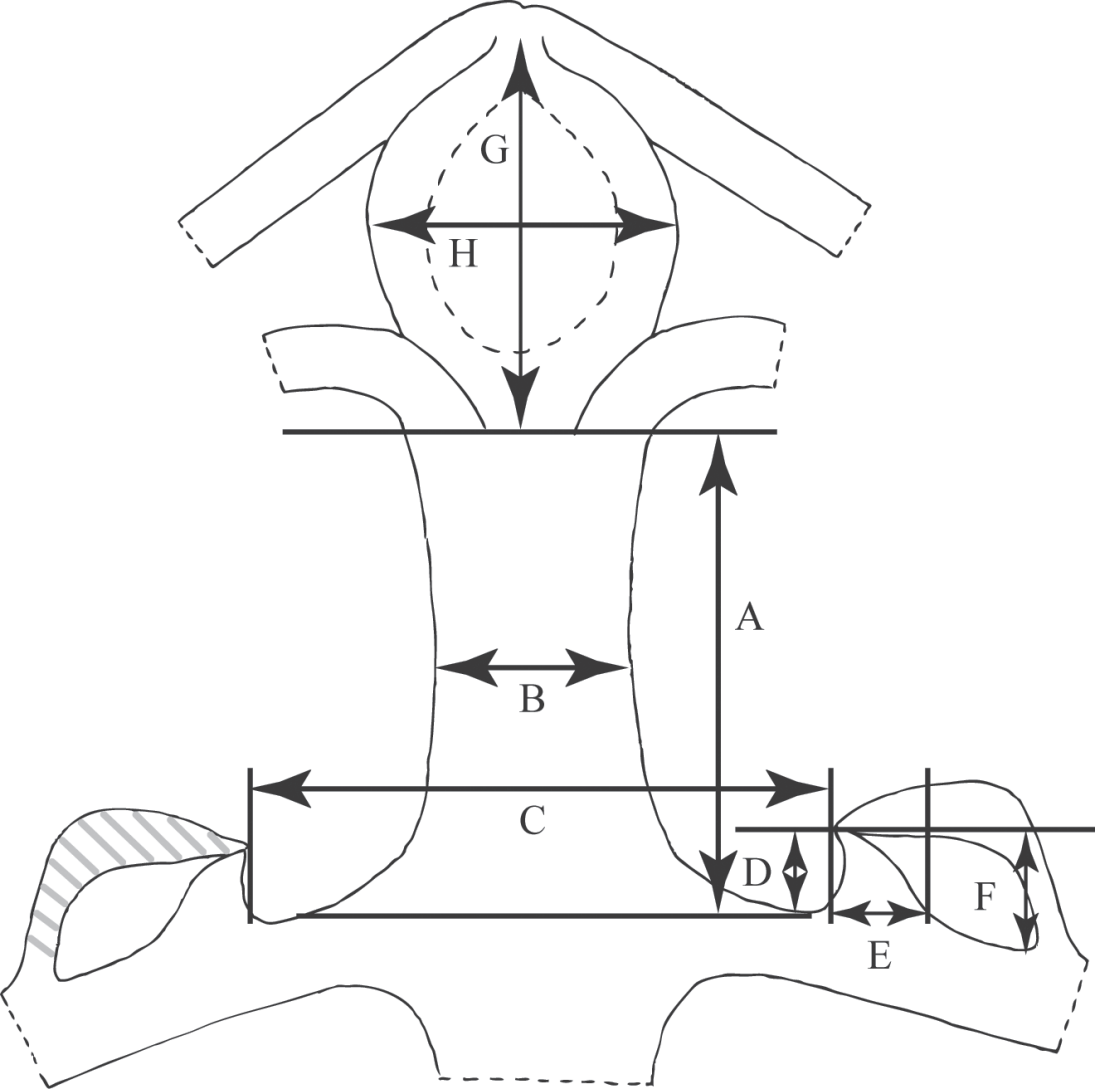
View of a longitudinal section through the flower of *Passiflora coriacea* demonstrating method of measurement. **a** Length of androgynophore **b** Width of androgynophore **c** Width of limen floor **d** Height of limen **e** Width of limen **f** Height of nectary **g** Length of ovary **h** Width of ovary. Note that the operculum is straightened and (shaded) measured from base to tip.

An average of 330 macromorphological characters were measured or observed on each of 95 plant specimens. All of the herbarium specimens representing supersection *Cieca* were carefully observed, and those spanning the morphological variation and geographical range of each species were chosen for measurement. Depending upon the material available, up to five measurements were taken for each quantitative character on each specimen. Characters were measured or scored from corresponding positions on mature, reproductive plants from throughout the geographical range of the supersection in order to minimize error due to developmental differences. Measurements of the dried leaves of the species in supersection *Cieca* were taken according to the conventions in Figs 1–3. The flowers were measured in accordance with the standards in Figs 4–6. Dried flowers from herbarium specimens were rehydrated by placing them in warm water with a wetting agent (Aerosol OT) or immersing them in concentrated ammonia (Toscano de Brito 1996; Taylor 1975). Color names used in this treatment follow the Munsell Color System (Long and Luke 2001). All drawings of flowers were made either from material fixed in standard FAA [70% ethyl alcohol (90%), glacial acetic acid (5%) and formalin (5%)] and preserved in 70% ethyl alcohol or from herbarium material that was expanded and softened.

Seventy quantitative characters were initially evaluated for the Neighbor Joining analysis of the *Passiflora suberosa* complex, but 25 were discarded due to lack of variability or lack of unambiguous gaps in the pattern of variation, making state delimitations difficult. Of the 45 remaining, six show no overlap in the range of variation of their states. The remaining 39 quantitative characters were utilized even though they exhibit some arbitrariness in state delimitation.

Seventy quantitative characters were initially evaluated for the Neighbor Joining analysis of the *Passiflora coriacea* complex, but 37 were discarded due to lack of variability or lack of gaps in the pattern of variation, making state delimitations difficult. Of the 33 remaining, 17 show no overlap in the range of variation of their states. The remaining 16 quantitative characters were utilized even though they exhibit some overlap between delimited states.

**Figure 7.**
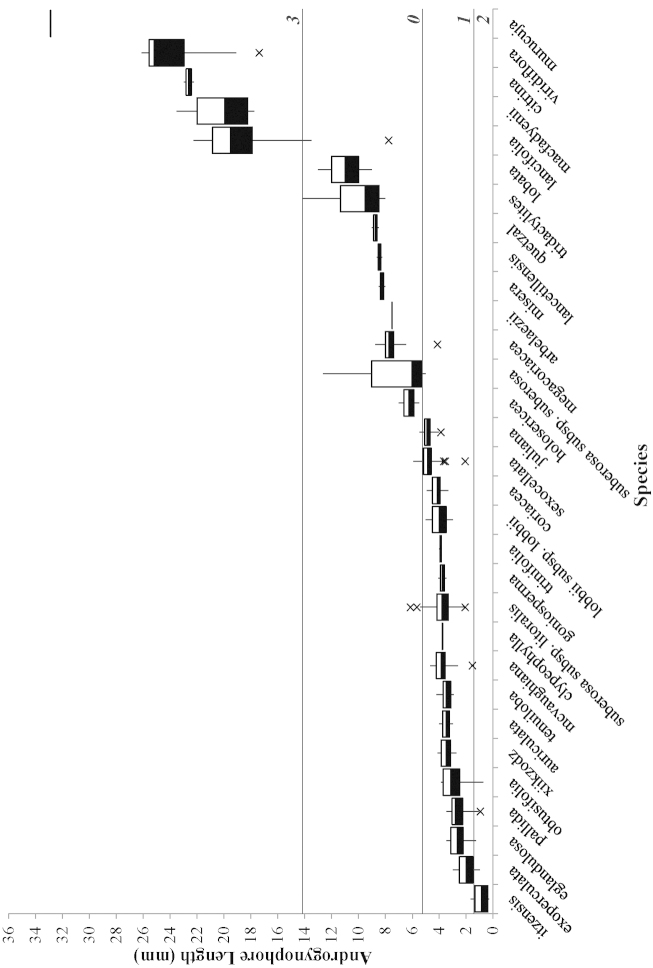
Box plots of character 18 (androgynophore length) used in the morphological cladistic analysis of Passiflora supersection Cieca. Bottom of black box = 1^st^ quartile; top of white box = 3^rd^ quartile; border between black and white box = median; top vertical line = greater of max value or 1.5 × (Q3-Q1); bottom vertical line = lower of min vlaue or 1.5× (Q3-Q1); × = outlier/s (value/s outside of 1.5×); horizontal lines extending the width of the graph = values used for the delimitation of the character into states; italicized and bolded numbers along right side of graph = assigned character states.

**Figure 8.**
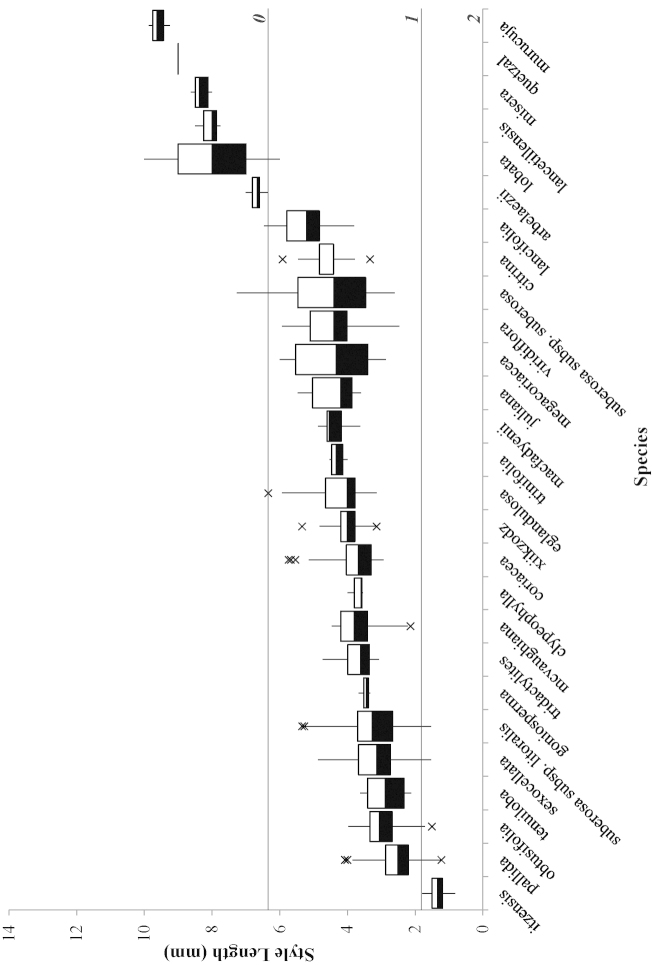
Box plots of character 15 (style length) used in the morphological cladistic analysis of Passiflora supersection Cieca. Bottom of black box = 1^st^ quartile; top of white box = 3^rd^ quartile; border between black and white box = median; top vertical line = greater of max value or 1.5 × (Q3-Q1); bottom vertical line = lower of min vlaue or 1.5× (Q3-Q1); × = outlier/s (value/s outside of 1.5×); horizontal lines extending the width of the graph = values used for the delimitation of the character into states; italicized and bolded numbers along right side of graph = assigned character states.

Seventy quantitative characters were initially evaluated for the cladistic analysis of the supersection, but 31 were discarded due to lack of variability or problems in delimiting character states. Of the 39 remaining, only one shows no overlap in the range of variation of its states (see for example Fig. 7). The remaining 38 quantitative characters were utilized even though they exhibit some overlap in the range of variation assigned to different character states between taxa (see for example Fig. 8). In all species descriptions, the flower diameters were mathematically determined: 2(sepal length) + hypanthium diameter.

Distribution maps were produced in ESRI® ArcMap^TM^ 10.0 (Environmental Systems Research Institute, Inc., Redlands, California, USA). Label data from herbarium specimens were used to determine the latitudes and longitudes employed in the construction of distribution maps. The gazetteer consulted for localities in the United States was the Geographic Names Information System (GNIS), developed by the U.S. Geological Survey (USGS) in cooperation with the U.S. Board on Geographic Names (US BGN). For international localities, the primary gazetteer consulted was the Geographic Names Database (http://www.nima.mil/geonames/GNS/index.jsp). The Geographic Names Database is on the GEOnet Names Server (GNS), the official repository of foreign place-name decisions approved by the United States Board on Geographic Names (US BGN). A secondary source for international localities was the Tageo Database of Geographic Coordinate Information. The coordinate system for data served by the GNIS, GNS and the Tageo Database of Geographic Coordinate Information is WGS84.

Principal components analyses (PCA) were produced using the computer program Multi-Variate Statistical Package (MVSP) 3.13d (Kovach Computing Services, Anglesey, Wales, UK). All measured quantitative characters for specimens in the *Passiflora suberosa* and *Passiflora coriacea* complexes were used in the analyses. Only those characters and specimens for which there was an abundance of missing data were deleted from the analyses. The mean values of measurements were used for each specimen. Box plots were produced using the computer program SPSS for Windows Release 11.5 (SPSS, Inc., Chicago, Illinois, USA). Neighbor joining trees were produced in PAUP* Version 4.0b10 for Macintosh (Sinaeur, Sunderland, Massachusetts, USA). Morphological characters were also analyzed cladistically, and cladistic methods are discussed under the heading “Phylogenetic Search Strategies”.

### Molecular data set

Total genomic DNA was extracted from fresh, heat dried, or silica dried leaves or flowers utilizing the CTAB method of Doyle and Doyle, scaled down to 1.0 ml extraction volumes (Doyle and Doyle 1987). Amplification of the internal transcribed spacer (ITS) region of 18S-26S nuclear ribosomal DNA (nrDNA) was performed using 50 µl reactions, 2.5 mmol/L MgCl_2_, 1.0 mol/L betaine, and a hot start at 94 °C for between 3 –10 minutes, using Epicentre (Epicentre Technologies, Madison, Wisconsin, USA) buffers and *Taq* polymerase. For material extracted from herbarium specimens, the above amplification protocol was modified by using 3.0 mmol/L MgCl_2_, no betaine, and a hot start at 99 °C for 30 seconds. A touchdown thermal cycling program was used for fresh and silica-dried samples. An initial denaturation at 94 °C for three minutes was followed by an initial annealing temperature of 76 °C (decreasing 1 °C per cycle for 17 cycles to 59 °C), extension at 72 °C for one minute and denaturation at 94 °C for one minute. This was followed by 22 cycles of annealing at 59 °C for one minute, extension at 72 °C for one minute, denaturation at 94 °C for one minute and a final extension at 72 °C for four minutes. Amplification and sequencing primers were those of Sun et al. (1994). For the herbarium material, an initial denaturation at 94 °C for two minutes was followed by ten cycles of annealing at 55 °C for 30 seconds, extension at 72 °C for 30 seconds and denaturation at 94 °C for 20 seconds. This was followed by 33 cycles of annealing at 55 °C, extension at 72 °C for one minute, denaturation at 94 °C for 20 seconds and a final extension at 72 °C for seven minutes. Amplification and sequencing primers were those of Blattner (1999). PCR products were cleaned using QIAquick columns (Qiagen, Santa Clarita, California, USA) and underwent dye terminator cycle sequencing with Applied Biosystems Inc. (ABI) (Foster City, California, USA) reagents (5 µL reactions). The ITS region for members of supersection *Cieca* was sequenced directly from the cleaned amplified product in the DNA Sequencing Core Facility on the University of Florida campus (DSEQ, UF) with ABI 377 and ABI 373A automated sequencers. Sequences were edited and assembled using the ABI software packages Sequence Navigator ™ Version 1.0.1 and Auto Assembler ™ Version 1.3.0 on an Apple PowerMac computer and aligned visually.

Selected cleaned PCR products were cloned into TOPO-TA Cloning® (Invitrogen, Carlsbad, California, USA) vectors according to the manufacturer’s instructions, except that the ligation reactions were halved. Transformation reactions were incubated in SOC broth (2.0% tryptone, 0.5% yeast extract, 10 mM NaCl, 2.5 mM KCl, 10 mM MgCl_2_-6H_2_O, 20 mM glucose) at 37 °C for one hour before being spread onto figs containing S-Gal™/LB Agar/Kanamycin Blend (Sigma, St. Louis, Missouri, USA) and incubated at 37 °C for 8–18 hours. Only large white colonies, representing potentially recombinant plasmids, were selected for amplification and sequencing.

### Phylogenetic search strategies

Two matrices were analyzed cladistically for this study: morphology (32 taxa including outgroups) and ITS sequence data (71 taxa including outgroups). The morphological character states were carefully delimited (see discussion under “Morphological Data Set”). Many characters are qualitative, and discrete states were delimited within quantitative characters by assessment of gaps in the pattern of variation (Stevens 1991). Multistate characters were considered to be unordered and ingroup/outgroup relationships were analyzed simultaneously. Outgroups from the subgenera *Decaloba* (supersections *Auriculata*, *Bryonioides*, *Decaloba*, *Hahniopathanthus*, *Multiflora*, and *Pterosperma*) and *Deidamioides* (section *Tryphostemmatoides*) were selected based on studies by two *Passiflora* specialists, C. Feuillet and J. M. MacDougal (Feuillet and MacDougal 2003); no modern cladistic analyses of the family or genus had been published at the time. Cladistic analyses were performed using PAUP* Version 4.0b10 for Macintosh (Sinauer, Sunderland, Massachusetts, USA) with all but one character equally weighted. In the morphological analysis, the absence of petals was given a weight of two merely to enforce the monophyly of the ingroup taxa, as is strongly supported by my DNA-based analysis. The morphological and molecular data sets were analyzed using the heuristic search option (MULTREES, SPR, 1000 random replicates, holding five trees per replicate, using the delayed transformation optimization).

Trees were evaluated on the basis of tree length, consistency index (CI), and retention index (RI) as calculated by PAUP*. Bootstrap consensus trees were generated for all data sets (1000 replicates). Congruence of the separate data sets was assessed by comparison of the tree statistics and topologies of the strict consensus trees.

### Species concepts

The phylogenetic species concept *sensu* Wheeler and Platnick was primarily employed in this study (Wheeler and Platnick 2000). However, other species concepts such as the biological species concept (Mayr 1942), phenetic species concept (Sokal and Crovello 1970) and autapomorphic concept (Donoghue 1985; Mishler 1985) were also considered and frequently proved useful. In the phenetic analyses of the *Passiflora suberosa* and *Passiflora coriacea* complexes, I looked for gaps in the pattern of variation and used sets of morphological characters in species delimitation. I also considered the inability to interbreed (through greenhouse studies conducted by J. M. MacDougal, unpublished data), along with other evidence (easily observed diagnostic morphological characters), as an indication that *Passiflora itzensis* (J.M. MacDougal) K. Port-Utl. is a distinct entity recognizable at the rank of species. In addition, nearly all of the species in supersection *Cieca* are cladospecies and possess molecular and morphological autapomorphies (Figs 19–12, 20–21).

## Morphology

### Habit

As noted by MacDougal (1994), the usual size or habit of passionflowers is seldom recorded by collectors and is poorly known for most species. This is especially true of the species in Passiflora subgenus Decaloba supersection Cieca; the plants are often small in stature and possess small flowers. According to my own field observations and notes from herbarium specimens the species in the supersection rarely reach a length greater than 8 m. They are perennial climbing or procumbent vines commonly found growing along forest edges.

### Stems

The pressing and drying which occurs during the making of herbarium specimens causes the stems of most exemplars to appear sulcate, however, observations of living and alcohol-preserved material show that the stems of the species in supersection *Cieca* are mostly terete; some species have stems that are slightly compressed. In subgenus *Decaloba*, the stem tip can be cernuous or more or less straight. The posture of the stem tip is likely an important taxonomic characteristic in the subgenus and is thought to be under selection by butterflies searching for ovipositioning sites (MacDougal 1994). In supersection *Cieca*, the apices are straight. The stems of all species in the supersection are antrorsely appressed-puberulent throughout, with small, unicellular, curved trichomes. Some species are also sparsely to densely pubescent with longer unicellular, rarely multicellular, curved trichomes.

### Stipules

In supersection *Cieca* the stipules are setaceous or narrowly triangular to foliaceous, but foliaceous stipules are found in only four species. The stipules that are setaceous or narrowly triangular have only one vein, but those with foliaceous stipules (particularly *Passiflora juliana* and *Passiflora eglandulosa*) possess 3–9. The stipule margins are always entire.

### Leaves

The laminas in supersection *Cieca*, as in the entire genus, are incredibly variable in shape. This is likely due to selection pressures from passionflower butterflies that visually search for particular leaf shapes when looking for ovipositioning sites. The leaves may be unlobed or 2-, 3-, or 5-lobed and often exhibit heterophylly (especially heterophyllous species are *Passiflora obtusifolia*, *Passiflora pallida*, *Passiflora suberosa*, and *Passiflora tenuiloba*). Several species are not heterophyllous and possess bi-lobed (e.g., *Passiflora itzensis*, *Passiflora tacanensis* K. Port.-Utl., and *Passiflora xiikzodz*) or tri-lobed (e.g., *Passiflora juliana*, *Passiflora lancifolia*, *Passiflora macfadyenii*, *Passiflora trinifolia*, and *Passiflora viridiflora*) mature leaves. *Passiflora tenuiloba*, the Texas longhorn, is the only species in the supersection that possesses leaves that have three or more leaf lobes; the primary lobes of the leaves may also have 2–4 smaller lobes. The venation pattern of the leaves in the supersection is usually palmate; even the unlobed leaves of *Passiflora pallida* possess palmate venation. The leaves may also be peltate (especially peltate species are *Passiflora coriacea*, *Passiflora juliana*, *Passiflora sexocellata*, and *Passiflora viridiflora*) or not. The first few leaves on the poorly known seedlings are usually peltate in most species. The margins of the leaves, however, are uniformly entire.

### Extrafloral nectaries

The leaves of supersection *Cieca* are simple and commonly bear functional nectaries on the petioles, though two species (*Passiflora eglandulosa* and *Passiflora mcvaughiana*) usually do not possess glands. Petioles are typically terete to slightly flattened. When glands are present on the petioles there are typically two and they are opposite, subopposite or alternate to one another. The glands are usually disc- or cup-shaped. Many species in supersection *Cieca* are characterized by disc-shaped petiolar nectaries that possess edges which are fused to the petiole (*Passiflora clypeophylla*, *Passiflora coriacea*, *Passiflora itzensis*, *Passiflora trinifolia*, *Passiflora viridiflora*, and *Passiflora xiikzodz*). Others possess cup-shaped nectaries that have raised edges that are not fused to the petiole (*Passiflora lancifolia*, *Passiflora macfadyenii*, *Passiflora pallida*, and Passiflora suberosa subsp. suberosa). Six species and one subspecies have individuals that possess either disc- or cup-shaped nectaries, but one type is more common than another. Passiflora suberosa subsp. litoralis is a widespread subspecies that more commonly possesses cup-shaped nectaries, but there are examples of this species (especially from the South America) that possess disc-shaped nectaries. In *Passiflora juliana*, *Passiflora megacoriacea*, *Passiflora mcvaughiana*, *Passiflora obtusifolia*, *Passiflora sexocellata*, and *Passiflora tenuiloba*, disc-shaped nectaries are much more frequent. The positioning of the nectaries can also vary. Most species have nectaries that are found only on the distal half of the petiole (*Passiflora itzensis*, *Passiflora lancifolia*, *Passiflora macfadyenii*, *Passiflora mcvaughiana*, *Passiflora megacoriacea*, *Passiflora obtusifolia*, *Passiflora pallida*, *Passiflora tenuiloba*, *Passiflora tridactylites*, and *Passiflora xiikzodz*). Others have nectaries that are on the proximal half of the petiole (*Passiflora juliana*, *Passiflora sexocellata*, *Passiflora tacanensis*, and *Passiflora viridiflora*). *Passiflora suberosa* and *Passiflora trinifolia* are the only two species that can possess nectaries in a variety of positions on the petiole.

Functional laminar nectaries are also present in many species of the supersection. In those species that possess them they occur as submarginal glands associated with minor veins of the abaxial surface. The glands are discoid and slightly raised. The absence of laminar nectaries is characteristic of *Passiflora eglandulosa*, *Passiflora lancifolia*, *Passiflora macfadyenii*, *Passiflora mcvaughiana*, *Passiflora pallida*, and *Passiflora tacanensis*.

### Inflorescence

Shawn Krosnick (2005) has produced the most modern interpretation of the structure of the inflorescence and the evolution of its various forms in the Passifloraceae; her interpretation follows Troll (1964) and Cussett (1968). Basically, the inflorescence is an axillary compound cyme. Various parts of this inflorescence, however, have been reduced. For example, in supersection *Cieca* and many other species in the family, the peduncle is completely reduced, giving rise to a sessile inflorescence. The first order axis of the sessile cyme terminates in a tendril and the second-order side branches terminate in flowers. The prophylls of the first order axis are displaced onto the branches that they normally subtend (Cussett 1968). In most other species in the family, one of the first order prophylls and the two second-order side branch prophylls are retained on each second-order side branch, giving rise to 3-bracteate pedicels collateral with the tendril in the axil of the leaf. Supersection *Cieca* is unique in that usually none of the prophylls of the first and second-order side branches are retained on the second-order side branches, giving rise to ebracteate pedicels collateral with the tendril in the axil of the leaf. In some species, however, one prophyll (likely from the first order axis) or two prophylls (likely one from the first order axis and one from the second order side branch) are retained on the pedicels. Thus, species of supersection *Cieca* usually have no floral bracts, or up to two; no species have three, as in most *Passiflora*. When present the bracts are setaceous and, in some cases, are quickly deciduous.

### Flowers

The flowers in supersection *Cieca* are apetalous and erect or, rarely, positioned horizontally; very rare occurances of one or two well-positioned petals in the otherwise apetalous flowers have been observed (e.g., Passiflora suberosa subsp. litoralis and *Passiflora itzensis*). Most flowers are greenish yellow in color with purplish to reddish markings; two species (*Passiflora lancifolia* and *Passiflora macfadyenii*) possess red flowers. The flowers are small, rarely exceeding 3 cm in diameter; most species possess flowers that are less than 2 cm wide. Most are bowl- or saucer-shaped, but three species (*Passiflora lancifolia*, *Passiflora macfadyenii* and *Passiflora viridiflora*) are tubular.

### Hypanthium

The hypanthium is the portion of the flower that holds the nectary and associated structures (the operculum and limen) at its base and bears the perianth, corona, and androgynophore. The hypanthium in the flowers of supersection *Cieca* is patelliform or dishlike and is less than 3 mm deep, with most species possessing a hypanthium that is less than 1 mm in depth. The diameter of the hypanthium is commonly 5–8 mm. *Passiflora pallida* has the smallest hypanthium diameter (<4 mm) and *Passiflora megacoriacea* the largest (9–13 mm).

### Sepals

The species in supersection *Cieca* possess five, ovate triangular sepals. In most taxa, the sepals are greenish yellow on their outer surfaces, though in *Passiflora lancifolia* and *Passiflora macfadyenii* they are red. Adaxially the sepals are greenish yellow (e.g., Passiflora suberosa subsp. litoralis, *Passiflora juliana*, *Passiflora mcvaughiana*), red (*Passiflora lancifolia* and *Passiflora macfadyenii*), or rarely whitish (e.g, Passiflora suberosa subsp. suberosa). The sepals are distinct, except in the tubular flowers of *Passiflora macfadyenii* and *Passiflora viridiflora* where they are partially connate. In most species, the sepals are reflexed at anthesis. The sepals are, on average, 8 mm in length in saucer-shaped flowers and 15 mm in length in tubular flowers.

### Corona

In supersection *Cieca*, the corona is mostly in 2 series, a shorter inner series and a (often) much longer outer series. In two species, *Passiflora itzensis* and *Passiflora xiikzodz*, the corona is in 7 series; the outer two rows are the longest and the other inner rows are much shorter and nearly equal in form. Occasionally, individuals in the *Passiflora suberosa* complex (sensu latu) lack an inner coronal row.

The filaments in the outer coronal row are terete, sometimes very slightly capitate, and have a base color of greenish yellow, or purple to red (sometimes very dark reddish purple). Greenish yellow filaments often have purplish to reddish spots and streaks and may be tipped with bright yellow or white. Red filaments are often uniform in color or possess yellowish tips. The orientation of the outer coronal row is commonly bowl-shaped at anthesis, but may be more or less erect or reflexed and flat.

The inner coronal filments are usually less than half the length of the outer coronal filaments and are capitate. The inner coronal filaments are commonly greenish yellow, purplish, or red and have lighter-colored tips. As in the outer coronal row, the greenish yellow filaments may possess reddish or purplish spots and/or streaks. The orientation of the inner filaments is frequently erect.

### Operculum

The operculum is considered to be the innermost coronal row in the genus *Passiflora*. The function of the operculum, however, is generally not to attract pollinators but to cover and protect the floral nectary. In supersection *Cieca*, the operculum is membranous and plicate or, in the nectarless *Passiflora itzensis* and *Passiflora xiikzodz*, denticulate. The operculum is curved over the nectary and commonly touches the tip of the limen or, particularly in tubular flowers, completely covers both the nectary and limen and leans against the androgynophore.

### Nectary

The nectary is positioned at the base of the hypanthium and is a trough that is covered by the operculum. In many species of *Passiflora*, there is a raised ring (sensu Jorgensen et al., 1974) or annulus (sensu Tillett 1988) in the nectary trough. A nectar ring or annulus is lacking or very inconspicuous in the species of supersection *Cieca*; however, in *Passiflora tenuiloba* the nectary is sulcate. The development and physiology of the floral necatary of *Passiflora eglandulosa* [as *Passiflora trinifolia*] was examined by Durkee et al. (1981). She found that the floral nectary development and nectar secretion in this species was similar to that in two other species of *Passiflora* (*Passiflora warmingii* and *Passiflora biflora*) that she studied. The activity of an intercalary meristem in the nectary and increased starch deposition in the amyloplasts of the secretory cells parallels the maturation of the nectary phloem and serves as the main source of nectar sugars at anthesis. Though she did not measure the sugar concentration of *Passiflora eglandulosa*, she found that in the other two species in her study the dominant sugar constituent of the nectar was sucrose, with fructose present only in moderate amounts; nectar sugar concentrations are presented below in the discussion on reproductive biology (Durkee et al. 1981).

### Limen

The limen is a structure that is situated between the nectary and the androgynophore. It is widely considered to be of staminodal origin and, along with the operculum, helps to protect the nectary (Killip 1938; Puri 1948; deWilde 1974). The limen in supersection *Cieca* is adnate to the hypanthium with only its outer edge free. The edge is commonly erect and inclined toward the nectary, though in some species it is curved toward the androgynophore. It commonly has a base color of greenish yellow, white, purple or red. When greenish yellow or white, it often possesses reddish spots and/or streaks.

### Androgynophore

The androgynophore is a central column in the flower which consists of an elongate gynophore surrounded by and fused to the staminal filaments. It is straight in all species of supersection *Cieca*. It is greenish yellow and often possesses reddish or purplish spots or streaks. It is generally less than 5 mm in height, though tubular species possess androgynophores that reach heights of 25 mm.

### Androecium

The androecium in supersection *Cieca* is very uniform. There are five greenish yellow filaments with versatile and dorsifixed anthers. In most species the anthers are introrse in bud but flip over and are extrorse at dehiscence. In these species, the long axis of the anthers remains parallel to the long axis of the filaments or, rarely, the long axis of the anthers are perpendicular (or nearly so) to the long axis of the filaments. Rarely, the anthers only move slightly from the original introrse position, remain introrse, and dehisce distally (upwards). The pollen is commonly yellow in color; however, in Passiflora suberosa subsp. suberosa the pollen is light yellow or whitish in color.

### Gynoecium

The ovary of three carpels is commonly ellipsoid to globose in shape; few species possessing fusiform (*Passiflora macfadyenii* and *Passiflora tridactylites*) ovaries. The ovary has one locule and the placentation is parietal. The ovary possesses a small stipe that extends no more than a millimeter above the adnation of the staminal filaments. It is more or less glabrous and greenish yellow in color.

The styles are slender and free to the base and may be straight or curved. They have a base color of greenish yellow but may possess reddish or purplish spots and/or streaks. The stigmas are depressed ovoid and greenish yellow to whitish in color.

### Fruits

The fruits in the supersection are small (commonly less than 2 cm long) berries that contain one (rarely) to many (80) seeds. Mature fruits are purple or very dark purple with a very thin pericarp. Often the epidermis has a glaucous bloom.

### Seeds

The seeds in supersection *Cieca* are compressed and often beaked at the chalazal apex. The sculpturing of the seeds is reticulate-foveate. Most species possess 20–30 seeds per fruit. The species that have the fewest (<10) seeds per fruit are *Passiflora eglandulosa*, *Passiflora mcvaughiana*, and some species of *Passiflora pallida* and Passiflora suberosa subsp. litoralis. The species that commonly possess more than 40 seeds per fruit are *Passiflora coriacea*, *Passiflora juliana*, *Passiflora sexocellata*, and *Passiflora viridiflora*. Each pale brown to dark brown seed is surrounded by a fleshy aril that is somewhat translucent; the aril usually covers only ¾ of the seed. The arils that I have tasted are either very mildly sweet or sour.

## Chromosome numbers

All of the published chromosome counts of the species of Passiflora supersection Cieca support n = 6 as the base chromosome number (Beal 1969, 1971; Diers 1961; Melo et al. 2001; Melo and Guerra 2003; Snow and MacDougal 1993; Storey 1950; Turner and Zhao 1992). Snow and MacDougal (1993) documented 2n = 12 for *Passiflora itzensis*, *Passiflora juliana*, *Passiflora obtusifolia*, *Passiflora sexocellata* and *Passiflora xiikzodz*. Turner and Zhao (1992) found the chromosome number of *Passiflora tenuiloba* to be 2n = 12, and Diers (1961) found the same number for *Passiflora coriacea*. Beal (1971) documented n = 6 in *Passiflora sexocellata*.

*Passiflora pallida* and *Passiflora suberosa* are the only known polyploids in supersection *Cieca*. Snow and MacDougal (1993) found that *Passiflora pallida* from Jamaica was a polyploid (tetraploid) with a chromosome number of 2n = 24. Beal (1971) determined that Passiflora suberosa subsp. litoralis from both coastal Argentina and New Guinea had a chromosome number of 2n = 24. He also counted the chromosomes (2n = 24) of a plant of the subspecies from the “U.S.A.”, but the locality seems questionable based upon the morphology of the voucher. In addition, Passiflora suberosa subsp. litoralis does not occur in the wild in the United States, but it is commonly cultivated there. He also found the same chromosome numbers for three clones of Passiflora suberosa subsp. litoralis collected in Australia (Beal 1969, 1971). Diers (1961) found the diploid chromosome number of 2n = 12 in Passiflora suberosa subsp. litoralis from Lomas de Lachay, Perú. However, I have not been able to locate his voucher specimens (Diers 1961). Storey (1950) also counted the chromosomes of Hawaiian material, which he called *Passiflora suberosa*. However, I was unable to locate his vouchers and because Passiflora suberosa subsp. suberosa and Passiflora suberosa subsp. litoralis both occur in the Hawaiian Islands, I cannot be certain which subspecies he sampled. However, he did find chromosome numbers of 2n = 24 and 36 in wild populations of the species. He determined that the form with 36 chromosomes was likely an autotriploid derivative of the 24 numbered form. He did not describe the plants that he sampled, but he noted that there were no conspicuous morphological differences between the two chromosomal races. He only found that the triploid race had slightly larger leaves and more anthocyanin pigmentation in the young stems and abaxial surfaces of the sepals (Storey 1950).

## Chemistry

The Passifloraceae are cyanogenic, along with over 110 families of flowering plants; however, the family is noteworthy in possessing cyanogenic glycosides with a cyclopentene moiety. Cyclopentene cyanogens have been found only in other families within the Malpighiales and in the Caricaceae (Brassicaceae) (Spencer, 1988). Spencer (1988) surveyed for cyanogenesis in over 570 accessions of *Passiflora*, and he found that the different types of cyclopentene cyanogenic compounds are nonrandom and taxonomically significant. He found that the two members of supersection *Cieca* that he tested (*Passiflora coriacea* and *Passiflora suberosa*) produce unique cyclopentene cyanogens that make them unique in subgen. *Decaloba* – epipassicoriacen, epipassisuberosin, passicoriacen, and passisuberosin (Spencer 1987a, 1987b).

In 1982, McCormick studied the flavonoid chemistry of Passiflora subgenus Decaloba and analyzed several members of Passiflora supersection Cieca. Flavonol 3-O-glycosides are limited to a few groups of subg. *Decaloba* (supersects. *Bryonioides* and *Hahniopathanthus* and sect. *Xerogona*) (MacDougal, 1994). She found detectible levels flavonol 3-O-glycosides in dried leaf samples of *Passiflora juliana*, *Passiflora macfadyenii*, and *Passiflora viridiflora*. In other species, however, they were lacking (*Passiflora mcvaughiana*, *Passiflora obtusifolia*, and *Passiflora tenuiloba*) or found in only trace amounts (*Passiflora eglandulosa*). Though *C*-glycosylflavones are prevalent in the genus (di-*C*-glycosylflavones are characteristic of subg. *Decaloba*) they are completely lacking in all the species of supersection *Cieca* examined (*Passiflora eglandulosa*, *Passiflora juliana*, *Passiflora macfadyenii*, *Passiflora mcvaughiana*, *Passiflora obtusifolia*, *Passiflora tenuiloba*, and *Passiflora viridiflora*) (McCormick 1982).

## Reproductive biology

The pollinators of only three species of supersection *Cieca* have been recorded: *Passiflora sexocellata*, pollinated by small to medium guild bees (a species of *Colletes* Latr.; R. Clinebell, pers. comm.); Passiflora suberosa subsp. litoralis, pollinated by wasps (a species of *Polistes* Latr.) (Koschnitzke and Sazima 1997); and *Passiflora viridiflora*, pollinated by hummingbirds (MacDougal 1992; label data from herbarium specimens collected by W. L. Foment - *Foment 1125*).

With regard to breeding systems, most species in supersection *Cieca* are self-incompatible. MacDougal, in controlled greenhouse studies (from unpublished data; MacDougal 1992), found that *Passiflora itzensis* (*MacDougal 4633*; MacDougal 1992), *Passiflora juliana* (*MacDougal 492GR*; MacDougal 1992), *Passiflora macfadyenii* (*MacDougal 452*), *Passiflora mcvaughiana* (*MacDougal 369*), *Passiflora megacoriacea* (*MacDougal 409*), *Passiflora tenuiloba* (*MacDougal 227*) *Passiflora trinifolia* (*MacDougal 637*), *Passiflora viridiflora* (*MacDougal 351GR*) and *Passiflora xiikzodz* (*MacDougal 4677*; MacDougal 1992) are not self-compatible. *Passiflora eglandulosa* (*MacDougal 316*), *Passiflora pallida* (*MacDougal 259*) and Passiflora suberosa subsp. suberosa (*MacDougal 421*) are, however, often self-compatible; *Passiflora pallida* and Passiflora suberosa subsp. suberosa are also autogamous. Passiflora suberosa subsp. litoralis, however, has been found to be mostly self incompatible. Two clones [New Caledonia (*MacDougal 438*) and Guadalajara, Mexico (*MacDougal 438*)] of this species from New Caledonia, however, did prove to be self-compatible. Koschnitzke and Sazima (1997) found that, in Brazil, the flowers of Passiflora suberosa subsp. litoralis are also self-compatible. MacDougal found that one clone of aff. Passiflora suberosa ssp. litoralis (*MacDougal 1486*) was self-incompatible in the greenhouse. Two clones of *Passiflora obtusifolia* (*MacDougal 495GR* and *MacDougal 4687*) did not set fruit over several years of cultivation at the University of Florida or at Missouri Botanical Garden.

It is possible to cross several species of supersection *Cieca*. MacDougal, in controlled greenhouse studies (from unpublished data – see voucher numbers above), successfully crossed: 1) *Passiflora tenuiloba* with *Passiflora juliana*, *Passiflora megacoriacea*, *Passiflora pallida*, and *Passiflora trinifolia*, 2) *Passiflora eglandulosa* with *Passiflora viridiflora*, 3) *Passiflora viridiflora* with *Passiflora eglandulosa*, *Passiflora macfadyenii*, *Passiflora megacoriacea*, *Passiflora mcvaughiana*, *Passiflora pallida*, *Passiflora trinifolia*, and 4) *Passiflora mcvaughiana* with *Passiflora megacoriacea*, *Passiflora viridiflora*, *Passiflora trinifolia*, *Passiflora pallida*, and Passiflora suberosa subsp. suberosa, and 5) *Passiflora megacoriacea* with *Passiflora mcvaughiana*, *Passiflora viridiflora*, *Passiflora tenuiloba*, and Passiflora suberosa subsp. suberosa.

The fruits of supersection *Cieca* are unilocular berries with thin pericarps that are very dark purple, sometimes with a glaucous bloom. They may contain one (rarely) to many arillate seeds, with the arils mostly clear to slightly opaque and covering one half to three quarters of the seed. The fruits also persist on the pedicels for some time after maturity. Van der Werff (*van der Werff 1951 and 1420*) reported that finches eat the fruits of *Passiflora tridactylites* and Passiflora suberosa subsp. litoralis in the Galapagos Islands. Clifford Smith (Univ. of Hawaii) has found that the seeds of *Passiflora suberosa* are dispersed by alien frugivorous birds in Hawaii (http://www.botany.hawaii.edu/faculty/cw_smith/pas_sub.htm). The Mariana fruit bat, *Pteropus mariannus mariannus* Desmarest, is known to feed on the fruits of *Passiflora pallida* on Guam. *Passiflora pallida* is a weedy vine there and will grow up into and cover the canopies of forest trees species, especially in disturbed habitats. Feeding by the Mariana fruit bat occurs mostly when the vines grow up in the tops of trees and the bat lands in the tree to feed (Dustin Janeke, pers. comm.; http://www.passionflow.co.uk/bats11.htm).

## Herbivory

Species of *Passiflora* are of particular interest to entomologists, as these plants are larval hosts for passion flower butterflies (Subfamily Heliconiinae, Family Nymphalidae). Larvae of the subfamily are almost uniquely restricted to food plants in the Passifloraceae, giving rise to the name “passion flower butterflies.” The close association of species in the Heliconiinae and Passifloraceae is commonly held up as an example of plant-insect coevolution.

Most of the species of Passiflora supersection Cieca are utilized by common and widespread species of the subfamily Heliconiinae. The known butterfly herbivores of species of supersection *Cieca* are listed in Table 1. Most species in the supersection have only one or two known herbivores, but, as one would expect, the species that are widely distributed have a greater diversity of herbivores. The more derived species of supersection *Cieca* (*Passiflora xiikzodz*, *Passiflora juliana*, *Passiflora viridiflora*, *Passiflora coriacea*, *Passiflora sexocellata*, *Passiflora megacoriacea*) are mainly used by species of *Heliconius*. The early branching species in the supersection (*Passiflora eglandulosa*, *Passiflora lancifolia*, *Passiflora pallida*, and Passiflora suberosa subsp. litoralis) are also commonly utilized by *Heliconius* spp. but are also hosts for early branching genera of the Heliconiinae (*Acraea*, *Agraulis*, *Dione*, *Dryas*, *Dryandula* and *Philaethria*). *Passiflora tenuiloba*, which is sister to *Passiflora pallida* in the morphological analysis but is more closely related to *Passiflora coriacea* and *Passiflora sexocellata* in the molecular analysis, serves as a host for *Agraulis* and *Dryas* and the more derived genus *Heliconius*.

**Table 1.** Herbivore records of Heliconiinae for the species of Passiflora supersection Cieca; “-” = taxa not included in the molecular analysis.

Species	Heliconiinae	Place	Reference
*Passiflora coriacea*	*Heliconius erato* Linnaeus, 1758	Central Colombian Valleys and N Venezuela	Benson et al. 1975
*Passiflora eglandulosa*	*Heliconius hortense* Guérin-Méneville, 1844	N Central America	MacDougal 1988; Benson et al. 1975
*Passiflora juliana*	*Heliconius charitonia* Linnaeus, 1767	Mexico	MacDougal 1992
*Passiflora lancifolia*	*Dryas iulia* Fabricius 1775	Jamaica	Benson et al. 1975
*Passiflora megacoriacea*	*Heliconius cydno* H. Bates, 1864 *Heliconius erato*	Panama and N Costa Rica Costa Rica	Benson et al. 1975 DeVries 1987
*Passiflora obtusifolia*	*Heliconius charitonia*	Mexico	J. M. MacDougal and J. Miley 495
*Passiflora pallida*	*Acraea andromacha* Fabricius, 1775 *Agraulis vanillae* Linnaeus, 1758 *Dione juno* Cramer, 1779 *Dryas iulia* *Euptoieta claudia* Cramer, 1776 *Euptoieta hegesia* Cramer, 1779 *Heliconius charitonia*	Australia Jamaica Peru Jamaica Florida, USA Texas, USA Jamaica	Hawkeswood 1991 Benson et al. 1975 Benson et al. 1975 Benson et al. 1975 Minno and Minno 1999 P.Schappert, pers. comm. Benson et al. 1975
*Passiflora sexocellata*	*Dryas iulia* *Heliconius erato*	Mexico and N Central America Mexico and N Central America	Benson et al. 1975 Benson et al. 1975 Meerman 2001
Passiflora suberosa subsp. litoralis	*Agraulis vanillae* *Dione juno* *Dryandula phaetusa* Linnaeus, 1758 *Dryas iulia* *Heliconius charitonia* *Heliconius erato* *Heliconius sara* Fabricius, 1793 *Philaethria wernickei*	SE Brazil Peru S Brazil Peru, SE Brazil Peru SE Brazil SE Brazil SE Brazil	Benson et al. 1975 Benson et al. 1975 Benson et al. 1975 Benson et al. 1975 Benson et al. 1975 Benson et al. 1975 Benson et al. 1975 Benson et al. 1975
*Passiflora tenuiloba*	*Agraulis vanillae* *Dryas iulia* *Heliconius charitonia*	Texas, USA Texas, USA Texas, USA	Benson et al. 1975 M. Quinn, pers. comm. L. Gilbert, pers. comm.
*Passiflora viridiflora*	*Heliconius charitonia*	Mexico	MacDougal 1983
*Passiflora xiikzodz*	*Heliconius erato*	Belize	Meerman 2001

## Distribution and habitats

Species of supersection *Cieca* are found from Florida and southern Texas in the United States of America, through Mexico and Central America, from Colombia and Venezuela to Argentina and southern Brazil, and in the Caribbean; they are absent from the Guyana Shield region. *Passiflora pallida* and *Passiflora suberosa* are also found in many areas of the Old World tropics and on many north and south Pacific islands to the east of the International Date Line, as the result of introduction by humans. However, the center of diversity is in southern Mexico and northern Central America.

Of the 19 species recognized here, five species (*Passiflora juliana*, *Passiflora viridiflora*, *Passiflora mcvaughiana*, *Passiflora tacanensis* and *Passiflora itzensis*) are endemic to Mexico, two to Guatemala (*Passiflora clypeophylla* and *Passiflora trinifolia*), two to Jamaica (*Passiflora lancifolia* and *Passiflora macfadyenii*), and one to the Galapagos Islands, Ecuador (*Passiflora tridactylites*). *Passiflora juliana* and *Passiflora viridiflora* are both found along the Pacific coast and in the Pacific coastal plain of southwestern Mexico in disturbed tropical deciduous or semideciduous forests of low to moderate elevation (Fig. 43). These two species are not sympatric, with *Passiflora juliana* found farther north, from areas around the Bahia Chamela in Jalisco to those just south of Manzanillo in southern Colima, and *Passiflora viridiflora* occurring from regions just north of Lazaro Cardenas in Michoacan to areas around the Gulf of Tehuantepec in southern Oaxaca. *Passiflora itzensis* is found in tropical semideciduous forests from areas near Chichen Itza in Yucatán to localities in southern Quintana Roo north of Chetumal (Fig. 55). *Passiflora mcvaughiana* is also found in southwestern Mexico, in high elevation oak, pine/oak or pine forests or montane mesophytic forests on moist hillsides and in barrancas (Fig. 46). *Passiflora tacanensis* is known only from three collections in a high altitude tropical montane forest on Volcán Tacaná in Chiapas, Mexico along the border with Guatemala (Fig. 46). *Passiflora clypeophylla* has not been found since the type was collected in 1889 (Fig. 39). Based upon locality information included on the herbarium specimens and information gathered by J. M. MacDougal (pers. comm.) on a recent trip to the type locality, *Passiflora clypeophylla* is a plant of moderate elevation (ca. 1115 m. alt.) and is (or was) likely found on slopes of premontane tropical moist forest. *Passiflora trinifolia* is a rare plant found on cliffs and rocks in open, strongly seasonally dry pine and oak forests in northeastern Baja Verapaz, Guatemala (Fig. 39). *Passiflora macfadyenii* was last collected in 1979 and repeated attempts to find the plant by myself, Elma Kay (Missouri Botanical Garden), and George Proctor (Institute of Jamaica) have failed. It has been found in tropical dry forests in roadside thickets and wooded limestone hills in the parishes of St. Andrew and St. Thomas (Fig. 32). *Passiflora lancifolia*, another Jamaican endemic of supersection *Cieca*, is found in tropical lower montane mist forests on steep wooded hillsides in the Blue Mountains (Fig. 32). *Passiflora tridactylites* is an endemic of the Galapagos and grows in dry tropical forests at altitudes ranging from sea level to 800 m (Fig. 30).

Many of the remaining species of the supersection have wider geographic ranges in Mexico, Central America, and South America. *Passiflora tenuiloba* is a plant occurring in arid and semiarid thorn scrub and grasslands from southern Texas to northern Mexico (Fig. 35). *Passiflora xiikzodz* is found in the same habitats as its sister *Passiflora itzensis*, but in addition to being found in the Yucatán Peninsula of Mexico, its range extends to Belize and Guatemala (Fig. 55). *Passiflora sexocellata* is found from southern Mexico to Nicaragua (Fig. 52). Throughout its range this species is found in low, moist to wet tropical forests near streams and rivers, but, in the state of Veracruz, Mexico, it can be found growing on seaside cliffs. *Passiflora megacoriacea* is found in Costa Rica and Panama. In the northwestern corner of Costa Rica, in the province of Guanacaste, this species is deeply trilobed and occurs in the premontane transitional belt between the dry tropical forests typical of the Cordillera de Guanacaste and wetter mid-elevation forests (Fig. 50). Throughout the remainder of its range it is found at lower elevations in dry to wet tropical forests inland and near the sea along the Atlantic and Pacific coasts. *Passiflora coriacea* is found from northern Colombia and northwestern Venezuela to northern Bolivia (Fig. 49). It occurs in moist to wet tropical forests commonly at elevations of 50–1500 m, reaching higher elevations in the northern part of its range. *Passiflora obtusifolia* is found from sea level to 1500 m elevation in tropical deciduous and semideciduous forests in the Pacific lowlands and foothills of southwestern Mexico, El Salvador, and Costa Rica; it is found at higher elevations of 500–1500 m in the southern part of its range (Fig. 39). *Passiflora eglandulosa* occurs in shady ravines and at the edges of premontane to montane (1500–2800 m) broad leaved forests on volcanic cones from Guatemala to El Salvador and central Honduras (Fig. 37).

The species in supersection *Cieca* with the widest ranges are *Passiflora pallida* and *Passiflora suberosa*. *Passiflora pallida* has a circum-Caribbean distribution and is found in and along the edges of low elevation, dry tropical forests both inland and near the seashore (Fig. 24). This species has also been introduced into the areas of the Old World such as Australia, the Northern Mariana Islands, Comoros, Micronesia, India, Madagascar, Maldives, Mauritius, Palau, the Seychelles, Singapore, the Solomon Islands, and Sri Lanka. Passiflora suberosa subsp. suberosa is primarily restricted to the islands of the Greater and Lesser Antilles and is found in and along the edges of semideciduous to deciduous, dry to moist tropical forests, both inland and near the seashore, from sea level to 1600 m, but it has also been collected in the Hawaiian Islands, where it is introduced (Fig. 26). In the Greater Antilles, Passiflora suberosa subsp. suberosa is commonly found in and along the edges of moist forests, primarily at higher elevations. It is relatively common on all of the islands of the Greater Antilles, except for Jamaica where it is very rare. In the Bahamas and the Lesser Antilles, it does occur at high elevations but primarily occurs at lower elevations and is found in dry to moist forests. Passiflora suberosa subsp. litoralis has the widest geographic range of the taxa in supersection *Cieca* (Fig. 28). It grows in and along the edges of semideciduous to deciduous, dry to moist tropical forests and in secondary successional areas, both inland and near the seashore, from sea level to 2800 m, from northern Mexico, through Central America, to central Argentina and Brazil. In the Old World tropics it has been introduced in Australia, Fiji, New Caledonia, India, Indonesia, South Africa, Spain, Sri Lanka, Taiwan, and Uganda.

## Results

### Molecular analyses of supersection *Cieca*

**Figure 9.**
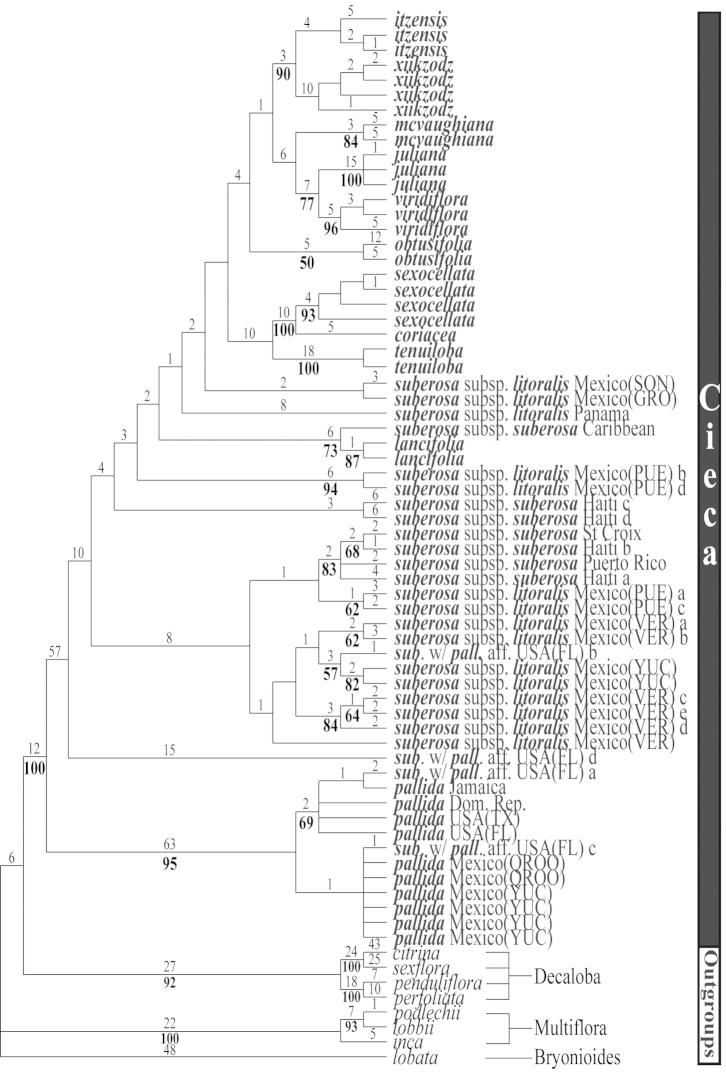
The first of three most parsimonious trees from the ITS-1 and ITS-2 data set of Passiflora supersection Cieca and outgroups. Numbers above branches are branch lengths. Bootstrap values are given below corresponding branches. Tree length = 590; CI = 0.636; RI = 0.837; RC = 0.532. Names followed by the letters “a”, “b”, “c”, “d”, and “e” denote clones of the same individual from a particular locality.

**Figure 10.**
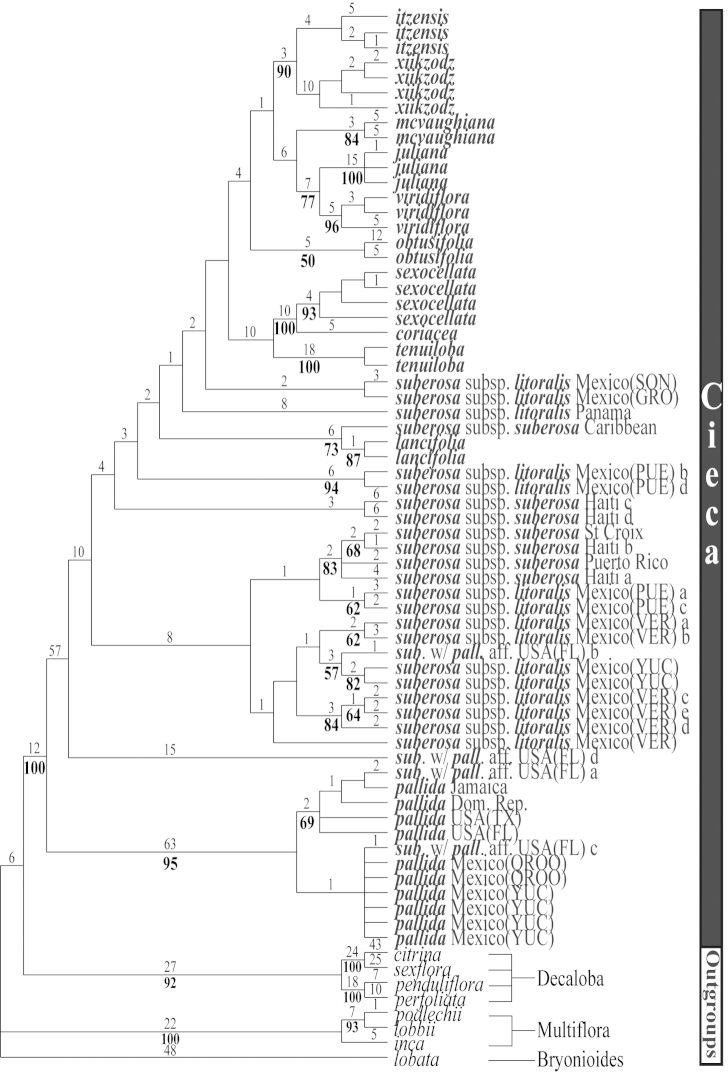
The second of three most parsimonious trees from the ITS-1 and ITS-2 data set of Passiflora supersection Cieca and outgroups. Numbers above branches are branch lengths. Bootstrap values are given below corresponding branches. Tree length = 590; CI = 0.636; RI = 0.837; RC = 0.532. Names followed by the letters “a”, “b”, “c”, “d”, and “e” denote clones of the same individual from a particular locality.

**Figure 11.**
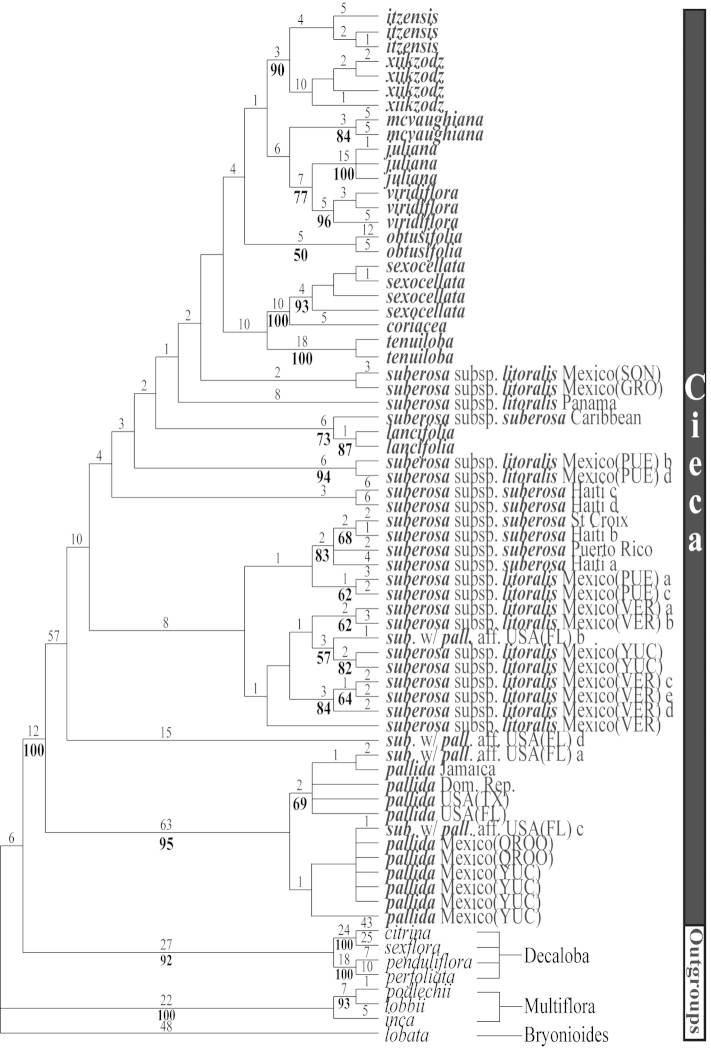
The third of three most parsimonious trees from the ITS-1 and ITS-2 data set of Passiflora supersection Cieca and outgroups. Numbers above branches are branch lengths. Bootstrap values are given below corresponding branches. Tree length = 590; CI = 0.636; RI = 0.837; RC = 0.532. Names followed by the letters “a”, “b”, “c”, “d”, and “e” denote clones of the same individual from a particular locality.

**Figure 12.**
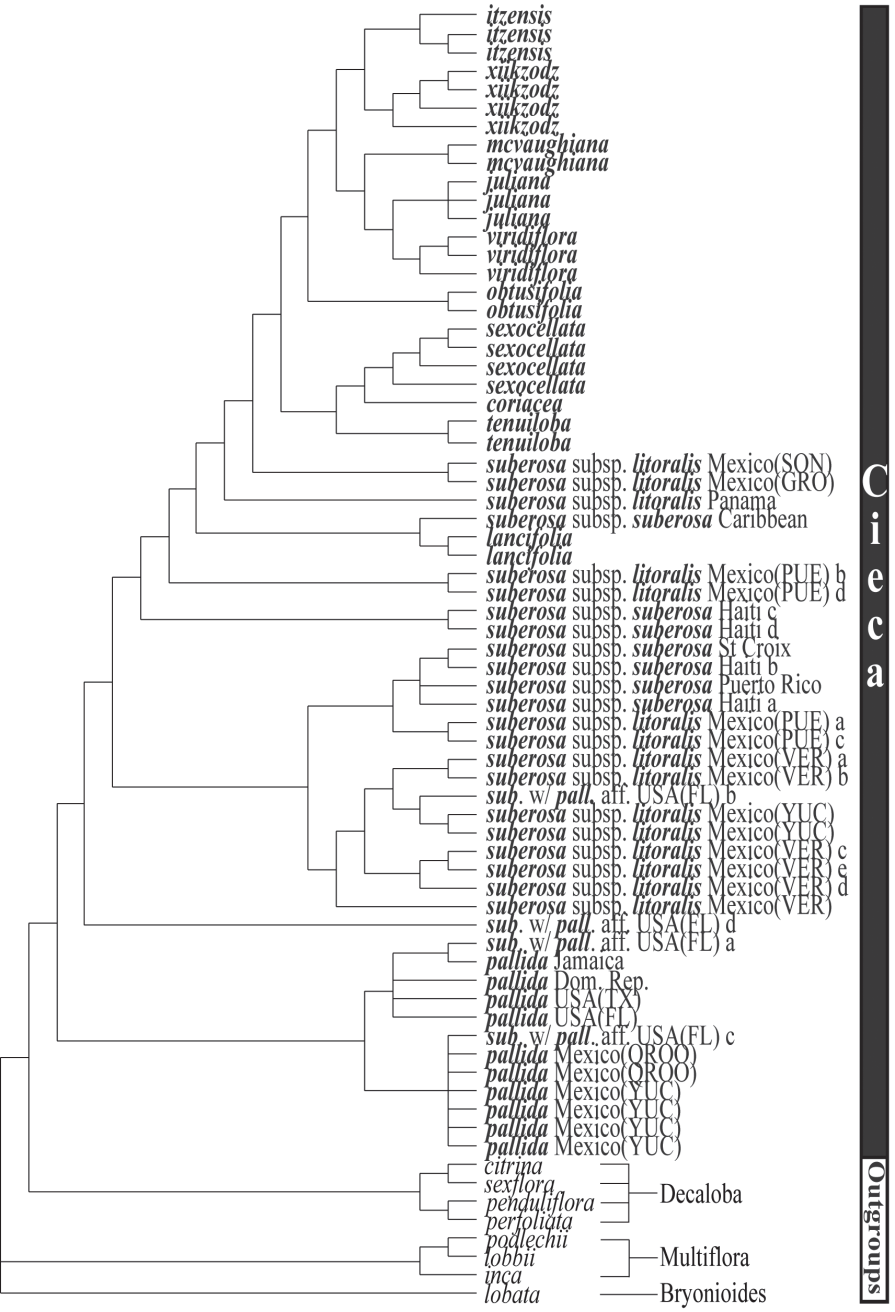
Strict consensus of three most parsimonious trees from the ITS-1 and ITS-2 data set of Passiflora supersection Cieca and outgroups. Tree length = 590; CI = 0.636; RI = 0.837; RC = 0.532. Names followed by the letters “a”, “b”, “c”, “d”, and “e” denote clones of the same individual from a particular locality.

The cladistic analysis (PAUP* 4.0b10) of the molecular ITS-1 and ITS-2 data resulted in the generation of three equally parsimonious trees (Figs 9–12) of 590 steps, a consistency index (CI) of 0.636, and a retention index (RI) of 0.837. The topologies presented in these trees are all quite similar, with only minor rearrangements occurring within the *Passiflora pallida* and *Passiflora suberosa* clades. The strict consensus tree is presented in Fig. 12.

Supersections *Decaloba* and *Multiflora* are monophyletic, with bootstrap values of 92% and 100%, respectively. The two taxa sampled from supersection Decaloba section Xerogona, *Passiflora citrina* J.M.MacDougal and *Passiflora rubra* L., form a monophyletic group (100% bootstrap). *Passiflora penduliflora* Bertero ex DC. and *Passiflora perfoliata* L. are also monophyletic in this analysis (100% bootstrap). The species sampled from supersection *Multiflora* (*Passiflora inca* P.Jørg., Passiflora lobbii Mast. subsp. ayacuchensis Skrabal & Weigend, and *Passiflora podlechii* Skrabal & Weigend) are monophyletic; the type species for the supersection, *Passiflora multiflora* L. was not sampled. *Passiflora lobata* (Killip) Hutch. ex J.M.MacDougal (supersection *Bryonioides*) forms a polytomy at the base of the tree with supersection *Multiflora*.

The monophyly of supersection *Cieca* is strongly supported (100% bootstrap). There is also evidence for the monophyly of *Passiflora pallida* (95% bootstrap), *Passiflora lancifolia* (87% bootstrap), *Passiflora tenuiloba* (100% bootstrap), *Passiflora sexocellata* (93% bootstrap), *Passiflora viridiflora* (96% bootstrap), *Passiflora juliana* (100% bootstrap), *Passiflora obtusifolia* (50% bootstrap), and *Passiflora mcvaughiana* (84% bootstrap). The *Passiflora coriacea/Passiflora sexocellata* and *Passiflora xiikzodz* J. M. MacDougal/*Passiflora itzensis* (J. M. MacDougal) K. Porter-Utley clades are monophyletic and well supported with bootstrap values of 100% and 90%, respectively. *Passiflora juliana* and *Passiflora viridiflora* form a clade (77% bootstrap). A clade consisting of several populations of Passiflora suberosa subsp. suberosa from the Greater Antilles and St. Croix is supported with a bootstrap value of 83%, though this subspecies is, as assessed in this analysis, paraphyletic. A moderately supported clade (73% bootstrap) indicates that an entity of Passiflora suberosa subsp. suberosa from the Caribbean is more closely related to the red, hummingbird-pollinated, Jamaican endemic *Passiflora lancifolia* than it is to other morphologically similar entities of Passiflora suberosa subsp. suberosa (e.g., Passiflora suberosa subsp. suberosa, St. Croix).

The strict consensus tree shows that *Passiflora itzensis*, *Passiflora xiikzodz*, *Passiflora mcvaughiana*, *Passiflora juliana*, *Passiflora viridiflora*, and *Passiflora obtusifolia* form a clade, with *Passiflora obtusifolia* sister to the other above-listed species. Within this group, *Passiflora juliana*, *Passiflora viridiflora*, and *Passiflora mcvaughiana*, three species from southwestern Mexico, constitute a clade. *Passiflora coriacea*, *Passiflora sexocellata*, and *Passiflora tenuiloba* are also grouped together in all trees. *Passiflora suberosa* is non-monophyletic, but a large number of the accessions of this species do constitute a clade in the strict consensus tree. In addition, Passiflora suberosa subsp. suberosa and Passiflora suberosa subsp. litoralis are also both non-monophyletic. In addition, the cladograms indicate that *Passiflora pallida* may be sister to the remaining species of supersection *Cieca*.

**Figure 13.**
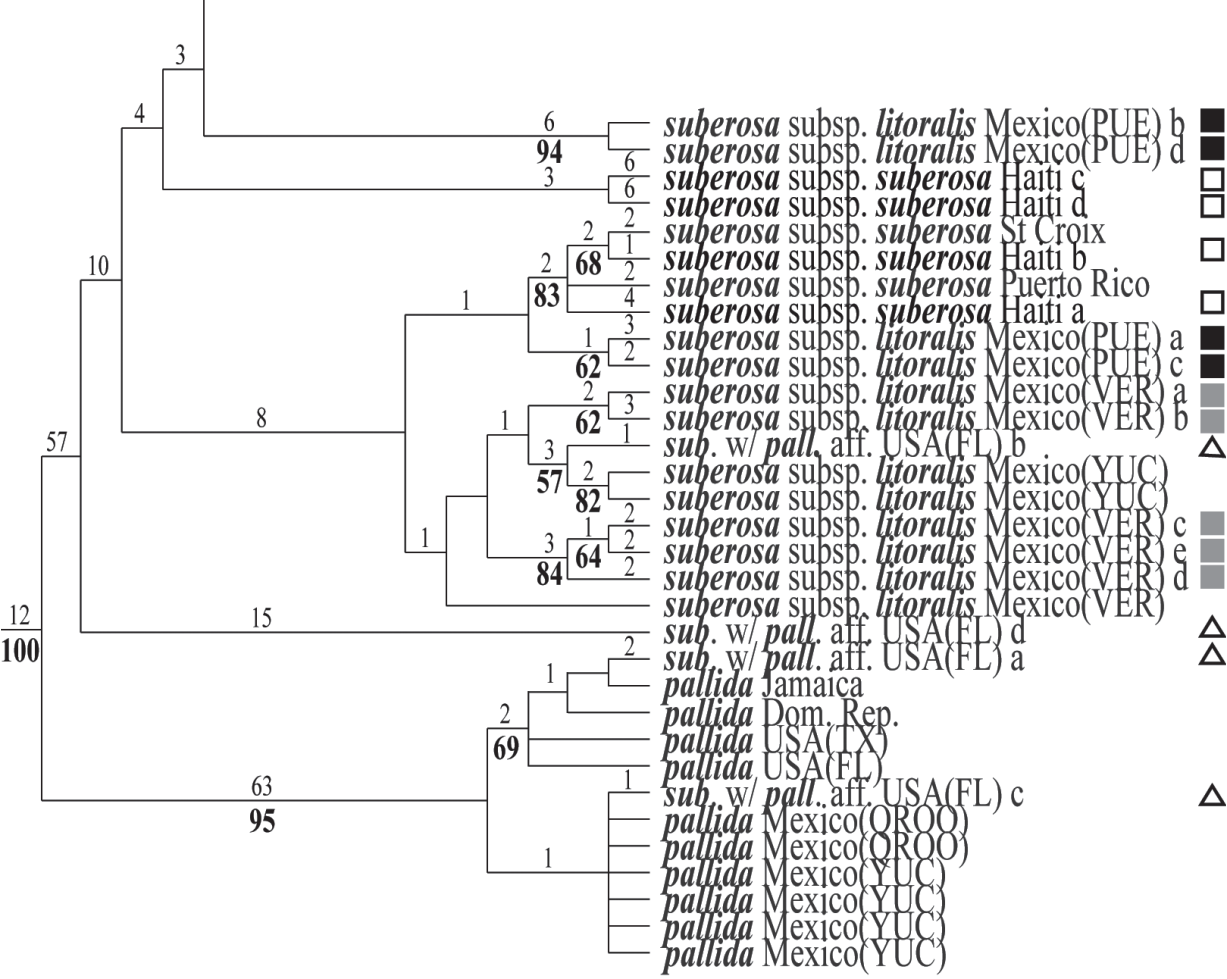
A portion of the strict consensus tree from the ITS-1 and ITS-2 data set of Passiflora supersection Cieca and outgroups highlighting cloned entities. Numbers above branches are branch lengths. Bootstrap values are given below corresponding branches. Tree length = 590; CI = 0.636; RI = 0.837; RC = 0.532. Names followed by letters (“a”, “b”, “c”, “d”, and “e”) and symbols (black square, gray square, white square, and white triangle) denote clones of the same individual from a particular locality.

Of particular interest are the clones of the various entities of *Passiflora suberosa* and *Passiflora pallida* (Fig. 13). Sequences cloned from a single individual of Passiflora suberosa subsp. suberosa from Haiti are found in two different clades, with two clones (“a” and “b”) falling within a moderately supported clade containing other members of the subspecies from the Caribbean and the other two (“c” and “d”) forming a group in the strict consensus tree that is positioned sister to most of the taxa in the supersection. In addition, cloned entities of Passiflora suberosa subsp. litoralis from the states of Puebla and Veracruz, Mexico are found in separate clades. The clones of the “*sub.* w/ *pall.* aff. USA(FL)” entity from Florida occur in both the well-supported *Passiflora pallida* clade (“a” and “c”) and the clade containing *Passiflora suberosa* along with the rest of the species from the supersection (“b” and “d”), indicating that there is gene flow, likely resulting from hybridization, between *Passiflora suberosa* and *Passiflora pallida*.

### Phenetic analyses of species complexes

**Figure 14.**
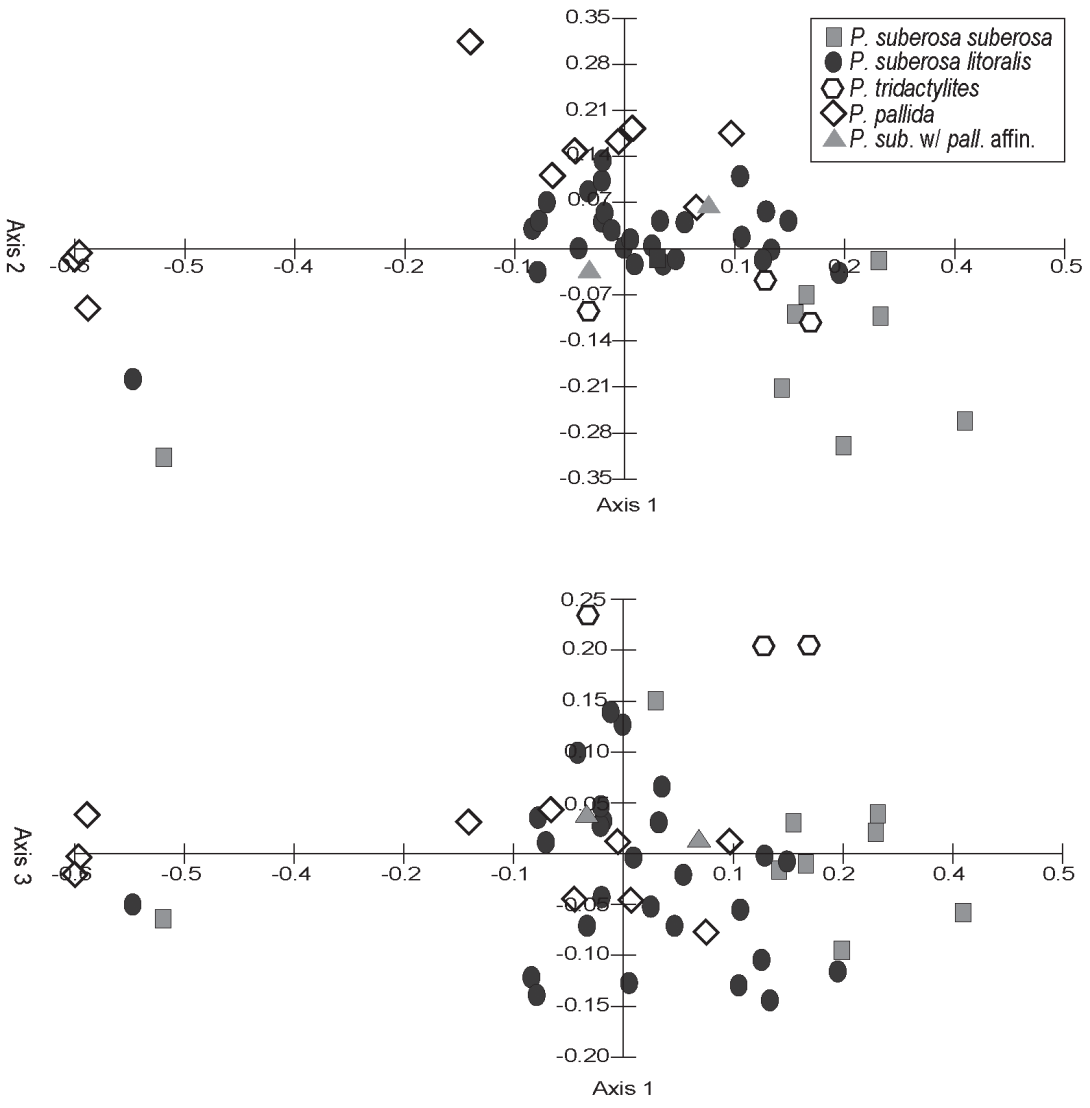
Principal components analysis of the data set for the *Passiflora suberosa* complex based on 51 morphological characters (Table 2).

*The* Passiflora suberosa *Complex.* A principal components analysis (PCA) of the 51 character morphological data set (Table 2) for the *Passiflora suberosa* complex is presented in Fig. 14. Taxa recognized in this revision as Passiflora suberosa subsp. suberosa, Passiflora suberosa subsp. litoralis, *Passiflora tridactylites*, and *Passiflora pallida* were included in the analyses and are labeled accordingly. In addition, entities that may be of hybrid derivation and possess both *Passiflora pallida* and *Passiflora suberosa* affinities are indicated. Principal components I, II, and III account for 48.3%, 14.2%, and 7.7% of the variation, respectively, for a total of 70.2%. Principal component axis I is most highly influenced by (presented in decreasing order of component loadings) (Table 3): (1) length of the lateral leaf lobe, (2) distance from the outline of the leaf to the margin of the leaf sinus, and (3) leaf width; axis II by (1) androgynophore length, (2) sepal length, and (3) stipe length; axis III by (1) petiole length, (2) distance of the petiolar nectaries from the petiole base, and (3) number of laminar nectaries. The PCA plots of axes I and II and axes I and III separate *Passiflora tridactylites* from the other taxa in the analysis, but the remaining taxa are poorly separated. The first principal component (PC1) consists primarily of information from vegetative characters and is largely an indication of leaf size and leaf lobe depth. The second principal component (PC2) has low component loadings for the vegetative characters and high component loadings for the floral characters and is primarily an indicator of flower size. Because PC1 and PC2 are by definition not correlated, this division of floral and vegetative characters between these first two PCs indicates that there is little correlation between floral and vegetative characters among the entities in the *Passiflora suberosa* complex.

**Table 2.** Characters used in the morphology-based principal components analysis of the *Passiflora suberosa* complex (* = characters used in the PCA analysis of the data set for *Passiflora suberosa* complex based on floral characters) All measurements were recorded in mm. For a discussion of character state delimitation see Methods.

N	Measurements
1.	*Pedicel length
2.	*Pedicel width
3.	*Stipe length
4.	*Stipe width
5.	*Hypanthium diameter
6.	*Sepal length
7.	*Sepal width
8.	*Number of coronal rows
9.	*Number of filaments in outer coronal row
10.	*Length of filaments in outer coronal row
11.	*Width of filaments in outer coronal row
12.	*Number of filaments in inner coronal row
13.	*Length of filaments in inner coronal row
14.	*Width of filaments in inner coronal row
15.	*Staminal filament length
16.	*Staminal filament width
17.	*Anther length
18.	*Anther width
19.	*Style length
20.	*Style width
21.	*Stigma width
22.	*Nectary width
23.	*Androgynophore length
24.	*Androgynophore width
25.	*Ovary length
26.	*Ovary width
27.	*Operculum length
28.	*Nectary height
29.	*Limen height
30.	*Limen diameter
31.	*Limen floor diameter
32.	Distance of petiolar nectary from petiole base
33.	Petiole length
34.	Petiolar nectary position
35.	Length of pubescence on petiole
36.	Petiolar nectary diameter
37.	Petiolar nectary height
38.	Degree leaf peltate
39.	Lateral leaf vein length
40.	Central leaf vein length
41.	Quotient: lateral to central lobe length
42.	Distance from leaf outline to sinus margin
43.	Depth of leaf lobe
44.	Leaf width
45.	Length of pubescence on leaf
46.	Number of laminar nectaries
47.	Stem diameter
48.	Length of pubescence on stem
49.	Stipule length
50.	Stipule width
51.	Tendril width

**Table 3.** Component loadings for axes I, II, and III from a principal components analysis of the *Passiflora suberosa* complex (Fig. 14) The values were computed from quantitative vegetative and floral variables.

Variables	PCI	PCII	PCIII
Pedicel length	0.072	-0.186	0.174
Pedicel width	0.002	-0.015	0.013
Stipe length	0.065	-0.294	-0.101
Stipe width	0.006	-0.047	0.005
Hypanthium diameter	0.060	-0.187	0.081
Sepal length	0.054	-0.307	0.127
Sepal width	0.046	-0.150	0.009
Number of coronal rows	0.003	-0.013	0.006
Number of filaments in outer coronal row	0.019	-0.005	0.040
Length of filaments in outer coronal row	0.081	-0.230	0.202
Width of filaments in outer coronal row	0.005	-0.024	0.029
Number of filaments in inner coronal row	0.039	0.058	0.177
Length of filaments in inner coronal row	0.071	-0.222	0.204
Width of filaments in inner coronal row	0.003	-0.019	0.005
Staminal filament length	0.053	-0.230	0.052
Staminal filament width	0.005	-0.034	-0.018
Anther length	0.022	-0.125	-0.029
Anther width	-0.001	-0.027	-0.037
Style length	0.058	-0.190	0.012
Style width	0.010	-0.054	-0.018
Stigma width	0.008	-0.022	0.030
Nectary width	0.069	-0.179	-0.048
Androgynophore length	0.093	-0.338	0.248
Androgynophore width	0.015	-0.067	-0.016
Ovary length	0.045	-0.169	0.148
Ovary width	0.036	-0.112	0.007
Operculum length	0.049	-0.166	0.093
Nectary height	0.022	-0.04	0.065
Limen height	0.022	-0.025	0.037
Limen diameter	0.005	-0.002	0.032
Limen floor diameter	0.034	-0.071	0.058
Distance of petiolar nectary from pet. base	0.040	-0.115	-0.336
Petiole length	0.081	-0.146	-0.357
Position of petiolar nectaries	-0.018	0.012	0.000
Length of pubescence on petiole	0.011	0.000	-0.058
Petiolar nectary diameter	0.032	-0.067	-0.112
Petiolar nectary height	0.000	-0.008	-0.058
Degree leaf peltate	0.032	-0.029	-0.114
Length of lateral leaf lobe	0.695	0.270	-0.127
Length of central leaf lobe	0.028	-0.212	-0.284
Quotient: lateral/central lobe length	0.095	0.041	0.018
Distance from leaf outline to sinus margin	0.608	0.148	0.214
Depth of leaf lobe	0.078	0.038	0.123
Leaf width	0.220	-0.177	-0.207
Length of pubescence on leaf	0.027	0.024	-0.083
Number of laminar nectaries	0.099	-0.243	-0.318
Stem diameter	0.030	-0.040	-0.093
Length of pubescence on stem	0.001	0.050	-0.079
Stipule length	0.016	0.020	-0.313
Stipule width	0.010	0.023	-0.094
Tendril width	0.017	-0.053	-0.075

**Figure 15.**
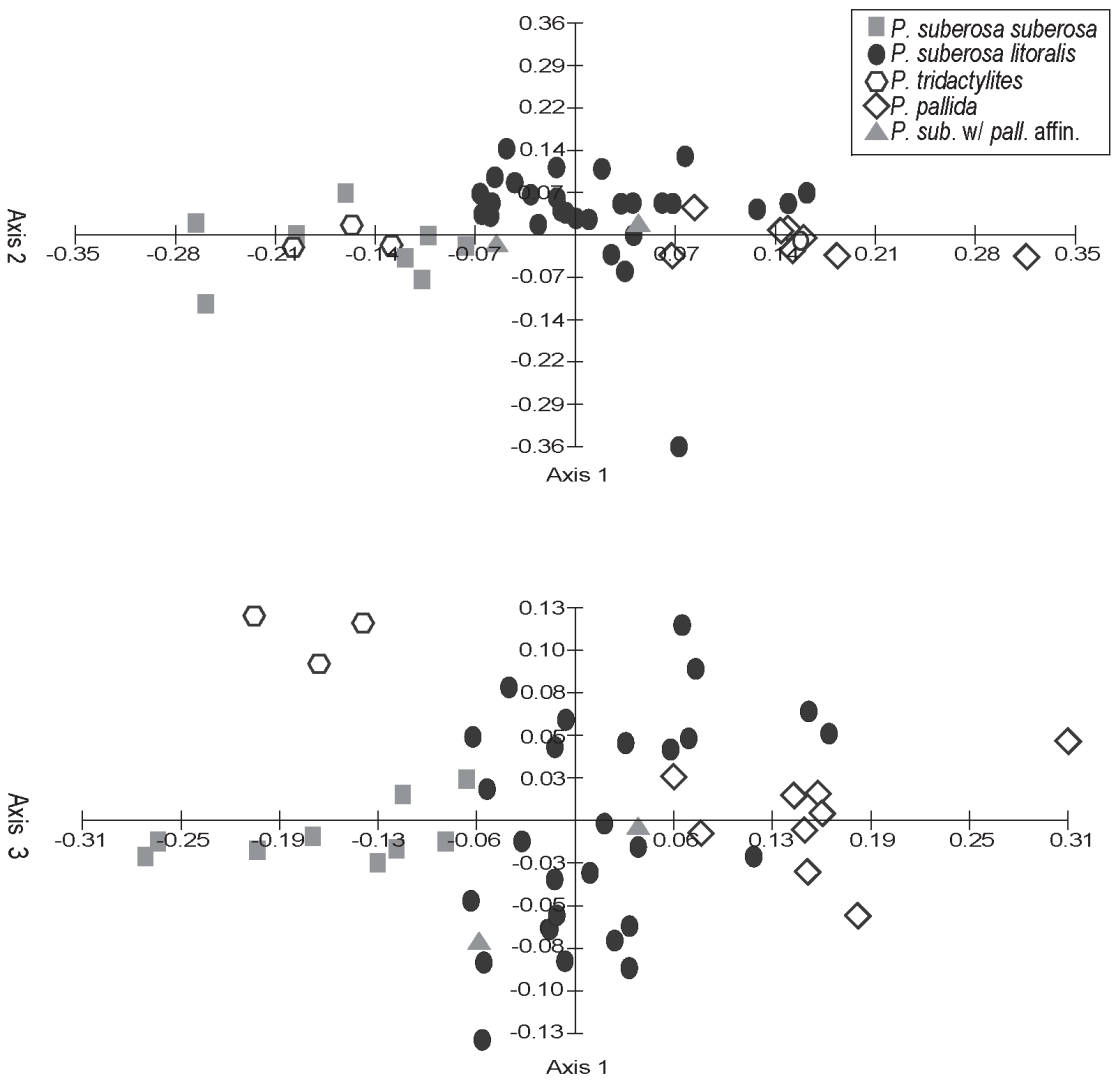
Principal components analysis of the data set for the *Passiflora suberosa* complex based on 31 floral characters (Table 2).

A PCA analysis of the data set for the *Passiflora suberosa* complex based on 31 floral characters (Table 2) is presented in Fig. 15. Principal components I, II, and III account for 47.1%, 15.0%, and 9.5% of the variation, respectively, for a total of 71.6%. Principal component axis I is most highly influenced by (presented in decreasing order of component loadings) (Table 4): (1) androgynophore length, (2) sepal length, and (3) length of the filaments in the outer coronal row; axis II by (1) number of filaments in the inner coronal row, (2) pedicel length, and (3) number of filaments in the outer coronal row; axis III by (1) pedicel length, (2) stipe length, and (3) nectary width. The PCA plots of axes I and II and axes I and III clearly separate *Passiflora tridactylites*, Passiflora suberosa subsp. suberosa, Passiflora suberosa subsp. litoralis, and *Passiflora pallida*. The first and third principal components are a measure of overall flower size and PC2 is mostly a measure of the relationship among the number of filaments present in each coronal row. When PC1 and PC3 of floral characters are plotted for individual plants (Fig. 15), individuals from the same species and subspecies tend to cluster together although there is limited overlap among entities of Passiflora suberosa subsp. litoralis and *Passiflora pallida*, possibly resulting from hybridization. The pattern reflected in the scatter plot of the first and third components in Fig. 15 strongly relates to the overall size of the flower, with entites in the left half of the scatter plot having larger flowers than those individuals in the right half.

**Table 4.** Component loadings for axes I, II, and III from a principal components analysis of the *Passiflora suberosa* complex (Fig. 15) The values were computed from quantitative floral variables.

Variables	PCI	PCII	PCIII
Pedicel length	-0.242	0.189	0.652
Pedicel width	-0.020	0.011	-0.035
Stipe length	-0.282	-0.032	-0.592
Stipe width	-0.048	0.016	-0.057
Hypanthium diameter	-0.231	0.021	-0.028
Sepal length	-0.335	-0.058	0.036
Sepal width	-0.174	0.015	-0.106
Number of coronal rows	-0.014	0.046	-0.016
Number of filaments in outer coronal row	-0.027	0.150	-0.001
Length of filaments in outer coronal row	-0.317	0.028	0.044
Width of filaments in outer coronal row	-0.032	0.003	0.022
Number of filaments in inner coronal row	-0.020	0.955	-0.153
Length of filaments in inner coronal row	-0.295	0.021	0.098
Width of filaments in inner coronal row	-0.021	-0.003	-0.015
Staminal filament length	-0.255	-0.050	-0.052
Staminal filament width	-0.033	-0.002	-0.058
Anther length	-0.125	-0.023	-0.172
Anther width	-0.018	-0.009	-0.142
Style length	-0.218	-0.029	-0.075
Style width	-0.050	-0.038	-0.018
Stigma width	-0.035	0.030	-0.053
Nectary width	-0.196	-0.062	-0.214
Androgynophore length	-0.417	-0.039	0.204
Androgynophore width	-0.072	0.017	-0.065
Ovary length	-0.213	-0.061	0.096
Ovary width	-0.131	-0.004	-0.077
Operculum length	-0.199	-0.041	-0.009
Nectary height	-0.066	-0.005	-0.012
Limen height	-0.048	0.015	-0.010
Limen diameter	-0.015	0.022	0.004
Limen floor diameter	-0.110	0.059	-0.016

**Figure 16.**
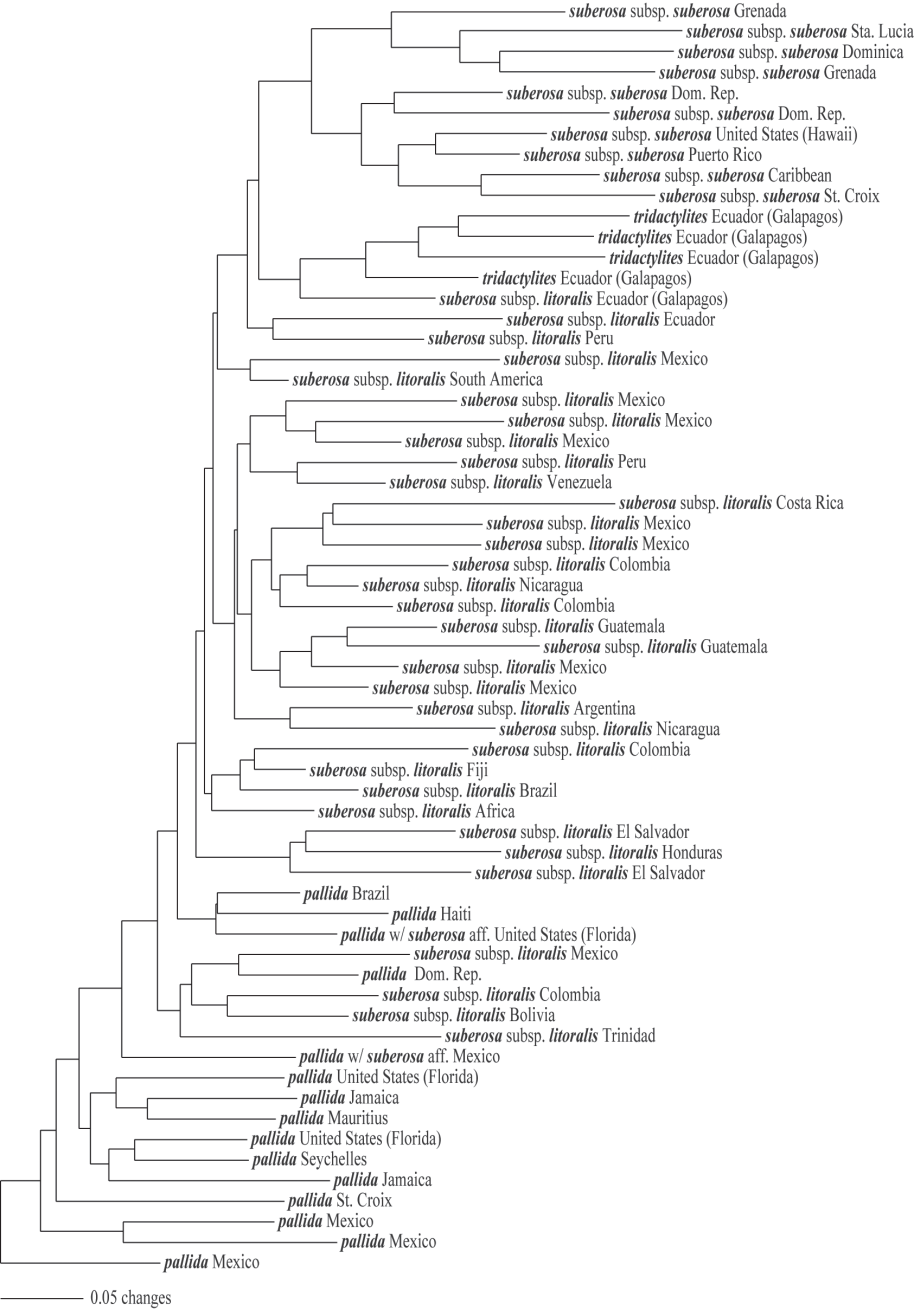
Unrooted neighbor joining tree resulting from the analysis of the entire mophological data set for the *Passiflora suberosa* complex based upon 77 morphological characters (Tables 5–6).

The unrooted neighbor joining tree produced from an analysis of the entire morphological data set of the *Passiflora suberosa* complex (Table 5–6) is shown in Fig. 16. Passiflora suberosa subsp. suberosa and *Passiflora tridactylites*, the two most morphologically distinct taxa in the *Passiflora suberosa* complex, are clearly resolved at the “top” of the tree. One accession of Passiflora suberosa subsp. litoralis from the Galapagos is more similar to *Passiflora tridactylites* than to other members of the subspecies from Central and South America, but *Passiflora tridactylites* can be easily separated from this accession by its elongated androgynophore (the average androgynophore length of *Passiflora tridactylites* is 7.5 mm and the average androgynophore length of this accession of Passiflora suberosa subsp. litoralis from the Galapagos is 5.9 mm; char. #19) and longer outer coronal filaments (the average length of the filaments in the outer coronal row of *Passiflora tridactylites* is 7.5 mm and the average length of the filaments in the outer coronal row of this accession of Passiflora suberosa subsp. litoralis from the Galapagos is 5.2 mm; char. #8). Passiflora suberosa subsp. suberosa forms two distinct clusters, a Lesser Antillean group and a largely Greater Antillean group, with one accession from Hawaii (Oahu) and one from St. Croix found within the Greater Antillean cluster.

**Table 5.** Characters used in the morphology-based neighbor joining analysis of the *Passiflora suberosa* complex. For a discussion of character state delimitation see Methods.

1.	Pedicel length (mm) ≤12.50 (0); >12.50 (2)
2.	Stipe length (mm) 2.10–6.70 (0); 6.71–9.00 (1); > 9.00 (2); <2.10 (3)
3.	Stipe length/pedicel length (quotient) <0.42 (0); 0.42–0.98 (1); >0.98 (2)
4.	Hypanthium diameter (mm) 3.37–4.94 (0); 4.95–6.54 (1); 6.55–8.25 (2); >8.25 (3); <3.37 (4)
5.	Sepal length (mm) <5.80 (0); 5.81–8.68 (1); 8.69–12.87 (2); 12.88–14.62 (3); 14.63–18.74 (4); >18.74 (5)
6.	Sepal width (mm) <2.74 (0); 2.74–4.13 (1); >4.13 (2)
7.	Number of filaments in the outer coronal row (number) >25 (0); 18–26 (1); <18 (2)
8.	Length of filaments in the outer coronal row (mm) <2.75 (0); 2.75–4.00 (1); 4.01–5.88 (2); >5.88 (3)
9.	Length of filaments in the inner coronal row/sepal length (quotient) 0.42–0.72 (0); >0.72 (1); <0.23 (2); 0.23–0.41 (3)
10.	Number of filaments in the inner coronal row (number) 18–31 (0); 32–37 (1); 38–45 (2); >45 (3); <16 (4); 17 (5)
11.	Length of filaments in the inner coronal row (mm) >3.26 (0); 1.95–3.26 (1); 1.33–1.94 (2); <1.32 (3)
12.	Length of filaments in the inner coronal row/length of filaments in the outer coronal row (quotient) >3.1 (0); <0.30 (1)
13.	Staminal filament length (mm) <2.51 (0); 2.51–3.53 (1); 3.54–4.34 (2); >4.60 (3)
14.	Anther length (mm) 1.48–2.00 (0); 2.01–2.47 (1); 2.48–2.67 (2); >2.67 (3) <1.48 (4)
15.	Style length (mm) <4.08 (0); 4.08–5.34 (1); 5.54–6.34 (2); >6.87 (3)
16.	Stigma width (mm) 0.61–1.12 (0); >1.12 (1); <0.61 (2)
17.	Ovary length (mm) <2.35 (0); ≥2.35 (1)
18.	Nectary width (mm) 0.41–1.33 (0); 1.34–2.67 (1); >2.67 (2); <0.41 (3)
19.	Androgynophore length (mm) <4.41 (0); 5.00–6.07 (1); 8.00–12.63 (2); >13.12 (3)
20.	Androgynophore width (mm) 0.57–1.40 (0); >1.52 (1); <0.57 (2)
21.	Staminal filament length/androgynophore length (quotient) >0.45 (0); 0.34–0.43 (1); 0.31 (2)
22.	Operculum length (mm) ≤2.00 (0); >2.00 (1)
23.	Nectary height (mm) ≤0.53 (0); >0.53 (1)
24.	Limen height (mm) ≤0.35 (0); >0.35 (1)
25.	Limen floor diameter (mm) >2.19 (0); ≤2.19 (1)
26.	Fruit length (mm) ≤9.63 (0); 11.10–11.50 (1); ≥11.88 (2)
27.	Fruit width (mm) ≤10.00 (0); ≥10.50 (1)
28.	Seed length (mm) ≤3.54 (0); ≥3.67 (1)
29.	Seed width (mm) <1.80 (0); 1.80–2.13 (1); >2.13 (2)
30.	Length of central leaf vein (mm) <136 (0); ≥136 (1)
31.	Width of central leaf lobe (mm) ≤10.00 (0); 10.01–26.00 (1); >26.00 (2)
32.	Length of central leaf vein/width of central leaf lobe (quotient) ≤4.00 (0); 4.01–9.50 (1); >9.50 (2)
33.	Width of lateral leaf lobe (mm) <11.00 (0); 11.00–20.00 (1); >20.00 (2)
34.	Angle between lateral leaf veins (degrees) >94 (0); 58–94 (1); <58 (2)
35.	Distance from the leaf outline to the sinus margin (mm) <8.26 (0); 8.26–20.99 (1); >20.99 (2)
36.	Distance from the leaf outline to the leaf base (mm) >24.00 (0); ≤24.00 (1)
37.	Leaf lobe depth (quotient: distance from leaf outline to margin of sinus/distance from leaf outline to leaf base) 0.22–0.35 (0); 0.36–0.63 (1); >0.63 (2); <0.22 (3)
38.	Length of central leaf vein/length of lateral leaf vein (quotient) >0.44 (0); ≤0.44 (1)
39.	Length of lateral leaf vein/width of lateral leaf lobe (quotient) <5.00 (0); 5.00–10.40 (1); >10.40 (2)
40.	Leaf width (mm) 5.01–41.99 (0); >49.99 (1); <5.01 (2)
41.	Distance from petiolar nectary to petiole base (mm) <14.13 (0); ≥14.13 (1)
42.	Position of petiolar nectary (quotient: distance from petiolar nectary to petiole base/petiole length) ≤0.49 (0); 0.50–0.65 (1); 0.66–0.75 (2); >0.75 (3)
43.	Diameter of petiolar nectary (mm) <0.75 (0); 0.75–1.20 (1); >1.20 (2)
44.	Stipule length (mm) >7.40 (0); 2.37–7.40 (1); ≤2.36 (2)
45.	Stipule width (mm) >0.80 (2); 0.48–0.80 (1); 0.20–0.47 (0); <0.20 (3)
46.	Inflorescences not present (0); inflorescences present as condensed shoots with aborted laminas (1)
47.	Bracts absent (0); bracts present (1)
48.	Spur absent (0); spur present (1)
49.	Sepals greenish yellow (0); sepals whitish (1)
50.	Sepals pubescent (0); sepals ± glabrous (1)
51.	Two coronal rows present (0); one coronal row present (1)
52.	Outer coronal filaments without red/purple pigmentation (0); outer coronal filaments with a flush of red/purple pigmentation at base (1); outer coronal filaments with evident red/purple pigmentation (2); outer coronal filaments with conspicuous red/purple pigmentation (3)
53.	Outer coronal filaments linear (0); outer coronal filaments dilated toward apex (1)
54.	Outer coronal filaments erect (0); outer coronal filaments spreading flat (1)
55.	Outer coronal filaments not fused (0); outer coronal filaments fused (1)
56.	Inner coronal filaments without red/purple pigmentation (0); inner coronal filaments with a flush of red/purple pigmentation at base (1); inner coronal filaments with evident red/purple pigmentation (2); inner coronal filaments with conspicuous red/purple pigmentation (3)
57.	Androgynophore without red/purple pigmentation (0); androgynophore with a flush of red/purple pigmentation at base (1); androgynophore with conspicuous red/purple pigmentation (2)
58.	Ovary ellipsoid (0); ovary globose (1); ovary fusiform (2); ovary obovoid (3)
59.	Ovary ± glabrous (0); ovary pubescent (1)
60.	Operculum present (0); operculum absent (1)
61.	Operculum without red/purple pigmentation (0); operculum with a flush of red/purple pigmentation at base (1); operculum with evident red/purple pigmentation (2); operculum with conspicuous red/purple pigmentation (3)
62.	Nectary without raised annulus (0); nectary with raised annulus (1)
63.	Limen recurved (0); limen erect or inclined toward the operculum (1)
64.	Limen without red/purple pigmentation (0); limen with a flush of red/purple pigmentation at base (1); limen with conspicuous red/purple pigmentation (2)
65.	Limen floor without red/purple pigmentation (0); limen floor with a flush of red/purple pigmentation at base (1); limen floor with evident red/purple pigmentation (2); limen floor with conspicuous red/purple pigmentation (3)
66.	Fruit globose (0); fruit ellipsoid (1); fruit fusiform (2); fruit ovoid (3)
67.	Petiolar nectaries opposite (0); petiolar nectaries subopposite (1); petiolar nectaries alternate (2)
68.	Petiolar nectaries capitate (0); petiolar nectaries obconical (1); petiolar nectaries cupulate (2); petiolar nectaries discoid (3); petiolar nectaries urceolate (4)
69.	Leaves not peltate (0); leaves peltate (1)
70.	Leaves trilobed (0); leaves unlobed (1); leaves bilobed to trilobed (2); leaves unlobed to trilobed (3)
71.	Leaf base cordate (0); leaf base not cordate (1)
72.	Leaf margin entire (0); leaf margin crenate (1)
73.	Leaves not variegated (0); leaves variegated (1)
74.	Leaves with primary veins diverging and branching at base (0); leaves with primary veins diverging and branching above base (1); leaves with secondary veins forming a series of loops (2)
75.	Laminar nectaries absent (0); laminar nectaries present (1)
76.	Stem entirely greenish yellow (0); stem with reddish purple coloration (1)
77.	Stipules glabrous (0); stipules pubescent (1)

**Table 6.** Character values used in the construction of the neighbor joining tree for taxa in the *Passiflora suberosa* complex (Fig. 16) A = 0/1; B = 0/2; C = 0/3; D = 0/5; E = 1/2; F = 1/2/3; G = 2/3; ? = condition unknown.

Taxon locality (Collection number)	1	2	3	4	5	6	7	8	9	0	1	2	3	4	5	6	7	8	9	0	1	2	3	4	5	6	7	8	9	0	1	2	3	4	5	6	7	8	9	0
suberosa subsp. suberosa Grenada (10859)	1	1	1	2	G	1	1	2	3	2	1	0	3	3	A	0	0	A	2	0	0	1	0	1	1	2	1	1	2	0	0	0	1	1	2	0	2	0	0	1
*tridactylites* Galapagos (1095)	A	C	0	2	2	1	0	3	0	0	2	0	1	A	A	0	1	0	2	0	1	1	0	0	0	?	?	?	?	0	2	2	0	0	2	0	2	0	2	1
suberosa subsp. suberosa Dom. Rep. (121)	1	0	A	1	1	1	0	2	0	0	0	0	2	1	0	0	1	0	1	0	0	0	0	A	0	?	?	?	?	0	0	1	1	1	2	0	2	0	A	1
suberosa subsp. litoralis Brazil (12264)	1	0	0	0	1	1	0	1	0	0	0	0	1	0	0	0	0	0	0	0	0	0	0	0	0	?	?	?	?	0	A	0	1	1	1	0	1	0	0	1
suberosa subsp. litoralis Mexico (1272)	0	0	E	1	1	1	1	3	1	1	1	0	1	1	0	0	0	0	0	B	0	1	0	0	1	?	?	?	?	0	2	A	0	0	1	1	E	0	A	0
suberosa subsp. litoralis El Salvador (1361)	0	1	1	0	1	1	1	1	3	0	0	0	1	3	0	0	0	0	0	0	0	0	0	0	1	?	?	?	?	0	0	0	1	0	2	0	1	0	0	1
suberosa subsp. litoralis Galapagos (1420)	0	0	0	1	1	1	1	2	0	0	1	0	1	0	0	0	0	0	1	0	0	1	0	0	0	1	0	0	0	0	0	0	1	0	A	0	1	0	0	1
*pallida* w/*suberosa* aff. USA-Florida (14284)	0	0	1	0	1	1	0	1	0	0	0	0	1	0	0	0	0	0	0	0	0	0	0	0	0	?	?	?	?	0	0	0	1	1	1	0	1	0	0	1
suberosa subsp. litoralis Costa Rica (1486)	1	2	1	1	1	1	1	2	0	2	1	0	1	0	0	0	?	0	0	0	0	0	0	1	0	?	?	?	?	0	1	0	1	1	0	0	3	0	0	1
suberosa subsp. litoralis Colombia (15029)	0	1	2	1	1	1	0	2	A	0	0	0	1	0	0	0	0	2	0	0	0	0	0	0	0	2	1	0	2	0	1	0	2	0	2	0	1	0	0	1
suberosa subsp. litoralis Ecuador (15217)	1	0	0	0	1	1	1	3	1	0	0	1	2	2	0	0	1	0	0	0	0	0	0	0	0	?	?	?	?	0	0	0	1	0	1	0	1	0	0	1
suberosa subsp. litoralis Colombia (16834)	1	0	0	1	1	1	0	2	0	1	0	0	1	1	0	0	1	0	0	0	0	0	0	0	0	?	?	?	?	0	1	0	A	1	0	0	3	0	0	A
suberosa subsp. litoralis Nicaragua (18533)	0	0	E	A	1	1	0	E	0	0	0	0	1	3	0	0	0	0	0	0	0	0	0	0	A	?	?	?	?	0	A	0	E	1	0	0	3	0	0	A
suberosa subsp. suberosa Hawaii-Oahu (188)	0	0	2	1	1	1	0	2	0	0	1	0	E	2	0	0	1	0	1	0	0	0	1	1	0	?	?	?	?	0	1	0	1	2	0	0	3	0	0	0
*pallida* USA-Florida (194)	0	0	1	0	0	0	0	0	0	0	4	0	0	5	0	0	0	0	0	0	0	0	0	0	1	?	?	?	?	0	2	1	0	0	2	0	2	0	1	1
suberosa subsp. litoralis Africa (2190)	0	0	A	0	1	1	1	1	0	0	1	0	1	1	0	0	A	0	0	0	0	0	0	0	1	0	0	0	2	0	0	0	1	0	1	0	1	0	0	1
suberosa subsp. litoralis Honduras (23340)	0	1	1	0	1	1	1	2	0	1	0	0	2	3	0	B	0	0	0	2	0	0	0	0	1	?	?	?	?	0	?	?	?	?	?	?	?	?	?	0
suberosa subsp. litoralis Colombia (2436)	0	0	1	1	1	1	1	2	0	2	0	0	1	1	0	0	0	0	0	0	0	0	0	0	0	?	?	?	?	0	0	0	1	1	0	0	C	0	0	0
suberosa subsp. suberosa St. Lucia (251)	0	2	1	2	5	1	1	3	3	4	2	0	3	3	3	0	1	1	2	0	0	1	0	0	0	1	0	1	2	0	1	0	2	1	1	0	0	0	0	1
suberosa subsp. litoralis Guatemala (25309)	0	0	0	0	1	1	0	2	0	4	0	0	1	1	0	0	0	0	0	0	0	0	0	0	A	?	?	?	?	0	0	0	1	1	0	1	C	0	0	0
*tridactylites* Galapagos (2620)	?	0	A	0	2	0	1	3	0	0	1	0	2	1	0	0	1	0	2	0	1	0	0	0	0	2	0	0	0	0	0	0	1	?	?	?	?	0	0	?
suberosa subsp. suberosa Caribbean (281)	0	0	1	E	2	E	0	2	0	0	1	0	2	F	1	1	1	0	1	0	0	0	1	1	0	?	?	?	?	0	1	0	E	E	E	0	0	0	0	1
suberosa subsp. litoralis Mexico (283)	0	0	1	5	0	0	0	1	1	0	0	0	0	0	0	0	0	0	0	2	0	0	0	0	1	?	?	?	?	0	0	0	1	0	0	A	0	0	0	1
suberosa subsp. litoralis Mexico (2966)	0	?	?	0	1	1	2	2	0	0	A	0	0	0	0	2	0	0	0	0	0	0	?	?	1	?	?	?	?	0	0	1	A	0	2	0	2	0	1	1
suberosa subsp. litoralis Colombia (3002)	0	0	0	0	0	0	1	0	0	0	4	0	0	0	0	2	0	0	0	2	0	0	0	0	1	?	?	?	?	0	A	0	E	0	E	0	1	0	0	1
suberosa subsp. litoralis Mexico (303)	0	3	0	1	1	1	0	2	1	3	1	0	1	0	0	0	0	0	0	0	0	0	0	0	0	?	?	?	?	0	1	0	1	1	0	0	C	0	0	1
suberosa subsp. litoralis Argentina (30408)	0	1	2	0	1	1	1	1	0	1	1	0	1	0	0	0	0	0	0	0	0	0	0	0	0	0	0	0	1	0	0	0	1	0	0	0	0	0	0	1
suberosa subsp. suberosa Dominica (3082)	A	1	1	2	2	2	1	3	0	0	1	0	3	1	2	0	1	1	2	0	0	1	0	1	0	?	?	?	?	0	A	0	2	0	1	0	0	0	0	1
suberosa subsp. litoralis Trinidad (31838)	0	0	1	5	A	0	0	1	0	4	?	1	0	0	0	0	A	0	0	0	0	?	0	0	1	?	?	?	?	0	1	0	E	0	1	0	A	0	0	1
*pallida* Jamaica (32)	0	0	1	0	0	0	0	0	C	0	4	0	0	5	0	0	0	0	0	0	0	0	0	0	1	?	?	?	?	0	B	A	0	2	1	0	1	0	1	0
suberosa subsp. litoralis Mexico (343)	0	0	1	A	1	1	1	2	0	2	A	0	A	0	0	0	0	0	0	0	0	0	0	0	0	?	?	?	?	0	0	A	0	1	0	1	0	1	0	0
suberosa subsp. litoralis Mexico (344)	0	A	E	0	2	1	1	3	0	A	1	0	1	3	1	0	0	0	1	0	0	0	0	1	0	?	?	?	?	0	0	0	0	1	0	1	A	0	0	0
*pallida* Mauritius (3558)	0	0	2	0	0	0	0	0	0	0	4	0	0	5	0	0	0	0	0	2	0	0	0	0	1	0	0	0	2	0	A	0	1	1	1	0	1	0	0	0
*pallida* Mexico (393)	0	0	1	0	1	1	0	1	3	0	0	0	0	0	0	0	0	3	0	0	0	0	0	0	1	?	?	?	?	0	?	?	?	?	?	?	?	?	?	0
*tridactylites* Galapagos (403)	1	0	0	1	2	1	1	3	A	0	E	0	3	A	0	0	1	0	2	0	0	1	0	0	0	?	?	?	?	0	2	0	0	0	0	1	1	0	0	0
*pallida* Mexico (403)	0	0	1	5	1	0	0	0	2	0	4	0	1	0	0	2	0	3	0	2	0	0	0	0	1	?	?	?	?	0	?	?	?	?	?	?	?	?	?	0
*pallida* Mexico (412)	0	0	0	5	1	A	0	1	3	4	4	0	0	0	0	0	0	3	0	0	0	0	0	0	1	?	?	?	?	?	?	?	?	?	?	?	?	?	?	?
*pallida* St. Croix (413)	0	0	1	5	0	0	1	1	A	0	4	0	?	?	0	2	0	0	?	?	?	?	0	0	1	?	?	?	?	0	?	?	?	?	?	?	?	?	?	1
*pallida* wl *suberosa* aff. Mexico (413)	0	0	1	0	1	1	0	0	3	A	4	0	A	5	0	0	?	0	0	0	0	0	0	0	1	?	?	?	?	0	0	0	0	1	1	0	1	0	0	A
suberosa subsp. litoralis Nicaragua (4162-b)	0	0	2	1	1	1	1	1	0	1	1	0	1	A	0	0	0	0	0	0	0	0	0	0	0	?	?	?	?	0	0	A	0	2	0	1	3	1	0	0
*pallida* USA-Florida (41876)	0	0	1	0	0	0	0	0	0	0	4	0	0	5	0	0	A	3	0	0	0	0	0	0	1	?	?	?	?	0	A	0	E	1	0	0	3	0	0	0
suberosa subsp. litoralis Peru (4514)	0	3	0	0	1	1	1	2	1	2	0	0	1	0	0	0	0	0	0	0	0	0	0	0	0	?	?	?	?	0	B	0	0	0	0	1	1	0	0	0
suberosa subsp. litoralis Bolivia (4707)	0	3	0	0	0	A	1	A	0	0	0	0	0	0	0	2	A	3	0	0	0	0	0	0	0	?	?	?	?	0	0	0	1	0	0	0	0	0	0	A
*pallida* Seychelles (49451)	0	3	A	0	A	0	1	0	C	0	4	0	A	0	0	0	0	0	0	0	0	0	0	0	1	?	?	?	?	0	?	?	?	?	?	?	?	?	?	0
suberosa subsp. litoralis El Salvador (495)	0	1	1	0	1	2	0	2	0	0	0	0	1	3	0	2	0	0	0	1	0	0	0	0	0	?	?	?	?	0	1	0	2	0	1	0	A	0	0	1
suberosa subsp. litoralis Guatemala (50460)	0	0	1	0	1	A	0	2	0	4	?	0	1	1	0	0	1	0	0	0	0	0	1	1	1	?	?	?	?	0	0	0	0	2	0	1	0	0	1	0
suberosa subsp. litoralis Brazil (51299)	0	0	1	0	1	1	0	2	1	0	1	0	1	A	0	0	0	0	0	0	0	0	0	0	A	0	?	?	?	0	0	A	1	A	2	0	1	0	0	A
suberosa subsp. litoralis Mexico (5394)	0	C	0	0	1	1	A	E	0	0	0	0	1	1	0	0	0	0	0	0	0	0	A	1	1	?	?	?	?	0	0	0	1	0	0	A	1	0	0	0
suberosa subsp. suberosa Puerto Rico (5)	0	0	1	1	2	1	0	2	0	0	1	0	2	2	0	0	1	0	1	B	0	0	A	1	0	?	?	?	?	0	0	0	1	1	1	0	1	0	0	0
suberosa subsp. litoralis Fiji (7288)	0	0	E	E	1	1	1	2	0	0	A	0	1	1	0	0	A	0	0	0	0	0	0	0	0	?	?	?	?	0	0	0	1	0	2	0	1	0	0	1
suberosa subsp. litoralis Mexico (764)	0	0	0	0	1	1	0	1	C	0	0	0	1	0	0	0	0	0	0	0	0	0	A	A	0	?	?	?	?	0	0	0	0	2	0	A	3	0	0	0
suberosa subsp. litoralis Mexico (9262)	0	0	0	E	1	1	1	2	0	1	1	0	1	1	0	0	0	0	0	0	0	0	A	A	0	?	?	?	?	0	0	0	0	A	1	1	1	0	0	0
suberosa subsp. suberosa Dom. Rep. (94)	0	1	1	1	2	1	0	2	3	4	1	0	2	1	0	0	1	0	1	1	0	A	0	0	0	?	?	?	?	1	?	?	?	?	?	?	?	?	?	A
suberosa subsp. litoralis Venezuela (9567)	0	C	0	1	1	2	1	2	A	0	1	0	1	1	0	0	A	0	0	0	0	0	0	0	0	0	0	0	1	0	0	0	A	0	A	A	1	0	0	A
*tridactylites* Galapagos (9665)	1	0	0	A	3	1	0	3	0	0	2	0	2	0	0	B	1	0	3	0	2	A	0	A	0	?	?	?	?	0	2	2	0	0	2	0	2	0	2	1
suberosa subsp. litoralis Peru (97/922)	0	0	1	1	1	1	1	3	1	3	1	0	1	E	0	0	0	0	0	0	0	0	0	0	0	?	?	?	?	0	0	0	1	0	0	0	0	0	0	1
*pallida* Haiti (AP516)	A	C	0	0	1	1	0	1	0	0	0	0	1	5	0	0	0	0	0	0	0	0	0	0	A	?	?	?	?	0	1	0	1	1	1	0	0	0	0	1
suberosa subsp. litoralis S. America (DG0001)	0	0	E	A	1	1	1	1	0	A	0	0	1	1	A	0	0	0	0	0	0	0	0	0	0	?	?	?	?	?	?	?	?	?	?	?	?	?	?	?
suberosa subsp. suberosa Grenada (s.n.)	A	2	1	3	4	2	1	3	3	0	2	0	3	2	2	0	1	1	2	0	0	1	0	1	0	?	?	?	?	0	A	0	E	0	2	0	1	0	0	1
suberosa subsp. suberosa St. Croix (P-4)	0	0	1	2	1	2	0	2	0	0	1	0	2	3	1	1	1	1	1	0	0	1	1	1	0	?	?	?	?	0	A	0	1	0	2	0	1	0	0	1
*pallida* Jamaica (P-57)	0	3	0	5	0	0	1	0	0	5	4	1	0	5	0	0	0	0	0	2	0	0	0	0	1	?	?	?	?	0	0	0	0	0	0	1	1	0	0	0
*pallida* Dom. Rep. (SQFR1)	?	?	?	0	0	0	0	1	0	0	0	0	A	5	0	0	0	3	0	0	0	0	0	0	1	?	?	?	?	0	0	0	1	0	A	0	0	0	0	1

Taxon locality (Collection number)	1	2	3	4	5	6	7	8	9	0	1	2	3	4	5	6	7	8	9	0	1	2	3	4	5	6	7	8	9	0	1	2	3	4	5	6	7
suberosa subsp. suberosa Grenada (10859)	0	1	1	0	1	0	0	0	0	1	0	?	0	?	0	?	?	A	0	0	?	0	0	?	?	0	0	3	0	0	0	0	0	0	0	0	0
*tridactylites* Galapagos (1095)	0	1	1	2	0	0	0	0	0	1	0	?	?	?	0	?	?	0	0	0	?	0	1	?	?	?	1	3	0	0	1	0	0	0	0	0	0
suberosa subsp. suberosa Dom. Rep. (121)	0	1	0	0	0	0	0	0	?	1	0	?	0	?	0	?	?	0	0	0	?	0	0	?	?	1	1	2	0	0	0	0	0	0	0	0	0
suberosa subsp. litoralis Brazil (12264)	0	1	1	0	A	0	0	0	?	0	0	?	0	?	0	?	?	0	0	0	?	0	0	?	?	?	0	0	0	0	0	0	0	0	0	0	0
suberosa subsp. litoralis Mexico (1272)	0	2	A	0	A	0	0	0	0	0	0	?	0	?	0	?	?	?	0	0	?	0	0	?	?	0	0	4	0	0	1	0	0	0	1	0	0
suberosa subsp. litoralis El Salvador (1361)	0	3	1	0	0	1	0	0	0	0	0	?	0	?	0	?	?	0	0	0	?	0	0	?	?	?	0	3	1	0	1	0	0	1	1	0	0
suberosa subsp. litoralis Galapagos (1420)	0	2	0	2	3	0	0	0	0	0	0	?	0	?	0	?	?	2	0	0	?	0	0	?	?	?	0	3	0	0	0	0	0	0	0	0	0
*pallida* w/*suberosa* aff. USA-Florida (14284)	0	1	0	0	1	0	0	0	0	?	0	1	0	0	0	1	0	1	0	0	1	0	1	0	0	0	0	2	0	2	1	0	0	0	0	0	0
suberosa subsp. litoralis Costa Rica (1486)	1	3	2	0	0	0	0	0	0	0	0	0	1	0	0	0	0	A	0	0	1	1	1	0	0	?	2	3	0	3	1	0	0	B	1	1	0
suberosa subsp. litoralis Colombia (15029)	0	0	2	0	1	0	1	1	0	0	0	0	0	?	0	?	?	1	0	0	?	0	1	?	?	0	0	3	0	0	0	0	0	0	1	0	0
suberosa subsp. litoralis Ecuador (15217)	0	0	A	0	1	0	0	0	0	1	0	2	1	?	0	2	0	0	0	0	2	0	0	?	?	?	2	2	0	0	0	0	0	0	0	0	0
suberosa subsp. litoralis Colombia (16834)	0	1	1	1	1	0	0	0	0	0	0	0	0	?	0	?	?	1	0	0	?	0	0	?	?	0	2	3	0	0	0	0	0	0	0	0	0
suberosa subsp. litoralis Nicaragua (18533)	0	1	1	0	1	0	0	0	0	0	0	0	0	?	0	1	0	A	0	0	?	0	0	?	?	0	2	3	0	0	1	0	0	0	0	1	0
suberosa subsp. suberosa Hawaii-Oahu (188)	0	A	0	0	1	0	0	0	1	1	0	?	0	?	0	?	?	0	0	0	?	0	0	?	?	1	0	0	0	1	0	0	0	0	0	0	0
*pallida* USA-Florida (194)	0	3	0	0	1	0	0	0	?	0	0	?	0	?	0	?	?	1	0	0	?	0	1	?	?	0	2	0	0	0	0	0	0	0	0	0	0
suberosa subsp. litoralis Africa (2190)	0	0	1	0	1	0	0	0	?	0	0	?	0	?	0	?	?	A	0	0	?	0	1	?	?	0	0	0	0	0	0	0	0	0	1	0	0
suberosa subsp. litoralis Honduras (23340)	0	3	1	0	0	1	0	0	0	0	0	?	0	?	0	?	?	1	0	0	?	0	0	?	?	?	0	3	0	1	0	0	0	2	1	0	0
suberosa subsp. litoralis Colombia (2436)	0	0	1	0	1	0	0	0	0	0	0	?	0	?	0	?	?	0	0	0	?	0	1	?	?	?	0	3	0	0	1	0	0	0	0	0	0
suberosa subsp. suberosa St. Lucia (251)	0	3	2	0	1	0	0	0	0	1	0	?	0	?	0	?	?	0	0	0	?	0	0	?	?	0	0	3	0	0	0	1	0	0	1	0	0
suberosa subsp. litoralis Guatemala (25309)	0	1	1	0	1	0	0	0	0	0	1	?	0	?	0	?	?	0	0	0	?	0	0	?	?	?	1	2	0	0	1	0	0	0	0	?	0
*tridactylites* Galapagos (2620)	0	3	0	0	3	0	0	0	0	0	0	?	0	?	0	?	?	B	0	0	1	0	0	?	?	2	0	3	0	0	0	0	0	0	0	0	0
suberosa subsp. suberosa Caribbean (281)	0	1	1	0	E	0	0	0	1	0	0	2	0	0	0	2	0	0	1	0	2	0	0	1	0	3	0	0	0	0	0	0	0	0	0	1	0
suberosa subsp. litoralis Mexico (283)	1	2	0	0	3	0	0	0	0	0	0	?	0	?	0	?	?	3	0	0	?	0	0	?	?	?	2	3	0	0	0	0	0	0	0	0	0
suberosa subsp. litoralis Mexico (2966)	1	2	0	0	3	0	0	0	?	?	0	?	0	?	0	?	?	0	0	0	?	0	0	?	?	?	2	0	0	0	0	0	0	0	0	0	0
suberosa subsp. litoralis Colombia (3002)	0	0	1	0	1	0	0	0	1	0	0	?	0	?	0	?	?	1	0	0	?	0	0	?	?	?	0	3	0	0	0	0	0	0	1	0	0
suberosa subsp. litoralis Mexico (303)	0	3	1	0	0	0	1	0	0	0	0	?	0	?	0	?	?	1	0	0	0	0	1	0	0	?	0	0	0	0	1	0	0	0	0	0	0
suberosa subsp. litoralis Argentina (30408)	0	2	1	0	1	0	1	0	1	0	0	?	0	?	0	?	?	1	0	0	?	0	1	?	?	0	0	3	0	0	0	0	0	0	0	0	0
suberosa subsp. suberosa Dominica (3082)	0	0	1	0	0	0	0	0	0	1	0	2	0	?	0	?	?	0	0	0	?	0	0	?	?	?	2	2	1	0	0	0	0	1	1	0	0
suberosa subsp. litoralis Trinidad (31838)	0	1	1	0	1	0	0	0	0	0	1	?	0	?	1	?	?	A	0	1	?	0	1	?	?	?	0	1	0	0	0	0	0	0	1	1	0
*pallida* Jamaica (32)	0	2	0	0	C	0	0	0	?	0	0	?	0	?	0	?	?	0	0	0	?	0	0	?	?	?	0	0	1	2	0	0	0	1	0	0	0
suberosa subsp. litoralis Mexico (343)	0	3	0	0	0	0	1	0	0	0	0	0	0	0	0	0	0	A	0	0	0	0	1	0	0	?	1	2	0	0	1	0	0	0	0	1	0
suberosa subsp. litoralis Mexico (344)	0	1	0	0	0	0	0	0	0	0	0	0	0	1	0	0	0	0	0	0	0	0	0	0	0	?	2	0	0	0	1	0	0	0	0	0	0
*pallida* Mauritius (3558)	0	G	0	0	0	0	0	0	?	0	0	?	0	?	0	?	?	1	0	0	?	0	0	?	?	0	2	2	0	2	0	0	0	0	0	0	0
*pallida* Mexico (393)	0	3	0	0	0	0	0	0	0	0	0	3	1	0	0	3	2	1	0	0	3	1	1	3	3	?	0	0	0	1	1	0	0	2	0	0	0
*tridactylites* Galapagos (403)	0	3	0	2	3	0	0	0	1	1	0	?	0	?	0	?	?	2	0	0	?	0	0	?	?	2	0	3	0	0	1	0	0	0	0	0	0
*pallida* Mexico (403)	0	3	0	0	0	0	1	0	0	0	0	3	0	0	?	3	2	0	0	0	3	0	1	3	3	?	0	3	0	1	1	0	0	2	0	1	0
*pallida* Mexico (412)	?	?	?	?	?	0	0	0	0	0	0	0	0	0	0	0	0	0	0	0	0	0	1	0	0	?	1	2	0	1	1	0	0	2	0	0	0
*pallida* St. Croix (413)	0	1	0	0	0	0	0	0	?	1	0	?	0	?	0	?	?	0	0	0	?	0	0	?	?	?	2	2	0	1	0	0	0	2	0	?	0
*pallida* wl *suberosa* aff. Mexico (413)	0	1	0	0	2	0	0	0	0	0	0	0	0	0	0	0	0	1	1	0	0	0	1	0	0	?	0	G	0	0	1	0	0	0	0	1	0
suberosa subsp. litoralis Nicaragua (4162-b)	0	2	E	0	A	0	0	0	?	0	0	?	0	?	0	?	?	1	0	0	?	0	1	?	?	?	0	3	0	2	0	0	1	0	1	1	0
*pallida* USA-Florida (41876)	0	G	0	0	1	0	0	0	0	0	0	?	0	?	0	?	?	A	1	0	?	0	1	?	?	0	0	2	0	2	0	0	0	0	0	0	0
suberosa subsp. litoralis Peru (4514)	0	1	1	0	2	0	1	0	?	0	0	?	0	?	0	?	?	0	0	0	?	0	0	?	?	0	1	3	0	0	0	0	0	0	0	0	0
suberosa subsp. litoralis Bolivia (4707)	0	2	1	0	1	0	0	0	1	0	0	?	0	?	0	?	?	1	0	0	?	0	1	?	?	?	1	3	0	0	0	0	0	0	0	0	0
*pallida* Seychelles (49451)	0	E	0	0	1	0	0	0	0	0	0	?	0	?	0	?	?	0	1	0	?	0	1	?	?	0	0	2	0	1	0	0	0	2	0	1	0
suberosa subsp. litoralis El Salvador (495)	0	E	1	0	1	1	0	0	0	0	0	?	0	?	0	?	?	0	0	0	?	0	0	?	?	?	0	3	1	0	0	0	0	1	?	0	0
suberosa subsp. litoralis Guatemala (50460)	0	1	0	0	1	0	0	0	0	0	1	2	0	?	0	?	?	0	0	0	?	0	1	?	?	?	0	0	0	0	1	0	0	0	0	0	0
suberosa subsp. litoralis Brazil (51299)	0	0	1	0	1	0	1	0	0	0	0	?	0	?	0	?	?	1	0	0	?	0	0	?	?	0	0	3	0	3	0	0	0	0	0	0	0
suberosa subsp. litoralis Mexico (5394)	0	G	1	0	0	0	0	0	0	0	0	?	0	?	0	?	?	0	0	0	?	0	1	?	?	?	0	3	0	0	1	0	0	0	0	0	0
suberosa subsp. suberosa Puerto Rico (5)	0	0	0	0	0	0	0	0	1	1	0	2	0	0	0	?	?	0	0	0	?	0	0	?	?	1	0	0	0	0	0	0	0	0	0	0	0
suberosa subsp. litoralis Fiji (7288)	0	0	1	0	1	0	0	0	0	0	0	0	0	?	0	1	?	A	0	0	?	0	1	?	?	0	0	1	0	0	0	0	0	0	0	0	0
suberosa subsp. litoralis Mexico (764)	0	1	0	0	0	0	0	0	0	0	0	?	0	?	0	?	?	A	0	0	?	0	0	?	?	?	0	2	0	0	1	0	0	0	0	0	0
suberosa subsp. litoralis Mexico (9262)	0	A	0	0	A	0	0	0	0	0	0	?	0	?	0	?	?	0	0	0	?	0	0	?	?	0	2	4	0	0	0	0	0	0	0	1	0
suberosa subsp. suberosa Dom. Rep. (94)	0	A	A	0	0	0	0	0	1	1	0	?	0	?	0	?	?	0	0	0	?	0	0	?	?	?	3	2	0	1	0	0	0	0	0	0	0
suberosa subsp. litoralis Venezuela (9567)	0	1	1	A	2	0	0	1	0	0	0	?	0	?	0	?	?	1	0	0	?	0	0	1	2	0	0	3	0	0	0	0	0	0	0	1	0
*tridactylites* Galapagos (9665)	0	3	0	0	3	0	0	0	0	?	0	?	0	?	0	?	?	2	0	0	?	0	0	?	?	?	0	3	0	0	1	0	0	0	0	0	0
suberosa subsp. litoralis Peru (97/922)	0	G	1	0	1	0	0	0	?	0	0	?	1	?	0	?	?	1	0	0	?	0	0	?	?	?	2	3	0	0	0	0	0	0	1	0	0
*pallida* Haiti (AP516)	0	3	0	0	1	0	0	0	0	?	0	0	0	0	0	1	0	1	0	0	1	0	1	1	0	?	1	4	1	0	0	0	0	1	0	0	0
suberosa subsp. litoralis S. America (DG0001)	?	?	?	?	?	0	0	0	0	1	0	1	0	0	0	1	1	1	0	0	2	0	0	1	0	0	?	?	?	0	0	0	0	0	?	0	0
suberosa subsp. suberosa Grenada (s.n.)	A	1	1	0	0	0	0	0	?	1	0	?	0	?	0	?	?	A	1	0	?	0	0	?	?	?	0	2	0	0	0	0	0	0	1	0	0
suberosa subsp. suberosa St. Croix (P-4)	0	1	0	0	0	0	0	0	1	1	0	1	0	?	0	2	0	0	0	0	2	0	0	1	0	0	0	4	0	0	0	0	0	0	1	1	0
*pallida* Jamaica (P-57)	0	3	0	0	0	0	0	0	0	0	0	0	0	?	0	0	0	0	1	0	0	0	0	0	0	?	0	2	0	0	0	0	0	0	0	0	0
*pallida* Dom. Rep. (SQFR1)	0	1	0	0	1	0	0	0	0	?	0	0	0	0	0	0	1	1	0	0	1	1	1	1	0	0	2	3	0	0	0	0	0	0	0	0	0

Most of the *Passiflora pallida* accessions form a cluster toward the “base” of the neighbor joining tree, but four accessions of *Passiflora pallida* are placed elsewhere, intermixed with Passiflora suberosa subsp. litoralis, indicating that the differences between these two taxa are sometimes difficult to discern. There are many smaller clusters of Passiflora suberosa subsp. litoralis, all primarily positioned adjacently on the tree between the *Passiflora tridactylites*/Passiflora suberosa subsp. suberosa group and the *Passiflora pallida* group. These results suggest that both *Passiflora tridactylites* and Passiflora suberosa subsp. suberosa may have evolved from a non-monophyletic Passiflora suberosa subsp. litoralis. One small cluster of two accessions from coastal areas of Ecuador and Peru are located in a small group just “below” the larger *Passiflora tridactylites*/Passiflora suberosa subsp. suberosa cluster. However, the rest of the clusters of Passiflora suberosa subsp. litoralis contain individuals from mainly high elevations in Mexico and Central America with South American accessions scattered within them. In addition, two individuals, which may be of hybrid origin and possess affinities of *Passiflora pallida* and *Passiflora suberosa*, occur at two different locations in the neighbor joining tree, but they are both most similar to *Passiflora pallida*.

**Figure 17.**
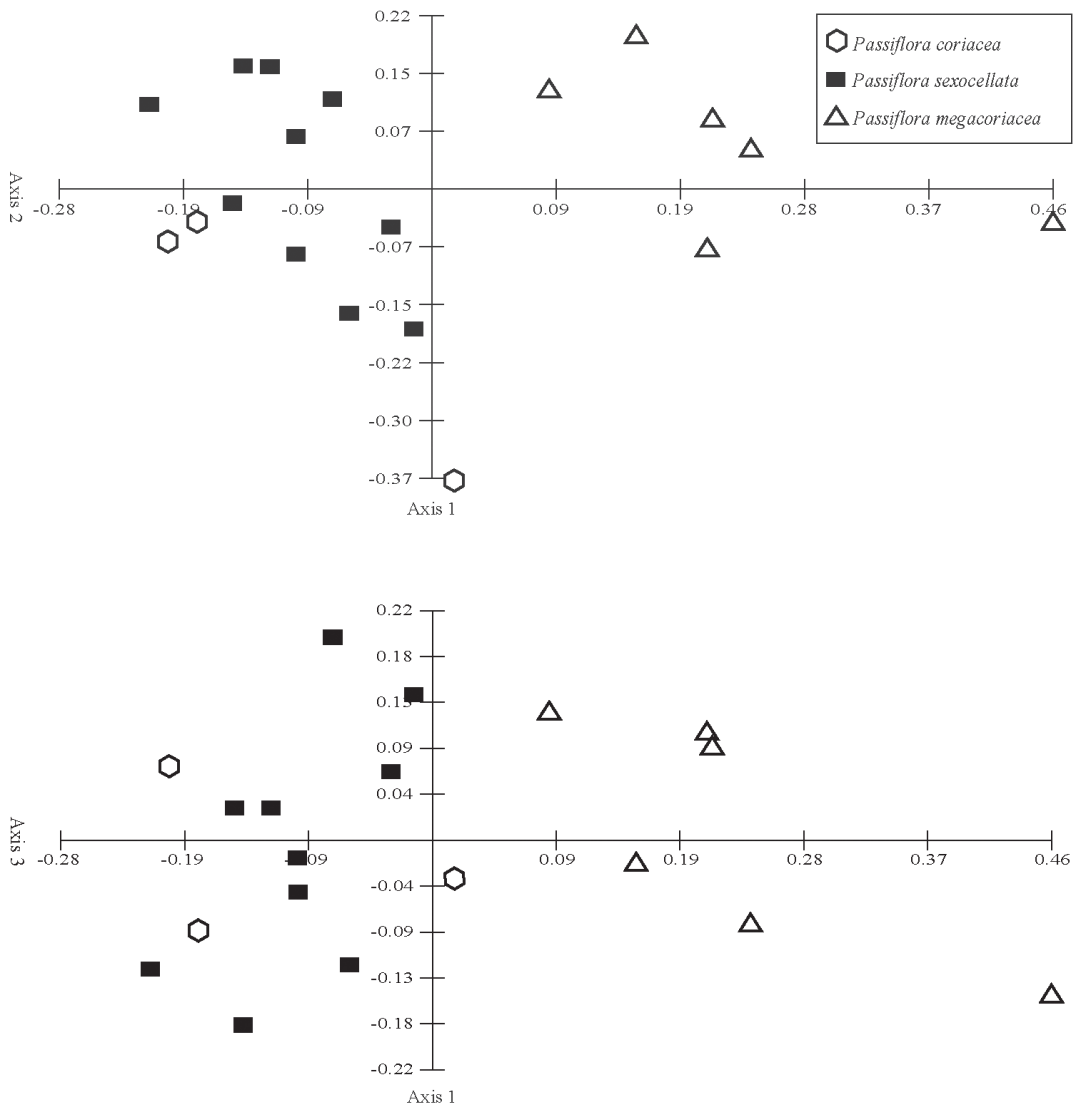
Principal components analysis of the data set for the *Passiflora coriacea* complex based on 44 morphological characters (Table 7).

*The* Passiflora coriacea *Complex.* A principal components analysis (PCA) of the entire morphological data set (Table 7) for the *Passiflora coriacea* complex is presented in Fig. 17. Species that are recognized in this revision as *Passiflora coriacea*, *Passiflora megacoriacea*, and *Passiflora sexocellata* are included and labeled accordingly. Principal components I, II, and III account for 30.8%, 19.2%, and 10.5% of the variation, respectively, for a total of 60.5%. Principal component axis I is most highly influenced by (presented in decreasing order of component loadings) (Table 8): (1) degree that the leaf is peltate, (2) stipe length, and (3) androgynophore length. Axis II is most highly influenced by (1) degree that the leaf is peltate, (2) leaf width, and (3) length of the lateral leaf lobe, and axis III by (1) pedicel length, (2) number of filaments in the inner coronal row, and (3) length of the pubescence on the stem. The PCA plots of axes I and II and I and III separate *Passiflora megacoriacea* from *Passiflora coriacea* and *Passiflora sexocellata*. The first principal component (PC1) and the third principal component (PC3) consist of information from both floral and vegetative characters. The second principal component (PC2) has low component loadings for the floral characters and high component loadings for the vegetative characters and is primarily an indicator of the degree that the leaf is peltate and leaf size. The graphs in Fig. 17 place individuals with larger flowers that have short floral stipes and long pedicels and larger leaves that are less peltate with narrower lateral lobes in the right half of the scatter plots.

**Table 7.** Characters used in the morphology-based principal components analysis of the *Passiflora coriacea* complex (* = characters used in the PCA analysis of the data set for the *Passiflora coriacea* complex based on floral characters) All measurements were recorded in mm. For a discussion of character state delimitation see Methods.

1.	*Pedicel length
2.	*Pedicel width
3.	*Stipe length
4.	*Stipe width
5.	*Hypanthium diameter
6.	*Sepal length
7.	*Sepal width
8.	*Number of filaments in outer coronal row
9.	*Length of filaments in outer coronal row
10.	*Width of filaments in outer coronal row
11.	*Number of filaments in inner coronal row
12.	*Length of filaments in inner coronal row
13.	*Width of filaments in inner coronal row
14.	*Stamen filament length
15.	*Stamen filament width
16.	*Anther length
17.	*Anther width
18.	*Style length
19.	*Style width
20.	*Stigma width
21.	*Nectary width
22.	*Androgynophore length
23.	*Androgynophore width
24.	*Ovary length
25.	*Ovary width
26.	*Operculum length
27.	*Limen floor diameter
28.	Distance of petiolar nectary from petiole base
29.	Petiole length
30.	Length of pubescence on petiole
31.	Petiolar nectary diameter
32.	Degree leaf peltate
33.	Lateral lobe length
34.	Lateral lobe width
35.	Central lobe length
36.	Number of laminar nectaries
37.	Laminar nectary diameter
38.	Angle between lateral lobes
39.	Leaf width
40.	Stem diameter
41.	Length of pubescence on stem
42.	Stipule length
43.	Stipule width
44.	Tendril width

**Table 8.** Component loadings for axes I, II, and III from a principal components analysis of the *Passiflora coriacea* complex (Fig. 17) The values were computed from quantitative vegetative and floral variables.

Variables	PCI	PCII	PCIII
Pedicel length	0.164	-0.012	0.826
Pedicel width	0.016	0.017	-0.050
Stipe length	-0.309	-0.092	0.056
Stipe width	0.005	0.045	0.004
Hypanthium diameter	0.190	0.104	0.044
Sepal length	0.211	0.188	-0.011
Sepal width	0.163	0.092	-0.057
Number of filaments in outer coronal row	-0.179	-0.107	0.000
Length of filaments in outer coronal row	0.182	0.187	-0.031
Width of filaments in outer coronal row	0.081	0.074	-0.011
Number of filaments in inner coronal row	-0.197	0.054	0.352
Length of filaments in inner coronal row	0.130	0.116	-0.008
Width of filaments in inner coronal row	-0.074	-0.010	-0.034
Staminal filament length	0.166	0.024	0.020
Staminal filament width	0.074	0.037	-0.043
Anther length	0.178	0.036	0.029
Anther width	0.074	0.055	0.003
Style length	0.141	0.068	-0.116
Style width	0.015	-0.002	-0.023
Stigma width	0.087	0.059	-0.069
Nectary width	0.135	0.072	0.015
Androgynophore length	0.246	0.125	0.028
Androgynophore width	0.031	0.073	-0.154
Ovary length	0.146	0.099	0.026
Ovary width	0.149	0.098	-0.022
Operculum length	0.163	0.079	-0.009
Limen floor diameter	0.211	0.136	-0.064
Distance of petiolar nectary from pet. base	0.126	0.302	0.116
Petiole length	-0.180	0.262	0.058
Length of pubescence on petiole	-0.050	-0.001	-0.117
Diameter of petiolar nectaries	0.014	0.018	0.002
Degree leaf peltate	-0.375	0.400	0.013
Length of lateral leaf lobe	-0.128	0.335	-0.015
Width of lateral leaf lobe	-0.223	0.307	-0.051
Length of central leaf lobe	0.125	0.302	-0.053
Angle between lateral leaf lobes	-0.109	0.054	-0.017
Leaf width	-0.183	0.367	-0.038
Number of laminar nectaries	0.037	-0.114	-0.192
Diameter of laminar nectaries	-0.095	-0.002	0.033
Stem diameter	-0.016	0.065	0.054
Length of pubescence on stem	0.122	-0.007	-0.194
Stipule length	0.013	0.001	0.106
Stipule width	-0.004	0.002	0.011
Tendril width	0.004	0.092	0.034

**Figure 18.**
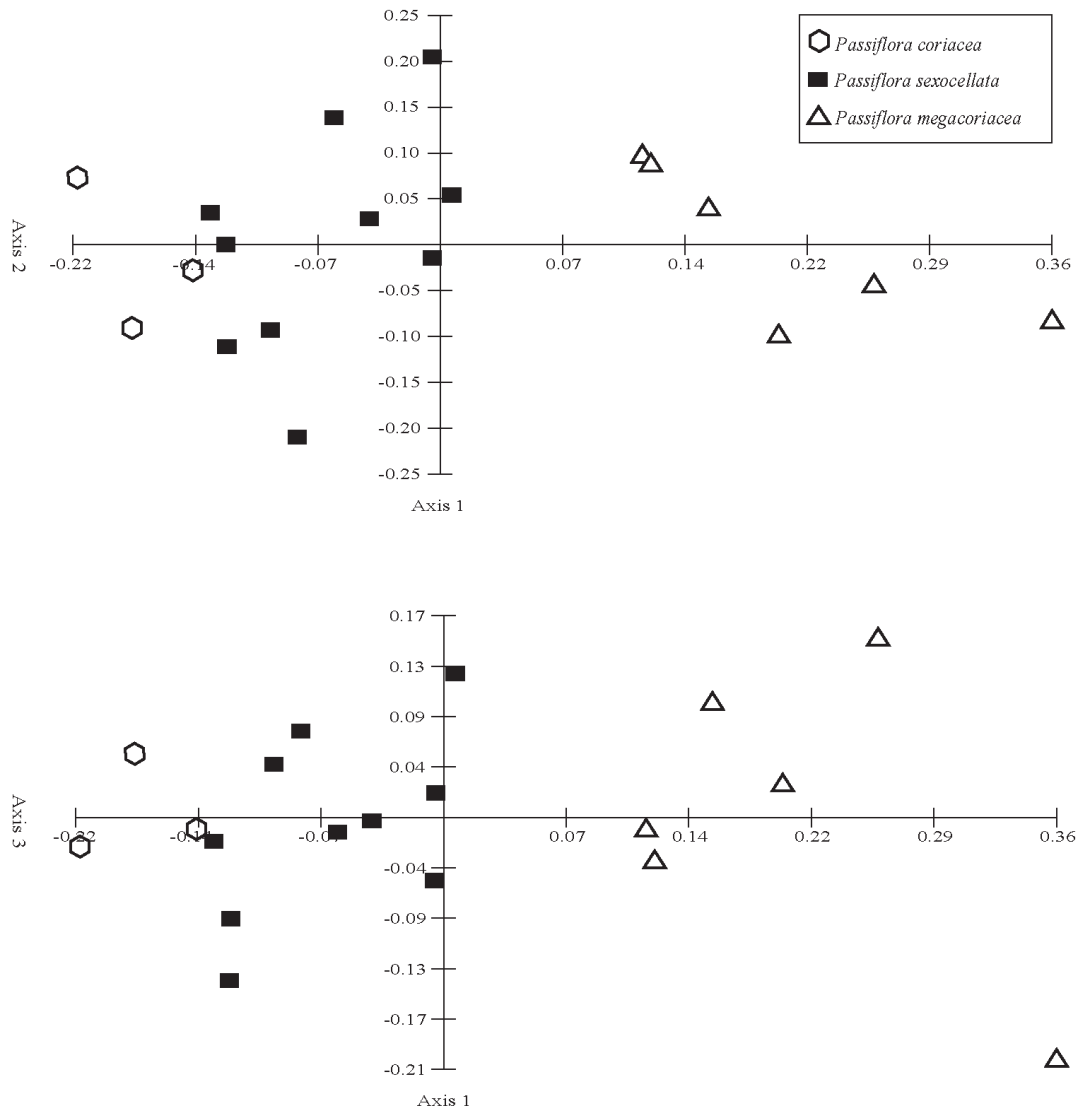
Principal components analysis of the data set for the *Passiflora coriacea* complex based upon 27 floral characters (Table 7).

**Figure 19.**
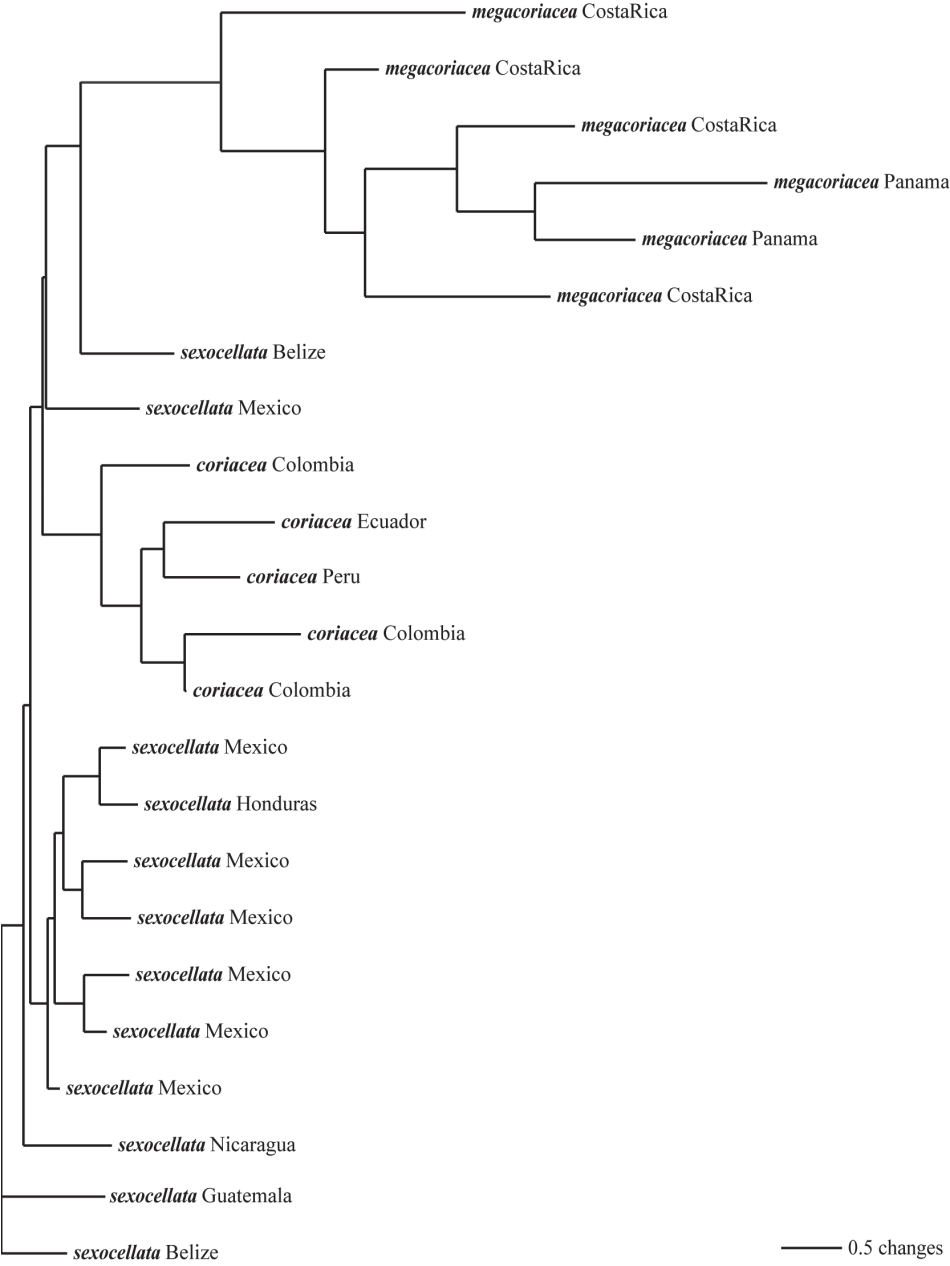
Unrooted neighbor joining tree resulting from the analysis of the morphological data from entities within the *Passiflora coriacea* complex based upon 38 morphological characters (Tables 10–11).

A PCA analysis of the data set for the *Passiflora coriacea* complex based on floral characters (Table 7) is presented in Fig. 18. Principal components I, II, and III account for 41.5%, 16.6%, and 12.0% of the variation, respectively, for a total of 70.1%. Principal component axis I is most highly influenced by (presented in decreasing order of component loadings)(Table 8): (1) stipe length, (2) androgynophore length, and (3) sepal length; axis II by (1) pedicel length, (2) number of filaments in the inner coronal row, and (3) androgynophore width; axis III by (1) stipe length, (2) number of filaments in the inner coronal row, and (3) nectary width. The PCA plots of axes I and II and I and III indicate that all three species, *Passiflora megacoriacea*, *Passiflora coriacea*, and *Passiflora sexocellata*, are phenetically separable. Individuals of the same species tend to cluster together although there is only a small amount of overlap among entities of *Passiflora coriacea* and *Passiflora sexocellata*. The pattern reflected in both plots in Fig. 19 strongly relates to the overall size of the flower, with entites in the right half of the scatter plot having larger flowers than those individuals in the left half.

The unrooted neighbor joining tree produced from an analysis of the entire morphological data set (Table 9–10) of the *Passiflora coriacea* complex is shown in Fig. 19. The accessions representing *Passiflora megacoriacea* and *Passiflora coriacea*, the two most morphologically distinct taxa in the *Passiflora coriacea* complex, are each clearly clustered toward the “top” of the neighbor joining tree. Accessions representing *Passiflora sexocellata* are clustered toward the “base” of the tree, but two accessions representing *Passiflora sexocellata* (from Belize and Mexico) are more similar to *Passiflora megacoriacea* than to other members of *Passiflora sexocellata* from Mexico and Central America. However, *Passiflora megacoriacea* can be easily separated from these accessions by its elongated androgynophore (the average androgynophore length of *Passiflora megacoriacea* is 7.5 mm and the average androgynophore lengths of the accessions of *Passiflora sexocellata* from Belize and Mexico are between 3 and 5 mm, respectively; char. #15).

**Table 9.** Component loadings for axes I, II, and III from a principal components analysis of the *Passiflora coriacea* complex (Fig. 18) The values were computed from quantitative floral variables.

Variables	PCI	PCII	PCIII
Pedicel length	0.192	0.919	-0.054
Pedicel width	0.017	-0.067	0.075
Stipe length	-0.358	-0.018	0.694
Stipe width	0.017	-0.019	0.057
Hypanthium diameter	0.241	0.000	0.231
Sepal length	0.283	-0.059	0.169
Sepal width	0.215	-0.072	0.140
Number of filaments in outer coronal row	-0.213	0.042	-0.066
Length of filaments in outer coronal row	0.268	-0.041	0.045
Width of filaments in outer coronal row	0.119	-0.009	0.018
Number of filaments in inner coronal row	-0.194	0.271	0.478
Length of filaments in inner coronal row	0.191	-0.004	0.062
Width of filaments in inner coronal row	-0.072	-0.026	0.041
Staminal filament length	0.173	0.006	0.021
Staminal filament width	0.092	-0.055	0.033
Anther length	0.186	-0.001	0.012
Anther width	0.099	-0.001	-0.086
Style length	0.165	-0.132	0.056
Style width	0.020	-0.024	0.032
Stigma width	0.115	-0.073	0.001
Nectary width	0.196	-0.021	0.324
Androgynophore length	0.290	-0.011	0.121
Androgynophore width	0.058	-0.171	0.033
Ovary length	0.185	0.031	0.034
Ovary width	0.199	-0.015	0.100
Operculum length	0.194	-0.023	0.016
Limen floor diameter	0.281	-0.079	0.135

**Table 10.** Characters used in the morphology-based neighbor joining analysis of the *Passiflora coriacea* complex. For a discussion of character state delimitation see Methods.

1.	Pedicel length (mm) ≤10.75 (0); 11.25–13.15 (1); ≥15.25 (2)
2.	Pedicel width (mm) ≤0.80 (0); ≥0.87 (1)
3.	Stipe length (mm) ≤3.88 (0); 4.00–9.38 (1); ≥10.88 (2)
4.	Stipe length/pedicel length (quotient) ≤0.56 (0); 0.66–1.20 (1); 1.45–1.60 (2); ≥2.05
5.	Hypanthium diameter (mm) ≤6.50 (0); 6.88–9.50 (1); 10.88–11.00 (2); 12.13–12.88 (3)
6.	Sepal length (mm) ≤10.88 (0); ≥11.25 (1)
7.	Sepal width (mm) ≤5.50 (0); >5.50 (1)
8.	Number of filaments in the outer coronal row (number) 25 (0); 31 (1); 34–42 (2); 43–51(3)
9.	Length of filaments in the outer coronal row (mm) ≤4.60 (0); 5.47–8.25 (1); ≥9.25
10.	Width of filaments in the outer coronal row (mm) ≤0.73 (0); ≥0.88 (1)
11.	Number of filaments in the inner coronal row (number) <10 (0); 27–43 (1); ≥45 (2)
12.	Length of filaments in the inner coronal row (mm) ≤1.91 (0); ≥2.20 (1)
13.	Width of filaments in the inner coronal row (mm) ≤2.50 (0); >2.50 (1)
14.	Nectary width (mm) ≤1.20 (0); 1.33–2.50 (1); ≥2.94 (2)
15.	Androgynophore length (mm) ≤5.94 (0); ≥7.00 (1)
16.	Androgynophore width (mm) ≤1.20 (0); 1.21–1.40 (1); ≥1.47 (2)
17.	Staminal filament length (mm) ≤2.87 (0); ≥3.00 (1)
18.	Staminal filament length/androgynophore length (quotient) ≤0.55 (0); 0.56–0.87 (1); ≥0.89 (2)
19.	Anther length (mm) ≤2.13 (0); 2.14–2.47 (1); 2.48–2.80 (2); 2.81–3.50 (3); ≥3.74 (4)
20.	Style length (mm) ≤2.40 (0); 2.54–3.94 (1); ≥4.00 (2)
21.	Operculum length (mm) <2.00 (0); ≥2.00 (1)
22.	Nectary height (mm) ≤0.80 (0); ≥1.40 (1)
23.	Limen floor diameter (mm) <4.94 (0); 4.94–5.27 (1); ≥5.60 (2)
24.	Seed length (mm) <3.75 (0); 3.75–4.80 (1); ≥4.88 (2)
25.	Seed width (mm) <3.00 (0); ≥3.00 (1)
26.	Position of petiolar nectaries (quotient: distance from petiolar nectary to petiole base/petiole length) <0.53 (0); ≥0.53 (1)
27.	Number of leaf lobes (number) 2 (0); 3(1)
28.	Central leaf vein length/central leaf lobe width (quotient) ≤0.76 (0); 0.93–1.54 (1); ≥1.63 (2)
29.	Lateral leaf vein length/central leaf vein length (quotient) ≤1.26 (0); 1.30–1.39 (1); >1.39 (2)
30.	Distance from the leaf outline to the leaf base (mm) ≤21 (0); 29–58 (1); ≥67 (2)
31.	Distance from the leaf outline to the sinus margin (mm) ≤4 (0); 5–12 (1); ≥14 (2)
32.	Leaf lobe depth (quotient: distance from leaf outline to margin of sinus/distance from leaf outline to leaf base) ≤0.24 (0); ≥0.26 (1)
33.	Angle between lateral leaf veins (degrees) ≤120 (0); ≥122 (1)
34.	Outer coronal filaments dark reddish purple at base with yellow apex (0); outer coronal filaments purplish at base and whitish toward tips (1)
35.	Limen erect or inclined toward the operculum (0); limen recurved (1)
36.	Nectary without raised annulus (0); nectary with raised annulus (1)
37.	Leaves transversely elliptic (0); leaves distinctly trilobed and ovate in general outline (1)
38.	Leaves not variegated (0); leaves variegated (1)

### Morphological analyses of supersection *Cieca*

**Figure 20.**
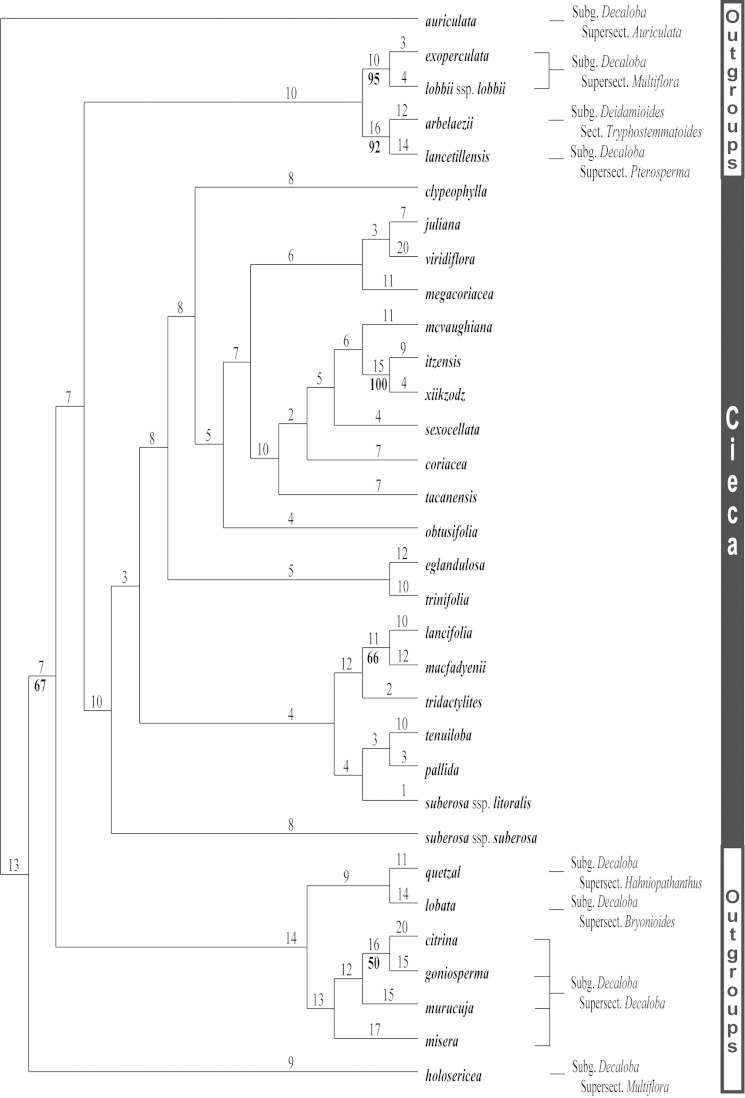
The single most parsimonious tree from the morphological data set of Passiflora supersection Cieca (Table 13) Numbers above branches are branch lengths. Bootstrap values are given below corresponding branches. Tree length = 548; CI = 0.429; RI = 0.526; RC = 0.226; HI = 0.571; G-fit = -48.398.

**Figure 21.**
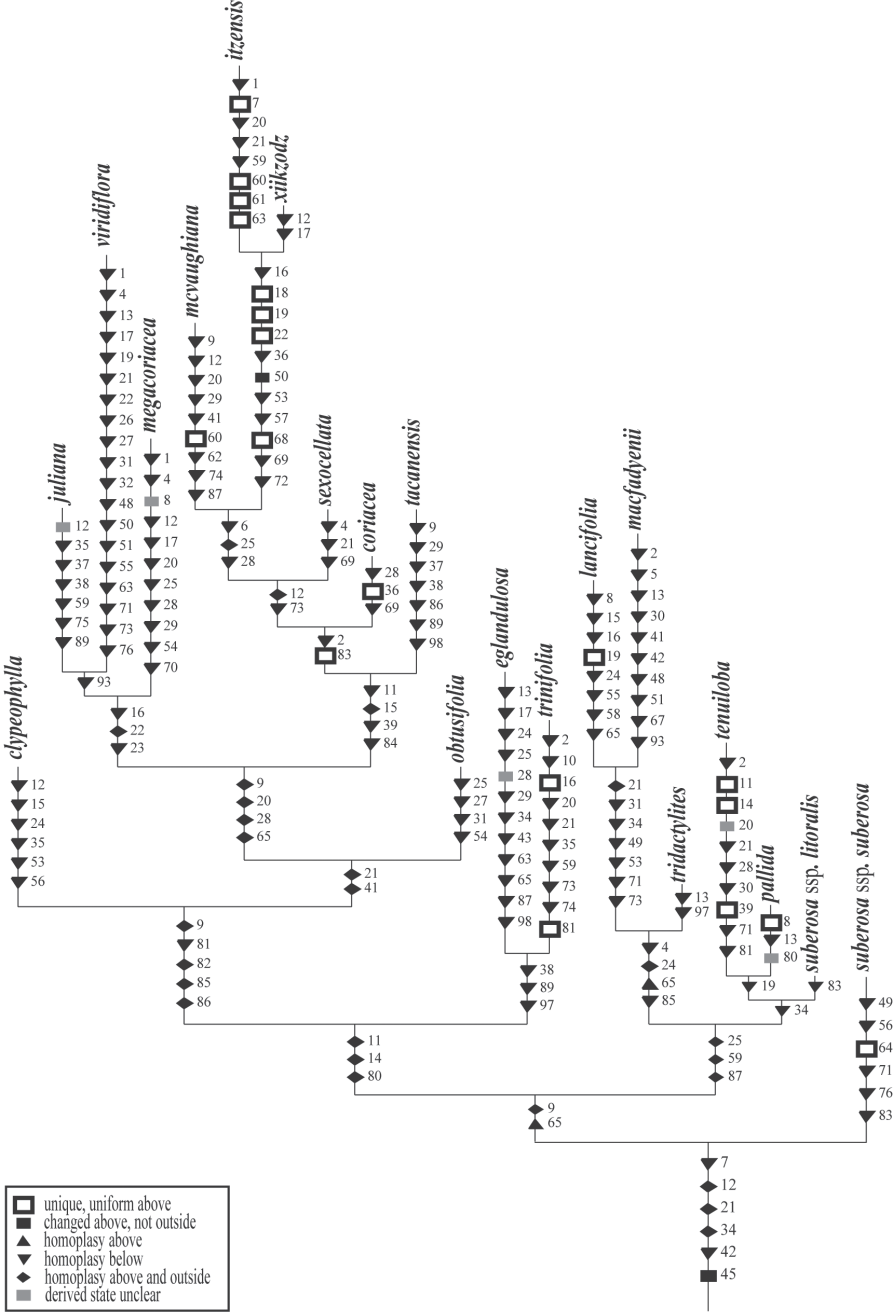
The single most parsimonious tree from the morphological data set of Passiflora supersection Cieca (Table 13) The numbers beside the branches and symbols on the branches indicate character changes. Tree length = 548; CI = 0.429; RI = 0.526; RC = 0.226; HI = 0.571; G-fit = -48.398.

The cladistic analysis of the morphological data (Tables 12–13) for supersection *Cieca* and outgroups resulted in the generation of one equally parsimonious tree of 548 steps, a consistency index (CI) of 0.429, a retention index (RI) of 0.526, and a rescaled consistency index (RC) of 0.226 (Figs 20–21). While only one tree was found in the analysis, branch support is low for most of the tree. Only six of the branches have bootstrap scores >50%, and only three have values where some confidence can be obtained: the branch grouping *Passiflora arbelaezii* L.Uribe and *Passiflora lancetillensis* J.M.MacDougal & J.Meerman has a bootstrap of 92%, the branch grouping Passiflora lobbii Mast. subsp. lobbii and *Passiflora exoperculata* Mast. has a bootstrap of 95%, and the branch grouping *Passiflora itzensis* and *Passiflora xiikzodz* has a bootstrap of 100%.

**Table 11.** Character values for taxa used in the phenetic analysis of the *Passiflora coriacea* complex (Fig. 19) A = 0/1; ? = condition unknown.

	Character																																					
										1										2										3								
Taxon locality (Collection number)	1	2	3	4	5	6	7	8	9	0	1	2	3	4	5	6	7	8	9	0	1	2	3	4	5	6	7	8	9	0	1	2	3	4	5	6	7	8
*sexocellata* Guatemala (1172)	0	0	1	1	1	0	1	3	1	0	1	1	1	2	0	1	0	0	2	1	0	0	1	?	?	0	0	?	2	?	?	?	1	?	0	0	?	0
*sexocellata* Belize (12214)	0	1	1	1	1	0	0	3	1	0	1	1	1	0	0	1	0	0	2	0	0	0	0	0	0	0	A	2	2	1	0	0	1	?	0	0	0	0
*sexocellata* Belize (1327)	0	0	1	3	1	1	0	3	1	0	1	1	1	0	0	2	0	0	2	2	0	0	0	?	?	0	0	?	2	?	?	?	1	?	0	1	0	0
*coriacea* Colombia (13808)	0	0	1	2	1	0	0	3	0	0	1	1	1	0	0	0	0	1	3	1	0	0	0	?	?	0	A	1	2	0	0	0	1	1	1	0	0	0
*sexocellata* Nicaragua (14759)	0	0	1	1	1	0	0	3	1	0	1	1	1	0	0	1	0	1	0	2	0	0	0	?	?	0	1	1	2	1	0	0	1	?	0	0	0	0
*sexocellata* Mexico (16155)	0	0	1	1	0	0	0	2	1	0	1	1	1	0	0	0	0	0	1	1	0	0	0	0	0	0	0	?	2	?	?	?	1	?	0	0	0	0
*coriacea* Ecuador (16532)	0	0	1	1	0	0	0	3	0	0	2	0	0	0	0	0	0	1	1	1	0	0	0	?	?	0	A	0	2	1	0	0	1	0	1	0	0	0
*megacoriacea* Costa Rica (184)	0	0	0	0	1	1	0	2	1	0	2	1	0	0	1	1	1	0	3	1	1	0	1	?	?	1	1	1	1	1	1	0	0	?	1	1	1	0
*sexocellata* Mexico (1888)	0	0	1	3	1	0	0	3	1	0	1	1	1	0	0	0	0	0	1	1	0	0	0	?	?	0	1	1	2	1	1	0	1	?	0	0	0	0
*sexocellata* Mexico (19595)	2	0	1	0	A	0	0	3	1	0	1	1	1	0	0	0	0	0	1	1	0	0	0	0	0	0	1	1	2	1	0	0	1	?	0	0	0	1
*sexocellata* Mexico (26567)	0	0	0	1	A	0	0	3	1	0	1	1	1	0	0	0	0	1	2	0	0	0	0	?	?	0	0	?	2	?	?	?	1	0	0	1	0	0
*megacoriacea* Costa Rica (2782)	0	A	1	1	3	1	1	1	2	1	1	1	1	1	1	2	1	0	3	1	1	0	2	?	?	0	0	?	2	?	?	?	1	?	0	1	0	0
*sexocellata* Honduras (3298)	0	0	1	2	0	0	0	2	1	0	1	1	1	0	0	0	0	0	0	1	0	0	0	0	0	0	A	1	2	1	0	0	1	?	0	0	0	0
*sexocellata* Mexico (3449)	0	0	1	1	1	0	0	3	1	0	1	1	1	0	0	0	0	0	2	A	0	0	0	0	0	0	A	1	2	1	0	0	1	0	0	0	0	0
*coriacea* Colombia (3752)	0	0	2	3	0	0	0	3	0	0	1	0	1	0	0	1	0	1	2	1	0	0	0	?	?	0	A	0	2	2	0	0	0	?	1	0	0	0
*coriacea* Peru (3823)	?	?	?	?	0	0	0	2	0	0	1	0	A	0	0	0	1	2	1	1	0	0	0	?	?	0	0	?	2	?	?	?	1	0	0	0	0	0
*megacoriacea* Panama (4473)	0	1	0	1	2	1	1	2	1	0	1	1	0	1	1	2	1	0	4	2	1	0	2	?	?	1	1	2	0	1	2	1	1	?	?	?	1	0
*sexocellata* Mexico (6045)	1	0	1	1	1	0	0	2	1	0	1	1	1	0	0	0	0	0	2	1	0	0	0	?	?	0	A	1	2	0	0	0	1	?	0	0	0	0
coriacea Colombia (761)	0	0	2	3	0	0	0	2	0	0	1	1	1	0	0	0	0	0	?	1	0	0	0	?	?	0	0	?	2	?	?	?	1	1	1	0	0	0
*megacoriacea* Costa Rica (79273)	0	0	0	0	1	1	0	2	1	0	1	1	0	1	1	0	1	A	4	1	1	0	A	?	?	1	A	1	2	1	0	0	1	?	1	1	0	0
*megacoriacea* Panama (86)	0	0	0	0	1	1	1	2	2	0	0	1	0	A	1	1	1	0	4	2	1	1	2	?	?	1	1	2	0	1	2	1	0	?	?	1	0	0
*megacoriacea* Costa Rica (89)	0	1	1	1	2	1	1	0	1	0	1	1	0	0	1	0	1	0	4	2	1	0	1	1	1	1	1	2	0	1	2	1	1	?	1	0	1	0
*sexocellata* Mexico (djcor)	1	0	1	0	1	0	0	3	1	0	1	1	1	0	0	0	0	0	2	1	0	0	0	?	?	0	A	?	2	?	?	?	1	?	0	0	0	0

**Table 12.** Characters used in the morphology-based cladistic analysis of Passiflora supersection Cieca. See Methods for a discussion of state delimitations and codings.

1.	Pedicel length (mm) ≤12.50 (0); 12.51–17.83 (1); ≥17.84 (2)
2.	Stipe length (mm) ≤2.75 (0); 2.76–8.00 (1); 8.01–14.00 (2); 14.01–19.00 (3); >19.00 (4)
3.	Stipe length/pedicel length (quotient) >2.86 (0); 2.53–2.86 (1); 0.1–2.52 (2); ≤0.14 (3)
4.	Hypanthium diameter (mm) >12.00 (0); 8.24–12.00 (1); 4.00–8.25 (2); 2.20–3.99 (3)
5.	Sepal length (mm) 15.76–21.00 (0); 7.13–15.75 (1); 4.85–7.12 (2); 21.01–23.28 (3); >23.38 (4)
6.	Sepal length/sepal width (quotient) 1.33–3.00 (0); 3.01–4.38 (1); >4.39 (2)
7.	Number of filaments in the outer coronal row (number) >60 (0); 41–60 (1); 31–40 (2); 21–30 (3); 17–20 (4)
8.	Length of filaments in the outer coronal row (mm) >15.00 (0); 7.67–15.00 (1); 5.87–7.66 (2); 5.00–5.86 (3); 1.22–4.99 (4)
9.	Length of filaments in the inner coronal row/sepal length (quotient) >0.75 (0); 0.35–0.75 (1); 0.25–0.34 (2); 0.09–0.24 (3)
10.	Number of filaments in the inner coronal row (number) >75 (0); 38–75 (1); 13–37 (2); 10–12 (3); 5–9 (4); 2–4 (5)
11.	Length of filaments in the inner coronal row (mm) >5.00 (0); 2.68–5.00 (1); 1.53–2.67 (2); 0.75–1.52 (3); 0 (4)
12.	Length of filaments in the inner coronal row/length of filaments in the outer coronal row (quotient) 0.15–0.36 (0); 0.37–0.73 (1); >0.73 (2)
13.	Length of staminal filaments (mm) >5.63 (0); 3.34–5.62 (1); 1.10–3.33 (2)
14.	Anther length (mm) 1.00–3.50 (0); >3.50 (1)
15.	Style length (mm) >6.00 (0); 2.00–6.00 (1); 0.81–1.99 (2)
16.	Ovary length (mm) >2.54 (0); 1.06–2.54 (1)
17.	Nectary width (mm) 0.30–0.59 (0); 0.60–1.22 (1); 1.23–2.00 (2); 2.01–3.54 (3); >3.54 (4)
18.	Androgynophore length (mm) 5.95–14.13 (0); 1.68–5.94 (1); 0–1.67 (2); >14.13 (3)
19.	Length of staminal filament/androgynophore length (quotient) 0.69–0.92 (0); >0.92 (1); 0.25–0.68 (2); 0.13–0.24 (3)
20.	Operculum length (mm) >4.79 (0); 3.00–4.79 (1); 1.87–2.99 (2); 1.28–1.86 (3); 0.73–1.27 (4); 0.29–0.72 (5)
21.	Nectary height (mm) >2.62 (0); 0.86–2.62 (1); 0.05–0.85 (2)
22.	Limen height (mm) >1.8 (0); 0.47–1.8 (1); 0.15–0.46 (2); 0.10–0.14 (3)
23.	Limen floor diameter (mm) 2.14–5.00 (0); 0.63–2.13 (1); 5.01–8.5 (2); >8.5 (3)
24.	Fruit length (mm) >30.00 (0); 25.00–30.00 (1); 17.14–24.99 (2); 9.10–17.13 (3)
25.	Fruit width (mm) >30.00 (0); 15–30 (1); 6–14 (2)
26.	Seed length (mm) >4.88 (0); 3.95–4.88 (1); 2.67–3.94 (2)
27.	Seed width (mm) >3.63 (0); 2.89–3.63 (1); 1.81–2.88 (2); 1.53–1.80 (3)
28.	Seed length/seed width (quotient) 1.21–1.42 (0); 1.43–1.82 (1); >1.82 (2)
29.	Central vein length (mm) >116.00; 44.00–116.00 (1); 19.00–43.00 (2); 3.00–18.00 (3)
30.	Lateral vein length (mm) 0 (0); 8.00–28.00 (1); 29.00–84.50 (2); >84.50 (3)
31.	Lateral vein length/central vein length (quotient) 0 (0); 0.31–1.02 (1); 1.03–2.97 (2); >2.97 (3)
32.	Central leaf vein/leaf width (quotient) >0.77 (0); 0.54–0.77 (1); 0.17–0.53 (2); 0.04–0.16 (3)
33.	Angle between primary lateral veins (degrees) 48.00–57.00 (0); 58.00–114.50 (1); 114.51–162.00 (2); >162.00 (3)
34.	Leaf lobe depth (quotient: distance from leaf outline to margin of sinus/distance from leaf outline to leaf base) not lobed (0); 0.02–0.11 (1); >0.11 (2)
35.	Degree peltate (distance from point of petiolar insertion to leaf base)(mm) not peltate (0); 0.34–2.12 (1); 2.13–7.83 (2); >7.83 (3)
36.	Position of petiolar nectary (quotient: distance from petiolar base to nectary/petiole length) 0.12–0.50 (0); 0.51–0.84 (1); >0.90 (2); no nectaries (3)
37.	Number of laminar nectaries (number) >3 (0); 1–3 (1); 0 (2)
38.	Stipule length (mm) 0.47–0.91 (0); 0.92–6.38 (1); >6.38 (2)
39.	Stipule width (mm) 0.09–1.38 (0); >2.5 (1)
40.	Inflorescences not present (0); inflorescences present as condensed shoots with aborted laminas (1)
41.	True peduncles branching off of tendril (0); true peduncles present but not branching off of tendril (1); true peduncles absent (2)
42.	Three floral bracts present (0); 0–2 floral bracts (1)
43.	Spur absent (0); spur present (1)
44.	Flowers actinomorphic (0); flowers zygomorphic (1)
45.	Petals present and half to three quarters the length of the sepals; petals present and less than half the length of the sepals (1); petals absent (2)
46.	Petals not fused (0); petals fused (1)
47.	Petals greenish white (0); petals greenish yellow (1); petals white (2); petals yellow (3); petals red (4)
48.	Sepals not fused (0); sepals fused (1)
49.	Sepals greenish white (0); sepals white (1); sepals greenish yellow (2); sepals yellow (3); sepals red (4)
50.	Three coronal rows present (0); two coronal rows present (1); one coronal row present (2); seven coronal rows present (3)
51.	Outer corona not adnate to perianth (0); outer corona adnate to perianth (1)
52.	Outer coronal filaments linear (0); outer coronal filaments distinctly tapering to a point toward apex (1); linear/capitate (2); fused into a tube (3)
53.	Outer coronal filaments without red/purple pigmentation (0); outer coronal filaments with a flush of red/purple pigmentation at base (1); outer coronal filaments with evident red/purple pigmentation (2); outer coronal filaments with band of red/purple pigmentation (3); outer coronal filaments with conspicuous red/purple pigmentation (4)
54.	Outer coronal filaments not distinctly capitellate, capitate or dilated toward apex (0); outer coronal filaments distinctly capitellate, capitate or dilated toward apex (1)
55.	Outer coronal filaments not connate (0); outer coronal filaments connate (1)
56.	Inner coronal filaments without red/purple pigmentation (0); inner coronal filaments with a flush of red/purple pigmentation at base (1); inner coronal filaments with evident red/purple pigmentation (2); inner coronal filaments with conspicuous red/purple pigmentation (3)
57.	Inner coronal filaments not distinctly capitellate, capitate or dilated toward apex (0); inner coronal filaments distinctly capitellate, capitate or dilated toward apex (1)
58.	Inner coronal filaments not connate (0); inner coronal filaments connate (1)
59.	Androgynophore without red/purple pigmentation (0); androgynophore with a flush of red/purple pigmentation at base (1); androgynophore with evident red/purple pigmentation (2); androgynophore with conspicuous red/purple pigmentation (3)
60.	Styles greenish yellow (0); styles very pale greenish yellow (1); styles pale greenish yellow with purplish spots and streaks (2); styles red (3); styles very dark reddish purple (4)
61.	Staminal filaments greenish yellow (0); staminal filaments pale greenish yellow with pink streaks at the base (1); staminal filaments red (2); staminal filaments very dark reddish purple (3)
62.	Anthers with red/purple pigmentation (0); anthers lacking red/purple pigmentation (1)
63.	Presentation of pollen subproximal to proximal (0); presentation of pollen lateral (1); presentation of pollen distal (2)
64.	Pollen yellow (0); pollen whitish (1)
65.	Ovary ellipsoid (0); ovary globose (1); ovary fusiform (2)
66.	Ovary not edged (0); ovary edged (1)
67.	Ovary glabrous (0); ovary with appressed, small, curved trichomes (1); ovary pubescent with dense long unicellular or multicellular hairs (2)
68.	Operculum plicate (0); operculum denticulate (1)
69.	Operculum without red/purple pigmentation (0); operculum with a flush of red/purple pigmentation at base (1); operculum with evident red/purple pigmentation (2); operculum with conspicuous red/purple pigmentation (3)
70.	Nectary without raised annulus (0); nectary with raised annulus (1)
71.	Nectary floor not sulcate (0); nectary floor sulcate (1)
72.	Limen present (0); limen absent (1)
73.	Limen recurved (0); limen erect or inclined toward operculum (1)
74.	Limen whitish (0); limen yellow (1); limen greenish yellow (2); limen greenish yellow with reddish purple spots and streaks (3); limen greenish yellow with a yellowish red tip (4); limen greenish yellow with a reddish purple tip (5); limen greenish yellow with purplish tip (6); limen bright reddish purple (7); limen red (8); limen very dark reddish purple (9)
75.	Limen floor without red/purple pigmentation (0); floor with a flush of red/purple pigmentation at base (1); floor with evident red/purple pigmentation (2); floor with conspicuous red/purple pigmentation (3)
76.	Fruits globose (0); fruits subglobose to widely ellipsoid (1); fruits ellipsoid (2); fruits ovoid (3); fruits ovoid with conical tip (4); fruits fusiform-ellipsoid (5)
77.	Fruits indehiscent (0); fruits dehiscent (1)
78.	New growth straight (0); new growth cernuous (1)
79.	Nine petiolar nectaries present (0); eight petiolar nectaries present (1); seven petiolar nectaries present (2); six petiolar nectaries present (3); five petiolar nectaries present (4); three petiolar nectaries present (5); two petiolar nectaries present (6); one petiolar nectary present (7); no petiolar nectaries present (8)
80.	Petiolar nectaries capitate (0); petiolar nectaries obconical (1); petiolar nectaries cupulate (2); petiolar nectaries discoid (3); petiolar nectaries auriculate (4)
81.	Leaves membranous (0); leaves chartaceous (1); leaves coriaceous (2); leaves sclerophyllous (3)
82.	Leaves not peltate (0); leaves peltate (1)
83.	Leaf venation with primary veins diverging and branching at base (0); leaf venation with secondary veins forming a series of loops (1); leaf venation with primary veins diverging and branching above base (2)
84.	Leaves unlobed (0); leaves bilobed (1); leaves bilobed and rarely trilobed (2); leaves trilobed (3); leaves trilobed and rarely unlobed or bilobed (4)
85.	Leaves ovate (0); leaves obovate (1); leaves transversely elliptic (2); leaves elliptic to circular (3)
86.	Leaves unlobed (0); leaves with the central lobe not narrowed at the base (1); leaves with the central lobe narrowed at the base (2)
87.	Leaf base cordate (0); leaf base not cordate (1)
88.	Leaf margin with 1–2 teeth or glandular denticulate at the leaf base (0); leaf margin entire (1)
89.	Laminar nectaries marginal (0); laminar nectaries submarginal, associated with minor veins of the abaxial surface and with several nectaries proximal to the lateral lobes (1); laminar nectaries submarginal and associated with minor veins of the abaxial surface (2), laminar nectaries present as ocellae between the central and lateral veins and with several nectaries proximal to the lateral lobes (3); laminar nectaries present as ocellae between the central and lateral veins (4); laminar nectary present at the very apex of the central vein (5); no laminar nectaries present (6)
90.	Stipules narrowly ovate (0); stipules ovate auriculate (1); stipules ovate (2)
91.	Stem terete or subterete (0); stem angled (1); stem 3-carinate (2)
92.	Plants without hooked trichomes (0); plants with hooked trichomes (1)
93.	Two prophylls present (0); one present (1)
94.	Seeds punctate-reticulate (0); seeds grooved-sulcate (1)
95.	Seeds with the chalazal beak inclined away from the raphe (0); chalazal beak erect (1); chalazal beak inclined toward the raphe (2)
96.	Chalazal beak well developed (0); chalazal beak poorly developed (1)
97.	Plants possessing C-glycosylflavones and lacking 3-O-glycosylflavonoids (0); plants lacking C-glycosylflavones and possessing 3-O-glycosylflavonoids (1)
98.	Chromosome number 2n = 18 (0); chromosome number 2n = 12 (1); chromosome number 2n = 24.

**Table 13.** Character values for taxa used in the morphological cladistic analysis of Passiflora supersection Cieca (Figs 20–21) A = 0/1; B = 0/2; C = 0/3; D = 0/1/2; E = 0/1/2/3; F = 0/1/2/4; G = 0/1/3; H = 1/2; I = 1/2/3; J = 1/3; K = 1/6; L = 2/3; M = 2/4; N = 2/5; O = 2/5/8/9; P = 2/6; Q = 3/4; R = 3/4/5; S = 3/6; T = 4/6; U = 5/6; V = 5/6/7; W = 6/7; X = 6/7/8; Y = 6/8; ? = condition unknown.

	**Character**																																																
										**1**										**2**										**3**										**4**									
Taxon	1	2	3	4	5	6	7	8	9	0	1	2	3	4	5	6	7	8	9	0	1	2	3	4	5	6	7	8	9	0	1	2	3	4	5	6	7	8	9	0	1	2	3	4	5	6	7	8	9
*abelaezii*	0	1	2	1	0	1	1	1	1	1	1	0	A	0	0	0	2	0	0	2	0	?	0	1	0	A	A	0	2	0	0	0	?	0	0	2	1	0	0	0	0	0	0	0	0	0	0	0	0
*auriculata*	A	0	2	?	1	1	1	A	0	1	H	0	?	?	?	?	?	1	1	?	?	?	?	3	H	D	H	1	A	?	1	0	?	A	0	0	0	0	0	0	2	0	0	0	1	0	2	0	2
*citrina*	2	0	3	1	4	2	4	3	3	T	Q	C	1	1	1	0	H	3	3	?	?	?	1	1	H	H	2	1	1	2	2	0	0	2	0	3	2	1	0	0	2	1	0	0	0	1	3	1	3
*exoperculata*	0	1	2	?	1	0	3	1	0	2	H	A	?	?	?	1	?	H	1	?	?	?	?	3	2	A	1	A	2	2	2	2	H	1	0	A	2	1	0	0	2	0	0	0	0	0	1	0	2
*goniosperma*	1	1	2	2	1	0	4	4	1	6	4	3	H	0	1	A	1	1	0	3	2	2	1	0	2	2	L	2	2	3	2	2	0	2	0	3	2	1	0	1	2	1	0	0	0	0	0	0	0
*holosericea*	A	0	2	?	A	1	1	1	A	1	D	1	?	0	?	?	?	A	?	?	?	?	?	H	1	H	H	1	A	2	1	0	?	A	0	0	0	1	0	0	1	0	0	0	0	0	2	0	1
*lancetillensis*	0	4	0	2	0	0	0	0	0	0	0	0	0	0	0	0	0	0	0	0	0	2	0	0	0	0	0	0	0	0	0	0	?	0	0	0	0	0	0	0	0	0	0	0	0	0	0	0	0
*lobata*	H	?	?	0	Q	1	1	A	1	6	4	3	?	1	0	0	?	0	2	A	?	?	?	4	0	0	0	A	A	3	1	0	1	H	0	D	0	?	1	0	2	0	0	1	0	0	2	0	1
lobbii subsp. lobbii	0	1	2	?	1	0	3	3	1	1	1	2	?	?	?	1	?	1	1	?	?	?	?	L	H	0	1	0	2	1	2	2	1	2	0	A	2	H	0	0	2	0	0	0	0	0	0	0	2
*misera*	2	0	3	0	0	0	1	0	0	1	1	0	0	1	0	0	2	0	0	2	1	1	2	3	2	2	2	0	L	2	2	2	2	0	A	3	0	1	0	0	2	0	0	0	0	0	2	0	0
*murucuja*	0	1	2	0	4	1	?	0	1	6	4	3	0	1	0	0	4	3	3	0	0	4	3	3	2	2	L	A	3	1	2	2	2	0	0	3	0	1	0	0	2	0	0	0	0	0	4	0	4
*quetzal*	2	A	3	0	0	0	?	0	0	?	0	1	0	1	0	0	?	0	0	0	?	?	?	0	0	0	A	1	1	?	1	0	0	A	2	0	0	2	0	0	2	1	0	0	0	0	2	0	0
*clypeophylla*	1	2	2	2	1	0	3	3	1	2	2	1	1	0	1	1	1	1	1	3	2	2	0	?	?	?	?	?	1	2	1	1	2	1	3	0	1	1	0	0	2	1	0	0	2	?	?	0	2
*coriacea*	O	H	1	2	1	0	1	4	1	H	2	1	2	0	1	1	1	1	0	3	2	2	0	L	1	2	2	1	2	2	2	2	2	1	2	0	0	1	0	1	2	1	0	0	2	?	?	0	2
*eglandulosa*	1	1	2	2	H	0	3	4	1	2	3	A	2	0	1	1	1	1	1	3	2	2	1	3	2	A	1	1	1	2	1	1	2	2	0	3	2	2	1	0	2	1	1	0	2	?	?	0	2
*itzensis*	0	2	0	2	1	0	3	2	0	2	1	1	2	0	2	1	5	2	?	5	3	4	0	1	2	0	2	2	2	2	2	2	2	1	2	1	0	1	0	1	2	1	0	0	2	?	?	0	2
*juliana*	A	0	L	2	1	0	1	L	1	1	1	1	1	1	1	0	1	1	0	2	1	1	0	L	H	1	2	1	1	2	1	1	2	2	2	0	0	2	1	1	2	1	0	0	2	?	?	0	2
*lancifolia*	2	1	2	1	3	2	3	1	2	U	2	G	0	0	1	0	3	3	2	2	2	1	B	3	2	2	2	1	1	1	1	0	1	1	0	1	2	H	0	0	2	1	0	0	2	?	?	0	4
*macfadyneii*	1	A	2	2	3	2	3	4	3	S	3	C	0	0	1	0	H	3	2	3	2	2	0	1	2	2	3	2	2	1	1	0	1	2	0	1	2	1	0	1	2	1	0	0	2	?	?	1	4
*mcvaughiana*	D	1	2	2	1	0	2	3	A	1	L	A	2	0	1	1	1	1	0	3	2	2	0	3	2	0	0	0	2	2	2	2	2	1	2	J	2	1	0	0	2	1	0	0	2	?	?	0	2
*megacoriacea*	0	1	2	A	1	0	2	1	1	H	1	1	1	A	1	0	H	0	2	2	H	1	2	2	1	0	1	1	H	2	H	H	2	H	2	1	0	1	0	1	2	1	0	0	2	?	?	0	2
*obtusifolia*	0	1	2	2	2	0	L	4	1	1	2	2	2	0	1	1	1	1	A	L	2	2	A	2	1	2	2	1	H	B	A	1	H	B	B	1	1	1	0	1	2	1	0	0	2	?	?	0	2
*pallida*	0	A	2	3	2	0	3	4	1	2	3	1	2	0	1	1	0	1	A	4	2	2	1	3	2	2	2	1	H	D	A	0	1	B	A	1	2	1	0	0	2	1	0	0	2	?	?	0	B
*sexocellata*	0	1	2	2	1	0	1	2	A	2	1	1	2	0	1	1	1	1	2	3	2	2	0	2	1	1	2	1	2	2	2	2	2	1	2	0	0	1	0	1	2	1	0	0	2	?	?	0	2
suberosa subsp. suberosa	0	1	2	2	H	0	3	4	1	2	2	1	2	0	1	1	1	1	0	Q	2	2	0	3	2	2	2	1	1	H	A	0	1	2	B	A	H	1	0	A	2	1	A	0	2	?	?	0	B
suberosa subsp. litoralis	0	1	2	2	1	A	3	Q	1	2	H	1	1	0	1	0	H	A	0	L	2	2	0	3	2	2	2	1	1	B	A	0	1	B	B	A	A	1	0	0	2	1	0	0	2	?	?	0	1
*tacana*	?	?	?	?	?	?	?	?	?	?	?	?	?	?	?	?	?	?	?	?	?	?	?	2	1	1	1	1	1	2	2	2	2	1	2	0	2	2	1	?	2	1	?	?	?	?	?	?	?
*tenuiloba*	0	0	2	2	2	0	H	4	1	1	2	1	2	0	1	1	0	1	0	4	2	2	A	3	2	1	2	2	3	2	3	3	3	2	0	1	2	1	0	0	2	1	0	0	2	?	?	0	2
*tridactylites*	1	1	2	2	1	1	3	H	1	2	1	1	1	0	1	0	1	0	2	2	2	2	0	3	2	2	3	1	H	H	1	A	H	2	0	1	2	1	0	0	2	1	0	0	2	?	?	0	2
*trinifolia*	0	0	2	2	1	0	2	4	1	1	2	1	1	0	1	1	1	1	1	3	2	3	0	3	2	2	2	1	2	1	1	1	H	2	A	A	2	H	1	0	2	1	0	0	2	?	?	0	2
*viridiflora*	0	1	2	2	3	2	1	4	3	6	4	3	1	1	1	0	2	3	3	1	1	1	1	L	1	1	H	1	H	2	1	1	2	2	2	0	2	1	0	1	2	1	0	0	2	?	?	1	2
*xiikzodz*	0	3	0	2	1	0	1	1	3	1	1	1	2	0	1	A	5	1	0	5	3	4	2	H	H	0	2	2	2	2	2	2	H	H	2	1	0	1	0	1	2	1	A	0	2	?	?	0	2

	Character																																																
	5										6										7										8										9								
Taxon	0	1	2	3	4	5	6	7	8	9	0	1	2	3	4	5	6	7	8	9	0	1	2	3	4	5	6	7	8	9	0	1	2	3	4	5	6	7	8	9	0	1	2	3	4	5	6	7	8
*abelaezii*	1	0	0	0	0	0	0	0	0	0	0	0	0	0	0	0	0	0	0	0	0	1	0	0	1	0	1	0	0	R	2	0	0	1	0	0	0	0	1	5	0	0	0	0	A	2	0	?	3
*auriculata*	1	0	1	2	0	0	0	1	0	0	0	0	0	0	0	0	0	2	0	1	0	0	0	?	1	0	0	0	0	6	4	1	0	0	C	0	A	1	1	3	0	0	0	1	1	1	1	0	?
*citrina*	H	1	0	0	0	1	0	0	1	1	1	1	0	1	0	0	1	2	0	0	0	1	0	0	0	0	N	?	1	8	?	1	0	0	3	1	1	0	1	6	0	2	0	1	1	2	0	0	2
*exoperculata*	1	0	0	2	0	0	2	1	0	A	0	0	1	0	0	0	0	0	0	A	0	0	0	?	?	?	0	0	0	6	2	2	0	0	J	2	1	0	1	6	0	0	0	0	0	2	?	?	?
*goniosperma*	2	0	0	2	0	0	?	?	?	0	0	0	0	0	0	0	1	1	0	2	1	0	0	1	2	0	5	0	1	8	?	1	0	0	1	1	1	0	1	6	0	2	0	1	1	2	0	?	?
*holosericea*	1	0	1	2	0	0	2	1	0	2	0	0	1	0	0	0	0	2	0	2	0	0	0	?	8	2	A	0	0	6	2	1	0	0	3	0	2	0	0	2	0	0	0	1	0	2	1	0	1
*lancetillensis*	0	0	0	0	0	0	0	0	0	0	0	0	1	0	0	0	0	2	0	0	0	0	0	0	1	0	1	0	1	D	1	2	0	1	0	0	0	0	1	2	0	0	0	0	0	0	0	?	0
*lobata*	2	0	0	C	0	0	?	?	?	0	0	0	1	0	0	0	0	0	0	H	1	0	0	0	0	0	H	1	0	U	D	1	0	0	3	0	H	0	0	1	0	3	1	1	0	2	0	0	1
lobbii subsp. lobbii	1	0	1	2	0	0	2	1	0	A	0	0	1	0	0	0	0	0	0	A	0	0	0	?	?	?	0	0	0	6	2	2	0	0	J	2	1	0	1	6	0	0	0	0	0	2	?	1	?
*misera*	1	0	0	3	0	0	0	1	0	0	0	0	0	0	0	0	0	0	0	0	0	0	0	0	0	0	C	0	1	8	?	1	1	2	1	2	1	0	1	4	0	2	0	1	1	1	1	?	?
*murucuja*	2	0	3	0	0	1	?	?	?	2	3	2	0	0	0	0	0	0	0	2	1	1	1	?	?	2	0	0	1	8	?	1	0	0	1	2	1	0	1	4	0	2	0	1	1	2	0	?	?
*quetzal*	A	0	0	?	0	0	?	1	0	0	?	?	1	0	0	0	0	0	0	?	0	0	0	?	?	?	2	0	1	6	H	1	1	2	3	3	1	0	0	0	1	0	0	0	0	2	0	?	?
*clypeophylla*	1	0	0	2	0	0	2	1	0	?	0	0	1	?	0	1	0	0	0	2	0	0	0	0	?	?	?	?	?	6	3	2	1	2	3	0	1	1	1	2	0	0	0	1	?	?	?	?	?
*coriacea*	1	0	0	3	0	0	2	1	0	1	0	0	1	0	0	0	0	0	0	2	0	0	0	0	7	1	0	0	0	6	3	2	1	2	2	2	1	1	1	1	0	0	0	1	0	2	0	?	?
*eglandulosa*	1	0	0	0	0	0	0	1	0	0	0	0	1	1	0	0	0	0	0	0	0	0	0	0	2	0	H	0	0	8	?	1	0	0	3	0	1	0	1	6	2	0	0	1	0	1	1	1	?
*itzensis*	3	0	0	4	0	0	3	0	?	3	4	3	1	2	0	0	0	0	1	3	?	?	1	?	?	3	4	0	0	6	3	2	1	2	2	2	1	1	1	1	0	0	0	1	0	2	0	?	2
*juliana*	1	0	1	A	0	0	A	1	0	2	0	0	1	0	0	0	0	0	0	0	0	0	0	0	P	2	A	0	0	6	L	2	1	2	3	0	2	1	1	2	2	0	0	1	0	H	0	1	2
*lancifolia*	H	0	0	4	0	1	?	A	1	?	0	0	1	1	0	0	0	0	0	?	0	1	0	1	?	?	0	0	?	X	D	1	0	0	3	0	1	1	1	6	0	0	0	1	0	2	0	?	?
*macfadyneii*	H	1	0	4	0	0	?	A	0	?	0	0	1	1	0	2	0	2	0	?	0	1	0	1	?	?	5	0	0	X	H	1	0	0	3	0	2	1	1	6	0	0	0	1	0	2	0	1	?
*mcvaughiana*	1	0	0	2	0	0	1	1	0	2	2	0	0	0	0	0	0	0	0	0	0	0	0	1	6	1	1	0	0	X	L	2	1	2	2	2	1	1	1	6	0	0	0	1	0	H	0	1	?
*megacoriacea*	1	0	2	0	1	0	0	1	0	A	0	0	1	0	0	0	0	A	0	A	1	0	0	0	2	0	0	0	0	6	L	2	1	2	J	B	1	1	1	1	0	0	0	1	0	2	0	1	?
*obtusifolia*	1	0	2	A	1	0	A	1	0	0	0	0	1	0	0	1	0	B	0	0	0	0	0	0	2	0	1	0	0	6	L	2	A	B	3	B	A	A	1	P	0	0	0	1	0	2	0	?	2
*pallida*	1	0	0	F	0	0	E	1	0	G	0	0	1	A	0	A	0	A	0	E	0	0	0	A	0	E	0	0	0	V	A	1	A	D	G	O	D	A	1	6	0	0	0	1	0	2	0	1	3
*sexocellata*	1	0	0	2	0	0	A	1	0	2	0	0	1	0	0	A	0	A	0	1	0	0	0	1	9	3	0	0	0	U	L	2	1	2	2	2	1	1	1	1	0	0	0	1	0	2	0	?	2
suberosa subsp. suberosa	H	0	0	A	0	0	A	1	0	D	0	0	1	A	0	A	0	B	0	D	0	0	0	A	N	A	A	0	0	W	D	1	A	D	4	0	D	A	1	P	0	0	0	1	0	2	0	1	L
suberosa subsp. litoralis	1	0	0	2	0	0	2	1	0	0	0	0	1	0	1	0	0	A	0	2	0	1	0	0	5	0	3	0	0	6	E	1	A	D	4	0	D	0	1	P	0	0	0	1	0	2	0	1	?
*tacana*	?	?	?	?	?	?	?	?	?	?	?	?	?	?	?	?	?	?	?	?	?	?	?	?	?	?	0	0	?	6	3	2	1	0	3	2	1	1	1	K	2	0	0	1	0	2	1	?	?
*tenuiloba*	1	0	0	D	0	0	D	1	0	1	0	0	A	0	0	1	0	0	0	B	0	1	0	0	2	A	0	0	0	6	L	2	0	0	3	B	2	0	1	6	0	0	0	1	0	2	0	1	?
*tridactylites*	1	0	0	A	0	0	1	1	0	1	0	0	1	?	0	2	0	0	0	2	0	0	0	0	M	A	5	0	0	6	I	1	0	0	3	0	1	1	1	6	0	0	0	1	0	1	0	?	?
*trinifolia*	1	0	0	A	0	0	A	1	0	1	0	0	1	0	0	1	0	0	0	B	0	0	0	1	3	A	?	0	0	X	3	3	A	B	3	0	1	0	1	2	2	0	0	1	0	1	0	?	?
*viridiflora*	2	1	1	0	0	1	?	?	?	0	0	0	1	1	0	0	0	0	0	0	0	1	0	1	2	0	4	0	0	Y	3	2	1	2	3	0	2	1	1	2	0	0	0	1	0	H	0	1	?
*xiikzodz*	3	0	0	4	0	0	3	0	0	2	0	0	1	0	0	0	0	0	1	3	?	?	1	?	?	3	3	0	0	6	3	2	1	2	2	2	1	1	1	1	0	0	0	1	0	2	0	?	2

The presence/absence of petals (character # 45, state 2) was given a weight of two in this analysis (all other characters having a weight of one) in order to insure that the ingroup is resolved as monophyletic; the monophyly of the supersection has already been confidently established (100% bootstrap value) with molecular sequence data. The monophyly of supersection *Cieca* is supported by the following apomorphies: the absence of petals, styles that are less than 6 mm in length (#15, 1), outer coronal filaments that are commonly less than 5 mm in length (#8, 4), fewer than 35 filaments present in the inner coronal row (#10, 2), staminal filaments that are frequently less than 5.5 mm long (#13, 1), and 0–2 floral bracts (#42, 1). The absence of petals is the only nonhomoplasious character supporting the monophyly of the supersection. Within supersection *Cieca*, *Passiflora clypeophylla*, *Passiflora juliana*, *Passiflora viridiflora*, *Passiflora megacoriacea*, *Passiflora mcvaughiana*, *Passiflora xiikzodz*, *Passiflora itzensis*, *Passiflora sexocellata*, *Passiflora coriacea*, *Passiflora tacanensis*, and *Passiflora obtusifolia* form a clade. Within this group, *Passiflora juliana*, *Passiflora viridiflora*, and *Passiflora megacoriacea* constitute a clade, with *Passiflora juliana* and *Passiflora viridiflora* being most closely related. A clade consisting of *Passiflora mcvaughiana*, *Passiflora xiikzodz*, *Passiflora itzensis*, *Passiflora tacanensis*, *Passiflora sexocellata*, and *Passiflora coriacea* is also evident, with *Passiflora xiikzodz* and *Passiflora itzensis* present as sister species. The two Guatemalan endemics, *Passiflora trinifolia* and *Passiflora eglandulosa*, form a clade. A clade consisting of *Passiflora tridactylites*, *Passiflora lancifolia*, and *Passiflora macfadyenii* is also present, with *Passiflora lancifolia* and *Passiflora macfadyenii* sister to each other. *Passiflora tenuiloba*, *Passiflora pallida*, and Passiflora suberosa subsp. litoralis form a clade, with *Passiflora tenuiloba* and *Passiflora pallida* being most closely related. Finally, Passiflora suberosa subsp. suberosa is cladistically basal within the supersection.

Passiflora suberosa subsp. suberosa is defined by the presence of white sepals (#49, 1), filaments in the inner coronal row that are reddish purple at the base with a yellow capitate head (#56, 2), whitish pollen (#64, 1), a sulcate floral nectary floor (#71, 1), ovoid fruits (#76, 3) and three-lobed leaves (rarely unlobed or bilobed)(#84, 4). The remaining members of the supersection form a clade based upon a shift from plants that commonly possess four or more laminar nectaries to those that possess none (#37, 2)(with shifts to leaves with less than four and more than four laminar nectaries occurring in many taxa) and ellipsoid ovaries (#65, 1)(with several shifts to globose and fusiform ovaries in several taxa).

Within Passiflora supersection Cieca Passiflora lancifolia, *Passiflora macfadyenii*, *Passiflora tridactylites*, *Passiflora tenuiloba*, *Passiflora pallida*, and Passiflora suberosa subsp. litoralis form a clade (Figs 20–21). This group possesses petiolar nectaries that are positioned on the upper half of the petiole (#36, 1) and androgynophores that possess red or purple pigmentation (#59, 1). The taxa commonly lack laminar nectaries, but when laminar nectaries are present they are submarginal glands that are associated with minor veins of the abaxial surface and never occur proximal to the lateral leaf veins (#89, 6). *Passiflora lancifolia* and *Passiflora macfadyenii* form a clade with 66% bootstrap support. *Passiflora lancifolia* and *Passiflora macfadyenii* are both Jamaican endemics and possess the following synapomorphies: sepals that are greatly elongated (21.06–24.13 mm)(#5, 3), long staminal filaments (commonly greater than 6 mm)(#13, 0), no inner coronal filaments or a very reduced inner corona (0–10 filaments)(#10, 6), an erect limen (#73, 1), a distinctly sulcate floral nectary floor (#71, 1), and bright red flowers (#49, 4; #53, 4). *Passiflora lancifolia* is unique in having the widest floral nectary in the supersection (2.08–3.00 mm)(#17, 3). It is also defined by its long pedicel (> 20 mm)(#1, 2), wide hypanthium (8.34–10.31 mm)(#4, 1), connate inner and outer coronal filaments (#55, 1; #58, 1), tall limen (commonly 0.44–1.10 mm)(#22, 1), ellipsoid ovary (#65, 0), and shallow leaf lobes (quotient, i.e., distance from the leaf outline to the sinus margin/distance from the leaf outline to the leaf base, commonly 0.05–0.15)(#34, 1). *Passiflora macfadyenii* bears flowers in pairs at the nodes as well as in inflorescences present as condensed terminal shoots with aborted laminas (#40, 1). It is also distinguished by its fused sepals (#48, 1), outer coronal filaments that are adnate to the sepals (#51, 1), inner coronal filaments that, when present, are very short (< 1 mm) (#11, 3), an ovary that is conspicuously and densely pubescent with long hairs (#67, 2), very long fusiform fruits (> 25 mm)(#24, 1), seeds that are at least twice as long as they are wide (#28, 2), long central leaf veins (commonly 31–41 mm)(#29, 2), and a central leaf lobe that is narrowed at the base (#86, 2). *Passiflora tridactylites* is positioned as more closely related to *Passiflora lancifolia* and *Passiflora macfadyenii* than it is to other taxa that comprise the *Passiflora suberosa* complex *s. l.* *Passiflora lancifolia*, *Passiflora macfadyenii*, and *Passiflora tridactylites* possess staminal filaments that are commonly less than half the length of the androgynophore (#19, 2), long pedicels (commonly 16–40 mm)(#1, 1), fusiform ovaries (with a reversal to ellipsoid ovaries in *Passiflora lancifolia*)(#65, 2), and a leaf base that is not cordate (#87, 1). *Passiflora tridactylites* is defined by its very long inner coronal filaments (commonly 3–4 mm)(#11, 1) and seed with the micropylar end and chalazal beak erect and not inclined toward the raphe (#95, 1). Relatively short staminal filaments (<3.5 mm)(#13, 2) unite *Passiflora tenuiloba*, *Passiflora pallida*, and Passiflora suberosa subsp. litoralis. *Passiflora pallida* and *Passiflora tenuiloba* are placed as sister species and have very narrow floral nectaries (commonly <0.5 mm in diameter)(#17, 0). *Passiflora pallida* is distinctive in having the narrowest hypanthium in the supersection (commonly 3.34–4.00 mm)(#4, 3), very short inner coronal filaments (commonly <1.5 mm)(#11, 3) and capitate to obconical petiolar nectaries (#80, 0/1). *Passiflora tenuiloba* is defined by its very short central leaf vein as compared to its leaf width (quotient, i.e., central vein length/leaf width, commonly ranging from 0.64–0.22)(#32, 3), very wide angle between the lateral leaf veins (145–343 degrees)(#33, 3) and often very long lateral lobes as compared to the central lobe (quotient, i.e., lateral vein length/central vein length, commonly ranging from 3.35–7.75)(#31, 3). In addition, this species has between 35 and 50 coronal filaments in its outer and inner coronal rows (#7, 1/2; #10, 1), a floral nectary floor that is sulcate (#71, 1), seeds that are between 4 and 5 mm long (#26, 1) and narrower than they are long (quotient, i.e., seed length/seed width, commonly ranging from 1.94–2.93)(#28, 2), and coriaceous leaves (#81, 2) with a short central vein (commonly 5.74–18.50 mm)(#29, 3). Passiflora suberosa subsp. litoralis commonly possesses three leaf lobes (only very rarely having unlobed or bilobed leaves)(#84, 4).

The remaining members of the supersection (*Passiflora clypeophylla*, *Passiflora juliana*, *Passiflora viridiflora*, *Passiflora megacoriacea*, *Passiflora mcvaughiana*, *Passiflora xiikzodz*, *Passiflora itzensis*, *Passiflora sexocellata*, *Passiflora coriacea*, *Passiflora tacanensis*, *Passiflora obtusifolia*, *Passiflora eglandulosa*, and *Passiflora trinifolia*) are defined by their widely diverging lateral lobes (the angle between the lateral leaf veins is between 115 and 165 degrees) (#33, 2), central leaf veins that are between half to three quarters the width of the leaves (#32, 1), and discoid petiolar nectaries (#80, 3). *Passiflora eglandulosa* and *Passiflora trinifolia* are placed as sister species and possess wide foliose stipules (#39, 1; #90, 2) and seeds with the micropylar end and chalazal beak erect and not inclined toward the raphe (#95, 1). *Passiflora trinifolia* is unique in having a very reduced limen (0.13–0.14 mm in height)(#22, 3) that is greenish yellow with reddish purple spots and streaks (#74, 3) and leaves that are sclerophyllous (#81, 3). It is also distinguished by its short central (16–40 mm)(#29, 2) and lateral (14–31 mm)(#30, 1) leaf veins. The flowers of *Passiflora trinifolia* possess a short floral stipe (1.8–3.4 mm)(#2, 0), 35–40 outer (#7, 2) and 38–47 inner (#10, 1) coronal filaments, an androgynophore with a flush of red/purple pigmentation toward the base (#59, 1), and an erect limen (#73, 1). *Passiflora eglandulosa* possesses flowers with long pedicels (commonly 11.35–18.00 mm)(#1, 1), spurs that occur between each of the sepals (#43, 1), very short inner coronal filaments (0.66–1.50 mm)(#11, 3), narrow limen floors (1.57–2.13 mm)(#23, 1), short staminal filaments (2.13–3.67 mm)(#13, 2), ellipsoid ovaries (#65, 0), and anthers that present pollen laterally (#63, 1). Its seeds are greater than 5 mm in length (#26, 0/1) and 3 mm in width (#27, 1) with weakly developed chalazal beaks (#96, 1). Lastly, as its name implies, *Passiflora eglandulosa* lacks laminar (#89, 6), and petiolar nectaries (#36, 3).

*Passiflora clypeophylla* is sister to the remaining species in the supersection (*Passiflora juliana*, *Passiflora viridiflora*, *Passiflora megacoriacea*, *Passiflora mcvaughiana*, *Passiflora xiikzodz*, *Passiflora itzensis*, *Passiflora sexocellata*, *Passiflora coriacea*, *Passiflora tacanensis*, and *Passiflora obtusifolia*). All of the species in this larger terminal clade have coriaceous leaves (#81, 2) commonly peltate (#82, 1; #83, 2), rarely cordate (#87, 1) and possess laminar nectaries (#37, 1). *Passiflora clypeophylla* has flowers with long pedicels (16.8–17.3 mm)(#1, 1) and stipes (9.4–14.3 mm)(#2, 2), coronal filaments with flushes of red/purple pigmentation towards their bases (#53, 2; #56, 2) and outer coronal filaments that are moderately long (5.0–5.5 mm)(#8, 3). In addition, *Passiflora clypeophylla* has some of the shallowest leaf lobes in the supersection (quotient, i.e., distance from the leaf outline to the sinus margin/distance from the leaf outline to the leaf base, commonly 0.03–0.07)(#34, 1).

*Passiflora obtusifolia* is positioned as the sister of the remaining species in the supersection (*Passiflora juliana*, *Passiflora viridiflora*, *Passiflora megacoriacea*, *Passiflora mcvaughiana*, *Passiflora xiikzodz*, *Passiflora itzensis*, *Passiflora sexocellata*, *Passiflora coriacea*, and *Passiflora tacanensis*). All of the species in this group possess conspicuous inflorescences present as condensed terminal shoots with aborted laminas (#40, 1) and an average of over 40 filaments in their inner coronal rows (#10, 1). *Passiflora obtusifolia* possesses short sepals (<7 mm)(#5, 2), inner coronal filaments that are nearly equal in length to the outer coronal filaments (quotient, i.e., length of filaments in the outer row/length of filaments in the inner row, commonly ranging from 0.67–0.87)(#12, 2), outer coronal filaments that are slightly capitate (#54, 1), and petiolar nectaries that are positioned on the upper half of the petiole (#36, 1).

The remaining taxa in this analysis occur in two major clades. With the exception of two taxa (*Passiflora megacoriacea* and *Passiflora obtusifolia*) all of the species that have been separated out of the *Passiflora coriacea* complex (*Passiflora tacanensis*, *Passiflora mcvaughiana*, *Passiflora itzensis*, *Passiflora xiikzodz*, *Passiflora sexocellata*, and *Passiflora coriacea* *s. s.*) form one major clade. The second major clade consists of *Passiflora juliana* and *Passiflora viridiflora*, along with *Passiflora megacoriacea*. These two major groups form one larger clade that is delimited by the following synapomorphies: more than 40 filaments in the outer coronal row (#7, 1), ellipsoid ovaries (#65, 0), seeds that are more than 3.8 mm in length (#26, 1), and the presence of more than four laminar nectaries (#37, 0).

Positioned basally to *Passiflora viridiflora* and *Passiflora juliana* is *Passiflora megacoriacea*. All three species possess tall limens (commonly 0.75–1.14)(#22, 1), long opercula (commonly 2.0–4.0 mm)(#20, 2), and long ovaries (> 2.5 mm)(#16, 0). *Passiflora juliana* and *Passiflora viridiflora* are sister species in this analysis, and aside from adaptations in *Passiflora viridiflora* resulting from a shift in pollinators, these two species with greenish yellow flowers from the Pacific coast and coastal plain of southwestern Mexico are very similar vegetatively with leaves that have a central lobe which is distinctly narrowed at the base (#86, 2). *Passiflora juliana* possesses a very short floral stipe (the shortest in the supersection)(#2, 0), a limen floor that is distinctly purple (#75, 2) and an androgynophore flushed with purple at the base to just above the middle (#59, 2), moderately long outer coronal filaments (commonly 3.13–3.69 mm)(#8, 2/3), and large (9.44–14.81 mm long and 3.81–9.31 mm wide)(#38, 2; #39, 1) foliose stipules (#90, 2). *Passiflora viridiflora* differs from *Passiflora juliana* in its adaptations for hummingbird pollination: a greatly elongated androgynophore (17.40–26.10 mm)(#18, 3) that far exceeds the length of the stamen filaments (quotient, i.e., stamen filament length/androgynophore length, commonly ranging from 0.16–0.20)(#19, 3), no inner coronal filaments (#10, 6; #11, 4; #12; 3; #50, 2), very narrow limen floor (commonly 0.63–2.07 mm)(#23, 1), wide floral nectary (commonly 1.30–1.82 mm)(#17, 2), long operculum (3.00–4.60 mm)(#20, 1) that is not incurved at the margin but erect and lies against the androgynophore, fused sepals (#48, 1) that are greatly elongated (16.88–32.00 mm long and ca. 7.5 times longer than they are wide)(#5, 3; #6, 2) and much longer than the outer coronal filaments (quotient, i.e., length of filaments in the outer row/sepal length, commonly ranging from 0.09–0.21)(#9, 3), outer coronal filaments that are connate (#55, 1) and adnate to the sepals (#51, 1), pollen that is presented laterally (#63, 1), a sulcate floral nectary floor (#71, 1), and a limen that is not recurved but inclined toward the operculum (#73, 1). *Passiflora viridiflora* also possesses fruits that are widely ovoid with a conical tip (#76, 4). *Passiflora megacoriacea* is distinctive based upon its wide hypanthium (commonly 8.9–12.5 mm)(#4, 0/1) and limen floor (commonly 5.0–7.0 mm)(#23, 2), elongated androgynophore (commonly 7.3–8.0 mm long)(#18, 0), staminal filaments that are generally less than half the length of the androgynophore (#19, 2), 30 to 40 outer coronal filaments (#7, 2) that are between 7.7 and 10.3 mm in length (#8, 1) and somewhat dilated toward the apex (#54, 1), floral nectary with a raised annulus (#70, 1), petiolar nectaries that are most commonly on the upper half of the petiole (#36, 1), and seeds that are about 5.0 mm long (#26, 0) and 3.0 mm wide (#27, 1).

The clade containing most of the species that have, in the past, been separated out of *Passiflora coriacea* possess the following synapomorphies: transversely elliptic leaves (#85, 2) with short central leaf veins as compared to the leaf widths (quotient, i.e., central vein length/leaf width, commonly ranging from 0.19–0.44)(#32, 2), very shallow leaf lobes (quotient, i.e., distance from the leaf outline to the sinus margin/distance from the leaf outline to the leaf base, commonly ranging from 0.02–0.20)(#34, 1), and lateral veins that are much longer than the central veins (quotient, i.e., lateral vein length/central vein length, commonly ranging from 1.0–3.0)(#31, 2). *Passiflora tacanensis*, known from only one fruiting and two sterile herbarium specimens, is found on Volcán Tacaná in a high mesophytic forest in southern Mexico. It has no laminar nectaries (#37, 2), wide seeds (2.8–3.1 mm)(#27, 1), foliose stipules (#90, 2) that are 6.3–7.5 mm long (#38, 2) and 2.5–3.5 mm wide (#39, 1), nonpeltate leaves (#83, 0) and seeds with a chalazal beak that is not well-developed (#96, 1). *Passiflora coriacea*, occurring in tropical moist forests of Colombia, Ecuador, Peru, Bolivia, and Venezuela, is the sister taxon to a clade containing *Passiflora sexocellata*, *Passiflora mcvaughiana*, *Passiflora itzensis*, and *Passiflora xiikzodz*. These five species commonly possess bilobed leaves (#84, 2) with central veins that are frequently between 14.1 and 45.6 mm (from the point of petiole insertion to the central lobe apex)(#29, 2). *Passiflora coriacea* has a long stipe as compared to its pedicel (quotient, i.e., stipe length/pedicel length, ranging from 0.77–2.85)(#3, 1), seeds that are between 3.6 and 3.8 mm long (#26, 2), and a dark reddish purple operculum (#69, 2). *Passiflora sexocellata*, a species found primarily in low, moist forests from eastern Mexico to Nicaragua, is sister to *Passiflora xiikzodz*, *Passiflora itzensis*, and *Passiflora mcvaughiana*. These four species possess outer coronal filaments that are commonly greater than 5 mm in length (#8, 2) and limens that are absent (*Passiflora xiikzodz* and *Passiflora itzensis*) or not recurved (*Passiflora mcvaughiana* and *Passiflora sexocellata*)(#73, 1). *Passiflora sexocellata* possesses staminal filaments that are commonly half the length of the androgynophore (quotient, i.e., filament length/androgynophore length, ranging from 0.41–0.71)(#19, 2), between 29 and 30 inner coronal filaments (#10, 2), and a greenish yellow operculum with a flush of dark red at the base (#69, 1). *Passiflora mcvaughiana*, a species occurring in high pine-oak forests of southwestern Mexico, is sister to *Passiflora xiikzodz* and *Passiflora itzensis*. These three species possess fruits that are between 10 and 15 mm wide (#25, 2), seeds that are generally greater than 5 mm in length (#26, 0), and petiolar nectaries that are positioned on the upper half of the petiole (#36, 1). *Passiflora mcvaughiana* is distinctive because it possesses styles that are pale greenish yellow with reddish purple spots and streaks (#60, 2). It commonly lacks inflorescences (#40, 0), has between 31 and 36 outer coronal filaments (#7, 2) that are 3.5–7.1 mm long (#8, 3), anthers that possess reddish purple margins (#62, 0), the widest seeds in the subsection (at least 4 mm in width)(#27, 0), and lacks laminar nectaries (#37, 2; #89, 6). The taxa treated as *Passiflora xiikzodz* and *Passiflora itzensis* in this revision are sister species that constitute a well-supported clade in this analysis (100% bootstrap value). These two taxa are commonly found in tropical deciduous forests on limestone outcrops in southeastern Mexico, Guatemala, and Belize. They were treated at the subspecific level in MacDougal (1992) and are undoubtedly very closely related. However, the recognition of these taxa at the specific level seems justified based upon the large number of autapomorphies for *Passiflora itzensis*. Additionally, cross-pollinations performed by MacDougal (1992) in the greenhouse between these entities proved unsuccessful. *Passiflora xiikzodz* and *Passiflora itzensis* are sister species based upon their dark red coronal filaments (#53, 4) occurring in seven series (#50, 3), lack of floral nectaries (#17, 5; #21, 3), lack of limens (#22, 4; 72, 1), denticulate (#68, 1), dark reddish purple (#69, 3) opercula that are between 0.25 and 0.72 mm in length (#20, 5), second coronal rows with filaments that are not capitate or capitellate (#57, 0), and long stipes as compared to their pedicels (quotient, i.e., stipe length/pedicel length, ranging from 2.3–12.4)(#3, 0). *Passiflora itzensis* has the following apomorphies: the lack of or presence of a greatly reduced dark reddish purple androgynophore (0–1.7 mm)(#18, 2; #59, 3), fewer filaments in its outer (22–31)(#7, 3) and second (20–30)(#10, 2) coronal rows, short styles (0.81–1.78 mm)(#15, 2), androecium and gynoecium with red pigmentation (#60, 4; #61, 3), and distal presentation of pollen (#63, 2). *Passiflora xiikzodz* possesses the apomorphies of long outer coronal filaments (commonly 7.63–9.28 mm)(#8, 1) and a wide limen floor (commonly 5.20–6.13 mm)(#23, 2).

### Discussion of all cladistic and phenetic analyses

The morphological and molecular analyses presented here confirm the monophyly of Passiflora supersection Cieca. In addition, both phenetic and cladistic analyses have increased our understanding of some of the complex biological issues influencing the evolution of the group.

Congruence between phylogenetic hypotheses generated from independent data sets, when subjected to reliable methods of phylogenetic analysis, is often thought to be evidence for considering those hypotheses as representative of the “true” phylogeny. Conflict may indicate theoretical or procedural problems in one or both of the analyses, or that additional data are needed to resolve the phylogenetic relationships in question (Hillis 1987). There is a considerable amount of incongruence between the molecular and morphological phylogenies for Passiflora supersection Cieca presented in previous chapters. Sample size has likely had an influence on this incongruity. For the morphological data set, specimens for all the species of the supersection from throughout their geographic ranges were carefully measured and examined in order to determine the extent of variation of the characters. However, such a large sample size in the molecular study was not feasible due to limited sample availability and expense of analysis. There were also several polymorphic sites evident in the ITS sequences for several of the species within supersection *Cieca*, further increasing the need for more infraspecific sampling. Most importantly, the complex evolutionary history (involving hybridization and polyploidy) of several of the entities within the group has undoubtedly had a significant effect on both data sets, increasing the amount of incongruity in the analyses.

In an attempt to overcome the difference in sample sizes between the morphological and molecular data sets, additional cladistic analyses were undertaken in which the operational taxonomic units were reduced to those for which both molecular and morphological data were available. Separate analyses of the reduced morphological and molecular data sets resulted in the production of four equally parsimonious trees that were 224 steps long and 1,638 equally parsimonious trees that were 332 steps in length, respectively. The consensus trees from both of these analyses were compared and were conspicuously incongruent in their topologies. In addition, there were significant changes in the resulting phylogenies, as compared to the original analyses, that were likely due to decreased (and likely inadequate) taxon sampling. I have chosen not to combine my morphological and molecular data sets because of this incongruence and the knowledge that complex biological processes are likely influencing the pattern of diversity within Passiflora supersection Cieca.

On a more positive note, the phylogenetic hypotheses based on the morphological and molecular data sets for supersection *Cieca* agree in several respects. In the molecular analysis, there is evidence for the monophyly of *Passiflora pallida* (95% bootstrap), *Passiflora lancifolia* (87% bootstrap), *Passiflora tenuiloba* (100% bootstrap), *Passiflora sexocellata* (93% bootstrap), *Passiflora viridiflora* (96% bootstrap), *Passiflora juliana* (100% bootstrap), *Passiflora obtusifolia* (50% bootstrap), and *Passiflora mcvaughiana* (84% bootstrap) (Figs 9–12). In the morphological analysis, each of these species is also diagnosable by unique combinations of character states (Figs 20–21). The molecular and morphological analyses also agree in their support for the monophyly of a clade containing *Passiflora itzensis* and *Passiflora xiikzodz* and a clade composed of *Passiflora juliana* and *Passiflora viridiflora*.

The sister-group relationship between *Passiflora xiikzodz* and *Passiflora itzensis* is highly supported in both the molecular (90%) and morphological (100%) trees. In the morphological analysis *Passiflora xiikzodz* and *Passiflora itzensis* are diagnosable by a number of morphological characters. In addition, crossing studies by MacDougal (1992) suggest that these species are unable to interbreed, whereas fruits with viable seeds were easily produced between two clones of *Passiflora xiikzodz* (*MacDougal 4690* and *MacDougal 4677*) (C. Feuillet, pers. comm.). However, though the two species are separated from one another in the strict consensus tree in the molecular analysis, there is no bootstrap support for either of the species-level clades. Despite the lack of statistical support for *Passiflora itzensis* and *Passiflora xiikzodz* as cladospecies in the molecular analysis, I have elevated Passiflora xiikzodz subsp. itzensis and Passiflora xiikzodz subsp. xiikzodz to the rank of species based on their consistent differences in floral morphology. The way in which *Passiflora itzensis* displays its pollen is dramatically different from that of *Passiflora xiikzodz*, indicating a shift in pollinators. Thus, *Passiflora xiikzodz* and *Passiflora itzensis* are considered sibling species, which do not appear to be able to interbreed and possess consistent and easily observed diagnostic morphological characters.

Both the molecular and morphological data support the monophyly of a clade containing *Passiflora juliana* and *Passiflora viridiflora*; each is considered a cladospecies, and together they compose a moderately supported (77%) clade in the molecular analysis. In his original description of *Passiflora juliana*, MacDougal (1992) discussed its similarities with *Passiflora viridiflora*, and both species are found in similar habitats along the Pacific coast and in the Pacific coastal plain of southwestern Mexico. The primary differences between these two species are the shape of the stipules and several changes in floral and vegetative morphology associated with a shift in pollinators (hymenopteran to hummingbird pollination). Again, each of these two species is well-supported with bootstrap values greater than 95% in the molecular analysis and are morphologically diagnosable cladospecies.

The phylogeny based on the molecular data is different from that based on the morphological data in many ways (compare Figs 9–12 and 20–21). *Passiflora mcvaughiana* is most closely related to *Passiflora juliana* and *Passiflora viridiflora* in the DNA-based trees. In the morphological analysis, *Passiflora mcvaughiana* is in a clade with *Passiflora itzensis*, *Passiflora xiikzodz*, *Passiflora sexocellata*, *Passiflora coriacea*, and *Passiflora tacanensis* because they all possess bilobed or shallowly trilobed, transversely elliptic leaves and very similar flowers, especially in the case of *Passiflora coriacea* and *Passiflora sexocellata*. *Passiflora obtusifolia* is sister to the clade containing *Passiflora itzensis*, *Passiflora xiikzodz*, *Passiflora mcvaughiana*, *Passiflora juliana*, and *Passiflora viridiflora* in the molecular cladogram. However, in the morphological analysis, *Passiflora obtusifolia* is sister to all of the species listed above (*Passiflora itzensis*, *Passiflora xiikzodz*, *Passiflora mcvaughiana*, *Passiflora juliana*, and *Passiflora viridiflora*) plus *Passiflora megacoriacea*, *Passiflora sexocellata*, *Passiflora coriacea*, and *Passiflora tacanensis*. *Passiflora coriacea*, *Passiflora sexocellata*, and *Passiflora tenuiloba* form a clade in the molecular analysis. In the morphological analysis, *Passiflora tenuiloba* is most closely related to *Passiflora pallida*, a species that also occurs in southwestern Texas and northeastern Mexico. In the molecular analysis, *Passiflora coriacea* is sister to *Passiflora sexocellata* and they form a monophyletic group. In the morphological analysis *Passiflora coriacea* is also sister to *Passiflora sexocellata*, but they are in a clade with *Passiflora mcvaughiana*, *Passiflora xiikzodz*, and *Passiflora itzensis*. Based upon the molecular data, *Passiflora lancifolia* is sister to Passiflora suberosa subsp. suberosa but is more closely related to *Passiflora macfadyenii*, *Passiflora pallida*, and Passiflora suberosa subsp. litoralis in the morphological analysis. In the molecular analysis *Passiflora pallida* is sister to all of the members of the supersection, whereas Passiflora suberosa subsp. suberosa is positioned as sister to the other taxa in the morphological analysis (see Figs 9–12 and 20–21).

Clearly, additional molecular data from more variable gene regions are needed to help resolve phylogenetic relationships in Passiflora supersection Cieca. Several gene regions were sequenced in an attempt to attain independent sets of molecular information, but none proved variable enough to resolve the phylogeny (*trn*L-*trn*F, cytosolic-expressed glutamine synthetase, *G3pdh*, *psb*A-*trn*H). The only region that proved promising was *waxy* (granule-bound starch synthase), but up to seven copies of the gene are found within diploid individuals from the supersection and my preliminary results (not shown) could not be interpreted without extensive and additional sampling.

*The* Passiflora coriacea *Complex.* Jussieu described *Passiflora coriacea* in 1805 from a specimen collected in Colombia by Bonpland. Shortly afterward several authors (e.g., Smith 1814 and Kunth 1817) described additional species based upon characters of the leaves (*Passiflora clypeata* Sm. and *Passiflora difformis* Kunth). However, their descriptions seem to give only an account of the vegetative variation within different populations of *Passiflora coriacea* Juss. in Colombia. Schlechtendal (1854) described *Passiflora sexocellata* and differentiated it from both *Passiflora coriacea* and *Passiflora difformis* based primarily upon the vegetative morphology of the species (because the flowers of *Passiflora coriacea* were largely unknown), but his careful description of the flowers of his new species differed markedly from those in Kunth’s brief description of *Passiflora difformis* (for further information please see discussion of *Passiflora sexocellata* below). In his revision of the American species of Passifloraceae, Killip (1938) listed all of the species discussed above plus several others (*Passiflora obtusifolia* and *Passiflora cheiroptera* Cortés) in synonomy under *Passiflora coriacea*. However, since that revision, MacDougal (1992, 2001) described two new species, *Passiflora xiikzodz* and *Passiflora mcvaughiana*, and resurrected one previously described species, *Passiflora obtusifolia*, that were reported from southwestern Mexico under the name *Passiflora coriacea*. In addition, both the phenetic and cladistic analyses based upon both molecular and morphological data presented here support the recognition of three distinct taxa: *Passiflora coriacea*, *Passiflora sexocellata*, and *Passiflora megacoriacea*.

The multivariate statistical analysis of the quantitative morphological characters for *Passiflora coriacea* *s. l.* produced a plotting pattern that clearly supports the delimitation of the previously undescribed species *Passiflora megacoriacea*, but it is not until an analysis of the floral characters alone was undertaken that *Passiflora coriacea* and *Passiflora sexocellata* became phenetically separable. Neighbor joining analyses of both the qualitative and quantitative morphological characters also support the recognition of these three taxa, with only two accessions representing *Passiflora sexocellata* (from Belize and Mexico) appearing more similar to *Passiflora megacoriacea* than to other members of *Passiflora sexocellata* from Mexico and Central America. The morphological cladistic analysis of the supersection suggests that *Passiflora sexocellata* and *Passiflora coriacea* evolved from a common ancestor, but that *Passiflora megacoriacea* is more closely related to *Passiflora juliana* and *Passiflora viridiflora*. *Passiflora megacoriacea* is placed with these species primarily based upon characters relating to an increase in flower size (ovary size, operculum length, etc.) that might be attributed to independent shifts to larger pollinators. However, vegetative and reproductive characters other than those relating to overall flower size (e.g., the shape of the flowers with an erect outer corona that is bent toward the androgynophore, leaf shape, etc.) in *Passiflora megacoriacea* suggest a closer relationship with *Passiflora coriacea* and *Passiflora sexocellata*. Unfortunately, I was unable to obtain material of *Passiflora megacoriacea* for DNA sequencing, and a molecular analysis that includes *Passiflora megacoriacea* is needed and will likely settle the issue. Nevertheless, it is clear in the molecular and morphological cladistic analyses that *Passiflora coriacea* and *Passiflora sexocellata* are sister to each other (with a bootstrap value of 100% in the DNA-based tree) and are both clearly diagnosable cladospecies, and it is also likely that *Passiflora megacoriacea* is closely related to these two taxa.

*The* Passiflora suberosa *Complex.* The oldest herbarium specimens that I have seen of any of the members of the *Passiflora suberosa* complex were collected in the 1700s, and since that time plant collectors have deposited thousands of specimens of *Passiflora suberosa* *s. l.* (3,244 of which I annotated) in herbaria around the world. Linnaeus (1745, 1753) originally described four species that have, over the years, been considered to be part of (at specific and/or subspecific levels) the *Passiflora suberosa* complex. Since that time, various systematists engaged in revising the genus and family have described new species (e.g., Cavanilles 1790; Hooker 1867), varieties and subvarieties (e.g., Roemer 1846; Masters 1872) or lumped various entities under one species name (Killip 1938). In his revision of the American species of Passifloraceae, Killip (1938) recognized *Passiflora suberosa* in the widest sense and considered the various species, subspecies, and varieties falling within this species to be too intergrading and indistinct to merit taxonomic recognition. He concluded that *Passiflora suberosa* was an extremely variable species and that no constant characters permitted the maintenance of the proposed variants as distinct taxa. Since Killip’s revision, the variability in gross morphological characters, as seen in herbarium specimens of *Passiflora suberosa* *s. l.*, the over-reliance of many authors on the vegetative morphology in sorting out entities within the “species,” and the sheer task involved in sorting through the thousands of specimens collected from around the world, has helped to perpetuate his broad concept of this species. However, both the phenetic and cladistic analyses based upon both molecular and morphological data presented here support the non-monophyly of *Passiflora suberosa* *s. l.* and the recognition of four distinct taxa within this complex: Passiflora suberosa subsp. suberosa, Passiflora suberosa subsp. litoralis, *Passiflora pallida*, and *Passiflora tridactylites*.

In the multivariate analyses of the quantitative morphological characters for *Passiflora suberosa* *s. l.*, little correlation was found between floral and vegetative characters. Instead, the variability in many of the vegetative characters in the complex made it difficult to elucidate distinct taxa. However, an analysis of the floral characters alone produced an ordination pattern that supports the delimitation of *Passiflora pallida*, Passiflora suberosa subsp. suberosa, Passiflora suberosa subsp. litoralis, and *Passiflora tridactylites*, though some overlap among entities of Passiflora suberosa subsp. litoralis and *Passiflora pallida* does exist. Neighbor joining analyses of both qualitative and quantitative morphological characters also support the recognition of four taxa, but accessions of *Passiflora pallida* were intermixed with accessions of Passiflora suberosa subsp. litoralis, further indicating that the differences between these two taxa are sometimes difficult to discern. The results of the neighbor joining analysis also suggest that both *Passiflora tridactylites* and Passiflora suberosa subsp. suberosa may have evolved from Passiflora suberosa subsp. litoralis. The morphological cladistic analysis of the supersection as a whole indicates the converse, that is, Passiflora suberosa subsp. suberosa is sister to the rest of the species in the supersection. The position of Passiflora suberosa subsp. suberosa in the cladistic analysis is questionable. Nevertheless, it is placed there because it shares a number of characters (e.g., sepal color) with the chosen outgroups which are probably actually derived within the supersection. Passiflora suberosa subsp. litoralis, *Passiflora pallida*, and *Passiflora tridactylites* are in a clade with *Passiflora tenuiloba*, *Passiflora lancifolia*, and *Passiflora macfadyenii* in the morphological analysis. *Passiflora pallida* and Passiflora suberosa subsp. litoralis are present in a clade with *Passiflora tenuiloba*, underscoring the close relationship between *Passiflora pallida* and Passiflora suberosa subsp. suberosa, with *Passiflora tenuiloba* forming a clade with *Passiflora pallida* based upon the width of the floral nectary. *Passiflora tridactylites* is placed in a clade with *Passiflora lancifolia* and *Passiflora macfadyenii*, which is likely a consequence of the increased flower size and other adaptations to non-hymenopteran pollinators in these three species. The molecular analysis indicates that *Passiflora lancifolia* is sister to Passiflora suberosa subsp. suberosa, though with only moderate support (73%), and according to my morphological analysis, *Passiflora macfadyenii* is sister to *Passiflora lancifolia*. In addition, *Passiflora tridactylites* is very similar in many morphological characters to Passiflora suberosa subsp. litoralis, both of which occur on islands of the Galapagos, underscoring their probable close relationship. *Passiflora tridactylites*, *Passiflora lancifolia*, and *Passiflora macfadyenii* are similar in many aspects to *Passiflora suberosa*, and all three likely evolved from it. However, it is doubtful that they are each other’s closest relatives.

In the molecular cladistic analysis, some of the ambiguities apparent in the results from the phenetic analyses and morphology-based cladistic analyses were resolved or at least clarified. In all three trees resulting from an analysis of the ITS sequence data of supersection *Cieca*, *Passiflora pallida* appeared to be monophyletic with bootstrap support of 95%, but Passiflora suberosa subsp. suberosa and Passiflora suberosa subsp. litoralis are not indicated as monophyletic. Their non-monophyly is likely due, at least in part, to some amount of gene exchange between these entities. While the amplification of the ITS region yielded a single product for all accessions of the supersection as revealed by gel electrophoresis, I noticed that the directly sequenced PCR product for several accessions of Passiflora suberosa subsp. suberosa, Passiflora suberosa subsp. litoralis and what morphologically appeared to be *Passiflora pallida* contained polymorphic sites (where two discernible peaks of approximately equal strength appeared in the chromatograms). Thus, I began to clone several of my PCR products and found that the accessions with polymorphic sites often proved to possess differing, apparently functional copies of ITS; there were no significant nucleotide substitutions, insertion-deletion events, or substitutions (particularly in conserved regions) apparent in the sequences that would indicate that the copies were nonfunctional. Four individuals of supersection *Cieca* contained polymorphic sites (two accessions of Passiflora suberosa subsp. litoralis from Puebla and Veracruz, Mexico, one accession of Passiflora suberosa subsp. suberosa from Haiti, and one accession from Florida, USA that had small flowers similar to *Passiflora pallida*), while the rest of the species did not contain any polymorphisms in the ITS region. The cloned sequences of Passiflora suberosa subsp. suberosa from Haiti were placed in two different clades, with two clones falling within a moderately supported clade containing other members of the subspecies from the Caribbean and the other two forming a group in the strict consensus tree, which is positioned sister to Passiflora suberosa subsp. litoralis. In addition, cloned entities of Passiflora suberosa subsp. litoralis from the states of Puebla and Veracruz, Mexico are found in separate clades. The clones of the accession that morphologically fits the description of *Passiflora pallida* [“*sub.* w/ *pall.* aff. USA (FL)”, see Fig. 13] from the United States occur in both the well-supported *Passiflora pallida* clade and the clade containing *Passiflora suberosa* along with the rest of the species from the supersection, indicating that there is gene flow, likely resulting from hybridization, between *Passiflora suberosa* and *Passiflora pallida*. This gene flow has likely obscured the distinctiveness of *Passiflora pallida* and contributed to the broad circumscription of *Passiflora suberosa*.

As shown in the phenetic analyses of *Passiflora suberosa* *s. l.*, there is clearly some overlap in the morphological characters of *Passiflora pallida* and *Passiflora suberosa*. This may indicate that there is limited gene flow occurring between these species, and the molecular data are consistent with this hypothesis. However, most specimens of *Passiflora suberosa* and *Passiflora pallida* are clearly separable and the inclusion of *Passiflora pallida*, a well-supported cladospecies, within the circumscription of *Passiflora suberosa* would render this species extremely non-monophyletic and obscure the distinctiveness of an early divergent lineage within the supersection. According to the molecular data there definitely seems to be gene flow between the subspecies of *Passiflora suberosa*, and though they are morphologically distinct, I felt it best to treat these two somewhat geographically isolated taxa at the subspecific level (as opposed to the species level). The exact impact that hybridization and polyploidy are having on the evolution of *Passiflora pallida* and *Passiflora suberosa* remains unknown, but it is clear that these processes have blurred the distinctions between these species and made the sorting out of phylogenetic relationships within these widespread and variable taxa extremely difficult. However, population level studies incorporating cytological data and DNA fingerprinting likely would reveal their consequences, clarifying the circumscription of *Passiflora suberosa* and its subspecies.

Phenetic and cladistic analyses of the supersection based on morphological and molecular characters were utilized to generate hypotheses of species phylogenetic relationships and redefine specific entities, especially within the two species complexes. The phylogenetic analyses presented here confirm the monophyly of the supersection. In the molecular and morphological analyses each of the species of the supersection, with the exception of *Passiflora suberosa*, is monophyletic and diagnosible by a unique combination of character states. There is support in the molecular and morphological analyses for the monophyly of a clade containing *Passiflora itzensis* and *Passiflora xiikzodz* and a clade composed of *Passiflora juliana* and *Passiflora viridiflora*. In addition, there is strong support in the molecular analysis for a clade comprising *Passiflora coriacea* and *Passiflora sexocellata*.

Four taxa that were formerly included in the *Passiflora suberosa* complex are recognized here: *Passiflora pallida*, Passiflora suberosa subsp. suberosa, Passiflora suberosa subsp. litoralis, and *Passiflora tridactylites*. Both the molecular and morphological analyses show that *Passiflora suberosa* is not monophyletic, a situation that may be quite common in plants, suggesting that a criterion of monophyly for species recognition may be inappropriate. It is quite possible that *Passiflora suberosa* has been caught in the paraphyletic “stage” of speciation, and the data indicate that it might be more logical to view the phylogenetic status of a species as a property that may change over time. Peripherial isolate speciation, such as that which presumably has given rise to the Galapagos endemic, *Passiflora tridactylites*. *Passiflora tridactylites* possesses a flower that appears to be adapted to a larger pollinator than its relative *Passiflora suberosa*, which is mainly pollinated by hymenopterans, and *Passiflora tridactylites* may be moth pollinated. The analyses also indicate that there is limited gene flow, likely in the form of hybridization, occurring between *Passiflora suberosa* and *Passiflora pallida*. This gene flow has obscured the distinctiveness of *Passiflora pallida*, a species that is likely sister to the remaining members of the supersection, and has contributed to the traditional broad circumscription of *Passiflora suberosa*. *Passiflora pallida* and *Passiflora suberosa* have also been shown to be polyploids (including triploid, tetraploid and hexaploid counts). The exact impact that hybridization and polyploidy are having on the evolution of *Passiflora pallida* and *Passiflora suberosa* is unknown; however, it is clear that these processes have blurred the distinctiveness of these two species and made the sorting out of phylogenetic relationships between and within them very difficult.

Three species from the *Passiflora coriacea* complex are recognized: *Passiflora coriacea*, *Passiflora megacoriacea*, and *Passiflora sexocellata*. It is clear in the molecular analysis that *Passiflora coriacea* and *Passiflora sexocellata* are sister to each other, and both are clearly diagnosable. It is also likely that *Passiflora megacoriacea* is closely related to these two taxa. *Passiflora megacoriacea* and *Passiflora sexocellata* were recognized as specifically distinct as a result of this investigation.

*Passiflora xiikzodz* and *Passiflora itzensis* are recognized at the specific level, as opposed to the subspecific level, due to consistent differences in floral morphology which likely resulted from a shift in pollinators. Based upon floral morphology, the majority of the species in the supersection are probably pollinated by insects, likely hymenoptera. However, pollination by hummingbirds has also been reported for several species of the supersection and appears to have evolved at least twice, once in *Passiflora viridiflora* and again in the common ancestor of *Passiflora lancifolia* and *Passiflora macfadyenii*, leading to dramatic shifts in floral form in these species. The species of supersection, for the most part, are not sympatric and where two or more species coincide, they are found growing at different elevations or in different habitats. Several species within the supersection (e.g., *Passiflora lancifolia*, *Passiflora macfadyenii*, *Passiflora tridactylites*, and *Passiflora viridiflora*) fit the peripheral isolate model of speciation and have developed divergent ecological amplitudes that have allowed them to invade novel habitats and exploit different spectrums of pollinators.

Most of the species of Passiflora supersection Cieca are utilized by common and widespread species of the subfamily Heliconiinae. Many of the species in the supersection have only one or two known herbivores, but, as one would expect, the species that are widely distributed have a greater diversity of herbivores. The extent and nature of mutual descent between the species of supersection *Cieca* and the Heliconiinae, still remains largely unknown.

Lastly, the utilization of only one concept to define the species of supersection *Cieca* was inadequate. However, meaningful biological entities were identified through the integration of elements from several concepts (e.g., the biological, phenetic, autapomorphic and diagnostic species concepts), along with information from many new taxonomic collections, observations of living material, and detailed phenetic and phylogenetic analyses (based on DNA and/or morphological data).

## Taxonomic treatment

### Passiflora

Taxon classificationPlantaeMalpighialesPassifloraceae

L., Sp. Pl. 955. 1753.

Passiflora Lectotype species, designated by N. Britton and A. Brown, 1913, pg. 565: *Passiflora incarnata* L.

#### Description.

Herbaceous or woody, perennial (rarely annual or with annual shoots from perennial roots), tendril-climbing vines or lianas, rarely shrubs or small trees lacking tendrils; usually containing cyanogenic glycosides having a cyclopentenoid ring system; glabrous to densely pubescent with simple trichomes, rarely gland-headed. Stems terete to lobed or sharply angled, occasionally with anomalous secondary growth, the shoot apex erect to cernuous. Leaves alternate (very rarely subopposite to opposite), simple (rarely palmately compound), petiolate, often with variously shaped and positioned extrafloral nectary glands on the petiole; laminas unlobed or lobed, often heteroblastic, pinnately to often palmately (rarely pedately) veined, variegated or not, entire to serrate, peltate or not, often bearing small nectaries associated with marginal teeth or indentations, or abaxially submarginal, or abaxial between the major veins. Stipules setaceous or narrowly triangular to foliaceous, persistent or early deciduous, entire to serrate, sometimes the margins with glands, occasionally cleft. Tendrils axillary, simple (rarely compound), representing a modified flower stalk of the central part of the inflorescence, straight, curved, or circinate during development at the shoot apex, rarely with adhesive terminal disks. Inflorescences axillary, bracteate or rarely ebracteate, cymose, the central pedicel developed into a tendril, the peduncle very reduced or usually absent, the pedicels then arising collateral to the tendril (sometimes aborted), solitary or paired; secondary inflorescences may be present as condensed axillary or terminal shoots, determinate or rarely indeterminate; pedicels articulate distal to bracts, the distal portion called the floral stipe; bracts setaceous and scattered to foliaceous or pinnatifid and involucrate, occasionally glandular at margin. Flowers bisexual (sometimes functionally staminate), actinomorphic or rarely the reproductive parts zygomorphic; hypanthium ± flat to campanulate, occasionally the perianth basally connate/adnate into a floral tube; sepals 5 (very rarely 8), quincuncially imbricated (rarely non-overlapping) in the bud, occasionally carinate, sometimes with a subapical projection; petals 5 (very rarely 8) or sometimes wanting, quincuncially imbricated (rarely non-overlapping) in the bud, the same length as or shorter (rarely slightly longer) than the sepals; corona present at the base of the calyx or corolla or adnate to the inside of the floral tube, in 1 to many series of distinct to occasionally connate, short to elongate, often showy filaments or outgrowths, sometimes membranous, the innermost series, called the operculum, often connate at least basally, frequently membranous and shielding the nectary; the limen (extrastaminal nectariferous disk) present as a ring or cup around base of androgynophore (or rarely the ovary if androgynophore absent), or discoid or conical and adnate to the floor of hypanthium. Stamens 5(8 in one species), usually alternate with the petals, borne on an often elongate androgynophore or androgynophore rarely absent; filaments free just below ovary or rarely connate into a tube around ovary; anthers introrse in bud, moving to become extrorse (rarely latrorse) at anthesis, dorsifixed, versatile, dehiscing longitudinally, borne parallel or perpendicular to their filaments; pollen binucleate, 3- to 12-colporate. Carpels 3(-5), connate, ovary superior, unilocular, borne on an often elongate androgynophore (rarely sessile), placentation parietal, anatropous ovules numerous on each placenta; styles distinct, rarely connate near base; stigmas capitate, clavate, reniform, or occasionally bilobed. Fruit a few to many seeded berry, rarely a loculicidal or anomalously dehiscent capsule. Seeds arillate, usually flattened, the testa pitted, reticulate-foveate, or transversely grooved or sulcate; endosperm slightly ruminate, oily, abundant; embryo straight, the cotyledons usually elliptic to oblong-elliptic; germination epigeal (rarely hypogeal). Chromosome numbers: n = 6, 9, 10, 12 (rarely 7, 11, 18, 42).

### Passiflora subgenus Decaloba (DC.) Reichenbach supersection Cieca

Taxon classificationPlantaeMalpighialesPassifloraceae

(Medikus) J. M. MacDougal & Feuillet, Passiflora 13(2):37. 2003 [2004]

Cieca Medikus, Malvenfam. 97. 1787, non *Cieca* Adanson (Euphorbiaceae), 1763, nom. rej. Lectotype species, designated by E.P. Killip 1938, p. 25: *Cieca viridis* Medikus [*Passiflora pallida* L].
Passiflora sect. Cieca (Medikus) DC. Mém. Soc. Phys. Genève 1: 435. 1822. Type species: Based on *Cieca* Medikus.
Passiflora subgenus Decaloba sect. Cieca (Medikus) Masters, Trans. Linn. Soc. 27: 630. 1871. Type species: Based on *Cieca* Medikus.
Monactineirma Bory. Ann. Gén. Sci. Phys. 2: 138. 1819. Lectotype species, designated here: *Passiflora suberosa* L.
Meioperis Rafinesque, Fl. Tellur. 4: 103. 1838. Lectotype species, designated here: *Passiflora suberosa* L.

#### Type species.

Based on *Cieca* Medikus.

#### Description.

**Figure 22.**
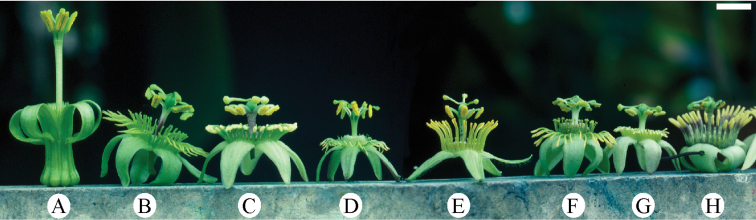
Flowers of several species of Passiflora supersection Cieca **a** *Passiflora viridiflora* (*MacDougal 351GR*) **b** *Passiflora juliana* (*MacDougal 492GR*) **c** *Passiflora trinifolia* (*MacDougal 637GR*) **d** *Passiflora eglandulosa* (*MacDougal 316*) **e** Passiflora suberosa subsp. litoralis (*MacDougal 568*) **f** Passiflora suberosa ssp. litoralis (*MacDougal 1486*) **g** *Passiflora obtusifolia* Mexico (*MacDougal 495GR*) **h** *Passiflora mcvaughiana* (*MacDougal 369GR*) Scale bar = 8.0 mm. Image a composite of two photographs taken by J.M. MacDougal.

Small to medium-sized climbing or procumbent vines with perennial stems from woody perennial rootstocks or taproots, antrorsely appressed-puberulent more or less throughout, with unicellular, curved or occasionally erect trichomes, and sometimes sparsely to densely pubescent with longer unicellular, rarely multicellular, curved trichomes. Stems terete to somewhat compressed and two-edged, the shoot apex erect. Leaves simple, commonly bearing nectaries on the petiole (except in *Passiflora eglandulosa* and *Passiflora mcvaughiana*); petioles sometimes canaliculate, biglandular (rarely eglandular or with only a single gland) with opposite, subopposite or alternate, discoid, cupulate, obconical or capitate extrafloral nectaries; laminas unlobed or 2- to 3-lobed (rarely 5-lobed), often exhibiting heterophylly, sometimes cordate at base, entire (very rarely crenate), venation palmate, variegated or not, peltate or not, sometimes bearing small abaxial disciform or crateriform nectaries present ± submarginally between the major veins (very rarely associated with leaf crenations). Stipules setaceous to foliaceous, persistent, narrowly to widely ovate, rarely oblong or obovate, symmetrical or sometimes asymmetrical, entire, not glandular. Tendrils simple, lacking adhesive disks, straight or slightly curved during development at shoot apex. Inflorescences sessile in leaf axils, the pedicels solitary or paired, collateral with tendril, articulate, the articulation generally several mm below the flower; secondary inflorescences sometimes present as condensed axillary or usually terminal shoots, determinate or usually indeterminate; bracts 1–2 or lacking, narrowly ovate to entire. Flowers erect or rarely ± horizontal, greenish yellow sometimes with purplish to reddish markings, or red, hypanthium usually shallow, occasionally the calyx basally connate into a conspicuous floral tube; sepals ovate-triangular, not corniculate, greenish yellow, red, or rarely whitish; coronal filaments in 2 series (rarely 1 or 7 series), greenish yellow, sometimes with yellow and/or purple to red markings, or purple to red (sometimes very dark reddish purple), linear, often subcylindrical in cross-section, inner filaments usually capitate; operculum connate, membranous, plicate (very rarely denticulate), incurved or rarely semierect and laying against androgynophore; nectary trough-shaped or rarely absent, commonly lacking or possessing a very inconspicuous nectar ring or annulus; limen adnate to floor of hypanthium or rarely absent (in *Passiflora viridiflora* the limen present as a shallow cup around base of androgynophore), the edge commonly erect and inclined toward the nectary, rarely curved toward the androgynophore. Staminal filaments with the free portions actinomorphic; anthers commonly extrorse at anthesis with their axes maintained parallel, rarely perpendicular, to the filament or rarely the anthers move only slightly from the original introrse position, remain introrse, and dehisce distally (upwards); pollen ellipsoid to spherical, 6-syncolporate. Carpels 3; ovary ellipsoid or globose, rarely slightly ovoid, obovoid or fusiform, glabrous or rarely densely pubescent with curved, unicellular or rarely multicellular trichomes; styles slender, less than 1.5 mm in diameter; stigmas capitate, depressed-ovoid. Fruit a one (rarely) to many-seeded purple or very dark purple berry, arils pale-translucent covering approximately 3/4 of the seed. Seeds more or less compressed, often beaked at chalazal apex, reticulate-foveate. Germination epigeal. Chromosome numbers: n = 6 (12, 18). Commonly lacking c-glycosylflavones and usually containing flavonol 3-O-glycosides. Fig. 22

#### Key to the species of *Passiflora* supersection Cieca

**Table:** 

1	Stipules 2.2–11.3(-15.0) mm wide	**2**
–	Stipules less than 1.5 mm wide	**5**
2	Leaves peltate, deeply trilobed (0.42-)0.50–0.86 the distance from the leaf outline to the leaf base, base truncate, central lobe narrowed at the base; 4–11 laminar nectaries present on the abaxial surface; petiolar glands present; southwestern Mexico	**11. *Passiflora juliana***
–	Leaves not peltate, distinctly trilobed 0.30–0.45 the distance from the leaf outline to the leaf base or bilobed to obscurely trilobed 0.02–0.29 the distance from the leaf outline to the leaf base, base cordate, central lobe not narrowed at the base; laminar nectaries absent or 1–4 nectaries present on the abaxial surface; petiolar glands absent or present	**3**
3	Petiolar glands absent (or extremely rarely inconsistently present); lateral leaf lobes 0.64–0.97 times the length of the central lobe;laminar nectaries absent; El Salvador, Guatemala	**7. *Passiflora eglandulosa***
–	Petiolar glands present; lateral leaf lobes 0.67–1.86 times the length of the central lobe; laminar nectaries absent or present	**4**
4	Laminar nectaries absent; lateral leaf lobes 1.41–1.86 times the length of the central lobe; 2 petiolar nectaries borne on the proximal half of the petiole (0.44–0.50 of the distance from the base toward the apex of the petiole); southwestern Mexico	**14. *Passiflora tacanensis***
–	Laminar nectaries 1–4, positioned near or at the leaf sinuses; lateral lobes 0.67–1.28 times the length of the central lobe; 2 petiolar nectaries borne proximally or distally on the petiole (0.29–0.90 of the distance from the base toward the apex of the petiole); Guatemala	**8. *Passiflora trinifolia***
5	Leaves peltate; sparsely to lightly pubescent with trichomes (0.2-)0.4–1.0 mm long; sepals greenish yellow to white	**6**
–	Leaves not peltate; sparsely to densely pubescent with trichomes (0.2-)0.4–1.0 (-1.4) mm long; sepals red or greenish yellow to white	**16**
6	Leaves as long as or longer than wide; capitate or somewhat discoid petiolar nectaries present; flowers not borne in leafless inflorescences or very rarely inflorescences present	**7**
–	Leaves wider than long; discoid petiolar nectaries present or absent, flowers sometimes borne in leafless inflorescences or rarely (*Passiflora clypeophylla*) inflorescences absent	**8**
7	Laminar nectaries absent; leaf base cuneate to acute; sepals (2.3-)4.0–7.0 (-8.3) mm long; hypanthium 2.8–4.1 mm wide; androgynophore (1.7-)2.2–3.5 mm long; outer coronal filaments 1.2–4.0 mm long; inner coronal filaments less than 1.4 mm long; staminal filaments 1.4–3.0 mm long, pollen yellow; fruits globose or ellipsoid; New World tropics, introduced in Old World tropics	**1. *Passiflora pallida***
–	Laminar nectaries present or absent; leaf base commonly cordate or cuneate to acute; sepals 4.0–14.6(-20.5) mm long; hypanthium (3.0-)4.0–8.8 mm wide; androgynophore (2.1-) 2.7–6.1(-12.6) mm long; outer coronal filaments 2.5–8.1 mm long; inner coronal filaments more than 1.4 mm long; staminal filaments 1.6–6.0(-6.8) mm long, pollen whitish or yellow; fruits ovoid, ellipsoid or transversely ellipsoid; New World tropics, introduced in Old World tropics	**2. *Passiflora suberosa***
8	Laminar nectaries absent throughout; petiolar nectaries absent or rarely 1–2 nectaries present; fruits with (2-)6–11 seeds per fruit, seeds more than 3.5 mm wide; southwestern Mexico	**13. *Passiflora mcvaughiana***
–	Laminar nectaries present on the distal leaf blades or sometimes absent; petiolar nectaries present; fruits commonly with more than 11 seeds per fruit, seeds less than 3.5 mm wide	**9**
9	Flowers with the corona in seven series; outer coronal filaments very dark reddish purple with yellow tips; floral nectary absent, operculum denticulate	**10**
–	Flowers with the corona in one or two series, outer coronal filaments greenish yellow, greenish yellow with yellow tips, greenish yellow with a flush of reddish purple at base and yellow at tips, reddish purple at base, greenish yellow at middle, yellow at tips, or white with a reddish purple base and appearing banded with light reddish purple near middle; floral nectary present; operculum plicate	**11**
10	Androgynophore 2.7–4.1 mm long; 40–50 filaments in the outer coronal row; androecium and gynoecium greenish yellow; anthers dehiscing proximally; styles 4.1–6.3 mm long including stigmas; southeastern Mexico, Belize, Guatemala	**19. *Passiflora xiikzodz***
–	Androgynophore absent to 1.7 mm long; 22–31 filaments in the outer coronal row; androecium and gynoecium reddish purple; anthers dehiscing distally; styles 1.8–3.1 mm long including stigmas; southeastern Mexico	**18. *Passiflora itzensis***
11	Androgynophore 17.4–26.1 mm long; corona in one series, 36–50 filaments; sepals 20.5–30.1 mm long, at least six times longer than wide, fused into elongate tube; pollen presented laterally; southwestern Mexico	**12. *Passiflora viridiflora***
–	Androgynophore less than 11 mm long; corona in two series, outer corona with 28–53 filaments, inner corona with 12–50 filaments; sepals 4.7–20.5 mm long, up to three times longer than wide, not fused; pollen presented subproximally to proximally	**12**
12	Leaves obscurely trilobed (0.02–0.07 the distance from the leaf outline to the leaf base) and subrotund; Guatemala	**9. *Passiflora clypeophylla***
–	Leaves distinctly trilobed (0.30–0.61 the distance from the leaf outline to the leaf base) or bilobed to obscurely trilobed (from 0.02–0.29 the distance from the leaf outline to the leaf base) and transversely elliptic	**13**
13	Outer coronal filaments 1.3–3.0(-4.3) mm long, strongly curved at the base so that the filaments spread ca. horizontally, with the tips often curved toward the sepals, linear, often capitellate; inner coronal filaments 0.9–3.3 mm long, with the inner coronal filaments commonly three quarters the length of to equal in length to the outer coronal filaments; leaves distinctly trilobed (0.36–0.60 the distance from the leaf outline to the leaf base) or bilobed to obscurely trilobed (0.09–0.28 the distance from the leaf outline to the leaf base); laminar nectaries present or absent; Mexico, El Salvador, Costa Rica	**10. *Passiflora obtusifolia***
–	Outer coronal filaments 3.1–14.0 mm long, suberect at base and spreading ca. 30–100° with the tips more or less curved toward the androgynophore, linear, sometimes slightly dilated toward tip; inner coronal filaments 1.4–5.6 mm long, with the inner coronal filaments commonly 1/2–3/4 the length of the outer coronal filaments; leaves bilobed to obscurely trilobed (0.02–0.27 the distance from the leaf outline to the leaf base) or distinctly trilobed (0.31–0.61 the distance from the leaf outline to the leaf base); laminar nectaries present	**14**
14	Limen floor very dark reddish purple or heavily spotted with very dark reddish purple; outer coronal filaments very dark reddish purple at base, greenish yellow at middle and yellow at tips; fruits globose; Mexico, Belize, Guatemala, Honduras, Nicaragua	**17. *Passiflora sexocellata***
–	Limen floor greenish yellow or greenish yellow with some reddish purple spots and streaks; outer coronal filaments greenish yellow with yellow tips, greenish yellow with a flush of reddish purple at base and yellow at tips, or white with a reddish purple base and appearing banded with light reddish purple near middle; fruits globose or ellipsoid	**15**
15	Outer coronal filaments white with a reddish purple base and appearing banded with light reddish purple near middle, 3.1–5.3(-7.0) mm long; inner coronal filaments 1.4–3.2 mm long; hypanthium 4.9–7.4(-8.1) mm wide; androgynophore (3.3-)3.8–5.0 mm long; fruits globose; Colombia, Bolivia, Ecuador, Peru. Venezuela	**15. *Passiflora coriacea***
–	Outer coronal filaments greenish yellow with yellow tips, sometimes with a flush of reddish purple at base, 6.8–14.0 mm long; inner coronal filaments 2.3–5.6 mm long; hypanthium (7.8-)8.1–16.1 mm wide; androgynophore 4.1–10.0 mm long; fruits ellipsoid; Costa Rica, Panama, Colombia	**16. *Passiflora megacoriacea***
16	Androgynophore 17.8–23.5 mm long; flowers red; laminar nectaries absent; densely pubescent	**17**
–	Androgynophore 1.7–14.1 mm long; flowers greenish yellow or whitish; laminar nectaries present or absent; sparsely to densely pubescent	**18**
17	Leaves unlobed or shallowly trilobed, lateral lobes usually less than half the length of the central lobe, central lobe not narrowed at the base; pedicels 2.4–5.5 cm long; outer coronal filaments free from sepals; fruits globose; Jamaica	**4. *Passiflora lancifolia***
–	Leaves distinctly trilobed, lateral lobes more than half the length of the central lobe, central lobe distinctly to obscurely narrowed at the base; pedicels 1.1–1.8(-2.3) cm long; outer coronal filaments adnate to sepals; fruits fusiform; Jamaica	**5. *Passiflora macfadyenii***
18	Fruits fusiform; outer coronal filaments 5.7–8.9 mm long; androgynophore 8.0–10.8(-14.1) mm long; Galapagos Islands, Ecuador	**3. *Passiflora tridactylites***
–	Fruits globose, ellipsoid, transversely ellipsoid, or ovoid; outer coronal filaments 1.2–6.0(-8.1) mm long; androgynophore 1.7–12.6 mm long	**19**
19	Leaves as long as or longer than wide	**20**
–	Leaves wider than long	**21**
20	Laminar nectaries absent; leaf base cuneate to acute; sepals (2.3-)4.0–7.0 (-8.3) mm long; hypanthium 2.8–4.1 mm wide; androgynophore (1.7-)2.2–3.5 mm long; outer coronal filaments 1.2–4.0 mm long; inner coronal filaments less than 1.4 mm long; staminal filaments 1.4–3.0 mm long, pollen yellow; fruits globose or ellipsoid; New World tropics, introduced in Old World tropics	**1. *Passiflora pallida***
–	Laminar nectaries present or absent; leaf base commonly cordate or cuneate to acute; sepals 4.0–14.6(-20.5) mm long; hypanthium (3.0-)4.0–8.8 mm wide; androgynophore (2.1-)2.7–6.1(-12.6) mm long; outer coronal filaments 2.5–8.1 mm long; inner coronal filaments more than 1.4 mm long; staminal filaments 1.6–6.0(-6.8) mm long, pollen whitish or yellow; fruits ovoid, ellipsoid, or transversely ellipsoid; New World tropics, introduced in Old World tropics	**2. *Passiflora suberosa***
21	Central vein length less than half the width of the leaf; central and/or lateral lobes often lobed; laminar nectaries commonly absent, petiolar glands positioned at or near the petiole apex, only very rarely found proximally; flowers not borne in inflorescences; floral stipes 1.1–4.1 mm long; U.S.A. (Texas), northern Mexico	**6. *Passiflora tenuiloba***
–	Central vein length more than half the width of the leaf; central and lateral lobes not lobed; laminar nectaries present or absent; petiolar glands present on the distal half of the petiole; flowers usually borne in inflorescences; floral stipes 3.1–4.6 mm long; Mexico, El Salvador, Costa Rica	**10. *Passiflora obtusifolia***

### Species descriptions

#### 1. Passiflora pallida

Taxon classificationPlantaeMalpighialesPassifloraceae

L. Sp. Pl. 955. 1753. non Passiflora pallida Lour., 1790. non Passiflora pallida Vell., 1827.

Figs 23 , 24

Passiflora hirsuta L. Sp. Pl. 958. 1753. non *Passiflora hirsuta* Loddiges, 1818. Type: Hispaniola, (no specimens extant; lectotype, designated here: Plumier Desc. Pl. Amer. pl. 88. 1693).
Passiflora minima L. Sp. Pl. 959. 1753. non *Passiflora minima* Blanco, 1837. Type: “Curassao,” [Curacao, Netherlands West Indies] *20* (lectotype, designated by Wijnands 1983 pg. 171: LINN 1070.20, microfiche seen).
Passiflora nigra Jacq., Observ. Bot. (Jacquin) 2: 27, pl. 46, fig. 3. 1767. Type: [Colombia, Cartagena, Boca Chica Inlet] (lectotype, designated here: Jacquin Observ. Bot. 2: 27 pl. 46, fig. 3. 1767).
Passiflora glabra Mill., Gard. Dict., eight ed., no. 4. 1768. non *Passiflora glabra* J.C. Wendl., 1805. Type: Based on *Passiflora pallida* L.
Passiflora parviflora Sw., Prodr. [O.P. Swartz] 97. 1788. Type: Jamaica, *O. P. Swartz s.n.* (holotype: S [S03-900][photograph seen]; isotype: MO! [MO-312541]).
Passiflora heterophylla Dryand., Hortus Kew. [W. Aiton] 3: 309. 1789. non *Passiflora heterophylla* Lam., 1789. non *Passiflora heterophylla* Jacq., 1797. Type: at Kew Gardens in ca. 1773, from the West Indies (no type material found).
Cieca minima (L.) Moench., Suppl. Meth. 102. 1802. Type: Based on *Passiflora minima* L.
Passiflora warei Nutt., Amer. J. Sci. Arts 5: 297. 1822. Type: United States of America. Florida: “Florida”, *A. Ware s.n.* (holotype: BM, ! [BM000563877][photograph AAU!]).
Meioperis pallida (L.) Raf., Fl. Tellur. 4: 103. 1838. Type: Based on *Passiflora pallida* L.
Meioperis minima (L.) Raf., Fl. Tellur. 4: 103. 1838. Type: Based on *Passiflora minima* L.
Cieca pallida (L.) M.Roem., Fam. Nat. Syn. Monogr. 2: 142. 1846. Type: Based on *Passiflora pallida* L.
Cieca warei (Nutt.) M.Roem., Fam. Nat. Syn. Monogr. 2: 146. 1846. Type: Based on *Passiflora warei* Nutt.
Passiflora hirsuta var. parviflora (Sw.) M.Roem., Fam. Nat. Syn. Monogr. 2: 174. 1846. Type: Based on *Passiflora parviflora* Sw.
Passiflora suberosa var. minima (L.) Mast., Trans. Linn. Soc. London 27: 630. 1871. Type: Based on *Passiflora minima* L.
Passiflora suberosa var. hirsuta (L.) Mast., Trans. Linn. Soc. London 27: 630. 1871. Type: Based on *Passiflora hirsuta* L.

##### Type.

l’Isle S. Domingue (lectotype designated here: Plumier, Pl. Amer. pl. 89. 1693).

##### Description.

Slender, climbing, perennial vine 1–7 m long or more, sparsely to densely pubescent with unicellular curved trichomes on petiole, leaf, stem, and stipule, 0.20–0.30(-0.7) mm long, 0.02–0.03 mm wide, also minutely antrorsely appressed-puberulent throughout with unicellular, curved trichomes, 0.06–0.11 mm long, 0.02–0.03 mm wide. Flowering stems 0.6–1.6(-2.5) mm in diameter, terete or somewhat compressed, greenish yellow to very dark reddish purple, with the base woody and cork-covered. Stipules 2.1–6.9 mm long, 0.2–0.9 mm wide, narrowly ovate-triangular, sometimes slightly falcate, acute; petioles 0.3–1.8(-2.9) cm long, with 2 (rarely 1), opposite to alternate, stipitate or sometimes sessile, slightly obconical to capitate nectaries (very rarely crateriform), 0.3–0.8 mm wide (on the widest axis), 0.2–1.1 mm high, borne in the distal half of the petiole (0.49–0.92 of the distance from the base toward the apex of the petiole). Laminas 1.8–8.8(-12.0) cm long, (0.3-)1.4–8.2(-10.6) cm wide, membranous, unlobed to 3-lobed, lobed 0.20–0.50(-0.90) the distance to the leaf base, ovate to elliptic (rarely obovate), base cuneate to acute, lateral lobes 1.0–5.1(-6.8) cm long, 0.3–2.1(-3.0) cm wide, ovate to oblong, acute (rarely obtuse or rounded), central lobe ovate to elliptic (rarely obovate), central vein 1.8–8.8(-12.0) cm long, angle between the lateral lobes (33-)50–110(-152)°, ratio of lateral lobe to central vein length 0.46–0.78(-0.87), margins entire, hyaline, primary veins 1–3 (when more than one veins diverge and branch at base), laminar nectaries absent; tendril 0.2–0.7(-1.1) mm wide, present at flowering node. Flowers borne in leaf axils. Pedicels (2.0-) 3.3–9.4(-17.0) mm long, 0.3–0.6 mm wide, 2 per node; bract(s) absent or rarely with one narrowly ovate, acute, bract present on the distal half of the pedicel, 0.4–0.6 mm long, ca. 0.1 mm wide; spur(s) absent. Flowers (6.9-)11.7–20.4 mm in diameter with stipe 1.4–4.4(-6.3) mm long, 0.3–0.7 mm wide; hypanthium 2.8–4.1 mm in diameter; sepals (2.3-)4.0–7.0(-8.3) mm long, 1.2–3.3 mm wide, ovate-triangular, acute to rounded, reflexed at anthesis, abaxially and adaxially greenish yellow to very light greenish yellow (5GY 7/4, 8/4–8/2); coronal filaments in 2 series, the outer 20–30(-34), 1.2–4.0 mm long, (0.1-)0.2–0.6 mm wide, linear, slightly spreading, greenish yellow with yellow tips (5Y 8/10) or flushed with reddish purple (5RP 5/6–3/6) at base and greenish yellow at middle with yellow tips or very dark reddish purple (5RP 3/4–2.5/4) at base and yellow toward tips, ratio of outer coronal row to sepal length 0.20–0.69(-0.82), the inner (11-)20–34, 0.8–1.3 mm long, 0.04–0.16 mm wide, linear, capitate, erect, greenish yellow with yellow tips or greenish yellow flushed with reddish purple at base and yellow toward tips or very dark reddish purple with yellow tips, ratio of inner coronal row to outer coronal row length 0.36–0.66; operculum (0.6-)1.0–1.4 mm long, plicate, greenish yellow or greenish yellow with a flush of reddish purple at base or reddish purple or very dark reddish purple, margin white with minutely fimbrillate teeth; nectary (0.1-)0.2–0.4(-0.6) mm high, 0.2–0.6(-0.8) mm wide; limen recurved, erect or slightly inclined toward the operculum, 0.1–0.4 mm high, 0.1–0.3 mm wide, greenish yellow or greenish yellow flushed with reddish purple or reddish purple or very dark reddish purple, limen floor 1.6–2.6 mm in diameter, greenish yellow or greenish yellow flushed with reddish purple or reddish purple or very dark reddish purple; androgynophore (1.7-)2.2–3.5 mm long, 0.4–0.9 mm wide, greenish yellow or greenish yellow with a flush of reddish purple at base or greenish yellow with reddish purple spots and streaks or very dark reddish purple; free portions of the staminal filaments 1.4–3.0 mm long, 0.2–0.4 mm wide, linear, greenish yellow; anthers 1.1–1.9 mm long, 0.5–1.3 mm wide, pollen yellow; styles 1.6–4.3 mm long including stigmas, 0.1–0.4 mm wide, greenish yellow; stigmas 0.5–1.2 mm in diameter; ovary 1.1–1.8 mm long, (0.7-)1.0–1.5(-1.9) mm wide, ellipsoid to globose, greenish yellow. Berry 7.6–9.5 mm long, 6.9–8.8 mm in diamater, globose, or ellipsoid, very dark purple (5P 2.5/2). Seeds (4-)8–24(-33), 2.8–3.5 mm long, 1.9–2.2 mm wide, 1.1–1.4 mm thick, obovate in outline, acute at both ends, reticulate-foveate with each face marked with ca. 12–20 foveae; germination type epigeal.

**Figure 23.**
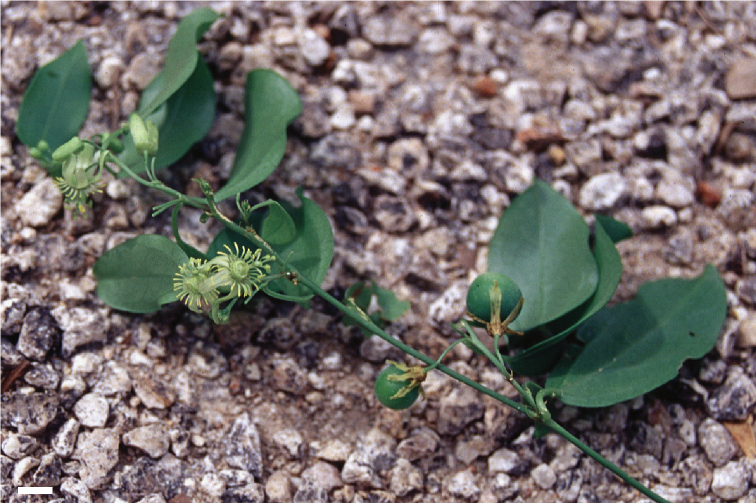
*Passiflora pallida* (*Porter-Utley & Mondragón 412*) from the Yucatán Peninsula, Mexico. Scale bar = 4.0 mm.

**Figure 24.**
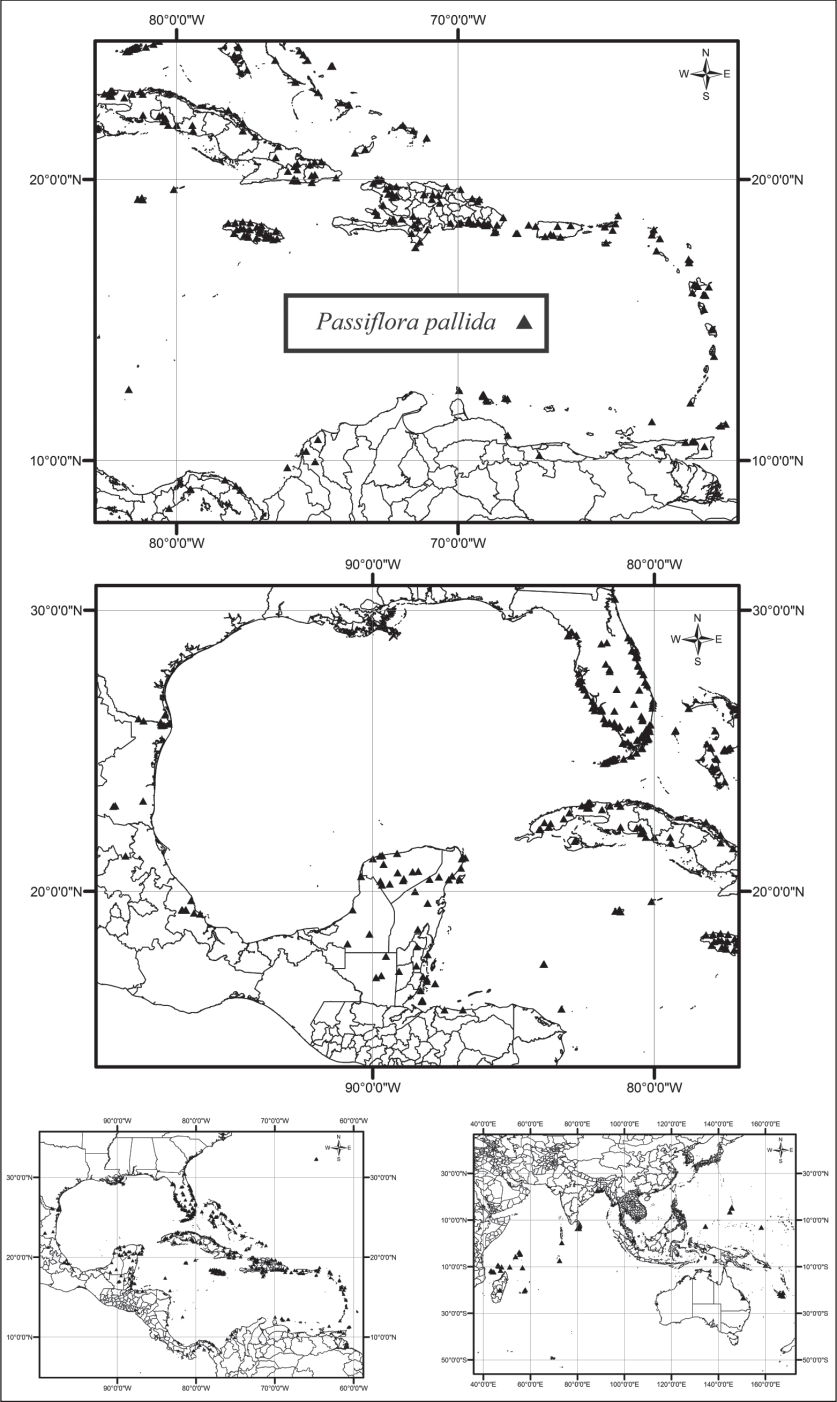
Distribution of *Passiflora pallida*.

##### Phenology.

Flowering and fruiting throughout the year.

##### Distribution.

In the New World tropics: Central America, Mexico, United States (Florida and Texas), Venezuela, and the West Indies. Introduced in the Old World tropics: Africa, Asia, and Australia. Growing in shrubs, trees or trailing on the ground in secondary successional areas and along the edges of dry tropical forests, both inland and near the seashore, primarily at low elevations but sometimes occurring at elevations as high as 800 m. Commonly associated with calcareous/alkaline substrate.

##### Ethnobotany.

In Réunion, the fruits may be used as a substitute for ink (Jean Jacques, pers. comm.).

##### Discussion.

*Passiflora pallida* as recognized here exhibits a substantial amount of morphological variation across its range. The various forms that the leaves may take have led to the proposal of many species and varietal names. For example, a plant of this species may possess only unlobed leaves, only trilobed leaves, or leaves that are unlobed, bilobed, and trilobed. This type of variation can be seen throughout the range of this species. However, the flowers of *Passiflora pallida* are diagnostically small, with a narrow hypanthium, short sepals, short coronal filaments, and narrow floral nectaries.

The only species with which *Passiflora pallida* may be confused is *Passiflora suberosa*. *Passiflora pallida* is vegetatively similar to both Passiflora suberosa subsp. litoralis and Passiflora suberosa subsp. suberosa, and without flowering material these taxa can be difficult to distinguish. The position of the petiolar nectaries has often been used to separate species in closely related taxa in *Passiflora*. However, though the petiolar nectaries are generally located closer to the petiole apex in *Passiflora pallida* than in the South American populations of Passiflora suberosa subsp. litoralis and Passiflora suberosa subsp. suberosa, the upland Mexican/Central American populations of Passiflora suberosa subsp. litoralis also have petiolar nectaries positioned very near the petiole apex. The leaf base of *Passiflora pallida* is commonly not cordate. *Passiflora suberosa* possesses leaves that are frequently cordate, though this character is somewhat variable in the upland Mexican/Central American populations of Passiflora suberosa subsp. litoralis. Though foliage color is difficult to discern from herbarium specimens, my experience in the field and photos taken of *Passiflora pallida* and *Passiflora suberosa* in the field by others show that *Passiflora pallida* commonly possesses leaves that are paler in color and often less lustrous than *Passiflora suberosa*. Reproductive structures are more reliable in separating *Passiflora pallida* and *Passiflora suberosa*. The hypanthium of *Passiflora pallida* is commonly 2.8–4.1 mm in diameter and the inner coronal filaments are usually less than 1.5 mm long. In *Passiflora suberosa* *s. l.*, the hypanthium is commonly 4.0–8.8 mm in diameter and the inner coronal filaments are frequently 1.5–3.9 mm long. The outer coronal filaments are also short, less than 4.0 mm in *Passiflora pallida*, and although that overlaps with the 2.5–8.1 mm range observed in *Passiflora suberosa*, the character is frequently observable in herbarium specimens. Where the distributions of *Passiflora pallida* and *Passiflora suberosa* overlap in the Antilles, *Passiflora pallida* is typically found in and along the edges of subtropical and tropical forests at or near sea level (rarely exceeding 200 m), whereas *Passiflora suberosa* commonly occurs in and along the edges of tropical forests above 500 m.

The most common variant of *Passiflora pallida* (as exemplified by *E. Killip 41876*, on Sugarloaf Key, Monroe Co., Florida, USA; *E. Cabrera 1475*, S of Akumal, Quintana Roo, Mexico; and *J. Tillich 3558*, in Black River, Mauritius) has ovate leaves that may be unlobed, bilobed or trilobed on the same plant. When unlobed, the leaves are commonly greater than 2.0 cm wide. When lobed, the leaves are usually shallowly lobed 0.20–0.41 the distance to the base, the lateral and central lobes are greater than 1.0 cm wide, and the angle between the lateral lobes is 45–100°. Another less common variant of *Passiflora pallida* (as exemplified by *J. K. Small & C. Mosier 5511*, from Cox Hammock, Miami-Dade Co., Florida, USA; and *J. Small & J. Carter 194*, between Perrine and Long Prairie, Miami-Dade Co., Florida, USA), has narrowly ovate leaves that may be unlobed, bilobed or trilobed on the same plant. When unlobed, the leaves are commonly less than 1.0 cm wide. When lobed, the leaves are usually deeply lobed 0.82–0.90 the distance to the base, the lateral and central lobes are commonly less than 0.7 cm wide, and the angle between the lateral lobes is greater than 100°. However, all the specimens brought together here as *Passiflora pallida* are all relatively small in stature in their native habitats in the New World, possess similar small flowers with short coronal filaments and occur in a similar range of elevations.

MacDougal has reported the appearance of an occasional, well-formed but small petal in other species within supersection *Cieca* (MacDougal, 1992). I have also seen this in *Passiflora pallida* in several of my greenhouse accessions and in the field in Quintana Roo, Mexico.

Stegmaier (1973) reported that *Dasiops passifloris* McAlpine (Diptera: Family Lonchaeidae) infests the fruits of *Passiflora pallida* in southern Florida. He found that the female fly oviposits on the fruits and the larvae feed on the arils and fruit flesh. In this study, in which he collected a total of 1040 wild passion fruits from *Passiflora pallida* occurring on a single farm in Hialeah, Florida, he also found that the mature fruit may contain from 4 to 17 seeds per fruit (Stegmaier 1973).

*Passiflora pallida* is a pest plant where it occurs in many areas of the Old World. In New Guinea, Neville Kemp reports that the probable disperser of *Passiflora pallida* is the long-tailed macaque or crab-eating macaque (*Macaca fascicularis*) (Kemp and Burnett 2003).

In Linnaeus’ 1753 edition of *Species Plantarum*, he describes three small-flowered entities, *Passiflora pallida* L. (“Habitat in Dominica, Brasilia”), *Passiflora hirsuta* L. (“Habitat in Dominica and Curassao”) and *Passiflora minima* L. (“Habitat in Curassao”), for which the historical references include phrases such as ”flore minore” (*Passiflora pallida* L.), “flore & fructu minimis” (*Passiflora hirsuta*) and “flore flavescente omnium minimo” (*Passiflora minima* L.). Charles Wright (1869), in an article discussing the genus *Jussiaea* L., chose the name *Passiflora pallida* over *Passiflora minima*. In the article he commented on the “embarrassing” status of the species of *Passiflora* and the unwise reliance upon vegetative morphology in species circumscription within the Cuban species of *Passiflora* (Wright, 1869:480). In the article he states, “I have lately carefully examined the Cuban species called *Passiflora minima*, *hederacea*, *pallida*, *angustifolia*, *suberosa*, &c., and come to this conclusion:–*Passiflora pallida*, L., is an old and appropriate name, to which belong *Passiflora minima*, L., and *Passiflora angustifolia*, Sw., certainly; *Passiflora hederacea*, Cav., *Passiflora suberosa* L., probably; and, from the description, I judge *Passiflora lineariloba*, Hook. f. to be only another form of it.” It is possible that *Passiflora hirsuta* was not considered by Wright in his article because of the confusion surrounding its circumscription (see below) or because he had not encountered the taxon in Cuba.

In the 1753 edition of *Species Plantarum*, Linnaeus indicated that he was well-acquainted with *Passiflora pallida* and refers to the diagnosis and drawing in his *Dissertatio botanica de* Passiflora (1745), that shows an unlobed, ovate leaf with two petiolar nectaries positioned near the apex of the petiole. Linnaeus cites an illustration by Plumier (*pl. 89*, in *Description des plantes de l’Amérique* 1693) that also exemplifies his *Passiflora pallida*. However, he also refers to a figure by Morison (1680) that shows a plant with a large flower that possesses sepals and petals (likely in the subgenus *Passiflora*) with unlobed, ovate leaves. In the 1745 dissertation, Hallman specifically states that the flowers of *Passiflora pallida* L. are “pentapetala”, referring to the lack of petals; this decision was based upon the careful comparison of diagnoses from other petalous taxa in the treatment. An examination of the Linnaean herbarium (microfiche) did not reveal an herbarium specimen that could reasonably be attributed to the species described as *Passiflora pallida* by Linnaeus. There is one specimen in the Linnaean herbarium labeled *Passiflora pallida*, but it is a post 1753 accession that represents a large-flowered taxon from subgenus *Passiflora*. Though there is a small amount of confusion surrounding *Passiflora pallida* L., largely attributable to Linnaeus’ reference to Morison’s illustration and the post-Linnaean accession referred to above, it is clear from the diagnoses in the 1753 edition of *Species Plantarum* and the 1745 dissertation, that Linnaeus was referring to a plant that had unlobed, ovate leaves and small, pale, apetalous flowers (Jarvis 2007). The lectotype of *Passiflora pallida* L. (designated here) is Plumier’s fig in *Description des plantes de l’Amérique* (1693) in which he illustrated several entities of both *Passiflora pallida* and Passiflora suberosa subsp. suberosa. Incidentally, Linnaeus chose the epithet, *pallida*, to refer to the pale-colored flower. Though the flowers are frequently pale in color, they may also be highly colored.

Linnaeus (1753) also describes *Passiflora minima* L. as a trilobed plant in which the central lobe is longer than the lateral lobes. He cites the diagnosis and drawing in the 1745 dissertation that shows a plant with narrowly trilobed leaves that lack petiolar glands. Linnaeus (1753) also refers to a figure by Plukenet (1696) that closely matches Linnaeus’ diagnoses and the drawing in the dissertation. Neither Linnaeus nor Hallman described the flowers of *Passiflora minima* L., but it can be inferred by the historical references in the dissertation that the flowers were small and lacked petals. In Killip’s treatment of *Passiflora suberosa*, he states that there are two sheets of *Passiflora minima* from the “West Indies” of uncertain origin in the Linnaean Herbarium and designated them “type of *Passiflora minima*” (1938:93) without specifying one of the sheets specifically. According to Jarvis (2007), the lectotype of *Passiflora minima* L. (designated by Wijnands 1983) is specimen 1070.20 (LINN). The lectotype closely matches Linnaeus’ diagnoses and the drawing in the dissertation and possesses small flowers apparently lacking petals (as observed on a microfiche of the herbarium). However, the lectotype of *Passiflora minima* is a very unusual example of the small-flowered entity, as the lack of petiolar nectaries in this taxon is very rare.

*Passiflora hirsuta* L. has been the source of confusion for several taxonomists of *Passiflora*, and under his treatment of Passiflora foetida var. moritziana (Planch.) Killip ex Pulle, Killip (1938) discussed the problem. Linnaeus (1753) cited several references in his treatment of *Passiflora hirsuta*, often with accompanying illustrations, that undoubtedly refer to *Passiflora foetida*. However, he also referred to an illustration by Plumier (pl. 88, in *Description des plantes de l’Amérique* 1693) that is clearly *Passiflora pallida*. As in his other species descriptions, he also cites the diagnosis and drawing in the 1745 dissertation by Hallman that shows a trilobed, densely pubescent leaf with rather large petiolar nectaries that are positioned on the distal half of the petiole and, thus, cannot be *Passiflora foetida* as this species lacks petiolar nectaries. The diagnosis in the 1753 edition of *Species Plantarum* is unclear. However, in the 1745 dissertation Hallman states that the flowers of this taxon are pale and small, the involucre is lanceolate, and the fruits are deep blue. Hallman goes on to say that the taxon that he is describing is somewhat similar to the next (*Passiflora foetida* L.) but differs in that the flowers are opposite (paired) and the involucre consists of only a single bract. Hallman is clearly describing one of the entities in the *Passiflora suberosa* complex, as the flowers are commonly paired in the leaf axils, members of the species complex do sometimes possess one or two lanceolate bracts, and the fruits are very dark purple. In *Passiflora foetida* only one flower is present in the leaf axils, the involucre consists of three large bracts that are pinnatifid or pinnatisect, and the fruits are yellow to red. Though the leaf as illustrated in the dissertation is distinctly cordate and broadly ovate, which is a bit unusual for *Passiflora pallida*, Linnaeus’ reference to Plumier’s drawing leads me to conclude that *Passiflora hirsuta* L. is a synonym of *Passiflora pallida*. It is also the only original material that corresponds to the current concept of the species.

##### Selected specimens examined.

**United States. Florida:** *Brevard Co*.: near Cape Malabar, *Curtiss 974* (BM, G, GH, M, MIN, MO, NY, US). *Broward Co*.: along U.S. 27, 6 mi. N of Andytown, *Beckner 769* (MO); along canal N of Rt. 84 near Florida State Forestry Station, W of Florida Turnpike, *Correll et al. 40198* (MO); along US 27, 1 mi. S of intersection with Fla. 84 at Andytown (“Twenty Mile Bend”), ca. 18 mi. W of Ft. Lauderdale, *Ward & Burch 3334* (FLAS). *Charlotte Co*.: Bull Key, opposite Lemon City, *Small & Carter s.n.*, 6 November 1903 (NY). *Collier Co*.: on tram, lower Fahkahatchee strand, *Atwater 684* (FLAS); Goodland Point, Marco Island, *Brass 18081* (FLAS, US); lagoon embankment W of Everglades City, *Lakela 31716* (CONN, FLAS, GH, MIN). *Duval Co*.: Mouth of the St. John’s River, *Curtiss 973* (FLAS, G, GH, M, MO, NY, US). *Hendry Co*.: E side on dirt road, 5 mi. S of Fla. 846, ca. 10 mi. due ESE of Immokalee, *Ward et al. 5396* (FLAS). *Highlands Co*.: The Archbold Biological Station, *Cooley et al. 9434* (GH). *Hillsborough Co*.: 2511 LaSalle St., *Almeda 367* (FLAS). *Indian River Co*.: near Roseland; near corner of 110th St. along road bordering Indian River, 0.3 mi. S of junction with US 1., *Wunderlin & Beckner 6490* (NO). *Lake Co*.: in city park, Leesburg, *Baltzell 6619* (FLAS). *Lee Co*.: Pine Island, S of jct. Rt. 767 & Rt. 78, *Lakela, Long & Broome 305897* (FLAS). *Levy Co*.: Waccasassa Bay State Preserve, N of Turtle Creek, *Abbott & Williams 8461* (FLAS); Seahorse Key, *West et al. s.n.*, 1 August 1958 (GH). *Manatee Co*.: Perico Island, *Tracy 7655* (BM, G, GH, MIN, MO, NY, US). *Martin Co*.: S.R. 714, 16 mi. W of Palm City, *McCart 11226* (FLAS). *Miami-Dade Co*.: 0.2 mi. N of SW 288th St., to the W side of SW 170th Ave., Homestead, *Goldman & Hammer 1654* (MO); between Perrine & Long Prairie, *Small & Carter 194* (US); Goodburn Hammock, *Small & Mosier 5923* (G, NY, UC); Miami, *Tracy 9168* (BM, G, GH, MIN, MO, PH, NY, TEX, US); along US 41, 2.2 mi. W of int. with Fla 27 W of Miami, *Ward & Burch 3983* (FLAS). *Monroe Co*.: along road 4A about 0.5 mi. S of Islamorada, *Deam 61084* (DUKE); Sugerloaf Key, *Killip 41876* (B, NO, TEX, US); Key West, Hammock between Flagler Ave. and airport, *Killip 44478* (US); N Key Largo, 6.5 mi. from US 1, on Fla 905, *Long et al. 2800* (MIN); Cox Hammock, *J. K. Small & C. Mosier 5511* (NY); Pinecrest, S of Fla. 94 (“Loop Road”), 4 mi. W of Miami-Dade-Monroe county line, NE corner of *Ward & Burch 3309* (FLAS, GH). *Palm Beach Co*.: Singer Island, 9 mi. S of Lost Tree Village, *McCart 11155* (FLAS). *Pinellas Co*.: N end of Long Key, town of St. Petersburg Beach, near Ciega Bay, *Ward & Ward 2333* (FLAS). *Polk Co*.: vicinity of Crooked Lake, *McFarlin 3958* (TEX). *St. Johns Co*.: S end near South Point loop, *Harrison & Harrison 722* (FLAS). *St. Lucie Co*.: along edge of Indian River, Fla. 707, 1.3 mi. S of jct. with Fla. 712, ca. 4 mi. S of Ft. Pierce city limit, *Ward & Crosby 4839* (FLAS, NY). *Sarasota Co*.: 2482 Linwood Dr., north of Bee Ridge and Webber, east of McIntosh, south of Bahia Vista, west of I-75, *Abbott, J. R. 14284* (FLAS); Manasota Key, 6070 Manasota Key Road (S.R. 776), 3.2 mi. from S.R. 774, *Lott & Lott DT1085* (FLAS). *Volusia Co*.: N end of Meritt Island, Apollo Beach between Turtle Mound and the House of Refuge Site, Cape Canaveral National Seashore, *Judd et al. 3245* (FLAS, NY, U). **Texas:** *Cameron Co*.: La Palmas Plantation, about 4 mi. SW of Brownsville, *Correll 14852* (GH, TEX, US). *Hidalgo Co*.: ca. 0.5 mi. ESE of Anzaldua Dam, ca. 4.5–4.6 airmi. S of jct. of US Rt. 83 and F.M. 1016 at Mission, Río Grand Valley National Wildlife Refuge, Gabrielson Tract, Mission Quadrangle, 105–110 ft., *Carr & Hernández 14374* (LL).

**Antigua and Barbuda.** Antigua, Bodkin Estate, *Box 1258* (BM, US).

**Bahamas. Acklins and Crooked Islands:** Acklins Island, Gold Rock, *Brace 4414* (NY, US); Acklins Island, about 4 mi. N of Pinefield, *Correll 44459* (NY). **Bimini:** S Bimini, *Howard & Howard 10072* (A, GH, NY, S, US). **Cat Island:** Atlantic shore at Bird Point, *Byrne 242* (A). **Exuma:** Hummingbird Cay, at top of Mt. Earlham, *Nickerson & Gross 3017* (A, MO). **Freeport:** Grand Bahama, ca. 0.5 mi. W of Hawksbill Creek channel (vicinity of Eight Mile Rock), *Webster 10914* (US). **Fresh Creek:** Andros, across road from Andros Town Airport, *Fehling 17* (NY). **Governor’s Harbour:** Great Abaco, Abaco, along Queen’s Hwy. about 8 mi. S of Marsh Harbour, *Correll & Wassjausen 52077* (US). **Harbour Island:** N Eleuthera, in coppice near turn-off to road to ferry slip to Harbour Island, about 1.5 mi. N of Lower Bogue, *Correll 40996* (NY). **Kemps Bay:** Andros Island, Mangrove Cay, *Bryant 4* (GH). **Long Island:** upper beach strand, Clarence Town, Harbor Area, *Hill 823* (A). **Marsh Harbour:** Abaco, along Forest Drive, about 1.5 mi. NW of Marsh Harbour, *Correll & Meyer 44600* (NY). **New Providence:** near Harrold & Wilson Ponds, *Degener 18964* (NY). **Nichollstown and Berry Islands:** Anderson Cay, Great Harbour Cay, *Correll & Correll 43669* (NY). **San Salvador:** off Jake Jone’s Road (to Little Lake S from Queen’s Hwy.) near Barker’s Point, NW part of island, *Romansky et al. 13* (FLAS).

**British Overseas Territory. Turks and Caicos Islands:** E Caicos, Jacksonville and vicinity, *Millspaugh & Millspaugh 9073* (NY).

**British Virgin Islands.** Anegada, near settlement, *Britton & Fishlock 1005* (NY); Virgin Gorda, *Fishlock 136* (NY).

**Cuba. Camagüey:** Silla de Cayo, Cayo Romano, *Shafer 2529* (BM, NY, US). **Cienfuegos:** Farallones de Guajímico, on the coast E of Cienfuegos, *Morton 10492* (US). **Ciudad de la Habana:** Near Havana, *Curtis 552* (BM, C, G, GH, M, MINN, MO, NY, PH, PR, US). **Guantánamo:** Beside the Río Jauco, S coast of Oriente, 50 m, *Morton & Alain 9159* (US). **Holguin:** Holguin, Cerro de Fraile, *Ekman 7551* (S). **Isla de Juventud:** along road from Nueva Gerona to Santa Bárbara, *Killip 45109* (US). **La Habana:** between Madruga and Robles, *Shafer 36* (NY). **Matanzas:** vicinity of Matanzas, Empalme, *Britton et al. 584* (NY). **Pinar del Río:** trail from Buenaventura to San Juan de Guacamaya, *Wilson 9348* (NY, U). **Santiago de Cuba:** between Sardinero and Siboney, Santiago, *Clemente 6396* (GH). **Villa Clara:** 5 km W of Santa Clara, *Howard et al. 441* (A, MIN, UC).

**Dominica. Saint Patrick:** SE coast, path between Delices & Belvedere Estate, 250 m, *Whitefoord 3758* (BM).

**Dominican Republic. Baoruco:** Dos Brazos, 8.5 km N of Neiba, 400 m, *Maas et al. 8391* (U). **Barahona:** N end of Beata Island, *Howard 12449* (A, US). **Dist. Nacional:** 8 km from La Batatas (via Laguna La Jagüita) on road to Mata de Piedra and La Catalina, 20 m, *Mejia & Zanoni 9744* (MO, NY). **El Seibo:** vicinity of Higuey, near Canada Honda, *Howard & Howard 9754* (BM, GH, NY, S, US). **Elías Piña:** Vieja Estrelleta, 9.1 km SW de El Cercado en la carretera a Hondo Valle, entre Sonador & Juan Santiago, 740 m, *Zanoni 27976* (JBSD). **Independencia:** aprox. 12 km S de Duvergé, en el lugar llamado Monte Palma, 860 m, *Garcia et al. 4451* (B). **La Altagracia:** half-way between Boca del Yuma town and El Caracol (old bridge over Río Duey, N of town), 20–60 m, *Zanoni et al. 10740* (JBSD, MO, NY). **La Romana:** Río Cumayasa river valley, just N of town of boca de Cumayasa, on SW side of river mouth, 0–20 m, *Mejia & Ramírez 14786* (JBSD, MO, NY). **La Vega:** vicinity of Piedra Blanca, knob S of Piedra Blanca, 200–500 m, *Allard 17783a* (US). **María Trinidad Sánchez:** Cabo Frances Viejo, *Smith 10452* (JBSD, NY). **Monte Cristi:** near Puerto Libertador, Manzanilla Bay, *Howard & Howard 9633* (BM, GH, NY, S, US). **Pedernales:** 22 km N del puerto de Cabo Rojo en la carretera de Alcoa Exploration Company a las Mercedes & Aceitillar, 400 m, *Zanoni & Pimentel 25943* (JBSD). **Puerto Plata:** en manigua costera, Cabarete, Puerto Plata, *Alain & Liogier 26347* (JBSD). **Samaná:** Samaná, en la zona Costera entre el km 4 & el km 5 E del pueblo de Las Terrenas, en el área de “protillo,” 3 m, *Zanoni & Mejia 17728* (NY). **San Cristóbal:** Nigua, *Faris 442* (US). **San Pedro de Macoris:** Town S of Boca de Soco, at SW bank of Río Soco at its mouth, small village along river and sea coast, 5 m, *Mejia & Zanoni 8595* (JBSD). **Santiago Rodríguez:** 20 km desde Sabaneta en la carretera a Monción, 250 m, *Zanoni & Pimentel 25441* (JBSD, MO, US).

**French Overseas Department. Guadeloupe.** Lamentin, Apres Duportail, en prenant le sentier du Chemin des Contrebandiers, 300 m, *Jeremie 295* (A, US). **Martinique:** Saint Martin, near Mullet Pond, *Boldingh 2764* (U).

**Grenada. St. George:** near Mount Parnassus, *Broadway 1720* (GH, NY).

**Haiti. Artibonite:** NE of Gros Morne, 235 m, *Leonard 9788* (US). **Nord:** vicinity of St. Michel del Atalaye, 350 m, *Leonard 7140* (NY, US). **Nord-Ouest:** vicinity of Port de Paix, *Leonard & Leonard 11181* (NY, S, UC, US). **Ouest:** herbarium at the Faculte D’Agronomie at Medicine Veterinaire at Damien, 310 m, *Paul & Porter-Utley AP516* (FLAS).

**Jamaica. Clarendon:** Peckham Woods, near Aenon Town, 2300 ft., *Crosby & Anderson 1233* (DUKE). **Hanover:** Lucea, *Hitchcock s.n.*, 3 January 1891 (MO). **Kingston:** vicinity of Kingston, *Maxon & Killip 338* (BM, GH, MO, US). **Manchester:** vicinity of Mandeville, *Crawford 742* (NY, PH). **Portland:** Navy Island, *Fredholm 3076* (NY, US). **St. Andrew:** 1.5 mi. SSW of Lucky Valley, along the road to Bull Bay, 403 ft., *Porter-Utley et al. P-57* (FLAS). **St. Ann:** Union Hill and vicinity, N slopes of Mount Diablo, 400–750 m, *Maxon 10398* (US). **St. Catherine:** Pigeon Island, 10 mi. off Old Harbour Bay, *Maxon & Killip 1580a* (US). **St. Elizabeth:** near pit 101, Kaiser mine area S of Gutters, *Howard & Proctor 13763* (A). **St. James:** near the mouth of Great River, W of Montego Bay, sea level, *Maxon & Killip 1426a* (US). **St. Thomas:** 14 mi. SE of Kingston toward Morant Point, *Wunderlin 5135* (MO). **Trelawny:** Quickstep Forestry Road, *Kay SQFR1* (FLAS). **Westmorland:** about 2 mi. W of White House, 0–100 m, *Yuncker 18026* (NY). **Unknown Parish:** *Ehb 32* (B).

**Netherlands Antilles. Bonaire:** Mont Kr, *Boldingh 7396* (U); *Stoffers 545* (U). **Curaçao:** near Sint Christoffelberg, *Curran & Haman 205* (CAS, GH, PH, US). **Sint Eustatius:** N rim of the Quill, 580 m, *Howard 18112* (A).

**Netherlands Autonomous Country. Aruba:** *Arnoldo 187* (U); *Boldingh 6515* (NY, U); *Stoffers 2036* (U).

**Puerto Rico. Bayamón:** Bo. Hato Tejas, series of mogotes W of Rt. 871 (only central pair collected), 25–100 m, *Axelrod & Axelrod 2346* (MO). **Cabo Rojo:** Salinas de Cabo, Rojo ad Punta de Aguila, *Urban 644* (BM, G, GH, M, PR, S). **Caja de Muertos:** Cayo Muertos, N.L. Britton, *Cowell & Brown 5030* (NY). **Ciales:** Bo. Hato Viejo, Rt. 6685, 1 km N jct Rt. 632, 50 m, *Axelrod & Axelrod 4430* (NY). **Dorado:** Mpio. de Dorado, Rte 693 at the freeway extension and on mogotes just E, *Taylor et al. 10050* (MO). **Guánica:** prope Guánica in litoralibus ad salinas, *Urban 3488* (BM, G, GH, M, MO, NY). **Isla Desecheo:** Desecheo Island, *Warshall 106* (GH). **Mayagüez:** Isla de Mona; Sardinera, lado E, *Acevedo & Siaca 4302* (NY). **Ponce:** Ponce to Peñuelas, *Britton et al. 1764* (NY). **Quebradillas:** Rt. 437 ca 1-2 km S of rte 113, 100-200 m, *Taylor & Gereau 10487* (MO). **Rincón:** Rincón, *Urban 5667* (G). **Toa Alta:** Rt. 677 ca. 3 km S of Rt. 2, 100 m, *Taylor & Miller 10409* (FLAS, MO). **Vega Alta:** Rte 620 km, 4.0 m, *Taylor & Molano 8681* (MO).

**Saint Lucia.** Vieux-Fort, Maria Island, *Pierre et al. 261* (A).

**Trinidad and Tobago. Trinidad:** Bird of Paradise Island, Yellowtail Walk, 60 m, *Webster 24186* (TRIN). **Tobago:** Banaan, *Broadway 4236* (M).

**United Kindom Overseas Territory. Anguilla:** Bottom district, N of The Valley, *Proctor 18538* (A, BM, US). **Bermuda:** Spittle Pond, *Brown 718* (GH, NY, PH, US). **Cayman Islands:** Grand Cayman, near larva survey site 80, trans island road, *Brunt 2164* (BM). **Paget:** Paget, *Harshberger s.n.*, 19 June 1905 (MO).

**United States Virgin Islands. St. Croix:** *A. Benzon 199-5098* (C); *Reugen 199* (C). **St. John:** Cruz Bay, Maria Bluff, 90 m, *Acevedo et al. 2330* (US). **St. Thomas:** *Orsted s.n.* (C).

**Mexico. Campeche:** Mpio. Hopelchén, 11 km S de la frontera Yucatán-Campeche, ca. de San Antonio Yax-che, *Carnevali et al. 5675* (CICY). **Quintana Roo:** Hwy. 307 between Chetumal and Cancún, 30 m, *Porter-Utley & Mondragón 393* (FLAS, CICY); road between Chetumal and Cancún, *Porter-Utley & Mondragón 398* (CICY). **Tamaulipas:** Gómez Farias, 3 km below city plaza off main road, *MacDougal 259* (DUKE, US). **Veracruz:** Mpio. Emiliano Zapata, 0.5 km de la desviación a Carrizal por la carretera Xalapa-Veracruz, *Calzada 1838* (F, XAL). **Yucatán:** off of Mexico 180 between X-can and X-Uilub, 60 m, *Porter-Utley & Mondragón 403* (CICY); road between X-can andX-Uilub, small path off main highway, *Porter-Utley & Mondragón 405* (CICY); on small dirt road off of the road between Vallodolid and Tulum, *Porter-Utley & Mondragón 407* (CICY); Mérida, CICY, Jardín Botánico, 20 m, *Porter-Utley & Mondragón 412* (CICY); Oxkutzcab, Labná, S de la entrada, 10 m, *Puch & Narvaez 488* (CICY, XAL); Oxkutzcab, Xul, camino antiguo a Benito Juárez 4 km, 60 m, *Sanabrio & Sima 194* (CICY).

**Belize. Belize:** Caye Caulker, N Island, *Whitefoord 8223* (BM, F, MA, MO). **Stann Creek:** Northeast Cay, Glover’s Reef, 0–3 m, *Fosberg & Sachet 53819* (B, BM, F, GH, MO, NY, US). **Toledo:** NE Sapodilla Cay, *Spellman & Stoddart 2322* (MO, US).

**Guatemala: Petén:** Dos Lagunas, Ixcanrío, on Aguas Turbias Road, *Contreras 8687* (F, LL, MO).

**Honduras. Islas de la Bahía:** Cayo Grande de Cayos Vivorillo, *Valerio 270* (MO, TEFH).

**Nicaragua. Zelaya:** Cayo Palmeta, 0–10 m, *Stevens & Krukoff 20764* (MO).

**Panamá. Panamá:** Bella Vista, at sea level, *Killip 12039* (US). **San Blas:** Soskatupu, island ca 1.5 mi. long, 0.5–0.7 mi. broad, 0–150 ft., *Elias 1692* (MO, UC).

**Colombia. Atlántico:** Usiacurí, Arroyo del Higuerón, 100 m, *Dugand & Garcia 2277* (US). **Bolívar:** vicinity of Turbaco, 200–300 m, *Killip & Smith 14329* (GH, US). **Magdalena:** Buritaca, 50 mi. E of Santa Marta, *Smith 1531* (NY). **San Andrés and Providencia:** San Andrés Island, along beach near Sound Bay Cemetary, *Weston & Weston 5542* (UC). **Sucre:** Mpio. Cartagena, Archipelago San Bernardo, Isla de Tintipán, Mar Caribe, 2 horas por bote NW de Tolú, *Callejas & Bornstein 11031* (HUA).

**Venezuela. Bolívar:** Carapo, *unknown collector 27* (PR). **Dependencias Federales:** Archipielago Los Testigos, Isla Testigo Grande, Playa Guzmán, Fernández José, *Flores-Javier & Fernández 607* (NY). **Falcón:** Dist. Silva, al pie de los penascos calcareos, S de la Punta Faustino, SE de Chichiriviche, 1–3 m, *Steyermark & Manara 110380* (MO, US). **Sucre:** vicinity of Cristóbal Colón, La Planisa, *Broadway 340* (US).

**Comoros. Anjouan:** *Schlieben 11161* (B, M, MO). **Gran Comore:** S edge of Moroni, *D’Arcy 17538* (MO). **Moheli:** *Schlieben 11248* (B, M, MO).

**French Overseas Possession.** Glorioso Islands, NW corner of Gloriosa Island, sea level, *Frazier 107b* (CONN, US).

**Madagascar.** Lemberano, *Hildebrandt 3264* (G, M).

**Republic of Seychelles. Aldabra Islands:** Mahé Island, Mahé, Pointe La Rue hill, 1200 ft., *Osborne-Day* 124 (BM).

**British Indian Ocean Territory.** Chagos Archipelago, Diego Garcia, *Hutson 27* (BM, US).

**India. West Bengal:** Calcutta, *Kuntze 6385* (NY).

**Maldives. Seenu:** Addu Atoll, Gan Island, S side of airstrip, *Sigee 55* (US).

**Mauritius. Agalega Islands:** S Island, *Stoddart 7263* (US). **Unknown District and Dependency:** Black River, *Tillich 3558* (MSB).

**Singapore.** Telok Paku, *Sinclair 6467* (US).

**Sri Lanka. Central:** campus of Univ. of Ceylon, Peradeniya, 500 m, *Comanor 324* (MO, US). **Sabaragamuwa:** 12th mi. post on the road between Panamure and Kilanne, Ratnapura Dist., *Balakrishnan & Jayasuriaya NBK911* (US). **Western:** Induruwa, *Jacobsen 13-6* (C).

**Australia. Queensland:** Rockhampton, *Boorman s.n.*, August 1912 (B).

**Commonwealth of the Northern Mariana Islands.** Saipan, E of Ogso Tafotchau just N of Kannat Tadung Laulau, 170–190 m, *Fosberg 50550* (US).

**Federated States of Micronesia. Pohnpei:** Ascension Island, 500 ft., *Saltis 385/5* (BM).

**French Overseas Territory. New Caledonia:** Sous-bois S sol calcaire, Îlot Maître pres Nouméa, *Guillaumin & Hurlimann 727* (NY, US).

**Palau. Koror:** *R. Bishop P-10192* (US).

**Solomon Islands. Guadalcanal:** Lunga, sea level, *Brown 1448* (BM).

**United States Territory. Guam:** Trust Territory Compound, NAS, Agana, 70 m, *Fosberg 46212* (BM, UC, US).

**Cultivated Material. United States:** North Carolina, cultivated at Duke University 1980-1984 from seeds sent by Jack Longino in 1979 from Florida, *MacDougal 662* (FLAS, MO); Florida, cultivated at the University of Florida from material collected at Passiflora Society International meeting, *Porter-Utley P-65* (FLAS).

#### 2. Passiflora suberosa

Taxon classificationPlantaeMalpighialesPassifloraceae

L. Sp. Pl. 958. 1753.

**Figure 25.**
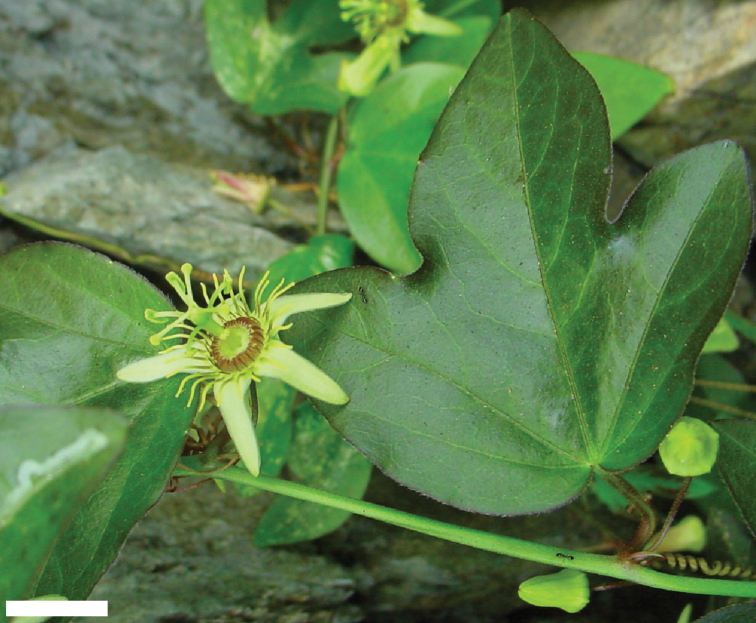
Flower of Passiflora suberosa subsp. suberosa (*Porter-Utley P-63*) from material collected by C. Feuillet in St. John. Scale bar = 10 mm. Photo by C. Feuillet.

**Figure 26.**
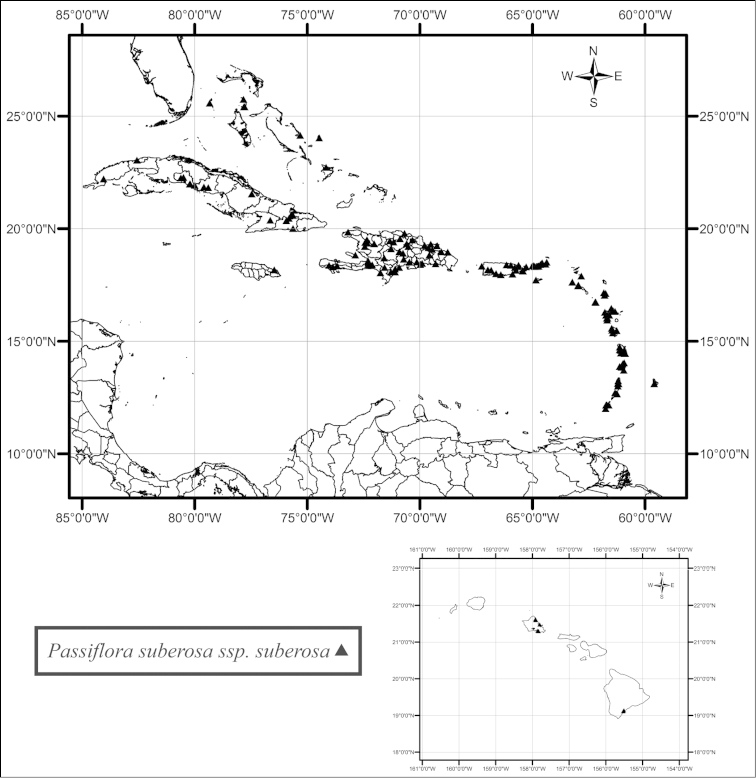
Distribution of Passiflora suberosa subsp. suberosa.

**Figure 27.**
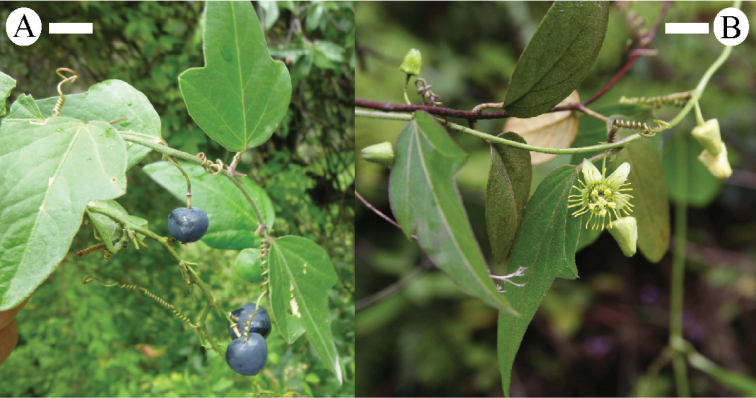
Flowers and fruits of Passiflora suberosa subsp. litoralis. **a** At edge of forest of *Cryptocarya*, *Ficus*, *Hypolepis*, *Rubus*, and *Melastoma taceae*, Jalisco, Mexico (*MacDougal 478*) Scale bar = 6.0 mm. Photo by J. M. MacDougal **b** From along a very dry roadside in Chiapas, Mexico (*Porter-Utley & Mondragón 456*) Scale bar = 6.0 mm.

**Figure 28.**
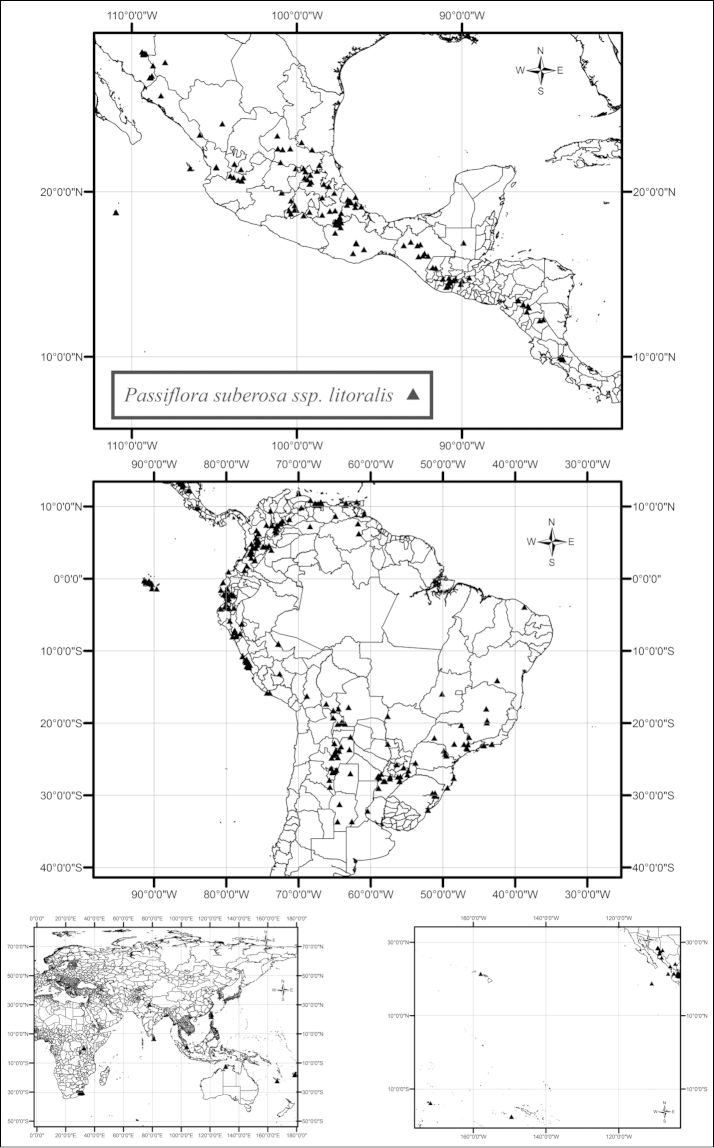
Distribution of Passiflora suberosa subsp. litoralis.

Figs 25 , 26 , 27 , 28

##### Description.

Slender, climbing, perennial vine 1–5(-10) m long or more, commonly sparsely to densely pubescent with unicellular or multicellular curved trichomes on leaf, petiole, stem, stipule, sepal, and tendril (very rare) (0.14-)0.20–1.13 mm long, 0.02–0.03 mm wide, also often minutely antrorsely appressed-puberulent on leaf, petiole, stem, stipule, and sepal with unicellular, curved trichomes, 0.05–0.10 mm long, 0.02–0.03 mm wide. Flowering stems 0.5–3.1 mm in diameter, terete or somewhat compressed, greenish yellow to reddish purple to red, with the base woody and cork-covered. Stipules (1.5-)2.2–8.4(-11.6)mm long, 0.1–1.3 mm wide, narrowly ovate-triangular, acute or rarely slightly attenuate; petioles 0.4–2.7(-3.7) cm long, with 2 (very rarely 1), opposite to alternate, stipitate or sessile, cupulate, discoid or capitate nectaries (very rarely urceolate), 0.4–1.5 mm wide, 0.2–1.6 mm high, commonly borne in the distal three quarters of the petiole (0.27–0.93 of the distance from the base toward the apex of the petiole). Laminas (1.4-)3.0–14.2(-19.0) cm long, (0.8-)1.6–10.0(-17.1) cm wide, not peltate or sometimes slightly peltate (the distance from leaf base to point of petiole insertion 2.3–2.5 mm), commonly membranous, 3-lobed, rarely unlobed, ovate, commonly with base cordate or cuneate to acute, lateral lobes (0.9-)1.4–7.5(-12.0) cm long, 0.3–3.0(-4.8) cm wide, ovate to oblong (very rarely obovate), acute (rarely obtuse or rounded), central lobe ovate to elliptic, sometimes obovate, central vein (1.4-)3.0–9.0(-14.2) cm long, angle between the lateral lobes (21-)40–140°, ratio of lateral lobe to central vein length (0.30-)0.38–0.87, margins entire, rarely crenate, hyaline, primary veins 1–3 (when more than one, veins diverge and branch at base or diverge and branch above base), laminar nectaries absent or sometimes with 1–10 submarginal nectaries associated with the minor veins of the abaxial surface, rarely associated with a crenation of the leaf margin, rarely with 2–4 nectaries proximal to the lateral leaf veins, 0.3–1.0 mm in diameter, circular to widely elliptic, sessile; tendril 0.2–1.1 mm wide, present at flowering node except in inflorescence. Flowers borne in leaf axils or sometimes in indeterminate axillary or terminal inflorescences; inflorescences 2.0–4.0 cm long, associated reduced laminas 2.0–4.3 mm long, 1.5–3.1 mm wide. Pedicels 2.3–17.9 mm long, 0.2–0.7 mm wide, 2 per node; bract(s) absent or rarely with one or two narrowly ovate-triangular bracts present at (0.23-)0.42–0.88 of the distance from the base toward the apex of the pedicel, 0.4–1.5(-2.3) mm long, 0.1–0.2 mm wide, acute; spur(s) absent. Flowers 12.3–49.1 mm in diameter with stipe (0.2-)1.4–11.5 mm long, 0.3–1.0 mm wide; hypanthium (3.0-)4.0–8.8 mm in diameter; sepals 4.0–14.6(-20.5) mm long, 2.0–5.0(-6.4) mm wide, ovate-triangular, acute to rounded, reflexed at anthesis, abaxially and adaxially greenish yellow to very light greenish yellow (5GY 7/4, 8/4–8/2) or white; coronal filaments in 2 series (very rarely 1 series), the outer 20–36, 2.5–8.1 mm long, 0.1–0.8 mm wide, linear, sometimes capitellate, erect (ca. 70°) or slightly spreading (ca. 110°) or spreading (ca. 180°-220°), greenish yellow with yellow tips (5Y 8/10), or flushed with reddish purple (5RP 5/6–3/6) at base and greenish yellow at middle with yellow tips, or reddish purple (5RP 3/8–4/8) at base, greenish yellow at middle and yellow toward tips, ratio of outer coronal row to sepal length 0.34–0.95, the inner (10-)18–45(-53), 1.5–3.9 mm long, 0.1–0.3 mm wide, linear, capitate, erect to slightly spreading, greenish yellow, or greenish yellow with yellow tips, or greenish yellow flushed with reddish purple at base and yellow toward tips, or reddish purple with greenish yellow tips, ratio of inner coronal row to outer coronal row length 0.21–0.76; operculum (0.7)1.0–3.0 mm long, plicate, greenish yellow, or greenish yellow with a flush of reddish purple at base, or reddish purple, margin white with minutely fimbrillate teeth; nectary 0.1–1.1 mm high, 0.3–1.8(-2.7) mm wide; limen recurved, erect or slightly inclined toward the operculum, 0.1–0.7 mm high, 0.1–0.6 mm wide, greenish yellow or greenish yellow with reddish purple tip, limen floor (1.3-)1.8–4.0 mm in diameter, greenish yellow or greenish yellow flushed with reddish purple; androgynophore (2.1-) 2.7–6.1(-12.6) mm long, 0.3–1.8 mm wide, greenish yellow or greenish yellow with a flush of reddish purple at base or greenish yellow with reddish purple spots and streaks; free portions of the staminal filaments 1.6–6.0(-6.8) mm long, 0.2–0.7 mm wide, linear, greenish yellow; anthers 1.4–3.3 mm long, 0.3–1.7 mm wide, pollen whitish or yellow; styles (1.7-)2.1–6.5(-7.7) mm long including stigmas, 0.1–0.5 mm wide, greenish yellow; stigmas 0.3–1.7 mm in diameter; ovary 1.2–4.1 mm long, 0.8–3.7 mm wide, ellipsoid to globose, greenish yellow. Berry 7.9–15.8 mm long, 7.4–13.4 mm in diameter, ovoid, ellipsoid or transversely ellipsoid, very dark purple (5P 2.5/2). Seeds ca. 8–34, 2.5–4.0 mm long, 1.5–2.5 mm wide, 1.0–1.8 mm thick, reticulate-foveate with each face marked with ca. 12–16 foveae, obovate in outline, acute at both ends, chalazal beak and micropyle inclined toward raphe; germination type epigeal.

##### Phenology.

Flowering and fruiting throughout the year.

##### Distribution.

In the New World tropics. Introduced in the Old World tropics. Growing in shrubs and trees or trailing on the ground in secondary successional areas, along the edges of semideciduous to deciduous, dry to wet tropical forests, both inland and near the seashore, 0–2500 m.

Passiflora suberosa subsp. suberosa and Passiflora suberosa subsp. litoralis have different geographic distributions, with Passiflora suberosa subsp. suberosa occurring in the Caribbean and Passiflora suberosa subsp. litoralis in Mexico, Central America, and South America. They only co-occur on the island of O’ahu, Hawaii, USA, where they have been introduced. The two subspecies are very similar vegetatively, but Passiflora suberosa subsp. litoralis is commonly conspicuously and densely pubescent with longer unicellular or multicellular curved trichomes, whereas Passiflora suberosa subsp. suberosa appears glabrous. Passiflora suberosa subsp. suberosa does not possess inflorescences present as condensed shoots with aborted lamina, but Passiflora suberosa subsp. litoralis may have them. The sepals of Passiflora suberosa subsp. suberosa are glabrous, and those of Passiflora suberosa subsp. litoralis are pubescent. The staminal filaments of Passiflora suberosa subsp. suberosa are often greater than 4 mm long, whereas those of Passiflora suberosa subsp. litoralis are less than 4 mm long. Passiflora suberosa subsp. suberosa also possesses a longer androgynophore (> 5 mm), and the androgynophore of Passiflora suberosa subsp. litoralis very rarely reaches a length of 5 mm. The fruits of Passiflora suberosa subsp. suberosa are larger (commonly > 1.0 cm) and usually ovoid, whereas Passiflora suberosa subsp. litoralis has depressed globose to globose to ellipsoid fruits that are commonly less that 1.0 cm long.

##### Key to the subspecies of *Passiflora suberosa*

**Table:** 

1a	Sepals white, 7.6–20.5 mm long, glabrous; androgynophore 5.0–12.6 mm long; outer coronal filaments reddish purple at base, greenish yellow at middle and yellow distally; inner coronal filaments reddish purple with yellow capitate heads; staminal filaments 3.4–6.8 mm long; pollen white; fruits ovoid	**2a. Passiflora suberosa subsp. suberosa**
1b	Sepals greenish yellow, 4.0–9.0(-10.8) mm long, pubescent with long, curved trichomes 0.16–1.13 mm long; androgynophore 2.1–4.4(-6.1) mm long; outer coronal filaments greenish yellow with yellow tips or greenish yellow with a flush of reddish purple at base and yellow at tips; inner coronal filaments greenish yellow, greenish yellow with yellow capitate heads, or greenish yellow with a flush of reddish purple at base and yellow capitate heads; staminal filaments 1.6–3.9 mm long; pollen yellow; fruits ellipsoid, transversely ellipsoid, or globose	**2b. Passiflora suberosa subsp. litoralis**

#### 2a. Passiflora suberosa subsp. suberosa

Taxon classificationPlantaeMalpighialesPassifloraceae

Figs 25 –26

Passiflora oliviformis Mill., Gard. Dict. ed. 8, no. 6. 1768. non *Passiflora oliviformis* Vell. [as “*olivaeformis*”], 1831. Type: Based on *Passiflora suberosa* L.
Passiflora angustifolia Sw., Prodr. [O.P. Swartz] 97. 1788. Type: Jamaica, *O. P. Swartz s.n.* (holotype: S, photograph seen [S-R-4073]; isotype: BM! [BM000563825]).
Passiflora hederifolia Lam., Encycl. 3(1): 38. 1789. Lectotype (designated here): Plumier, Desc. Pl. Amer. pl. 84. 1693.
Passiflora longifolia Lamarck, Encycl. 3(1): 40. 1789. Type: Hispaniola, *N. Desportes s.n.* (holotype: P-Juss [photograph seen] [P00307574]).
Passiflora peltata Cav., Decima diss. bot. 447 (pl. 274) 1790. Type: “Antilles”, *J. D. Surian 203* (holotype: P [in herb. Surian] [P00307395]).
Passiflora hederacea Cav., Decima diss. bot.: 448. 1790. Type: “Isles de la Martinique & de S. Domingue,” Plum. Desc. Pl. Amer. pl. 84. 1693.
Granadilla suberosa (L.) Gaertn., Fruct. Sem. Pl. 2(4): 480. 1791. Type: Based on *Passiflora suberosa* L.
Cieca heterophylla Moench, Suppl. Meth. 101. 1802. Type: Based on *Passiflora angustifolia* Sw.
Cieca suberosa (L.) Moench, Suppl. Meth. 102. 1802. Type: Based on *Passiflora suberosa* L.
Meioperis suberosa (L.) Raf., Fl. Tellur. 4: 103. 1838. Type: Based on *Passiflora suberosa* L.
Meioperis angustifolia (Sw.) Raf., Fl. Tellur. 4: 103. 1838. Type: Based on *Passiflora angustifolia* Sw.
Meioperis hederacea (Cav.) Raf., Fl. Tellur. 4: 103. 1838. Type: Based on *Passiflora hederacea* Cav.
Meioperis peltata (Cav.) Raf., Fl. Tellur. 4: 103. 1838. Type: Based on *Passiflora peltata* Cav.
Cieca angustifolia (Swartz) M.Roem., Fam. Nat. Syn. Mon. 2: 143. 1846. Type: Based on *Passiflora angustifolia* Sw.
Cieca hederacea (Cav.) M.Roem., Fam. Nat. Syn. Mon. 2: 141. 1846. Type: Based on *Passiflora hederacea* Cav.
Cieca peltata (Cav.) M. Roem., Fam. Nat. Syn. Mon. 2: 141. 1846. Type: Based on *Passiflora peltata* Cav.
Passiflora kohautiana C.Presl, Bot. Bemerk.: 72. 1844. Type: Martinique, *F. Kohaut s.n.* (holotype: PRC).
Passiflora suberosa var. hederacea (Cav.) Mast., Trans. Linn. Soc. London 27: 630. 1871. Type: Based on *Passiflora hederacea* Cav.
Passiflora suberosa var. angustifolia (Sw.) Mast., Trans. Linn. Soc. London 27: 630. 1871. Type: Based on *Passiflora angustifolia* Sw.
Passiflora calliaquatica Krause, Beih. Bot. Centralbl. 32(20): 340. 1914. Type: St. Vincent and the Grenadines: St. Vincent, between Kingstown and Calliagua, 25 January 1890, *H. Eggers 15718* (holotype: B! [B 10 0184893]).

##### Type.

“Habitat in Dominica, Antillis” [Dominica] (lectotype, designated by Wijnands 1983, pg. 171: LINN 1070.21 [microfiche seen]).

##### Description.

Sparsely to densely pubescent with unicellular or multicellular curved trichomes only on leaf, petiole and stem (very rarely on stipule) 0.14–0.62 mm long, 0.02–0.03 mm wide, also minutely antrorsely appressed-puberulent on leaf, petiole, stem, and stipule (sepal glabrous) with unicellular, curved trichomes, 0.06–0.10 mm long, 0.02–0.03 mm wide. Laminas not peltate or slightly peltate (the distance from leaf base to point of petiole insertion 2.3–2.5 mm). Flowers borne in leaf axils or sometimes in indeterminate axillary or terminal inflorescences; inflorescences 2.0–4.0 cm long, associated reduced laminas 2.0–4.0 mm long, 1.5–3.0 mm wide. Pedicels 6.9–17.6 mm long, 0.4–0.7 mm wide, 2 per node; bract(s) absent or with one or two narrowly ovate-triangular bracts present at (0.23-)0.42–0.66 of the distance from the base toward the apex of the pedicel, 0.4–1.5(-2.3) mm long, 0.1–0.2 mm wide, acute. Flowers 21.3–49.1 mm in diameter with stipe 3.3–11.5 mm long; hypanthium 5.5–8.8 mm in diameter; sepals 7.6–20.5 mm long, 2.9–6.4 mm wide, abaxially and adaxially white; coronal filaments in 2 series, the outer 3.7–8.1 mm long, linear, slightly spreading (ca. 110°), reddish purple (5RP 3/8–4/8) at base, greenish yellow (5GY 8/4–8/6) at middle and yellow (5Y 8/10) toward tips, ratio of outer coronal row to sepal length 0.34–0.74, the inner 10–30(-42), erect, reddish purple with greenish yellow tips; operculum 1.4–3.0 mm long, reddish purple, margin white; nectary 0.2–1.1 mm high, 0.7–1.8(-2.7) mm wide; limen recurved, greenish yellow with reddish purple tip, limen floor greenish yellow; androgynophore 5.0–12.6 mm long, 0.5–1.8 mm wide, greenish yellow; free portions of the staminal filaments 3.4–6.8 mm long, anthers with nearly white pollen; styles 3.2–7.7 mm long including stigmas. Berry 11.3–13.8 mm long, ovoid to ellipsoid. Seeds ca. 21–34.

##### Phenology.

Flowering and fruiting throughout the year.

##### Distribution.

Throughout the West Indies. Introduced in the Hawaiian Islands. Growing in shrubs, trees or trailing on the ground in secondary successional areas, along the edges of semideciduous to deciduous, dry to moist tropical forests, both inland and near the seashore, 0–1600 m.

##### Discussion.

In the Greater Antilles, Passiflora suberosa subsp. suberosa is commonly found in and along the edges of moist forests, primarily at higher elevations. It is relatively common on all of the islands of the Greater Antilles, except for Jamaica, where it is very rare. In the Lesser Antilles, it does occur at high elevations but primarily occurs at lower elevations and is found in dry to moist forests.

The vegetative morphology of Passiflora suberosa subsp. suberosa is incredibly variable. Nevertheless, throughout most of its range the subspecies commonly has trilobed leaves at reproductive nodes; only ca. 10% of the specimens examined have leaves that are unlobed at all nodes. Approximately 20% of the specimens possess unlobed, bilobed and trilobed leaves on sheets of the same collection. The leaves of Passiflora suberosa subsp. suberosa are commonly lobed less than 50% of the distance from the outline of the leaf to the leaf base and the lateral lobes are ½ - ¾ the length of the central lobe. The leaves are frequently dark green on their adaxial surfaces and have cordate bases. The juvenile leaves of Passiflora suberosa subsp. suberosa are often peltate and frequently possess laminar nectaries; however, the leaves on older plants are only very rarely peltate and usually do not have nectaries. The vegetative parts of the plant also possess varying amounts of reddish purple pigmentation, and the stems and new growth are often entirely reddish purple. Passiflora suberosa subsp. suberosa is relatively small in stature, rarely exceeding a length/height of five or six meters in the field. The flowers are more than 2.5 cm in diameter, with white sepals, coronal filaments that are dark reddish purple with yellow apices and whitish pollen. The fruits are usually ovoid and very dark purple.

In the Lesser Antilles, there are three morphological variants. One of these variants occurs in the Grenadines and has large leaves (over 10 cm wide) that are deeply trilobed (more than half the distance from the leaf outline to the leaf base) with long, commonly oblong lateral lobes that are at least three quarters the length of the lateral lobe. The leaves are often distinctly peltate and frequently possess four laminar nectaries (two on either side of the central leaf vein and one proximal to each lateral vein). Another variant occurs primarily in Dominica and Martinique and has deeply trilobed leaves with wider, ovate lateral lobes and deeply cordate bases. The leaves are not as large as the first variant (ca. 5–8 cm wide), but possess four laminar nectaries in the same positions as the entity in the Grenadines. The last variant occurs on several of the Windward Islands and has trilobed leaves with ca. 10 laminar nectaries. The nectaries are positioned near the leaf margin, creating crenations where they appear and are commonly positioned proximal to the lateral leaf veins, a very rare condition in the subspecies. All of these Lesser Antillean forms have the longest floral stipes and sepals in the subspecies. In the Dominican Republic and Cuba there is an additional variant that has unlobed leaves at all nodes. The leaves are exceptionally long for the subspecies (>10 cm), more coriaceous and possess petiolar nectaries that are wider and somewhat discoid, as opposed to the cupulate or capitate condition common in the subspecies.

Passiflora suberosa subsp. suberosa is sympatric with three species in supersection *Cieca*: *Passiflora pallida*, *Passiflora lancifolia*, and *Passiflora macfadyenii*. It can be easily separated from *Passiflora lancifolia* and *Passiflora macfadyenii* using both vegetative and reproductive characters. The most obvious features are that the leaves of *Passiflora macfadyenii* and *Passiflora lancifolia* are very densely pubescent with long, unicellular curved trichomes, whereas Passiflora suberosa subsp. suberosa appears glabrous (i.e., primarily microscopically antrorsely appressed-puberulent). The flowers of *Passiflora macfadyenii* and *Passiflora lancifolia* are also tubular and possess bright red sepals. Passiflora suberosa subsp. suberosa has the cup-shaped flowers typical of the supersection and white sepals. However, *Passiflora pallida* and Passiflora suberosa subsp. suberosa can be difficult to separate without reproductive material. The leaves of Passiflora suberosa subsp. suberosa are darker green in color than those of *Passiflora pallida* and sometimes have laminar nectaries, these strictly absent in *Passiflora pallida*. They are also wide, i.e., (2.9-)5.0–12.0(-17.1) cm, in Passiflora suberosa subsp. suberosa, and although this overlaps with the (0.3-)6.0–7.0(-10.6) cm range in *Passiflora pallida*, the character can frequently be used to distinguish between them. In addition, the leaf bases of Passiflora suberosa subsp. suberosa are cordate (when they are not peltate), whereas those of *Passiflora pallida* are very rarely cordate and usually are acute to cuneate. The stems, leaves (especially at their margins), tendrils, and stipules are frequently reddish purple in Passiflora suberosa subsp. suberosa, and the vegetative parts of *Passiflora pallida* generally possess little, if any, reddish purple coloration. *Passiflora pallida* may be densely pubescent where it occurs in the Caribbean, but Passiflora suberosa subsp. suberosa appears glabrous. The flowers of *Passiflora pallida* are much smaller than those of Passiflora suberosa subsp. suberosa. *Passiflora pallida* has sepals that are very rarely greater than 8 mm long, but the sepals of Passiflora suberosa subsp. suberosa are always longer than 8 mm. The hypanthium in *Passiflora pallida* is 2.2–4.2 mm wide, whereas that of Passiflora suberosa subsp. suberosa is 5.5–8.8 mm wide. *Passiflora pallida* has short staminal filaments (1.4–3.0 mm), and Passiflora suberosa subsp. suberosa has staminal filaments that are 3.4–6.8 mm long. The sepals of Passiflora suberosa subsp. suberosa are white, whereas those of *Passiflora pallida* are commonly greenish yellow; though *Passiflora pallida* may possess light colored sepals in the Yucatán Peninsula of Mexico. The fruits of these taxa are also quite different; Passiflora suberosa subsp. suberosa usually has ovoid fruits and *Passiflora pallida* has globose or ellipsoid fruits. In the Greater Antilles, Passiflora suberosa subsp. suberosa is commonly found at higher elevations and in more mesic habitats than *Passiflora pallida*. In other areas in the world their habitats are less distinct, but the species can be distinguished morphologically.

Clifford Smith in the Dep. of Botany at the University of Hawaii reports that Passiflora suberosa subsp. suberosa, as recognized here, is a minor weed in Hawaii in subcanopy layers where it smothers shrubs, small trees and the ground layer. In some areas it can also smother the upper canopy layer. He has also found that the seeds are dispersed by alien frugivorous birds.

##### Selected specimens examined.

**Antigua and Barbuda.** Antigua, Weatherills, *Box 1294* (MO).

**Bahamas. Acklins and Crooked Islands:** Crooked Island, road to Stopper Hill, *Brace 4810* (NY). **Bimini:** near center of Cat Cay, *Correll & Correll 45674* (GH, MO, NY). **Cat Island:** Gun Cay, *Millspaugh 2318* (NY). **Nichollstown and Berry Islands:** along Santa Maria Drive, Great Harbour Cay, *Correll & Correll 43707* (NY, TEX). **San Salvador Island:** near Museum, Sarant, *Saums & Warekois 57* (FLAS).

**Barbados.** upper Rusher Gully, 800 ft, *Blooding 128* (BM).

**British Virgin Islands.** Tortola, Harrigans, 300 m, *D’Arcy 253* (MO); Tortola, Slaney Point, *Fishlock 264* (NY).

**Cuba. Camagüey**, Sierra Cubitas, *Shafer 442* (NY, US). **Cienfuegos:** Trinidad Mountains, Santa Clara, Hanabanilla Falls, *Britton et al. 4857* (NY). **Granma:** Corojo, in “Pinarde Corojo”, ad viam (prope Bayamo ad. austr.-orient. versus), *Ekman 5045* (S). **Guantánamo:** vicinity of Baracoa, *Pollard et al. 249* (MIN, NY, US). **Holguín:** Sierra de Nipe prope Río Piloto in fruticetis, *Ekman 2696* (S). **Pinar del Río:** Limestone hills between Río Cayaguate and Sierra Guane, *Shafer 10474* (US). **Santiago de Cuba:** Bayate in sylvis prope Río Jagua, *Ekman 2025* (G, S). **Villa Clara:** San Blas-Buenos Aires, Trinidad Mountains, atop of Boma Ventana, *Howard 6507* (GH).

**Dominica. Saint David:** Carib trail from Salybia to Hatton Garden (1 mi.), *Hodge 3082* (GH, NY, US). **Saint George:** Deux Granges, 1000 ft., *Nicolson 2095* (DUKE, US). **Saint John:** N of Prince Rupert Bay, W Cabri, 50–190 m, *Smith 10323* (NY, US). **Saint Joseph:** Layou River Valley, Clarke Hall Estate, 400 ft, *Ernst 1265* (US). **Saint Peter:** 3 km S of DuBlanc on the coastal road to Roseau, 75 m, *Miller & Merello 8871* (MO).

**Dominican Republic. Azua:** cañada Miguel Martín between Sabana de Miguel Martín and Sabana de San Juan, 1500–1600 m, *Mejia & Zanoni 8250* (JBSD). **Baoruco:** Montiada Nueva, forested hillslopes SE of Polo, 3500 ft., *Howard & Howard 8509* (B, GH, NY, US); Río Baoruco from La Hortaliza (about 1.5 km up from mouth of Baoruco) to 2 km further upstream, 30–50 m, *Zanoni & Mejia 16485* (JBSD, MO, NY). **Dist. Nacional:** vicinity of Ciudad Trujillo, 0–25 m, *Allard 16370* (S, US). **El Seibo:** 0.5 km W of Sabana de Nisibón on hwy. to Miches, 15–20 m, *Mejia et al. 10130* (MO, NY). **Independencia:** aprox. 10.5 km al “S” de Puerto Escondido en la carretera a la Caseta No. 1 & la Caseta No. 2 de Foresta & Aceitillar, no lejos de la Caseta No. 1., 1240 m, *Zanoni et al. 34633* (JBSD). **La Vega:** Ruego la Devolución, Valle de Constanza, 1200 m, *Jiménez 1541* (US). **Peravia:** Arroyo de Parra, between Cerro de Quemada and Loma del Rancho, upstream from habitations of El Tamarindo, 800 m, *Mejia & Zanoni 8106* (NY). **Puerto Plata:** Puerto Plata, *Abbott 1469* (US). **Salcedo:** Cordillera Septentrional, 16 km N de Tenares, siguiendo la carretera hacia Gaspar Hernández; en el lugar llamado Boca Arriba, 550–600 m, *Garcia & Jiménez 4203* (JBSD). **Samaná:** Sección Las Galeras, Paraje Rincón, lugar denominado Laguna Salada, 10–20 m, *Peguero & Veloz 94* (JBSD). **San Cristóbal:** Gualupita (6 km N of Medina) which is 27 km N of main plaza of San Cristóbal on road to Medina and Madrigal, 100 m, *Mejia et al. 10379* (MO, NY). **San Juan:** Cordillera Central, Parque Nacional Ramírez, “La Lomita”, una loma cerca de la caseta del Parque Nacional de El Valle de Tetero, 1550 m, *Zanoni & Garcia 41471* (NY). **San Pedro De Macorís:** San Pedro de Macorís, *Rose et al. 4164* (NY, US). **Santiago:** Base Cordillera Central, Parcela 4 (de U. Klotz) del Compartimiento 493 del Proyecto Forestal La Celestina (W de San José de Las Matas) próximo al campamento del proyecto & la carretera a Rubio, *Klotz s.n.*, 1988–1990 (JBSD). **Santiago Rodríguez:** along Yaguajal River, 120 m, *Liogier 13235* (NY). **Unknown Province:** Haina, *Faris 121* (US).

**FRENCH OVERSEAS DEPARTMENT. Guadeloupe:** Basse Terre, Crete du Village, debut du chomin mesant aux 2 Mamelles, 700 m, *Sastre et al. 2566* (MO). **Martinique:** Anses d’ Arlets, Marm (Gommier) Case-Pilote, *Duss 873* (NY).

**GRENADA. Saint John:** Grand Anse, *Broadway s.n.*, December 1904 (GH, NY). **St. Mark:** Tufton Hall estate, 200–1000 ft., *Proctor 17151* (A, BM). **St. Patrick:** 0.5 mi. N of Tivoli, 100 ft., *Proctor 16888* (A, BM, US).

**HAITI. Artibonite:** vicinity of Kalacroix, Dessalines, 700 m, *Leonard 7952* (US). **Grand’ Anse:** Fonds Varettes, vicinity of Mission, 1000+ m, *Leonard 3610* (BM, GH, PH, US). **Nord:** St. Raphael Road 4 mi. E of St. Michel de l’Atalaye, 350 m, *Leonard 8521* (GH, US, NY). **Nord**-**Ouest:** vicinity of Jean Rabel, *Leonard & Leonard 12713* (NY, US). **Ouest:** Morne Boutellier, SE of Port-au-Prince, 3000 ft., just outside of Petionville in Duplan region, *Paul & Porter-Utley AP504* (FLAS). **Sud:** Massif de La Hotte, 13.6 km N de Camp Perrin en la carretera a Roseaux & Jérémie, “Tombeau Cheval”, 720 m, *Zanoni et al. 24320* (JBSD, MO). **Sud**-**Est:** Massif de la Selle, group Mornes des Commis-saires, Anse à Pitre, Banana, 200 m, *Ekman 6908* (S).

**JAMAICA.** See specimen listed under cultivated material.

**NETHERLANDS ANTILLES. Saba:** Windwardside, 500 m, *Arnoldo 3387* (U). **St. Eustatius:** Volcánic cone “The Quill”, 2–2.2 km E of Oranjestad, 200 m, *Iltis 30271* (WIS).

**PUERTO RICO. Barceloneta:** Bo. Garrochales, Rt. 22, km 57 (near jct Rt 140)., 50 m, *Axelrod et al. 8382* (MO). **Bayamón:** Cabra Island, San Juan, *Otero 108* (MO). **Carolina:** in NW urban Carolina, at Campo Rico Final and Fudalgo Díaz, *Taylor* 7835 (NY). **Culebra:** Culebra, Island of Culebra, *Britton & Wheller 80* (NY). **Fajardo:** Cabezas de San Juan Natural Reserve, along road that borders the lagoon, on the W side of the lagoon, near the reception house, sea level, *Ortiz-Zuazaga et al. 5* (US). **Humacao:** Cayo Santiago, Caribbean Primate Research Center, Big Key, side, 30 m, *Axelrod et al. 4158* (NY). **Juana Díaz:** Mpio. Juana Díaz, shore S of road 1 about 2 mi. E of road 149 W of Santa Isabel, SE of Juana Díaz, *Stimson 4031* (DUKE). **Luiza:** near Rd. 187 at Pinonez, *Houghton et al. 1234* (NY). **Maricao:** Maricao Forest Reserve, 20 mi. E of Mayagüez, 800 m, *Gentry & Zardini 50449* (MO). **Maunabo:** Punta de la Tuna, *Urban 5114* (BM, GH, NY). **Moca:** Bo. Rocha, Rt 112, km 13.0, 250-300 m, *Axelrod & Nir 8331* (MO). NAGUABO: junction of Rt. 31 & 3, *Evans 78* (A). **Ponce:** west on the Adjuntas road 10 mi. from Ponce, *Heller s. n* (NY). **Rincón:** Prope Rincón, *Urban 5668* (BM, GH). **Río Grande:** along Puerto Rico Rt. 185, on the W slopes of the Luquillo Mountains, *Pfeifer & class 2704* (CONN). **Vega Alta:** Bosque Estatal de Guajataca, Qiebradillos, *Kay 204* (MO). **Vieques:** vicinity of Isabel Segunda, *Shafer 2506* (CAS, NY). **Yauco:** Bo. Río Prieto, W slope of Monte Membrillo, along road above Hacienda Asunción, 850 m, *Axelrod & Axelrod 8546* (CICY).

**SAINT LUCIA. Gros-Islet:** Bois D’ orange near mouth of the river, 5 ft., *Slane & Boatman 251* (A). **Soufrière:** Colombette (Soufrière-Canaries Road), 1200 ft., *Box 1881* (BM). **Vieux Fort:** on the trail to the lighthouse from Vieux Fort, *Howard 11435* (A).

**SAINT VINCENT AND THE GRENADINES. Grenadines:** Carriacou, Grenada territory, *Howard 10859* (B, BM, GH, NY). **Saint Vincent:** cove on NW peninsula, Bequia, *Howard 11266* (GH).

**UNITED STATES VIRGIN ISLANDS. Saint Croix:** Eliras Retreat, *Eggers 437* (C). **Saint John.** Dirt road to Bordeaux Mountain, about 0.5 km from Center Line Road, *Acevedo 3133* (US). **Saint Thomas:** Fortuna Quarter, road 30, 140 m, *Acevedo et al. 5196* (US).

**UNITED KINGDOM OVERSEAS TERRITORY. Montserrat:** St. Peter, slopes of the Centre Hills, above Salem, 500–1000 ft., *Proctor 18884* (GH).

**UNITED STATES. Hawaii:** *Honolulu Co*.: O’ahu, on Hau’ula mountain rage, walking off the foot trail at the end of the jeep trail, around the ridge, 360 ft., *Herat & Wirawan 167* (B).

**CULTIVATED MATERIAL. United States:** Missouri, cultivated at the Missouri Botanical Garden and in J. M. MacDougal’s outdoor home garden 1987–1990 from seeds collected 26 Jan. 1987 in Jamaica, Portland Parish, *MacDougal 3026* (FLAS, MO); Florida, cultivated at the University of Florida from material collected by T. Zimmerman in St. Croix, *Porter-Utley P-4* (FLAS); Florida, cultivated at the University of Florida from material collected by C.Feuillet (*Feuillet 281*), *Porter-Utley P-63* (FLAS).

#### 2b. Passiflora suberosa L. subsp. litoralis

Taxon classificationPlantaeMalpighialesPassifloraceae

(Kunth) K.Port.-Utl. ex M.A.M.Azevedo, Baumbratz & Gonç.-Estev., Phytotaxa 53: 47. 2012.

Figs 27 –28

Passiflora litoralis Kunth, Nov. Gen. Sp. 2: 138. 1817. Type: Peru. Lima: [Pativilca],“Patibilca”, *A. Humboldt & A. Bonpland s.n.* (holotype: P [P00307301, photograph seen]; isotype: B, destroyed).
Passiflora limbata Ten., Index Seminum Horto Bot. Neapol. 10: 12. 1839. Type: Cultivated in Naples Botanical Garden, Italy (holotype: NAP [photograph seen]).
Passiflora pseudosuberosa Fisch., Index Sem. (St. Petersburg) 9: 82. 1843. Type: Cultivated in St. Petersburg (Russia), originally from Brazil, *Anon. s.n.* (type material not seen, probably at LE).
Passiflora oliviformis Vell., Fl. Flumin. 9: pl. 83. 1831, as “*olivaeformis*”. nom. superfl., non *Passiflora oliviformis* Mill. 1768. Type: Brazil (no specimens extant; lectotype, designated here: Vellozo, Fl. Flumin. 9: pl. 83. 1831.)
Passiflora globosa Vell., Fl. Flumin. 9: pl. 85. 1831. Type: Brazil (no specimens extant; lectotype, designated here: Vellozo, Fl. Flumin. 9: pl. 85. 1831.)
Passiflora flexuosa Gardn. London J. Bot. 1: 174. 1842. Type: Based on *Passiflora oliviformis* Vell.
Cieca oliviformis (Vell.) M.Roem., Fam. Nat. Syn. Mon. 2: 144. 1846. Type: Based on *Passiflora olivifornis* Vell.
Cieca globosa (Vell.) M.Roem., Fam. Nat. Syn. Monogr. 2: 144. 1846. Type: Based on *Passiflora globosa* Vell.
Cieca litoralis (Kunth) M.Roem., Fam. Nat. Syn. Mon. 2: 145. 1846, as “*littoralis*”. Type: Based on *Passiflora litoralis* Kunth
Cieca pseudosuberosa (Fisch.) M.Roem., Fam. Nat. Syn. Monogr. 2: 146. 1846. Type: Based on *Passiflora pseudosuberosa* Fisch.
Cieca limbata (Ten.) M.Roem., Fam. Nat. Syn. Monogr. 2: 148. 1846. Type: Based on *Passiflora limbata* Ten.
Cieca flexuosa (Gardn.) M.Roem., Fam. Nat. Syn. Monogr. 2: 148. 1846. Type: Based on *Passiflora flexuosa* Gardn.
Passiflora suberosa var. divaricata Griseb., Bonplandia (Hanover) 6 (1): 7. 1858. Type: Panamá, *E. Duchassaing s.n.* (holotype: GOET [photocopy seen] [GOET009402]).
Passiflora suberosa subvar. argentea Mast., Trans. Linn. Soc. London 27: 630. 1871. Lectotype (designated by Hemsley 1888, pg. 480): Mexico. Puebla: Tehuacán, *H. Galeotti 3663* (lectotype: K!; isolectotype: G!, [photocopies (2)], BR! [BR0000006943400]).
Passiflora suberosa var. longiloba Triana & Planch., Ann. Sci. Nat., Bot. 17: 157. 1873. Type: Colombia. “Tocayma”, *J. Goudot s.n.* (holotype: P [photograph seen]).
Passiflora suberosa var. longipes S. Watson. Proc. Amer. Acad. Arts 25: 149. 1890. Type: Mexico. Jalisco: in barranca near Guadalajara, 26 Sep 1889, *C. G. Pringle 2966* (holotype: GH! [GH00065788] [photographs, AAU!, DUKE!, F!]).

##### Type.

Based on *Passiflora litoralis* Kunth

##### Description.

Sparsely to densely pubescent with unicellular or multicellular curved trichomes on leaf, petiole, stipule, stem and sepal 0.16–1.13 mm long, 0.02–0.03 mm wide, also minutely antrorsely appressed-puberulent on leaf, petiole, stem, stipule and sepal with unicellular, curved trichomes, 0.05–0.10 mm long, 0.02–0.03 mm wide. Laminas not peltate. Flowers borne in leaf axils or sometimes in indeterminate axillary or terminal inflorescences; inflorescences 2.0–4.0 cm long, associated reduced laminas 2.0–4.3 mm long, 1.5–3.1 mm wide. Pedicels 2.3–17.9 mm long, 0.2–0.7 mm wide, 2 per node; bract(s) absent or rarely with 1 or 2 narrowly ovate-triangular bracts present at (0.23-)0.42–0.88 of the distance from the base toward the apex of the pedicel, 0.4–1.1(-2.3) mm long, 0.1–0.2 mm wide, acute. Flowers 12.3–26.1 mm in diameter with stipe 0.2–7.5(10.1) mm long; hypanthium (3.0-)4.0–6.3(-7.1) mm in diameter; sepals 4.0–9.0(-10.8) mm long, 2.0–5.5 mm wide, abaxially and adaxially greenish yellow to very light greenish yellow (5GY 7/4, 8/4–8/2); coronal filaments in 2 series (very rarely 1 series), the outer 2.5–7.5 mm long, linear, sometimes capitellate, erect (ca. 70°) or slightly spreading (ca. 110°) or spreading (ca. 180°-220°), greenish yellow with yellow tips (5Y 8/10) or flushed with reddish purple (5RP 5/6–3/6) at base and greenish yellow at middle with yellow tips, ratio of outer coronal row to sepal length 0.39–0.95, the inner (1-)20–50(-53), erect to spreading slightly, greenish yellow or greenish yellow with yellow tips or greenish yellow flushed with reddish purple at base and yellow toward tips; operculum 0.7–2.3 mm long, greenish yellow or greenish yellow with a flush of reddish purple at base or reddish purple, margin white; nectary 0.1–0.9 mm high, 0.3–1.3 mm wide; limen recurved, erect or slightly inclined toward the operculum, greenish yellow or greenish yellow with reddish purple tip, limen floor greenish yellow or greenish yellow flushed with reddish purple; androgynophore 2.1–4.4(-6.1) mm long, 0.3–1.3 mm wide, greenish yellow or greenish yellow with a flush of reddish purple at base or greenish yellow with reddish purple spots and streaks; free portions of the staminal filaments 1.6–3.9 mm long, anthers with yellow pollen; styles 1.7–4.7(-5.8) mm long including stigmas. Berry 7.9–11.9 mm long, depressed globose to globose to ellipsoid. Seeds ca. 8–34.

##### Phenology.

Flowering and fruiting throughout the year.

##### Distribution.

In the New World tropics: Central America, Mexico, Argentina, Bolivia, Brazil, Colombia, Ecuador, Paragual, Peru, and Venezuela. Introduced in the Old World tropics: Africa, Asia, Australia, and the Hawaiian Islands. Growing in shrubs, trees or trailing on the ground in secondary successional areas, along the edges of semideciduous to deciduous, dry to moist tropical forests, both inland and near the seashore, 0–2800 m.

##### Discussion.

Passiflora suberosa subsp. litoralis has the widest geographic range of any species in supersection *Cieca*. In the New World, its range extends from northern Mexico, through Central America, to central Argentina. In these areas it may be confused with *Passiflora pallida* and *Passiflora obtusifolia*, which are sometimes similar vegetatively. The similarities and differences between these two species are discussed under their respective descriptions. The primary difference between *Passiflora pallida* and Passiflora suberosa subsp. litoralis is the hypanthium diameter, with that of *Passiflora pallida* rarely exceeding a width of 4.0 mm and that of Passiflora suberosa subsp. litoralis commonly 4.0 mm or wider. One of the more useful characters employed in separating *Passiflora obtusifolia* and Passiflora suberosa subsp. litoralis is the presence/absence of inflorescences. When mature, *Passiflora obtusifolia* bears flowers in long inflorescences (i.e., 5.3–18.3 cm) and Passiflora suberosa subsp. litoralis almost always lacks inflorescences; when Passiflora suberosa subsp. litoralis does possess inflorescences they are not as long (i.e., 2.0–5.0 cm).

There are three major morphological variants of Passiflora suberosa subsp. litoralis. In Mexico and Central America, Passiflora suberosa subsp. litoralis possesses shallowly trilobed leaves (commonly less than half the distance from the leaf outline to the leaf base) with the length of the central lobe often greatly exceeding that of the lateral lobes and an angle between the lateral veins that is frequently between 40° and 80°. The lateral lobes are also oblong to elliptic. The broadly capitate petiolar nectaries are commonly positioned on the distal half of the petiole, often over 0.60 the distance from the base to the apex of the petiole. The leaf bases are often cuneate to acute but rarely cordate. In Mexico and Central America, Passiflora suberosa subsp. litoralis is often found in high elevation (1000–3000 m) moist pine and oak forests along streams and rivers, but it may also occur in very dry forests with cacti (e.g., *Cephalocereus* forests of Tehuacán) and other species common in matorral vegetation (e.g., Tamaulipan matorral).

On the western side of South America (Colombia to Peru and Argentina), Passiflora suberosa subsp. litoralis possesses leaves very much like those of the Mexico/Central American variant, but the petiolar nectaries may be more discoid and are commonly positioned on the proximal half of the petiole. The lateral lobes are commonly distinctly ovate and diverge at an angle of 80–100°. The leaf bases are also distinctly cordate. In this region it is found in low (near sea level and on cliffs above the sea) to high (to 3000 m) elevation moist forests commonly along streams and rivers, but it also occurs in tropical dry forests.

On the eastern side of South America, in southeastern Brazil, the leaves are commonly trilobed but may also have unlobed, bilobed or trilobed leaves present on the same plant. The petiolar nectaries are commonly discoid and positioned on the proximal half of the petiole. The lateral lobes are ovate, but longer than those common in western South America, and commonly diverge at an angle of greater than 100°. The leaf bases are distinctly cordate. In Brazil, Passiflora suberosa subsp. litoralis is more common in coastal dunes and tropical dry forests, but it does occasionally occur in higher elevation moist forests as well. This variant is the only form of Passiflora suberosa subsp. litoralis found in the Old World. Laminar nectaries are commonly present in all three of the these variants.

In a recent manuscript, Milward-de-Azevedo et al. (2012) incorrectly designated *Gardner 50* (BM) as a lectotype for *Passiflora flexuosa*. Gardner, in his manuscript (1842) was not publishing the new species, *Passiflora flexuosa*, but supplying a nomen novum for *Passiflora oliviformis* Vellozo. Therefore, the type of Gardner’s name is homotypic with *Passiflora oliviformis* Vellozo.

##### Selected specimens examined.

**MEXICO. Chiapas:** Barranca La Venta at Cascada El Aguacero, 16 km W of Oxozocuautla on Hwy. 190 and at the end of the road to the river, 650 m, Mayfield, *Hemple & Jack 977* (MEXU). **Chihuahua:** Guasaremos, Río Mayo, *Gentry 2910* (F, GH, MO). **Durango:** Sierra Madre, *Rose 3504* (US). **Guanajuato:** 5 km W de Iramuco, sobre el camino a Santa Ana Maya, 1950 m, *Rzedowski 44847* (CHAPA, XAL). **Guerrero:** Taxco, *Lyonnel 303* (US); close to mirador over Taxco on Mexico 95, 1790 m, *Porter-Utley & Mondragón 343* (CICY). **Hidalgo:** Trancas, 13 km NE de Zimapán, Mpio. de Zimapán, 2000 m, *Hernández 3695* (MEXU, MO). **Jalisco:** Guadalajara, Las Trancas, camino a Mascuala, Mpio. Ixtlahuacán del Río, 1600 m, *Guerrero & Chazaro 283* (MO, TEX). **México:** Cerro de los Capulines, Palmar Chico, 1100 m, *Matuda 31332* (US). **Michoacán:** Zitacuaro-Salto de Enandio, 1600 m, *Hinton 13492* (GH, LL, NA, NY, PH, TEX, US). **Morelos:** Xochitepec, *Lyonnel 1425* (US). **Nayarit:** W slope of Volcán San Juan at km 6 on road from Tepic to Jalcocotan, Mpio. Tepic, 900 m, *Breedlove & Almeda 45168* (CAS). **Oaxaca:** Mesa del Calvario, Cerro de el Ramón, NE de el Rodeo, Mpio. Tepelmeme, Dist. de Coixtlahuaca, 2100 m, *Tenorio et al. 9262* (US). **Puebla:** Jardín Botánico Helia Bravoh, road between Tehuacán and Zapotitlán Salinas., 1450 m, *Porter-Utley* & *Mondragón 344* (CICY); Tehuacán, *Purpus 1272* (G, MO, UC). **Querétaro:** Jalpan, 800 m, *Arguelles 909* (CAS); 4–5 km SE de Ayutla, Mpio. Arroyo Seco, 720–760 m, *Carranza 2819* (IEB). **San Luis Potosí:** 6.5 (rd) mi. S of Arista, in Chihuahuan Desert, on limestone hills, 5000 ft., *Henrickson 6428* (TEX); ca. 8 km NW de Guadalcázar, 2000 m, *Rzedowski 6673* (XAL). **Sinaloa:** 60 m W de la casa Ramón Cabrera, ejido Cuitavaca, a 35 km de Agua Caliente de Zevada, Mpio. Sinaloa de Leyva, *Perez 71* (CAS, UC). **Sonora:** Santa Ana de Yecora, 850 m, *Van Devender et al. 98-1420* (FLAS, MO). **Tamaulipas:** Las Yucas, along the road which bears W-SW from Village of Las Yucas for 2.2 miles, *Mayfield et al. 871* (TEX). **Veracruz:** Mpio. Emiliano Zapata, entre El Palmar & El Roble, *Castillo & Tapis 764* (Froad from Nautla to Tlapacoyan, *Porter-Utley & Mondragón 332* (CICY, FLAS); ); road from Nautla to Tlapacoyan, *Porter-Utley & Mondragón 333* (CICY, FLAS); Colonia Revolucion, Mpio. Boca del Río, 10 m, *Ventura 5394* (GH. MO, XAL). **Yucatan:** road between Chabihau and San Crisanto, *Porter-Utley & Mondragón 413* (CICY, FLAS); road between Chabihau and San Crisanto, *Porter-Utley & Mondragón 414* (CICY).

**COSTA RICA. Cartago:** Cartago, near the Mirador Ujarrás about 4.5 mi. SE of Paraíso, 1200 m, *MacDougal 906* (DUKE); vicinity of Cartago, *Standley 33363* (US). **Puntarenas:** Canton de Buenos Aires Rey Curre, camino a Sabana Mamey, *Rojas & Rojas 69* (MO). **San José:** San Francisco de Guadalupe, 1500 m, *Pittier 7151* (BR).

**EL SALVADOR. Ahuachapan:** San Benito, al N del mirador El Cerrito, *Sandoval & Roman1361* (MO).

**GUATEMALA. Baja Verapaz:** Mpio. San Jerónimo. km 137 carretera La Cumbre-Salamá, 1030 m, *Tenorio et al. 14839* (MEXU). **Chimaltenango:** between Chiquimula and La Laguna, 500–1000 m, *Steyermark 30703* (F). **Guatemala:** 20 km NW of Guatemala City, 5000 ft., *Andrews 541* (NY). **Hueheutenango:** vicinity of San Sebastián, 1600 m, *Molina & Molina 26507* (F, U); along Río Selegua, opposite San Sebastián H., 2000–2100 m, *Steyermark 50460* (F). **Jalapa:** Laguna de Ayarza, 8000 ft., *Heyde & Lux 3777* (GH, M, MO, NY, US). **Petén:** Santa Elena, *Walker 1344* (MO). **Sacatepéquez:** slopes of Volcán de Agua, N of Santa María de Jesús, 1800–2100 m, *Standley 59341* (F). **Sololá:** mountain slopes above Lake Atitlán, about 3–5 km W of Panajachel, 2100 m, *Williams et al. 25309* (F).

**HONDURAS. Comayagua:** Los Alpes on cordillera Montecillos, road to El Cedral, *Molina 23340* (F).

**NICARAGUA. Chontales:** along road from Juigalpa NE toward La Libertad, ca. 17.4 km NE of Río Mayales, at ford of Río Bizcocho, 350–400 m, *Stevens & Krudoff 4162-b* (MO). **Esteli:** Loma Ocotecalzado (Mesas Moropotente), ca 11 km NE of Hwy. 1 at Estelí, 1260–1300 m, *Stevens et al. 15609* (MO). **Jinotega:** along trail between Jinotega and Las Mesitas, W of Jinotega, 1100–1400 m, *Standley 9717* (F). **Madriz:** lado E cerro Volcán Somoto (Volcán Tepe Somoto), 1300 m, *Moreno 2949* (MO). **Matagalpa:** along road between San Simón de Palcila and Mesa La Cruz, 1150–1220 m, *Stevens et al. 18533* (G, HUA, MO). **Nueva Segovia:** La Tronquera, 660–700 m, *Moreno 19449* (MO).

**PANAMÁ. Unknown Province:** *Grisebach s.n.* (GOET). See specimen listed under cultivated material..

**ARGENTINA. Catamarca:** Sierra de Ancasti (Falda E), entre El Alto & el dique de Coyogasta, 950 m, *Hunziker & Cocucci 17284* (F, NY). **Chaco:** Fontana, *Meyer 2634* (F). **Corrientes:** Dep. Mburucuyá, 15 km NO de Mburucuyá, camino a Descabezado, *Krapovickas & Mroginski 22226* (G, MO). **Entre Ríos:** Dep. Diamante, Punta Gorda, *Bacigalupo & Deginani 16* (HUA). **Jujuy:** Dep. Santa Bárbara, NE of Libertador, ca. 20 km S of Palma Sola on the road to El Sauzal, 850 m, *Taylor et al. 11489* (MO). **Misiones:** Dep. Cainguás, Ruta Prov. 7, camino de Aristóbulo del Valle a Jardín América, 4 km de del Valle, 270 m, *Morrone et al. 629* (MO). **Salta:** Dep. Capital, 20 Km E de Salta, Ruta Salta a Gral. Güemes, *Krapovickas & Schinini 30408* (C, F, G, US). **Tucumán:** Dep. Alberdi/Cocha, along Ruta 9 from Juan B. Alberdi to Balcosna, 7 km below of dique Escaba, 670 m, *Till 10248* (MO).

**BOLIVIA. Chuquisaca:** Prov. Tomina, Monteagudo 64 km hacia Sucre, 1400 m, *Beck 6350* (MO). **Cochabamba:** Prov. Campero, a 26 km de Aiquile rumbo a Peña Colorada, 2240 m, *Saravia 522* (MO). **La Paz:** Viciniis Lorata, San Pedro, Larecaja, 2550 m, *Mandon 612* (BM, G, GH, NY, S). **Santa Cruz:** Prov. Cordillera, Camiri, 900 m, *Cardenas 4707* (US); Prov. Andrés Ibáñez, Jardín Botánico de Santa Cruz, 12 km E of center of Santa Cruz on road to Cotoca, 375 m, *Nee 40425* (NY). **Tarija:** Prov. Cercado, Bañado del Paray, 450 m, *Steinbach 13066* (F).

**BRAZIL. Bahia:** Mun. de Victoria da Conquista, 4.7 km south of center of city of Victoria da Conquista, along highway, *Eiten & Eiten 10892* (US). **Ceará:** Maranguape, Serra de Maranguape, *Trinta et al. 1280* (R). **Distrito Federal:** Santa Teresa, *unknown collector s.n.*, 1888 (R). **Goiás:** Goyaz, S le figau central de la province, *Glaziou 21461* (G). **Mato Grosso Do Sul:** Assentamento Tamarineiro, Mpio. Corumbá, *Pott 1812* (NY). **Minas Gerais:** Lema de Caldas, *Henschen & Regnelli III640* (MO, S). **Paraná:** Agua Branch (Mpio. Adrianópolis), 250 m, *Hatschbach & Silva 51299* (C, MO, US). **Pernambuco:** Tapera, *Pickel 465* (R). **Rio De Janeiro:** Climita Boa Vista, Rio de Janeiro, *Brade & Freire 24384* (R). **Rio Grande Do Sul:** Estrada Oscar Marcelino Cardoso próximo a Faz. Renato Johan, Banhado Grande, Viamão, *Abruzzi 814* (F). **Santa Catarina:** Mpio. Florianópolis, Canavieras, Ilha de Santa Catarina, 1–5 m, *Smith & Reitz 12264* (NY, R, US). **São Paulo:** Botucatu, Rubião Júnior, *Branzer 703501* (U).

**COLOMBIA. Antioquia:** Mpio. de Liborina, km 4 of road Liborina-Sabanalarga (32 km before Sabanalarga), 920 m, *Zarucchi et al. 7248* (HUA, MO). **Caldas:** al lado de la carretera entre La Felisa & Manizales, 1400 m, *Escobar & Brand 2059* (MA). **Cauca:** Cordillera Central, Vertiente Oriental, Mpio. de Inzá, Parque Arqueológico de San Andrés, 1700–2000 m, *Idrobo & Weber 1368* (US). **Cundinamarca:** Mpio. Apulo (Rafael Reyes), Vereda El Portillo, 9 km de Viotá, 455 m, *Escobar et al. 3002* (HUA). **Magdalena:** Sierra Nevada de Santa Marta, SE slopes, hoya del Río Donachui, below the village Donachui near the river, 1350–1230 m, *Cuatrecasas & Romero 24402* (US). **Norte De Santander:** between Chinácota and La Esmeralda, 1000–1300 m, *Killip & Smith 20887* (GH, US). **Quindío:** Mpio. Pijao, carretera a Caicedonia, 2 km antes de Barragán, Fca. Las Acacías, 1140 m, *Arbelaez et al. 970* (HUA). **Santander:** N slope of Mesa de los Santos, 1000–1500 m, *Killip & Smith 15029* (GH, NY, US); between Surata and California, *Killip & Smith 16834* (GH, NY, US). **Tolima:** Doima, 700 m, *Haught 2436* (US). **Valle De Cauca:** Cordillera Occidental, vertiente occidental, Hoya del Río San Quinini, 1200 m, *Cuatrecasas 15371* (F).

**ECUADOR. Chimborazo:** cañon of the Río Chanchán near Huigra, 4000–4500ft., *Camp 2945* (F, NY, US). **Esmeraldas:** Atacames, near Esmeraldas, *Barclay 764* (BM). **Galápagos:** Santa Cruz, near the Caseta, 800ft., *van der Werff 1420* (CAS, NY, U). **Guayas:** on the property of Richard Zeller near the village of Loma Alta, located about 10 km NE of the coastal village of Valdivia, N of Santa Elena peninsula, Río Valdivia drainage, 100 m, *Anderson 2480* (MO); Guayaquil, Cerro Santa Ana, *Asplund 15217* (AAU, B, NY, R, S). LA **Chorrera:** on Cerro Tiandeagote, 800 m, *Jativa & Epling 13* (UC). LOJA: Bosque Petrificado Puyango, quebrada Cochurco, 350 m, *Cornejo, Cornejo & Bonitaz 4034* (MO). **Los Ríos:** Hacienda Clementina between Babahoyo and Montalvo, 20 m, *Sparre 17922* (S). **Manabi:** P.N. Machalilla, Agua Blanca, hasta cerro Las Goteras, 380 m, *Josse 688* (AAU).

**PARAGUAY. Caaguazú:** Tavaí, 1 km S of Hospital, *Zardini 7744* (MO, TEX); Ruta 2, km 98, *Zardini & Aguayo 10551* (MO). **Concepción:** Arroyo Tagatiya-Misión, *Zardini & Tilleria 38858* (MO). **Guaira:** Mbocayaty-Melgarejo, 2 km E of Mbocayaty on Arroyo Gerbasia Gallery Forest, *Zardini & Tilleria 32349* (MO). **Neembucu:** Humaitá, *Schulz 7770* (F). **Paraguarí:** Parque Nacional Ybycuí, *Hahn 1951* (HUA, MO).

**PERÚ. Amazonas:** Prov. Chachapoyas. 9 km below and W of Chachapoyas on the road to Caclic, km 501, 2000 m, *Hutchison & Bennett 4514* (F, NY, UC, US). **Ancash:** Prov. Huacho, Sayán, road to Acobamba, 700–900 m, *Weigend & Dostert 97/116* (F, MSB). **Arequipa:** Prov. Caravelí, Lomas de Atiquipa (= km 591 Pananerica Sur), 150–750 m, *Weigend & Forther 97/922* (MSB). **Cajamarca:** Prov. Contumazá, road Contumazá to Chilete, 2500 m, *Weigend et al. 97/456* (MSB). **Callao:** Callao & Loma, *Didrichsen 4398* (C). **Cusco:** Prov. La Convención, 139 km de Cusco en Quellomayo, subiendo hacia la “ceja”, entre Santa Teresa & Chaullay, 1200–2600 m, *Nunez & Motocanchi 8782* (MO). **La Libertad:** Prov. Cajabamba, road from Cajabamba to Cajamarca, 8 km from Cajabamba, 2300 m, *Weigand et al. 97/318* (MSB). **Lima:** Prov. Lima, Loma de Amancae, S of Lima near Pachacamac, 120–410 m, *Gentry 16475* (AAU, MO, NY). **Piura:** Prov. Huancabamba, Procedencia, Porculla, km 38, 1800 m, *Quiroz 2349* (TEX). **Tumbes:** Prov. Zarumilla, Matapalo, El Cauco-Campo Verde parcela de evaluación permanente “E”, 700 m, *Díaz et al. 7426* (MO).

**VENEZUELA. Aragua:** Edos. Aragua & Miranda, bosque nublado de Loma de Hierro, 1350 m, *Colella & Morales 731* (VEN). **Bolívar:** in valley on road from El Valle to La Miranda, *Pittier 11970* (G, NY, US). **Distrito Federal:** on the old road from Caracas to La Guayra, between Bell Vista & Sanchorquiz, 1300–1450 m, *Pittier 9567* (GH, NY, US, VEN). **Falcón:** Cerro Santa Ana, ascensión del lado S desde el pueblo de Santa Ana, 600–700 m, *Steyermark & Braun 94621* (F, NY, US). **Lara:** al S de Quibor hacia Cubiro, Dist. Menez, 800 m, *Steyermark et al. 110061* (NY, VEN). **Mérida:** between Sabana Grande and Baruta, 1000 m, *Alston 5457* (BM); Colinas de Carrizal, 20 km de Caracas, *Morillo 2820* (NY). **Maracay:** SW del Valle de Caracas, Colinas de Bello Monto, Edo. Miranda, 1100 m, *Ramírez & Lopez 3273* (VEN). **Monagas:** Ladera S de Cerro San Bonifacio, en Bella Vista, 4 kms arriba del empalme con carretera Caripe-Teresén, 750–900 m, *Bunting 2645* (GH). **Sucre:** Los Cocoteros (Via Casanay), Dist. Ribero, *Cumana & Ceequea 4229* (WIS). **Tachira:** Sierra El Casadero, along hwy. between Las Dantas and Las Adjuntas, 850 m, *Steyermark et al. 120174* (MO, NY).

**SOUTH AFRICA. Kwazulu-Natal:** Colony, District Alesandra, Station Dumisa, 400 m, *Rudatis 1225* (BM); Denison Residence, Rutemaritburg, *Weigend 2190* (M).

**UGANDA. Kampala:** Kyadondo, Mengo, Kyambogo, 1200 m, *Rwaburindore 1735* (MO, US).

**INDIA. Uttar Pradesh:** Mothranwala, Dehra Døn, *Parker s.n.*, 27 November 1927 (UC).

**SRI LANKA. Uva:** road between Bandarawela and Haputale, just below Kahagalla tea factory, *Koyama et al. 16035* (AAU).

**INDONESIA. Java:** Cibodas, *Nitta 15054* (MO).

**SINGAPORE.** Nassim Road, *Togashi 6211611* (AAU).

**TAIWAN. Chia-I:** vicinity of Lianyun waterfall along the Tsengwen Hsi river, 300 m, *Bartholomew & Boufford 6176* (US). **P’ing-Tung:** Kenting National Park, Oluanpi park, 5 m, *Lammers 8488* (MO, US).

**AUSTRALIA. Northern Territory:** Nightcliff, Darwin, Arnhem Land Aboriginal Reserve, *Specht 160* (US).

**FIJI. Makondronga:** Makondronga Island, 60 m, *Degener & Orndonez 13801* (NY). NGAU: Shore of Herald Bay, in vicinity of Sawaieke, 0–30 m, *Smith 7923* (NY, UC, US). OVALAU: E of Lovoni Valley, 100–300 m, *Smith 7288* (NY, UC, US).

**FRENCH OVERSEAS TERRITORY. New Caledonia:** Au pied de l’Ouen Toro pres Nouméa, *Baumann 6059* (UC).

**FRENCH POLYNESIA. Society Islands:** Tahiti, Papeete, Crete est de la Tipaerui, sentier du Mt. Marau, 1200 m, *Florence 9735* (US).

**SAMOA.** Motootua, Upolu, *Whistler W5368* (BM).

**UNITED STATES. Hawaii:** *Honolulu Co*.: O’ahu, on steep ridges above ‘Aiea, *Iltis H-610* (US).

**SPAIN. Canarias:** Isle of La Palma. Santa Cruz, *Hausen 66* (C).

**CULTIVATED MATERIAL. United States:** Florida, grown in University of Florida Greenhouse (Gainesville, Alachua Co., Florida) from material collected in 1998 on an island off the coast of Panama, *Porter-Utley P-64* (FLAS); Florida, grown in University of Florida Greenhouse (Gainesville, Alachua Co., Florida) from material collected in Sonora Mexico by T.R. Van Devender et al. (*Van Devender et al. 98-1420*), *Porter-Utley P-58* (FLAS).

#### 3. Passiflora tridactylites

Taxon classificationPlantaeMalpighialesPassifloraceae

Hook.f,. Trans. Linn. Soc. London 20: 222. 1847.

Figs 29 –30

Passiflora lineariloba Hook.f., Trans. Linn. Soc. London 20: 222. 1847. Type: Ecuador. Galapagos: “Gallipagos, James Island” [Santiago], *J. Scouler s.n.* (lectotype, designated by Porter 1980, pg. 123: K [photocopy seen] [K000036556]).
Passiflora puberula Hook.f., Trans. Linn. Soc. London 20: 223. 1847. Type: Ecuador. Galapagos: “James Island” [Santiago], *C. Darwin s.n.* (lectotype designated by Porter 1980, pg 123: CGE [photocopy seen] [K000036541]; isolectotypes: CGE, K [photocopies seen]).
Passiflora suberosa var. lineariloba (Hook.f.) Mast., Fl. Bras. [Martius] 13(1): 579. 1872. Type: Based on *Passiflora lineariloba* Hook.f.

##### Type.

Ecuador. Galapagos: “Charles Island” [Floreana], Oct. 1835, *C. Darwin s.n.* (lectotype designated by Porter 1980, pg 123): CGE [photocopy seen]; isolectotype: K [photocopy seen] [K000036547]).

##### Description.

Slender, climbing, perennial vine to 2.5 m long or more, sparsely to densely pubescent with unicellular curved trichomes on petiole, leaf, and stem, 0.13–0.33 mm long, 0.02–0.03 mm wide, also minutely antrorsely appressed-puberulent on petiole, leaf, stem, stipule and sepal with unicellular, curved trichomes, 0.06–0.08 mm long, 0.02–0.03 mm wide. Flowering stems 0.5–1.3 mm in diameter, terete or somewhat compressed. Stipules 0.8–2.7(-3.6) mm long, 0.1–0.3 mm wide, narrowly ovate-triangular, acute; petioles 0.4–0.9(-1.7) cm long, with two, opposite to subopposite, sessile, discoid or widely obconical nectaries, 0.3–1.0 mm wide (on the widest axis), 0.1–0.5 mm high, commonly borne in the distal half of the petiole (0.44–0.86 of the distance from the base toward the apex of the petiole). Laminas 1.9–7.7 cm long, 1.8–7.9(-9.2) cm wide, membranous, shallowly to deeply 3-lobed, ovate in general outline, lateral lobes 1.0–5.5 cm long, 0.2–1.7 cm wide, ovate, elliptic, or very narrowly oblong (rarely obovate), acute (rarely obtuse), central lobe ovate, elliptic or very narrowly oblong (rarely obovate), acute (rarely obtuse), central vein 1.9–7.7 cm long, angle between the lateral lobes 92–129(-180)°, ratio of lateral lobe to central vein length 0.47–0.91, margins entire, hyaline, primary veins 3, diverging and branching at base, laminar nectaries absent (rarely present); tendril 0.2–0.5 mm wide, present at flowering node. Flowers borne in leaf axils. Pedicels 12.0–18.3 mm long, 0.3–0.5 mm wide, 2 per node; bract(s) absent; spur(s) absent. Flowers 23.9–33.3 mm in diameter with stipe (1.9-)3.3–5.3 mm long, 0.5–0.7 mm wide; hypanthium 4.6–7.1 mm in diameter; sepals 9.0–14.3 mm long, 2.0–4.3 mm wide, ovate-triangular, acute to rounded, sepals greenish yellow or whitish; coronal filaments in 2 series, the outer 21–30, 5.7–8.9 mm long, 0.1–0.5 mm wide, linear, not fused or fused 0.6–1.0 mm at base, filaments whitish with yellow tips or yellow, ratio of outer coronal row to sepal length 0.47–0.75(-0.89), the inner 19–30, 2.8–5.4(-6.4) mm long, 0.1–0.2 mm wide, linear, capitate, filaments whitish with yellow tips or yellow, ratio of inner coronal row to outer coronal row length 0.34–0.60(-0.94); operculum (1.5-)2.0–2.6 mm long, plicate, very pale yellow to yellowish dried, sometimes with reddish purple spots and streaks; nectary 0.2–0.5 mm high, 0.7–1.1 mm wide; limen recurved, (sometimes erect), 0.2–0.3(-0.6) mm high, 0.1–0.3 mm wide, yellowish or yellowish with a reddish purple base dried, limen floor 2.2–3.6 mm in diameter, yellowish or yellowish with reddish purple spots and streaks dried; androgynophore 8.0–10.8(-14.1) mm long, 0.6–1.0 mm wide, purplish; free portions of the staminal filaments 2.9–6.5 mm long, 0.3–0.5 mm wide, linear, yellowish dried; anthers 1.5–2.5 mm long, (0.3-)0.5–1.2 mm wide, oriented perpendicular or nearly so to their filaments; styles 3.4–5.0 mm long including stigmas, 0.2–0.4 mm wide, greenish yellow; stigmas 0.5–0.9 mm in diameter; ovary 2.8–5.3 mm long, 1.3–2.1(-2.9) mm wide, ellipsoid to fusiform, greenish. Berry 12.8–17.1(-21.1) mm long, 6.8–8.0(-10.0) mm in diameter, fusiform, very dark purple. Seeds ca. 20, 2.7–3.1 mm long, 1.5–1.8 mm wide, 1.2–1.4 mm thick, obovate in outline, acute at both ends, reticulate-foveate with each face marked with ca. 24 foveae.

**Figure 29.**
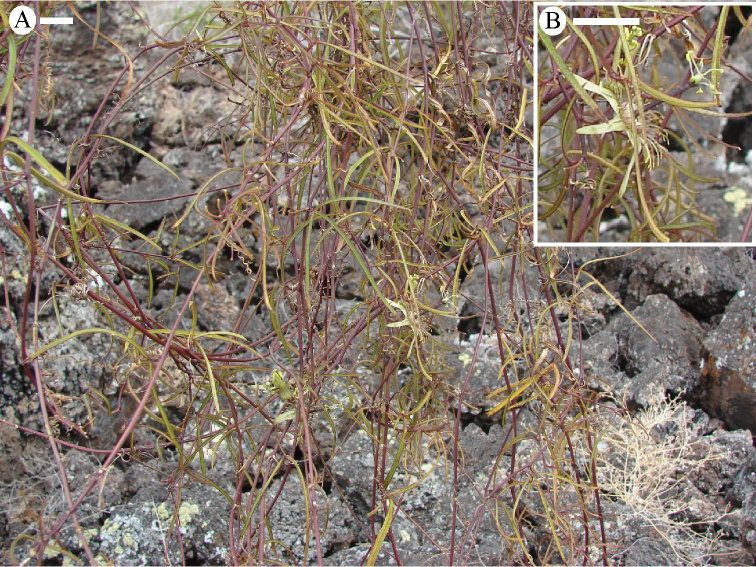
Flowers and leaves of *Passiflora tridactylites*. **a** View of whole plant. Scale bar = 10.0 mm **b** Enlargement of flower from the same photo. Scale bar = 10.0 mm. Photo by Walter Simbaña.

**Figure 30.**
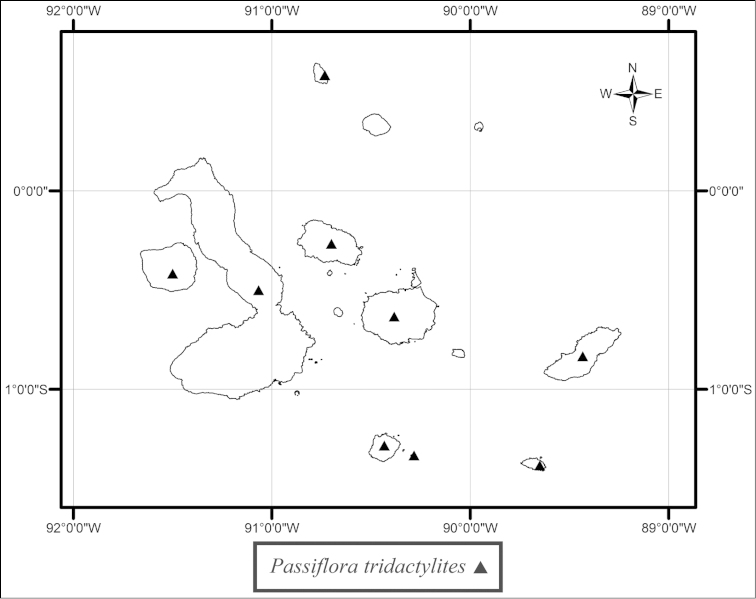
Distribution of *Passiflora tridactylites*.

##### Phenology.

Flowering and fruiting throughout the year.

##### Distribution.

Endemic to the Galapagos Islands. Growing in shrubs, trees or trailing on the ground in secondary successional areas and in dry tropical forests with *Castela*, *Scalesia*, *Psidium*, and *Bursera*, 0–800 m.

##### Discussion.

*Passiflora tridactylites* may be confused with Passiflora suberosa subsp. litoralis, which also occurs in the Galápagos Islands. Both species exhibit a great amount of variation in their vegetative morphology, with both species possessing all of the different vegetative forms described by Hooker, and I have not been able to find any vegetative characters that can reliably be used to distinguish between them. However, the flowers and fruits of these two species are quite different. The sepals of *Passiflora tridactylites* are commonly 10–14 mm long, whereas those of Passiflora suberosa subsp. litoralis do not exceed a length of 10 mm. The outer coronal filaments are long, more than 6.6 mm, in *Passiflora tridactylites*, and the filaments in Passiflora suberosa subsp. litoralis are commonly less than 6.0 mm long. The androgynophore in *Passiflora tridactylites* is diagnostically long, more than 8.0 mm, whereas that of Passiflora suberosa subsp. litoralis is always less than 6.0 mm. *Passiflora tridactylites* has long fusiform fruits, exceeding 12.8 mm. The fruits of Passiflora suberosa subsp. litoralis are 7.1–11.9 mm long and ellipsoid to globose. According to Lawesson (1988), the habitats of these two species are different, with *Passiflora tridactylites* occurring in dry lowland areas and Passiflora suberosa subsp. litoralis in mesic habitats. John MacDougal (pers. comm.) found abundant Lepidopteran scales on the inside of several flowers of pressed *Passiflora tridactylites* specimens, indicating visits by butterflies and/or moths and thus a probable shift in pollinators as a likely selective force leading to the clear floral differences in these two species. Van der Werff (*van der Werff 1951*) reported that finches eat the fruits of this species in the Galápagos.

*Passiflora tridactylites* was described by J. D. Hooker in 1851. At the time he actually described what he considered to be three distinct species on the Galapagos Islands: *Passiflora lineariloba*, *Passiflora tridactylites*, and *Passiflora puberula*. He based his descriptions primarily upon vegetative morphology. He described *Passiflora lineariloba* as a slender vine having deeply trilobed leaves with long, very narrow lateral lobes that are broadly diverging. Hooker apparently did not see the flowers of *Passiflora lineariloba* because he does not describe them and the type specimen is sterile. *Passiflora tridactylites* was described as having deeply trilobed leaves with subcordate bases and shorter, linear-oblong lateral lobes. Hooker described the flowers of this species as large (3/4 inch in diameter), with five linear, obtuse sepals with the ovary possessing a greatly elongated “pedicel” (androgynophore), and coronal filaments that are subequal to the sepals. *Passiflora puberula* was described as being covered in short, microscopic hairs and possessing trilobed leaves with cuneate bases and shorter, linear-lanceolate lateral lobes. Hooker goes on to describe the flowers, which possess five narrowly linear sepals that are pubescent, and fruits, which are ovate-oblong; though not mentioned in his description, the lectotype specimen of *Passiflora puberula* possesses a very long androgynophore. Lawesson (1988) differentiated between *Passiflora suberosa* and *Passiflora tridactylites*, but did not list the synonyms of either species in his treatment. Hooker based his description of *Passiflora tridactylites* on both vegetative and reproductive material with a detailed description of the flower and Lawesson (1988) used that name for the Galápagos entity, with *Passiflora lineariloba* and *Passiflora puberula* treated as synonyms. Though the type specimen of *Passiflora lineariloba* is sterile, vegetatively identical specimens with very large flowers and long androgynophores have been collected at the type locality. Thus, I have included it as a synonym of *Passiflora tridactylites* rather than Passiflora suberosa subsp. litoralis, which also occurs on the Galápagos Islands.

Killip (1938) lumped *Passiflora lineariloba*, *Passiflora tridactylites*, and *Passiflora puberula* with *Passiflora suberosa*. He noted that the entities on the Galápagos Islands with very narrow leaf lobes that had been labeled *Passiflora lineariloba* matched material collected by Safford and Mosier (*227*) from Florida. In addition, he noted that material similar to *Passiflora tridactylites* exactly matched specimens collected by Brown (*115*) in Jamaica. Based upon vegetative characters alone he is quite correct, but the flowers of these Galápagos specimens are distinctive. The specimens of Safford and Mosier and Brown are examples of *Passiflora pallida*, and the flowers and fruits of that species are far smaller than those of *Passiflora tridactylites*. Lawesson (1988) differentiated between *Passiflora tridactylites* and *Passiflora suberosa* stating that the species were easily separated by the shape and size of the sepals and the androgynophore length.

##### Specimens examined.

**Ecuador. Galápagos.** *Española*: Española, *Baur 160* (GH); “Gardner Island”, *Snodgrass & Heller 625* (GH); “Gardner Island”, *Snodgrass & Heller 321* (GH); Gardner Island, near Española, *Stewart 2075* (CAS, GH, MO, NY); Isla Española, landing site on N coast, beach area and area to El Chaco, *Lawesson 3126* (AAU). *Fernandina*: Isla Fernandina, SW slope of Narborough Island, 300 m, *Fosberg 45002* (CAS, K, MO); Fernandina, SW slope, in broad green strip running from summit to sea, 300 m, *Fosberg 45064* (CAS, K, MO). *Floreana*: Floreana, Andersson s.n., 1853 (AAU); Floreana, Andersson s.n. (AAU, S); Floreana, *Habel s.n.*, 1868 (K); September 1835 (K). *Isabela*: Isla Isabela, Volcán Alcedo, on the inner SW slope of the Caldera, 800 m, *Eliasson 1218* (S); Isla Isabela, Volcán Alcedo, SE part of the rim of the caldera, 1100 m, *Eliasson 1282* (S); Isla Isabela, W rim of Caldera of Alcedo, 3050 ft., *van der Werff 1951* (U). *Pinta*: Isla Pinta, S slope, 240–400 m, *Lawesson 2620* (AAU); Isla Pinta, first part of transect, 1–240 m, *Lawesson 2587* (AAU); Pinta, *Stewart 2079* (CAS, GH, US). *San Cristóbal*: San Cristóbal, Wreck Bay, 400-650 ft., *Stewart 2081* (CAS, GH); Isla San Cristobal, about 3.7 km above Puerto Bacqueriso (Wreck Bay) along road to El Progreso, *Wiggins & Porter 403* (CAS, GH, K, S). *San Salvador*: Isla San Salvador, James Bay, 20 ft., *van der Werff 1095* (AAU, CAS, K, U). *Santa Cruz*: Isla Santa Cruz, *Fagerlind & Wibon 3279* (S); Isla Santa Cruz, Academy Bay, 10 m, *Schimpff 52* (CAS); Isla Santa Cruz, 250 m, *Snow 470* (K); Santa Cruz, *Taylor TT126* (K). *Santiago*: Santiago, James Bay, 55 m, *Eliasson 1017* (AAU); Santiago, James Bay, 50 m, *Gradstein et al. V62* (U); Santiago, James Bay, *Howell 9665* (CAS, G).

#### 4. Passiflora lancifolia

Taxon classificationPlantaeMalpighialesPassifloraceae

Ham., Prod. Pl. Ind. Occ. [Hamilton] 48. 1825.

Figs 31 –32

Passiflora lanceolata Ham. ex G.Don, Gen. Hist. 3: 54. 1834, non *Passiflora lanceolata* Harms, 1894. Type: Based on *Passiflora lancifolia* Ham.
Decaloba lancifolia (Ham.) M.Roem., Fam. Nat. Syn. 2: 159. 1846. Type: Based on *Passiflora lancifolia* Ham.
Passiflora regalis Macfadyen ex Griseb., Fl. Brit. W. I. 292. 1860. Type: Jamaica. Saint Andrews: “Cold Spring Gap in S. Andrews, Port Royal”, *MacFadyen s.n.* (holotype: K!).

##### Type.

“Antilles”, *Anon. s.n.* ex Herb. Desvaux (holotype: P [P00605787, photograph seen] [photographs DUKE!, GH!, P!]; isotype: P [P00605788, photograph seen]).

##### Description.

Slender, climbing, perennial vine 3 m long or more, densely pubescent with unicellular curved trichomes throughout (except ovary), 0.5–1.4 mm long, 0.02–0.06 mm wide, also sparsely, antrorsely appressed-puberulent with unicellular, curved trichomes on stems, leaves and stipules, 0.03–0.05 mm long, 0.02 mm wide. Flowering stems 0.7–2.2 mm in diameter, subterete to terete, with the base somewhat cork-covered. Stipules 4.1–8.5 mm long, 0.3–0.9 mm wide; petioles 0.7–1.9 cm long, narrowly ovate, acute to attenuate, longitudinally striate-nerved, eglandular (rare) or commonly bearing in the distal third (0.69–0.97 of the distance from the base toward the apex of the petiole) (1-)2, round or elliptic, opposite to alternate, long-stipitate, cupulate nectaries, 0.1–0.5 mm wide, 0.4–1.2 mm high. Laminas 3.5–8.5 cm long, 1.5–5.2 cm wide, unlobed to shallowly 3-lobed 0.05–0.72 of the distance to the leaf base, when present, lateral lobes 1.1–4.0 cm long, 0.5–3.0 cm wide, elliptic, acute to rounded, central lobes 3.5–8.5 cm long, 1.0–3.5 cm wide, ovate to elliptic, acute to attenuate, angle between the lateral lobes 53–115°, ratio of lateral to central lobe length 0.29–0.56, margins entire, primary veins 1(rare) or 3, diverging and branching at base, laminar nectaries absent; tendril 0.3–0.6 mm wide, present at flowering node. Flowers borne in leaf axils. Pedicels 24.0–55.0 mm long, 0.3–0.8 mm wide; bract(s) absent or with one, narrowly ovate, acute bract, 0.9–1.8 mm long, 0.1–0.3 mm wide, the bract 20.6–34.8 mm from base of pedicel; spur(s) absent. Tubular flowers 7.1–12.8 mm in diameter with stipe 2.9–7.4 mm long, 0.5–1.0 mm wide; hypanthium 7.1–12.8 mm in diameter; sepals 20.1–31.8 mm long, 3.4–6.9 mm wide, narrowly ovate, acute, abaxially and adaxially reddish purple (5RP 4/6–4/8) dried; coronal filaments in 1 (rare) or 2 series, the outer 26–30, basally connate 1.1–3.8 mm, the free portions 5.8–10.3 mm long, 0.3–0.8 mm wide, linear to narrowly ovate, erect, reddish purple, lighter distally, ratio of coronal (fused and free portions) to sepal length 0.28–0.49, the inner not well-developed with 2–4 filaments or well-developed (rare) with 30–31 filaments, free or basally connate (rare) 0.8–0.2 mm, the free portions 1.1–2.9 mm long, 0.1–0.2 mm wide, linear, sometime capitellate, erect, appearing reddish purple when dried, ratio of inner coronal row to outer coronal row length (fused and free portions) 0.11–0.41; operculum 1.7–2.9 mm long, plicate, appearing light reddish purple dried, the margin with narrow minutely fimbrillate teeth; nectary 0.09–0.13 mm high, 1.1–3.5 mm wide, sulcate; limen slightly recurved to erect, occasionally slightly inclined toward operculum, 0.2–1.1 mm high, 0.1–0.3 mm wide, appearing light reddish purple (5RP6/6) dried, limen floor 2.1–6.1 mm in diameter, appearing light reddish purple dried; androgynophore 17.8–22.3 mm long, 0.6–1.3 mm wide, reddish purple dried; free portions of the staminal filaments 3.3–8.0 mm long, 0.3–0.7 mm wide, linear, greenish yellow; anthers 1.8–4.0 mm long, 0.5–2.0 mm wide; styles 4.3–7.0 mm long including stigmas, 0.1–0.4 mm wide, greenish yellow; stigmas 0.4–1.1 mm in diameter; ovary 2.6–6.7 mm long, 1.2–3.8 mm wide, elliptic, greenish yellow. Berry 12.8–13.9 mm long, 11.0–14.4 mm in diameter, ovoid to obovoid, very dark purple. Seeds ca. (6-)14–23, 3.0–3.2 mm long, 1.8–1.9 mm wide, 1.3 mm thick, obovate in outline, acute at both ends, reticulate-foveate with each face marked with ca. 15–17 foveae.

**Figure 31.**
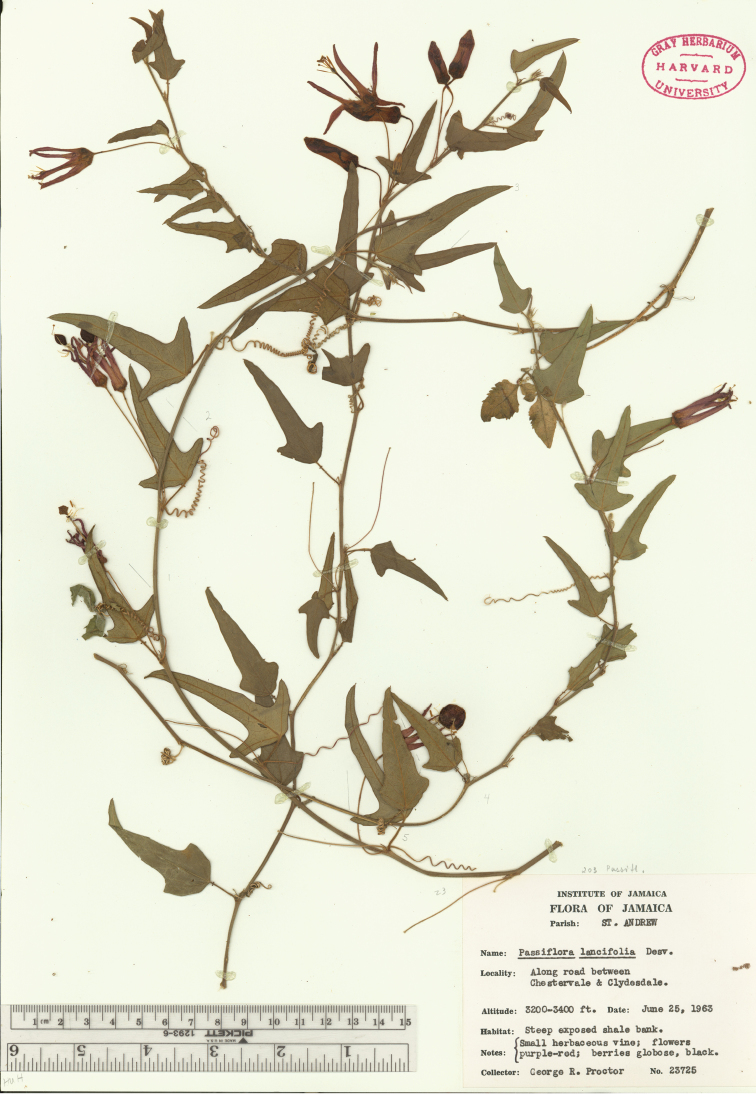
Herbarium specimen of *Passiflora lancifolia* (*G. Proctor 23725*).

**Figure 32.**
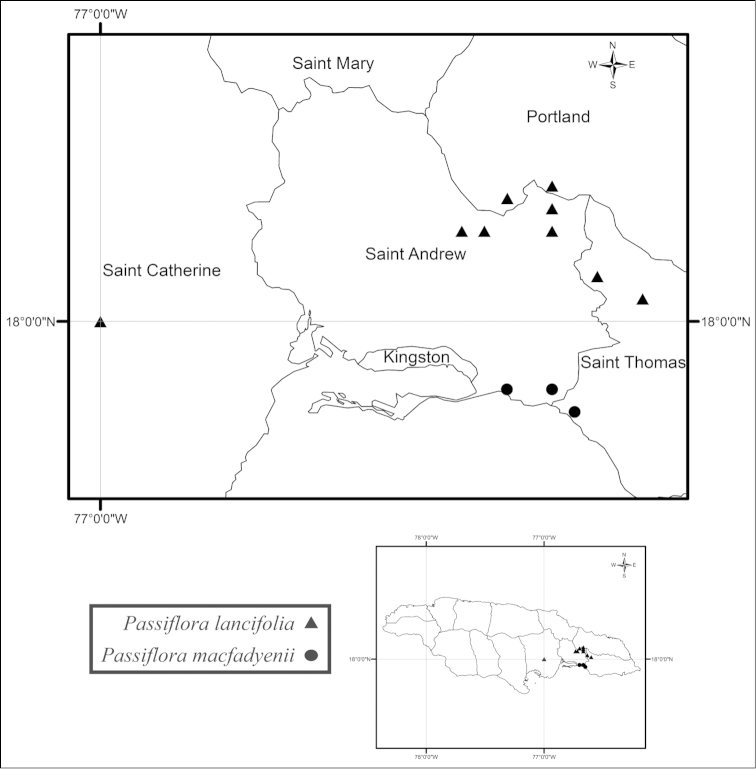
Distribution of *Passiflora lancifolia* and *Passiflora macfadyenii*.

##### Phenology.

Flowering and fruiting May to December.

##### Distribution.

Endemic to Jamaica, in the parishes of St. Andrew, St. Thomas, and Portland. Tropical lower montane mist forests on steep wooded hillsides and in thickets; growing on shrubs and trees; ca. 850–1220 m.

##### Discussion.

*Passiflora lancifolia* is very similar to another Jamaican endemic, *Passiflora macfadyenii*. They both possess bright red, elongated tubular flowers that are likely pollinated by hummingbirds. The two species can be easily separated utilizing both vegetative and reproductive characters. *Passiflora lancifolia* possesses shallowly trilobed leaves (rarely unlobed) with the lateral lobes commonly significantly less than half the length of the central lobe, and the central lobe is ovate and never narrowed at the base. *Passiflora macfadyenii* possesses distinctly trilobed leaves with the lateral lobes commonly more than half the length of the central lobe, and the central lobe is obovate with a distinctly narrowed base similar to that in *Passiflora juliana* and *Passiflora viridiflora*. The pedicels in *Passiflora lancifolia* are greater than 2.3 cm long, whereas those of *Passiflora macfadyenii* rarely exceed a length of 1.8 cm. The floral nectary of *Passiflora lancifolia* is the widest in the supersection, greatly exceeding that of *Passiflora macfadyenii*. The outer coronal filaments are connate and often not adnate to the sepals or barely so in *Passiflora lancifolia*, whereas those of *Passiflora macfadyenii* are distinctly adnate to the sepals. *Passiflora lancifolia* often has two rows of coronal filaments (rarely with one row or a poorly developed inner row) and *Passiflora macfadyenii* lacks an inner coronal row (or with a poorly developed second coronal row seen in one flower from a plant in cultivation, i.e., *MacDougal 452* - cultivated from cuttings of *Thomas 2032*). The fruits of *Passiflora lancifolia* and *Passiflora macfadyenii* are distinct, with *Passiflora lancifolia* having globose fruits and *Passiflora macfadyenii* possessing fusiform fruits. The habitats of the species are also different with *Passiflora lancifolia* growing in tropical lower montane mist forests at 850–1220 m and *Passiflora macfadyenii* found in tropical dry forests at 200–310 m.

The name *Passiflora lancifolia* was originally published by Hamilton as “*Passiflora lancifolia* Herb. Prof. Desv.,” and the species has often been cited as “*Passiflora lancifolia* Desv. in Ham.” or “*Passiflora lancifolia* Desv. ex Ham.” However, in the preface of his book, it appears that Hamilton himself took responsibility for the new species and genera described therein and only acknowledged the advice and assistance of Desvaux (see MacDougal and McVaugh 2001 for further details). Soon afterwards, Don (1834) described the taxon *Passiflora lanceolata*. However, Don’s description of *Passiflora lanceolata* is identical to that of *Passiflora lancifolia* in Hamilton and is based upon the same type material, therefore, the name *Passiflora lanceolata* G.Don is a nomenclatural synonym of *Passiflora lancifolia* Ham. In 1850, Macfadyen wrote his second volume of *Flora of Jamaica* and included in it the description of a different plant, which he called *Passiflora regalis*, now known as *Passiflora macfadyenii* C. D. Adams. However, Macfadyen unexpectedly passed away before the publication of his flora, though it was distributed. As a result, several authors viewed the new species that were described by Macfadyen as ineffectively published and began to publish new species based upon his work. Grisebach (1860) was one of these authors and published a description of *Passiflora regalis*, which he attributed to Macfadyen. However, the species that he described was *Passiflora lancifolia* and not Macfadyen’s *Passiflora regalis*. In addition, Ramírez Goyena (1909) published a description of *Passiflora regalis*, which he attributed to Macfadyen, but the species that he described was also *Passiflora lancifolia* and a later homonym of *Passiflora regalis* Macf. ex Griseb. Incidentally, Ramírez Goyena’s description of *Passiflora regalis*, other than being in Spanish and not in English, is virtually identical to that of Grisebach.

Killip (1938) placed *Passiflora lancifolia* together with *Passiflora viridiflora* in the subgenus *Chloropathanthus*. However, the discovery of *Passiflora juliana*, a species that very closely resembles *Passiflora viridiflora* but is clearly a member of supersection *Cieca*, reinforced MacDougal’s hypothesis (1983) that the apetalous, tubular-flowered species (including *Passiflora lancifolia*) belong in supersection *Cieca* (MacDougal 1983, 1992).

Benson et al. (1975), in a study of the coevolution of plants and herbivores, reported that *Dryas julia* is an herbivore of *Passiflora lancifolia*.

##### Specimens examined.

**JAMAICA. Portland:** Silver Hill Woodcutter’s Gap, 3500 ft., *Adams 11*, *936* (UCWI); Silver Hill, 3500 ft., *Harris 6536* (BM, UCWI); Silver Hill, Blue Mountains, 3000 ft., *Philipson 971* (BM); Buff Bay road west of Section, *Porter-Utley & Paul P-51* (FLAS); along the Buff Bay Road 0.5 mi. due W of Section, 3100 ft., *Proctor 22948* (GH, US). **St. Andrew:** Newcastle Rd., 2800 ft., *Adams 5723* (BM, UCWI); Newcastle to Hardwar Gap, 3700 ft., *Adams 8152* (BM); track Chestervale-Clydesdale, *Burrowes 13017* (UCWI); between Newcastle & Greenwich, *Hart 1440* (BM); along track between Bellevue & Mt. Rosanna, Port Royal Mts., 3800–4000 ft., *Proctor 23573* (GH); along road between Chestervale & Clydesdale, 3200–3400 ft., *Proctor 23725* (GH); road from Newcastle to Freewich, *RDR 1440* (UCWI); Fern Walk, Catherine’s Peak, 4000 ft., *Skelding 6788* (UCWI). **St. Thomas:** Farm Hill, *Orcutt 3437* (UC, US); Arntully, *Orcutt 3841* (UC, US); along track between Farm Hill and Whitfield Hall, 4000 ft., *Proctor 9659* (US); along the Stony Valley River near Arntully, 3000 ft., *Proctor 33513* (DUKE).

#### 5. Passiflora macfadyenii

Taxon classificationPlantaeMalpighialesPassifloraceae

C.D.Adams, Bull. Inst. Jam., Sci. Ser., 16: 27. 1967.

Figs 32 –33

##### Type.

Jamaica. St. Andrew: ca. 1.5 mi. SSE of Lucky Valley, 16 Dec 1956, *G. Proctor 15884* (holotype: IJ!; isotypes: GH! [GH00065787],MO! [MO-312538]).

##### Description.

Slender, climbing, perennial vine 3 m long or more, densely pubescent with unicellular curved trichomes throughout, 0.2–0.7 mm long, 0.02–0.03 mm wide, also minutely antrorsely appressed-puberulent throughout with unicellular, curved trichomes, 0.08–0.10 mm long, 0.02 mm wide. Flowering stems 0.9–2.1 mm in diameter, somewhat compressed, base somewhat woody and cork-covered. Stipules 2.0–8.0 mm long, 0.3–1.1 mm wide, linear-narrowly ovate, acute to attenuate, longitudinally striate-nerved; petioles 0.4–1.5(-3.7) cm long, commonly bearing in the distal half (0.54–0.83 of the distance from the base toward the apex of the petiole) (1-)2, round or elliptic, opposite to alternate, sessile (rare) or stipitate, cupulate nectaries, 0.3–0.6 mm wide (on the widest axis), 0.3–1.0 mm high. Laminas 1.4–9.0 cm long, 1.6–6.4(-11.9) cm wide, deeply 3-lobed 0.21–0.93 of the distance to the leaf base, lateral lobes (0.8-)2.0–4.2(-7.3) cm long, (0.1-)0.6–1.8(-2.3) cm wide, oblong to obovate, acute to rounded (rarely emarginate), central lobes 1.4–5.2 (-9.0) cm long, (0.2-)0.5–3.0 cm wide, elliptic to obovate, acute to rounded (rarely emarginate), often narrowed at base, angle between the lateral lobes 79–134°, ratio of lateral to central lobe lengths 0.60–0.96, margins entire, primary veins 3, diverging and branching at base, laminar nectaries absent; tendril 0.3–0.7 mm wide, present at flowering node. Flowers borne in leaf axils. Pedicels 11.0–18.0(-23.0) mm long, 0.4–0.8 mm wide; bract(s) absent; spur(s) absent. Tubular flowers 5.5–8.1 mm in diameter with stipe 1.5–6.5 mm long, 0.4–0.9 mm wide; hypanthium 5.5–8.1 mm in diameter; sepals 19.3–26.1 mm long, basally connate 7.1–12.5 mm, 1.3–3.1 mm wide, linear to narrowly ovate, acute to rounded, abaxially and adaxially red (ca. 5R 6/10), free portions of sepals reflexed at anthesis; coronal filaments in 1 series, adnate to the calyx tube, 25–30, the free portions 2.0–5.7 mm long, 0.1–0.3 mm wide, linear to narrowly ovate, erect, appearing red with yellow apices when dried, ratio of coronal (portion not adnate to sepal) to sepal (free portion) 0.25–0.44; rarely a trace second coronal row of filaments may be present just outside the operculum; operculum 1.4–2.0 mm long, plicate, appearing red when dried, the margin with narrow minutely fimbrillate teeth; nectary 0.1–0.5 mm high, 0.7–2.5 mm wide, sulcate; limen slightly recurved to erect, 0.1–0.7 mm high, 0.1–0.5 mm wide, red when dried, limen floor 2.9–5.0 mm in diameter, red when dried; androgynophore 17.8–23.5 mm long, 0.8–1.1 mm wide, red when dried gradually getting lighter distally or with the red coloration nearly reaching the apices of the staminal filaments; free portions of the staminal filaments 5.4–8.0 mm long, 0.3–0.6 mm wide, linear, greenish yellow or red; anthers 2.8–3.5 mm long, 0.7–2.0 mm wide; styles 4.2–5.5 mm long including stigmas, 0.1–0.3 mm wide, greenish yellow; stigmas 0.73–1.33 mm in diameter; ovary 3.6–8.0 mm long, 1.0–2.7 mm wide, fusiform, greenish yellow. Berry 25.0–26.0 mm long, 5.9–9.0 mm in diameter, ellipsoid and tapering at both ends (fusiform), very dark purple. Seeds ca. 20, 3.1–3.7 mm long, 1.6–1.8 mm wide, 1.2–1.3 mm thick, obovate in outline, acute at both ends, reticulate-foveate with each face marked with 15–17 foveae.

**Figure 33.**
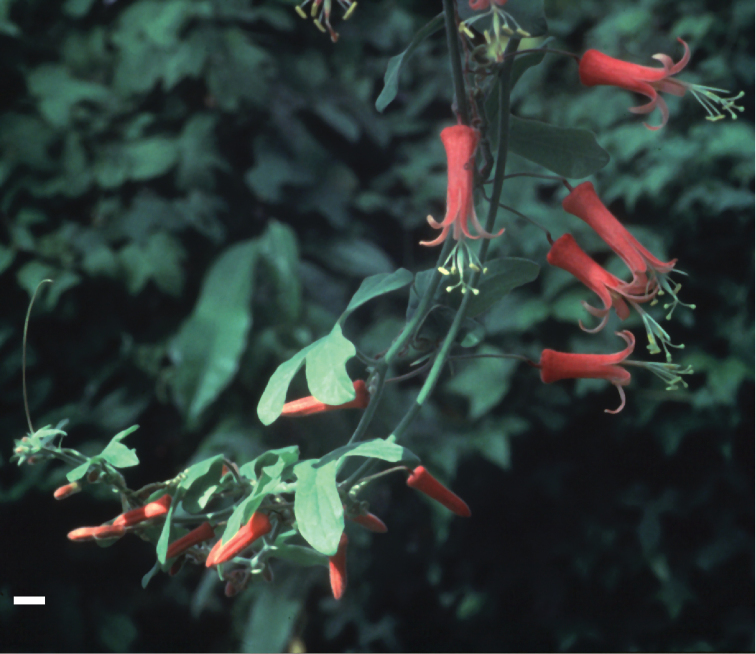
Leaves and flowers of *Passiflora macfadyenii* (*MacDougal 452*) Scale bar = 6.0 mm. Photo by J. M. MacDougal.

##### Phenology.

Flowering and fruiting from December to February, sometimes flowering in June.

##### Distribution.

Endemic to Jamaica, in the parishes of St. Andrew and St. Thomas. Tropical dry forests in roadside thickets and wooded limestone hills near Lucky Valley (St. Andrew) and Cambridge Hill (St. Thomas); growing on shrubs, small trees, limestone boulders and rocks on very limited to moderately developed soils; ca. 200–310 m.

##### Discussion.

As mentioned under *Passiflora lancifolia*, *Passiflora macfadyenii* is somewhat similar to that taxon but differs from it in characters of the leaf, flower, and fruit. Both species are quite distinct and can be easily separated in the field and herbarium. It is interesting that the leaf shape of *Passiflora macfadyenii* is very similar to that of *Passiflora juliana* and *Passiflora viridiflora*. Killip (1938), under his description of *Passiflora lancifolia*, also noticed their vegetative similarities.

*Passiflora macfadyenii* is very restricted in its distribution and has only been collected in the vicinity of Lucky Valley in the dry tropical forests of the Port Royal Mountains, St. Andrew, Jamaica. I visited this area in June of 2000, but the region was experiencing a severe drought and four days of searching for the plant revealed neither vegetative nor reproductive material. Elma Kay (St. Louis University and Missouri Botanical Garden) and George Proctor (University of the West Indies and the Institute of Jamaica) have also made several trips to the area and have not been able to find *Passiflora macfadyenii*. It was last collected in 1979 (*Thomas 2032*, *2034*) and was listed as a rare plant in the 1997 IUCN Red List of Threatened Plants. It is my opinion that its status should be upgraded to extinct/endangered. It is fortunate that MacDougal obtained cuttings of *Passiflora macfadyenii* from Thomas (*MacDougal 452*, *Thomas 2032*) and grew the plant in the greenhouses at Duke University from 1979–1982; it is no longer in cultivation. Thanks to their efforts we have a better understanding of the biology of this very rare taxon.

In an unpublished manuscript, MacDougal determined the total sugar concentration measured as sucrose equivalents in percent weight per total weight to be 29–44% in *Passiflora macfadyenii*. He also found the flower to have no odor. The flower shape and morphology, combined with these data, indicate that *Passiflora macfadyenii* is (or was) likely utilized by hummingbirds.

*Passiflora macfadyenii* was described by Adams as a new species in 1967, and he discussed the differences between it and *Passiflora lancifolia* and some of the taxonomic confusion associated with these species. As mentioned under *Passiflora lancifolia*, Macfadyen described the plant now known as *Passiflora macfadyenii* as *Passiflora regalis* in his *Flora of Jamaica* in 1850. Shortly afterwards, Grisebach (1860) and Ramírez Goyena (1909) incorrectly applied the name *Passiflora regalis* to another similar but distinct taxon, *Passiflora lancifolia*. Fawcett and Rendle, in 1926, did attempt to rectify this situation and published a description of Macfadyen’s true *Passiflora regalis*, which they attributed to him. However, *Passiflora regalis* Macf. ex Fawc. & Rend. is an illegitimate name because it is a later homonym of *Passiflora regalis* Macf. ex Griseb and *Passiflora regalis* Macf. ex Ramírez Goyena. Therefore, Adams gave Macfadyen’s true *Passiflora regalis* a new name, *Passiflora macfadyenii*, and designated a new type specimen.

##### Specimens examined.

**JAMAICA. St. Andrew:** Newstead, 500 ft., *Adams 8976* (UCWI);1.5 mi. SSW of Lucky Valley, along road between Bull Bay & Cane River Falls, 700 ft. *Proctor 16172* (BM); 1.5 mi. SSE of Lucky Valley, 700 ft. *Proctor 24913* (BM, US); 2 mi. N of Bullbay on road to Cane River Falls, *Thomas 2032* (DUKE). **St. Thomas:** Cambridge Hill, 1000 ft., *Adams 10232* (BM, DUKE, UCWI). **Parish Unknown:** Plato Road, *Harris s.n.*, 5 October 1897 (UCWI).

**CULTIVATED MATERIAL. United States of America:** North Carolina, Durham, Duke University, cultivated from material collected by Thomas (*2032*), *MacDougal 452* (FLAS).

#### 6. Passiflora tenuiloba

Taxon classificationPlantaeMalpighialesPassifloraceae

Engelm., Boston J. Nat. Hist. 6: 192. 1850.

Figs 9 , 34 –35

Passiflora bigelovii Small, Bull. N. York Bot. Gard. 1: 283. 1899. Type: United States of America. Texas: “Camp Green”, *C. C. Parry [Mexican Boundary Survey] 393c* (lectotype, designated here: NY! [NY00110395]; isolectotype: GH! [00065785])

##### Type.

United States of America. Texas: “Western Texas, On the Liano”, 1869, *F. Lindheimer s.n.* (holotype: MO! [MO-312539]).

##### Description.

Slender, low-climbing or scrambling, perennial vine 1 m long or more, densely pubescent with unicellular curved trichomes on petiole, and adaxial leaf surface, 0.21–0.38 mm long, 0.02 mm wide, also minutely antrorsely appressed-puberulent throughout with unicellular, curved trichomes, 0.01–0.29 mm long, 0.02–0.07 mm wide. Flowering stems 0.5–1.4 mm in diameter, terete, base somewhat woody and cork-covered. Stipules, 1.9–3.6 mm long, 0.2–0.5 mm wide, narrowly ovate, acute to attenuate, longitudinally striate-nerved; petioles 0.2–1.1 cm long, commonly bearing in the distal half, (0.36-)0.52–0.81 of the distance from the base toward the apex of the petiole, 2, elliptic, opposite, sessile, cup-shaped nectaries with raised rims, 0.8–2.2 mm wide (on the widest axis), 0.2–1.3 mm high. Laminas 0.3–3.7 cm long, 3.0–14.8 cm wide, coriaceous, occasionally variegated as juveniles, 3- to 5-lobed 0.37–0.90 of the distance to the leaf base at the deepest sinus, lateral lobes 0.3–7.0 cm long, 0.1–0.6 cm wide, linear to narrowly ovate, acute to attenuate, often the primary lateral lobes with 1 to 4 smaller lobes, central lobes 0.3–3.7 cm long, 0.1–3.0 cm wide, ovate to oblong, acute to obtuse, often with 2 to 3 smaller lobes toward apex, angle between the lateral lobes 145–343°, ratio of lateral to central lobe lengths 0.58–23.33, margins entire, hyaline, primary veins 3 to 5, diverging and branching at base, laminar nectaries absent or with one submarginal nectary associated with the minor veins of the abaxial surface, 0.6–0.9 mm in diameter, circular to widely elliptic, sessile; tendril 0.1–0.5 mm wide, present at flowering node. Flowers borne in leaf axils. Pedicels 1.3–8.5 mm long, 0.4–0.6 mm wide, paired in the leaf axils; bract(s) absent or rarely with one narrowly ovate, attenuate, bract present on the distal tip of the pedicel, ca. 0.8 mm long, 0.3 mm wide; spur(s) absent. Flowers 12.8–20.6 mm in diameter with stipe 1.1–4.1 mm long, 0.6–0.8 mm wide; hypanthium 4.3–5.9 mm in diameter; sepals 3.9–8.1 mm long, 1.7–4.3 mm wide, ovate-triangular, acute to rounded, abaxially and adaxially greenish yellow; coronal filaments in 2 series, the outer 35–47, 2.7–4.9 mm long, 0.2–0.5 mm wide, linear, tapering to a point or slightly capitellate, reflexed above middle and the tips often slightly incurved, greenish yellow toward the base and yellow toward the tip or reddish purple (5RP 3/4) at the base and yellow toward the tip, ratio of outer coronal row to sepal length 0.44–0.90, the inner 35–50, 1.6–3.1 mm long, 0.1–0.3 mm wide, linear, capitate, greenish yellow with yellow tips or reddish purple with yellow tips, erect, ratio of inner coronal row to outer coronal row length 0.46–0.68; operculum 0.9–1.3 mm long, plicate, greenish yellow with yellow margin or reddish purple with yellow margin, the margin with narrow minutely fimbrillate teeth; nectary 0.1–1.0 mm high, 0.4–0.7 mm wide, slightly sulcate; limen recurved, 0.1–0.7 mm high, 0.2–1.1 mm wide, greenish yellow with a white margin or reddish purple with a white margin, limen floor 1.1–2.9 mm in diameter, greenish yellow or greenish yellow with reddish purple spots and streaks; androgynophore 2.9–4.2 mm long, 0.8–1.2 mm wide, greenish yellow or greenish yellow with reddish purple spots and streaks; free portions of the staminal filaments 1.9–3.6 mm long, 0.3–0.7 mm wide, linear, greenish yellow; anthers 1.7–2.9 mm long, 0.5–1.9 mm wide; styles 2.5–4.0 mm long including stigmas, 0.2–0.5 mm wide, greenish yellow; stigmas 0.6–1.0 mm in diameter; ovary 1.0–2.6 mm long, 0.9–2.4 mm wide, globose to slightly obovoid, greenish yellow. Berry 7.1–14.6 mm long, 7.3–15.3 mm in diameter, ovoid to obovoid, very dark purple. Seeds 12–25, 4.1–4.8 mm long, 1.9–2.5 mm wide, 1.3–1.5 mm thick, obovate in outline, acute at both ends, reticulate-foveate with each face marked with ca. 17–25 foveae.

**Figure 34.**
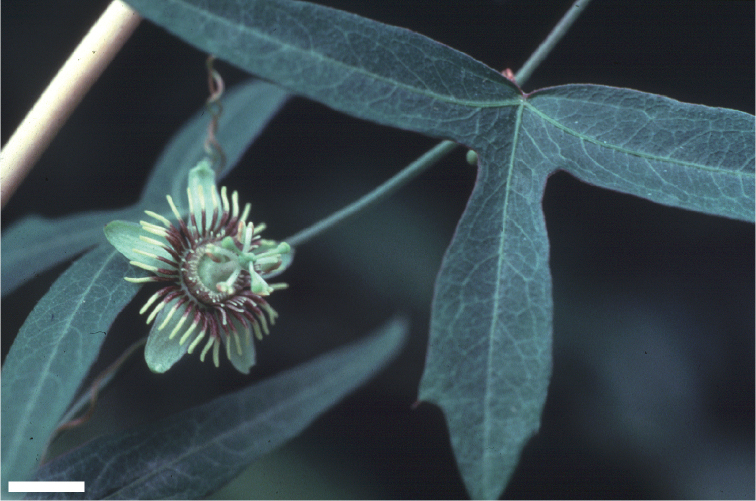
Flower and leaf of *Passiflora tenuiloba* (*MacDougal 227*) Scale bar = 6.0 mm. Photo by J. M. MacDougal.

**Figure 35.**
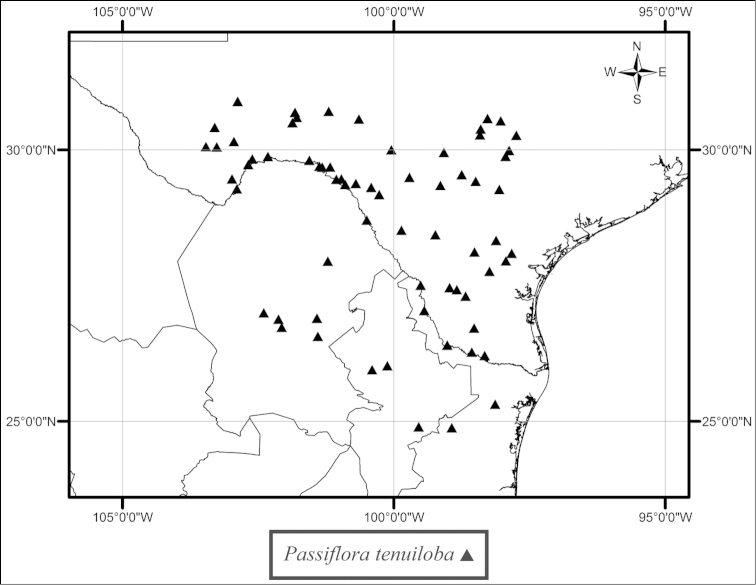
Distribution of *Passiflora tenuiloba*.

##### Phenology.

Flowering and fruiting March to December.

##### Distribution.

Northern Mexico and southern Texas in the United States. Arid and semiarid thorn scrub (e.g., Mesquite-Black brush, *Opuntia*-*Prosopis* scrub, Tamaulipan thorn scrub) and grasslands; climbing on shrubs or scrambling on limestone outcrops and hills, or in open grassy areas on very limited to moderately developed soils; ca. 150–1500 m.

##### Discussion.

*Passiflora tenuiloba* is very distinctive in the form of the leaves. It possesses leaves that are shallowly to deeply 3- to 5- lobed, often with lateral lobes that are up to 8.0 cm long and between 0.2 and 2.1 cm wide. The lateral lobes frequently possess 2–3 lobes at their apices. The central lobe is short (<1.0 cm) or longer (to 3.7 cm), sometimes with three lobes at its apex. The petiolar glands are positioned on the distal half of the petiole, often at the petiole apex or even on the base of the leaf. *Passiflora tenuiloba* also has very distinctive seeds with reticulate centers and grooved edges.

*Passiflora tenuiloba* occurs in southwest Texas and northern Mexico along with *Passiflora pallida*. The small flowers of these two species are somewhat similar, but they can be easily separated by vegetative characters. The most obvious difference is the shape of the lamina, with *Passiflora tenuiloba* possessing leaves that are transversely elliptic and *Passiflora pallida* possessing leaves that are ovate to elliptic in general outline. In addition, the flowers of *Passiflora tenuiloba* have a wider hypanthium than those of *Passiflora pallida* and have more and commonly longer filaments in their coronal rows. The seeds of *Passiflora tenuiloba* are 4.1–5.8 mm long, whereas those of *Passiflora pallida* do not exceed a length of 3.5 mm.

*Passiflora tenuiloba* has been included in three other studies of passionflowers. Benson et al. (1975) found that *Agraulis vanillae* (Gulf Fritillary) is an herbivore of this species. Klucking (1992) found that the leaf venation pattern of this species is similar to *Passiflora sexocellata* and *Passiflora eglandulosa* and was classified as actinodromous and pinnate secondary venation with irregular to regular intercostal venation consisting of lineate and transverse veins. According to Klucking, the leaves of *Passiflora tenuiloba* are more like those of *Passiflora eglandulosa*, because they have acute lateral lobes, an angle between the lateral veins that is between 120 and 140°, and leaf bases that are cordate.

Engelmann, in his description of *Passiflora tenuiloba*, states that the type specimen was collected in October “on the Liano” (likely meaning “on the llano”) by Lindheimer and that only a single specimen was collected (Goldman 2004). The specimen, which is clearly labeled as being collected “on the Liano” and possesses a Latin description of *Passiflora tenuiloba* from Engelmann, is held at the Missouri Botanical Garden (MO). There is another specimen collected by Lindheimer at MO, but it is not type material. The form described as *Passiflora bigelovii* possesses central leaf lobes that are longer and nearly equal in length to the lateral lobes. Small (1899) cites three specimens in his description of *Passiflora bigelovii*, but did not designate a holotype. Killip (1938) listed *Parry 393c* as the type of *Passiflora bigelovii*, but did not officially designate it as a lectotype or discuss the other specimens (syntypes) cited by Small. I have selected *Parry 393c* as the lectotype for *Passiflora bigelovii*, as the specimen is the most complete with leaves, flowers, and fruits.

##### Selected specimens examined.

**UNITED STATES. Texas:** *Bandera Co*.: N side of F.M. 470 ca. 300 ft. W of road-summit in Seco Pass, 9.3 mi. E of F.M. 187, Seco Pass Quadrangle, *Carr 9090* (TEX). *Bexar Co*.: on Austin Chalk plain at mouth of Government Canyon, E side of current main entrance road, W side of abandoned entrance road, 0.6 mi. N of gate at 90° curve on Galm Rd., ca 5.0 mi. (by air) WSW of jct. SR 16 and Loop 1604, 29°32'35"N, 98°44'54"W, 980–990 ft., *Carr 14560b* (TEX). *Blanco Co*.: *Tharp 203* (TEX). *Brewster Co*.: 3 mi. from mouth of Heath Canyon, *Correll 31586* (LL). *Brown Co*.: Dallas, *Reverchon s.n.*, 12 August 1877 (NY). *Burnet Co*.: Marble Falls, *Carsuer & Studhalter 4338* (TEX). *Crockett Co*.: 22 mi. W of Ozona on U.S. 290, *Flyr 5* (TEX). *Dimmit Co*.: Chaparral Wildlife Management Area, *Gilbert s.n.*, 10 May 1979 (TEX). *Duval Co*.: Texas hwy. 359, 6.5 mi. E of Bruni, Texas, *Vergara et al. 8570* (TEX). *Edwards Co*.: along the edge of Rt. 674, 6.9 mi S of the intersection with Rt. 377, and around 10 mi SW of Rocksprings, 29°56"N, 100°20"W, *Goldman 1782* (UT). *El Paso Co*.: W Texas, on the road to El Paso del Norte, *Schott s.n.* (NY). *Hays Co*.: San Marcos and vicinity, *Stanfield s.n.*, June 1897 (NY); NW from Kyle, *Tharp 1538* (TEX, US). *Hidalgo Co*.: E side of Sullivan City, *Correll & Johnston 18048* (TEX). *Jim Hogg Co*.: 17.6 mi. SW of Hebbronville along Farm Rd., 3073 to Miranda City, *Turner & Turner 15119* (TEX). *Jim Wells Co*.: along S.R. 624, ca. 5 mi. N of Orange Grove, *Brown 4884* (NA). *Kerr Co*.: Turtle Creek, *Bray 164* (TEX, US). *Kinney Co*.: 15 mi. E of Brackettville on hwy. #90, *Butterwick et al. 316* (TEX). *La Salle Co*.: near Cotulla, Laredo to San Antonio, *Small & Wherry 11947* (NY). *Live Oak Co*.: 7.2 mi. N of Live Oak-Jum Wells County line, Rt. 281, *Escobar et al. 602* (TEX). *Mason Co*.: Johnson City, Mason, *Whitehouse 203* (TEX). *Maverick Co*.: S side of Eagle Pass, *Correll & Wasshausen 27734* (TEX). *McMullen Co*.: 4.8 mi. S of Loma Alto, *Cory 17227* (GH). *Medina Co*.: Hondo, 750 ft., *Pillsbury s.n.*, 12 April 1903 (PH). *Pecos Co*.: Madera Mts., 28 mi. S of Ft. Stockton on road to Marathon, *Correll & Schweinfurth 25413* (TEX). *San Patricio Co*.: s. side of Park Rd., 0.2mi E. of the entrance to a Boyscout camp, Camp Karankawa, just n. of Lake Corpus Christi State Park, and around 3mi SW of Mathis, 28°03"N, 97°52"W, *Goldman 1770* (BH); s. side of Park Rd., 0.2mi E. of the entrance to a Boyscout camp, Camp Karankawa, just n. of Lake Corpus Christi State Park, and around 3mi SW of Mathis, 28°03"N, 97°52"W, *Goldman 1771* (BH). *Sterling Co*.: Sterling Co. hills, *Tharp 3615* (US). *Sutton Co*.: uncommon along X 290, 14.2 mi. E of Sonora, *Mears & Mears 1492* (TEX). *Terrell Co*.: along Rio Grande between Reagan Canyon & Sanderson Canyon, 2000 ft., *Warnock 15857* (TEX). *Travis Co*.: Austin, above Barton Creek, 1 mi. S of Loop 360, 600 ft., *Larke 1* (TEX, NY). *Uvalde Co*.: Concan, *Palmer 10192* (CAS, US). *Val Verde Co*.: 4 miles west of Langtry, *Johnston 6485* (LL); near entrance to Seminole Canyon State Park, along hwy. 90, 29°42'N, 101°19'W, *Turner & Zhao 16025* (TEX). *Webb Co*.: State Hwy. 359, 7 mi. E of Laredo, *Ramos et al. 200* (DUKE, TEX). *Wilson Co*.: Sutherland Spring, *Palmer s.n.*, August 1879 (GH). *Zapata Co*.: 13.9 mi. N of San Ygnacio along US Hwy. 83, W side of hwy. along either side of the barbed wire fence line, locality best marked as between 2.1 and 2.3 mi. S of county line marker (Webb/Zapata counties), *Turner 80-68M* (TEX).

**MEXICO. Coahuila:** 22 mi. N of Nueva Rosita, near K163, 1500 ft., *Bates et al. 1479* (CAS, TEX, NY); cañon de La Barrica (S-draining), gently SW-sloping upper-bajada-type area in mouth of canyon, 27°00'02"N, 102°23'50"W, 1490 m, *Wendt & Lott 1232* (TEX). **Nuevo León:** Mpio. Higueras; W side of Mex 85, 10 km N of Cienega de Flores, ca. 1.6 km S of El Ranchito, near S end of major curves in hwy., 26°01'30"N, 100° 07'15"W, 480–540 m, *Bridges & Woodruff 13121* (TEX). **Tamaulipas:** 48 mi. from Reynosa on the San Fernando Road, 27 mi. from Matamoros-San Fernando hwy. turnoff, *Graham & Johnston 4376* (GH).

**CULTIVATED MATERIAL. United States:** North Carolina, Durham, Duke University, cultivated from material collected 17 June 1978 at Pedernales State Park, Texas, *MacDougal 227* (FLAS).

#### 7. Passiflora eglandulosa

Taxon classificationPlantaeMalpighialesPassifloraceae

J.M. MacDougal. Annals of the Missouri Botanical Garden 75: 1658–1662. figs 1, 2B, and 3. 1988.

Figs 36 –37

##### Type.

Guatemala. San Marcos: wet mountain forest at Aldea Fraternidad, W-facing slope of Sierra Madre between San Rafael Pie de La Cuesta and Palo Gordo (ca. 14°56'N, 91°52'W), 1800–2400 m, 10–18 Dec.1963, *L. O. Williams*, *A. Molina & T. P. Williams 25997* (holotype: F [F0066764F, photograph seen]; isotypes: EAP [EAP110288. photograph seen], ENCB, C, G! [G00440998], NY! [NY00110403], S! [S04-205], US! [US00588642], W).

##### Description.

Slender, climbing, perennial vine 2–8 m long, sparsely to lightly pubescent with unicellular curved trichomes on petiole, stem, and stipule, (0.1)0.4–0.6(-0.8) mm long, 0.02 mm wide, also minutely antrorsely appressed-puberulent throughout (except ovary) with unicellular, curved trichomes, 0.05–0.10 mm long, 0.02–0.03 mm wide. Flowering stems 0.6–2.1 mm in diameter, terete or subterete, with little secondary growth (to 6 mm near base with corky, secondary growth). Stipules (3.5-)5.3–12.6(-20.0) mm long, 2.50–6.4(-9.0) mm wide, ovate, slightly oblique, acute to slightly attenuate, 5–9 veins departing from the base; petioles 0.7–4.6 cm long, eglandular. Laminas 2.5–12.0 cm long, 2.2–14.5(-17.0) cm wide, chartaceous, not variegated, ratio of leaf width to central vein length 0.28–1.88, 3-lobed 0.26–0.45 of the distance to the cordate leaf base, lateral lobes 1.6–8.7 cm long, 0.8–4.4 cm wide, ovate-triangular, acute to slightly attenuate, central lobes 2.5–11.1 cm long, 1.0–5.7 cm wide, ovate-triangular, acute to slightly attenuate, angle between the lateral lobes 127–170°, ratio of lateral to central lobe length 0.64–0.97, margins entire, primary veins 3, diverging and branching at base, laminar nectaries absent; tendril 0.3–1.1 mm wide, present at flowering node, absent in inflorescence. Flowers borne in leaf axils. Pedicels 5.6–20.0 mm long, 0.5–0.9 mm wide, (1-)2 per node; bract(s) absent; spur(s) absent (occasionally) or 5 retrorse spurs present between the bases of the sepals, 0.7–1.1 mm long. Flowers 16.9–21.5 mm in diameter with stipe 2.1–7.9 mm long, 0.5–0.9 mm wide; hypanthium 4.0–5.9 mm in diameter; sepals 5.5–8.5 mm long, 2.3–3.9 mm wide, ovate-triangular, acute to rounded, the 2–3 outermost with a (0.5-)0.8–1.2 mm blunt subapical horn, abaxially and adaxially greenish yellow, often with a flush of reddish purple (5PR 3/4–4/6) abaxially (rarely to fully dark reddish purple); coronal filaments in 2 series, the outer 24–31, 2.0–4.1 mm long, 0.1–0.3 mm wide, linear, reflexed above middle and the tips often slightly incurved, greenish yellow at base, yellow distally, ratio of outer coronal row to sepal length 0.30–0.70, the inner 18–34, 0.7–1.5 mm long, 0.1–0.2 mm wide, linear, often capitate, erect, greenish yellow, ratio of inner coronal row to outer coronal row length 0.28–0.66; operculum 1.4–2.9 mm long, plicate, greenish yellow, sometimes with a flush of reddish purple at center, whitish distally, the margin with narrow minutely fimbrillate teeth; nectary 0.06–0.88 mm high, 0.6–1.5 mm wide; limen recurved, 0.2–0.5 mm high, 0.2–0.3 mm wide, whitish, limen floor 1.6–2.1 mm in diameter, whitish; androgynophore 1.3–3.5 mm long, 0.8–1.3 mm wide; free portions of the staminal filaments 2.1–3.8 mm long, 0.3–0.5 mm wide, linear, greenish yellow; anthers 2.3–3.8 mm long, 0.5–1.7 mm wide, greenish yellow, long axis oriented perpendicular (or nearly so) to long axis of filaments at anthesis; styles 3.5–6.7 mm long including stigmas, 0.2–0.4 mm wide, greenish yellow; stigmas 0.5–0.9 mm in diameter; ovary 1.2–2.8 mm long, 0.8–2.2 mm wide, widely ellipsoid to globose, greenish yellow. Berry 8.0–14.4 mm long, (7-)9.0–15.3 mm in diameter, widely ellipsoid to globose, very dark purple with glaucous bloom. Seeds 4–10, 4.5–5.7 mm long, 3.1–3.5 mm wide, 2.0–2.7 mm thick, obovate in outline, acute at both ends, reticulate-foveate with each face marked with with ca. 15–19 foveae.

**Figure 36.**
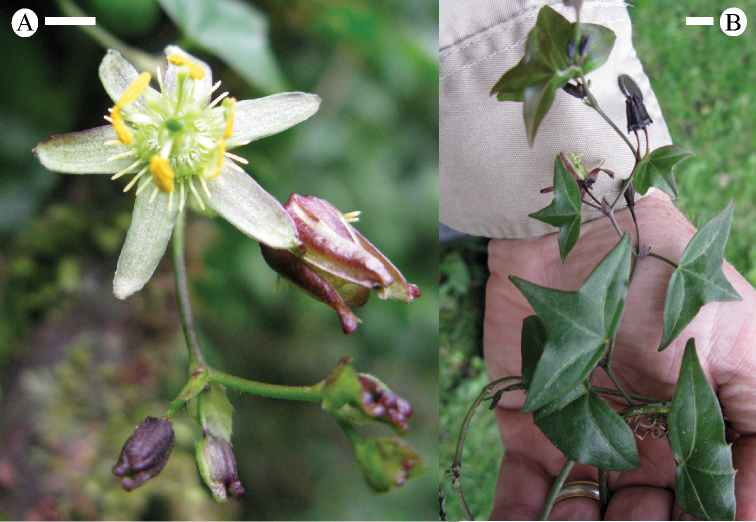
Flower and leaves of *Passiflora eglandulosa* (*MacDougal 6237*). **a** Flower and flower buds. Scale bar = 3 mm **b** Leaves, stem and flower buds. Scale bar = 3 mm. Photos by J. M. MacDougal.

**Figure 37.**
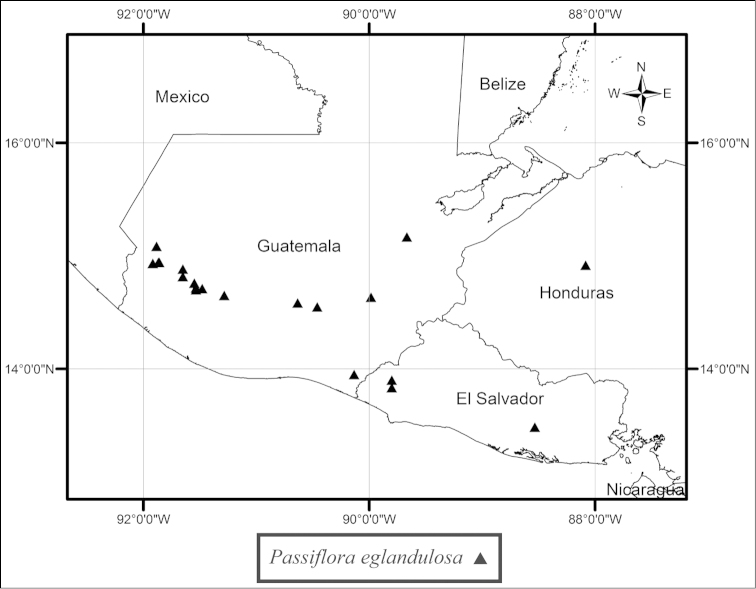
Distribution of *Passiflora eglandulosa*.

##### Phenology.

Flowering and fruiting January-May, July-September and December.

##### Distribution.

El Salvador, Guatemala, and Honduras. Growing in shrubs and small trees in shady ravines and at the edges of premontane to montane broad-leaved forests on volcanic cones; 1500–2650 m.

##### Discussion.

For many years after the publication of Killip’s 1938 monograph, the name *Passiflora trinifolia* Mast. was applied to two distinct taxa: *Passiflora eglandulosa* and *Passiflora trinifolia*. In fact, Standley and Williams (1961), in their description of *Passiflora trinifolia*, combined information from Killip’s description of *Passiflora trinifolia*, which strictly applied to *Passiflora trinifolia* in the sense of Masters, and their own personal observations of *Passiflora eglandulosa* (MacDougal 1988). It is true that the two species both possess wide foliose stipules, similarly trilobed leaves at fertile nodes and seeds with the micropylar end and chalazal beak erect and not inclined toward the raphe. However, *Passiflora eglandulosa* is distinguished by flowers with longer flower pedicels, spurs that occur between each of the sepals, narrower sepals, narrower outer coronal filaments, shorter inner coronal filaments that are not broadly capitate, narrow limen floors, short staminal filaments, and anthers that present pollen laterally as opposed to subproximally. The seeds are longer and wider than those of *Passiflora trinifolia*, and as its name implies, *Passiflora eglandulosa* lacks both laminar and petiolar nectaries; petiolar nectaries have been seen on only one specimen, *M. Veliz 16059*. *Passiflora eglandulosa* possesses flower buds that are slightly horned at the apex and flowers that are oriented above rather than near or below the horizontal plane. In comparing the habitats of the two species, MacDougal found that *Passiflora eglandulosa* is found in shady ravines and at the edges of wet premontane to montane broad-leaved forests on volcanic cones, whereas *Passiflora trinifolia* is found in open, seasonally dry pine/oak forests on rock outcrops. In addition, *Passiflora eglandulosa* is a larger plant that may climb to 4 m or more, but *Passiflora trinifolia* rarely exceeds a height of 1 m. The chartaceous leaves of *Passiflora eglandulosa* are bright green adaxially and possess drip tips, but the leaves of *Passiflora trinifolia* are dark green, lack long drip tips and are very stiff and rigid (MacDougal 1988).

*Passiflora eglandulosa* is also similar vegetatively to *Passiflora tacanensis*, a species found in montane forests on Volcán Tacaná of Chiapas, Mexico. Both species possess wide, foliose stipules. However, the two species are easily separated because *Passiflora tacanensis* possesses petiolar nectaries. The fruits of *Passiflora eglandulosa* also possess fewer than 10 seeds, whereas *Passiflora tacanensis* possesses ca. 20 seeds per fruit.

The development and physiology of the floral nectary of *Passiflora eglandulosa*, misidentified as *Passiflora trinifolia*, was examined by Durkee et al. (Durkee et al. 1981). She found that the floral nectary development and nectar secretion in this species is similar to that in the two other species of *Passiflora* that she studied. She concluded that the activity of an intercalary meristem increased starch deposition in the amyloplasts of the secretory cells parallels the maturation of the nectary phloem, and granulocrine secretion in the “starchy” nectaries does not occur. She also observed large membrane-bound protein bodies in the phloem parenchyma cells (Durkee et al. 1981).

Benson et al. (1975), in a study of the coevolution of plants and herbivores, reported that *Heliconius hortense* is an herbivore of *Passiflora eglandulosa* (misidentified as *Passiflora trinifolia*). This report was confirmed by MacDougal (MacDougal 1988).

##### Specimens examined.

**EL SALVADOR. Ahuachapán:** Cerro Grande de Apaneca, 1700 m, *Weberling 2610* (M). **Santa Ana:** Mountain Cerro Verde, 1800 m, *Molina & Montalvo 21514* (F, NY). **Sonsonate:** near top of Cerro Verde, 1860 m, *Croat 42222* (MO); Laguna de las Niñas, 1829 m, 13°53'N, 89°47'W, *Villacorta 750* (MO); Laguna Verde, 1650 m, 13°54'N, 89°48'W, *Villacorta & Gonzalez 683* (MO).

**GUATEMALA. El Progreso:** Montaña Canahui, between Finca San Miguel and summit of mountain, near upper limits of Finca Caieta, 1600–2300 m, *Steyermark 43787* (F). **Guatemala:** Choacorral, km 20 aprox. llendo a San Juan Sacatepéquez, 2000 m, *Castillo et al. 82347* (F); Santa Catarín Pinula, cerca la Cuidad Guatemala, barranca de Paraje Solar, Km 15.8 carr. de Cd. al Salvador, 1860 m, 14°32 N, 90°27 W, *MacDougal & MacVean 6210* (MO); vicinity of San Andrecillo, 1700 m, *Molina & Molina 27543* (F, U, US); near Canales, 1900 m, *Williams & Molina 11822* (F). **Huehuetenango:** Mpio. Jacaltenango, Montaña Aqo’ma, 2278 m, 15°40'N, 91°39'W, *Véliz et al. 16059* (BIGU). **Jalapa:** Volcán Jumay, N of Jalapa, 1300–2200 m, *Steyermark 32352* (F). **Quetzaltenango:** 2.5 mi. below tunnel at Santa María de Jesus between km post 202–203 on Hwy. 97, 14°42'N, 91°32'W, *MacDougal 316* (FLAS, MO); slopes of Volcán de Zunil, at and above Aguas Amargas, 2430–2850 m, *Standley 65404* (F, US); along road above Santa María de Jesús, 1680 m, *Standley 84846* (F, US); El Pocito, S of San Martín Chile Verde, on road to Colomba, 2200 m, *Standley 84997* (F, G); slopes and ridges between Quebrada Chicharro and Montaña Chicharro, on SE-facing slopes of Volcán Santa María, 1300–1400 m, *Steyermark 34360* (F, US). **San Marcos:** road between San Rafael Pie de La Cuesta and Palo Gordo, 3 km from Aldea Fraternidad toward San Marcos, parcelamiento “La Lucha,” between Km posts 264265, 2150 m, 14°56 N, 091°51 W, *MacDougal et al. 6234* (MO); road between San Rafael Pie de La Cuesta and Palo Gordo, 1 km above Aldea Fraternidad, between Km posts 266267, 1900 m, 14°56 N, 091°52 W, *MacDougal et al. 6237* (MO); road between San Rafael Pie de La Cuesta and Palo Gordo, 3 km from Aldea Fraternidad toward San Marcos, parcelamiento “La Lucha,” between Km posts 264265, 2150 m, 14°56 N, 091°51 W, *MacDougal et al. 6249* (MO); Barranco Eminencia, road between San Marcos and San Rafael Pie de la Cuesta, in upper part of the barranco between Finca La Lucha and Buena Vista, 2500–2700 m, *Standley 86379* (F); Barrancos 6 mi. S and W of Tajumulco, NW slopes of Volcán Tajumulco, below cliffs along Río Malacate, 2300–2800 m, *Steyermark 36663* (F, US); on outer slopes of Tajumulco Volcano, Sierra Madre mountains about 8–10 km W of San Marcos, 2300 m, *Williams et al. 26864* (F, GH, NY, US). **Suchitepéquez:** Volcán Santa Clara, between Finca El Naranjo and upper slopes, 1250–2650 m, *Steyermark 46628* (F, US). **Zacapa:** Ravine bordering Quebrada Alejandria, summit of Sierra de las Minas, vincinity of Finca Alejandria, 2500 m, *Steyermark 9859* (F).

**HONDURAS. Santa Bárbara:** Cuestas de piedra caliza, Dep. de Santa Bárbara, 10 km W de Lago Yojoa, 1500–2000 m, 14°55'N, 88°5'W, *Clewell & Hazlett 3858* (MO, TEFH).

#### 8. Passiflora trinifolia

Taxon classificationPlantaeMalpighialesPassifloraceae

Mast., Bot. Jahrb. 8: 217. 1887.

Figs 38 , 39

##### Type.

Guatemala. Baja Verapaz: Santa Rosa, 1600 m, 16 Apr 1882, *J. Lehmann 1314* (holotype: K [K000323139, photograph seen, photograph DUKE!]; isotype: G! [G00441028]).

##### Description.

Small, slender, low-climbing or trailing, perennial vine 0.2–1.5 (-2) m long, minutely antrorsely appressed-puberulent throughout (except ovary) with unicellular, curved and erect trichomes, 0.1–0.2 mm long, 0.02–0.03 mm wide. Flowering stems 0.9–1.8 mm in diameter, terete, sometimes red (5R 4/8) or dark purplish red, with the base somewhat cork covered. Stipules (3.8-)5.3–10.1 mm long, 2.2–7.3 mm wide, asymmetrically ovate, acute to attenuate, 5–13, veins departing from base; petioles 0.4–1.2 cm long, with 1 or 2 (rarely eglandular), round or elliptic, opposite to subopposite, sessile or shortly stipitate, saucer-shaped nectaries with flat rims, 1.0–1.5 mm wide (on the widest axis), 0.8–1.1 mm high, borne below the distal third of the petiole (0.29–0.90 of the distance from the base toward the apex of the petiole). Laminas 1.6–4.0 cm long, 2.1–6.7 cm wide, coriaceous, 3-lobed (very rarely 5-lobed) 0.05–0.52 the distance to the leaf base, lateral lobes 1.3–3.1 cm long, 0.5–1.7 cm wide, elliptic, acute, central lobe elliptic, acute to rounded, central vein 1.6–4.0 cm long, angle between the lateral lobes 93–145°, ratio of lateral lobe to central lobe length 0.67–1.28, margins entire, hyaline, primary veins 3, diverging and branching at base, laminar nectaries 1–4, circular, submarginal, associated with the minor veins of the abaxial surface, 0.6–1.1 mm in diameter, sessile; tendril 0.2–0.7 mm wide, present at flowering node. Flowers borne in leaf axils. Pedicels 5.3–7.6 mm long, 0.3–0.7 mm wide, 2 per node; bract(s) absent; spur(s) absent. Flowers 26.6–30.9 mm in diameter with stipe 1.8–3.4 mm long, 0.5–1.0 mm wide; hypanthium 8.1–8.4 mm in diameter; sepals 9.1–11.4 mm long, 5.5–6.3 mm wide, ovate-triangular, acute, abaxially and adaxially greenish yellow, reflexed at anthesis; coronal filaments in 2 series, the outer 35–39, 4.7–5.1 mm long, 0.4–0.5 mm wide, linear, somewhat dilated toward tips, semi-erect, greenish yellow at base, yellow at tips, ratio of outer coronal row to sepal length 0.42–0.53, the inner 38–47, 1.9–2.5 mm long, 0.1–0.2 mm wide, linear, capitate, erect, greenish yellow with purple (5P 5/8) spots and streaks toward base, tips whitish, ratio of inner coronal row to outer coronal row length 0.37–0.51; operculum 1.8–1.9 mm long, plicate, whitish, tinged with purple, the margin with narrow minutely fimbrillate teeth; nectary 0.1–0.5 mm high, 0.9–1.0 mm wide; limen erect, 0.1 mm high, 0.3–0.4 mm wide, limen floor 4.0–4.3 mm in diameter, whitish; androgynophore 3.8–4.0 mm long, 1.2–1.4 mm wide, whitish with purple spots and streaks; free portions of the staminal filaments 3.5–4.2 mm long, 0.7–0.9 mm wide, linear, greenish yellow; anthers 2.9–3.3 mm long, 1.8–2.0 mm wide, greenish yellow; styles 4.4–5.3 mm long including stigmas, 0.4–0.5 mm wide, greenish yellow; stigmas 1.3–1.7 mm in diameter; ovary 1.9–2.3 mm long, 1.8–2.0 mm wide, widely ellipsoid, greenish yellow. Berry 10.5–12.9 mm long, 10.3–10.5 mm in diameter, ellipsoid or globose, very dark purple. Seeds 19 (n = 1 *MacDougal* 6228), 3.6–4.0 mm long, 2.1–2.4 mm wide, 1.6–1.8 mm thick, obovate in outline, acute at both ends, reticulate-foveate with each face marked with ca. 12–15 foveae.

**Figure 38.**
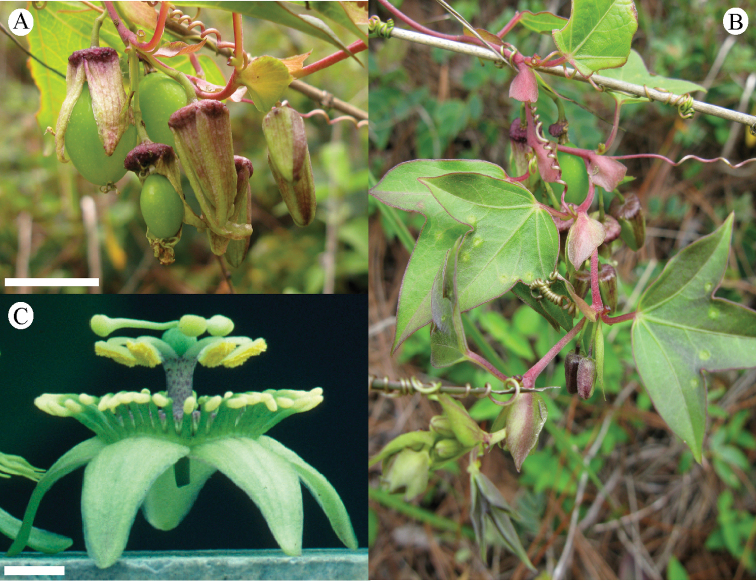
Flower, immature fruits, and leaves of *Passiflora trinifolia* **a** Immature fruits (*MacDougal 6223*) Scale bar = 10 mm. Photo by J. M. MacDougal **b** Plant habit (*MacDougal 6223*) Photo by J. M. MacDougal **c** Flower (*MacDougal* 637*GR*) Scale bar = 5.0 mm. Photo by J. M. MacDougal.

**Figure 39.**
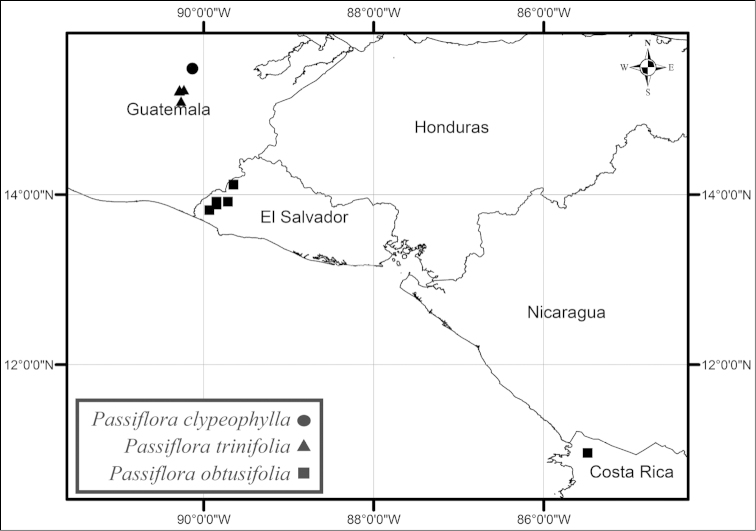
Distribution of *Passiflora clypeophylla*, *Passiflora trinifolia*, and *Passiflora obtusifolia*.

##### Phenology.

Flowering and fruiting in February, April, and July.

##### Distribution.

Endemic to Guatemala, in the department of Baja Verapaz. Seasonally dry rocky (the vernacular name for the rock type is “cascajo”) hills with open grassy forest of pine, some oak, and agave, especially near rock outcroups or cracks on cliffs and roadcut faces; 1345–1600 m.

##### Discussion.

*Passiflora trinifolia* is known only from Baja Verapaz, Guatemala. It is usually easily distinguished from other members of supersection *Cieca* by its small, stiff and rigid (often scleophyllous) leaves and very small stature. *Passiflora trinifolia* has been confused with *Passiflora eglandulosa*, but several vegetative and reproductive characters can be used to separate these taxa, as presented under the description of *Passiflora eglandulosa*. The most notable of these is the presence/absence of petiolar and laminar nectaries, with *Passiflora trinifolia* possessing 1–4 laminar nectaries and 1–2 petiolar nectaries, and *Passiflora eglandulosa* having neither laminar nor petiolar nectaries.

##### Specimens examined.

**GUATEMALA. Baja Verapaz:** Hacienda Santa Rosa, now in the Estrada family, type locality on old rd. from Pantín to Salamá (Rt. 5), ca. 4.5 km S of Pantín, 21 km from Salamá, 5200 ft., *MacDougal & Miley 637* (FLAS, MO); Finca Santa Rosa on old road between Pantín and Salamá, hill directly behind ruins of main finca house, ca. ½ way up, 1585 m, 15°13 N, 090°17 W, *MacDougal & Moroni 6223* (MO); Finca Santa Rosa on old road between Pantín and Salamá, first ridge on road leading out of valley to the SW from the old homestead, 1592 m, 15°13 N, 90°16 W, *MacDougal 6225* (MO); Finca Santa Rosa on old road between Pantín and Salamá, first ridge on road leading out of valley to the SW from the old homestead, 1592 m, 15°13 N, 90°16 W, *MacDougal & Moroni 6226* (MO); S of old Finca Santa Rosa on old road to Salamá, 1512 m, 15°13 N, 90°17 W, *MacDougal & Moroni 6227* (MO); old road between Pantín and Salamá, S of Finca Santa Rosa, near small river crossing, 1345 m, 15°12 N, 90°17 W, *MacDougal & Moroni 6228* (MO); Cuesta de Cachil, near Salamá, 1200–1600 m, *Pittier 160* (US); Santa Rosa, *von Tuerckheim 1207* (G, GH).

#### 9. Passiflora clypeophylla

Taxon classificationPlantaeMalpighialesPassifloraceae

Mast., Bot. Gaz. 16: 6–7. 1891.

Figs 39 , 40

##### Type.

Guatemala. Alta Verapaz: Barranca del Rubelcruz, 2500 pp., [estimated coordinates 15°29'N, 90°08'W], Apr 1889, *J. Donnell Smith 1625* (lectotype, designated here: K! [K000323141]; isolectotype: US! [US00036858]).

##### Description.

Climbing vine, minutely antrorsely appressed-puberulent throughout with unicellular, curved trichomes, 0.03–0.10 mm long, 0.03 mm wide. Flowering stems 2.1–3.4 mm in diameter, subterete. Stipules (3.3-)5.9–6.4 mm long, 0.8–1.3 mm wide, narrowly ovate-triangular, acute; petioles 3.3–3.8 cm long, with 2, opposite to subopposite, sessile, discoid nectaries with flat rims, 1.3–1.7 mm wide (on the widest axis), 0.5–0.6 mm high, borne on the proximal half of the petiole (0.37–0.47 of the distance from the base toward the apex of the petiole). Laminas 6.0–8.7 cm long, 6.7–10.8 cm wide, somewhat coriaceous, distinctly peltate (the distance from leaf base to point of petiole insertion 10.4–14.4 mm), subrotund, obscurely 3-lobed 0.02–0.07 the distance from the leaf outline to the leaf base, lateral lobes 4.0–6.2 cm long, ca. 3.0–5.6 cm wide, somewhat elliptic, obtuse to emarginate, central lobe somewhat elliptic, obtuse to emarginate, central vein 4.6–7.3 cm long (measured from point of petiole insertion to the leaf apex), angle between the lateral lobes 110–125°, ratio of lateral lobe to central vein length 0.76–0.87, margins entire, hyaline, primary veins 3, diverging and branching above base, laminar nectaries present, 2, submarginal, associated with the minor veins of the abaxial surface, 0.8–0.9 mm in diameter, circular to widely elliptic, sessile; tendril 0.5–0.9 mm wide, present at flowering node. Flowers borne in leaf axils. Pedicels 16.9–17.3 mm long, 0.6 mm wide, 2 per node; bract(s) absent; spur(s) absent. Flowers 25.0–26.3 mm in diameter with stipe 9.4–14.3 mm long, 0.5–0.8 mm wide; hypanthium 5.8 mm in diameter; sepals 9.6–10.3 mm long, 4.3–5.0 mm wide, ovate-triangular, acute, greenish yellow; coronal filaments in 2 series, the outer 28, 4.7–5.5 mm long, 0.4–0.6 mm wide, linear, spreading, purplish to reddish with greenish yellow or yellow tips when dried, ratio of outer coronal row to sepal length 0.46–0.58, the inner 31, 2.3–2.5 mm long, 0.3 mm wide, linear, capitate, erect, purplish to reddish when dried, ratio of inner coronal row to outer coronal row length 0.42–0.51; operculum 1.6 mm long, plicate, purplish to reddish with greenish yellow tip when dried, the margin with narrow minutely fimbrillate teeth; nectary 0.3 mm high, 1.2 mm wide; limen recurved, 0.3 mm high, 0.2 mm wide, purplish to reddish at base lightening toward tip when dried, limen floor 2.7 mm in diameter, purplish to reddish when dried; androgynophore 3.8 mm long, 0.9 mm wide, purplish to reddish on proximal half and greenish yellow on distal half when dried; free portions of the staminal filaments 3.4–3.8 mm long, 0.5–0.6 mm wide, linear, greenish yellow when dried; anthers 1.7–2.0 mm long, 0.9–1.3 mm wide; styles 3.8–4.3 mm long including stigmas, 0.4–0.5 mm wide, greenish yellow when dried; stigmas 1.1–1.3 mm in diameter; ovary 1.8 mm long, 1.5 mm wide, globose, greenish yellow when dried. Fruit unknown.

**Figure 40.**
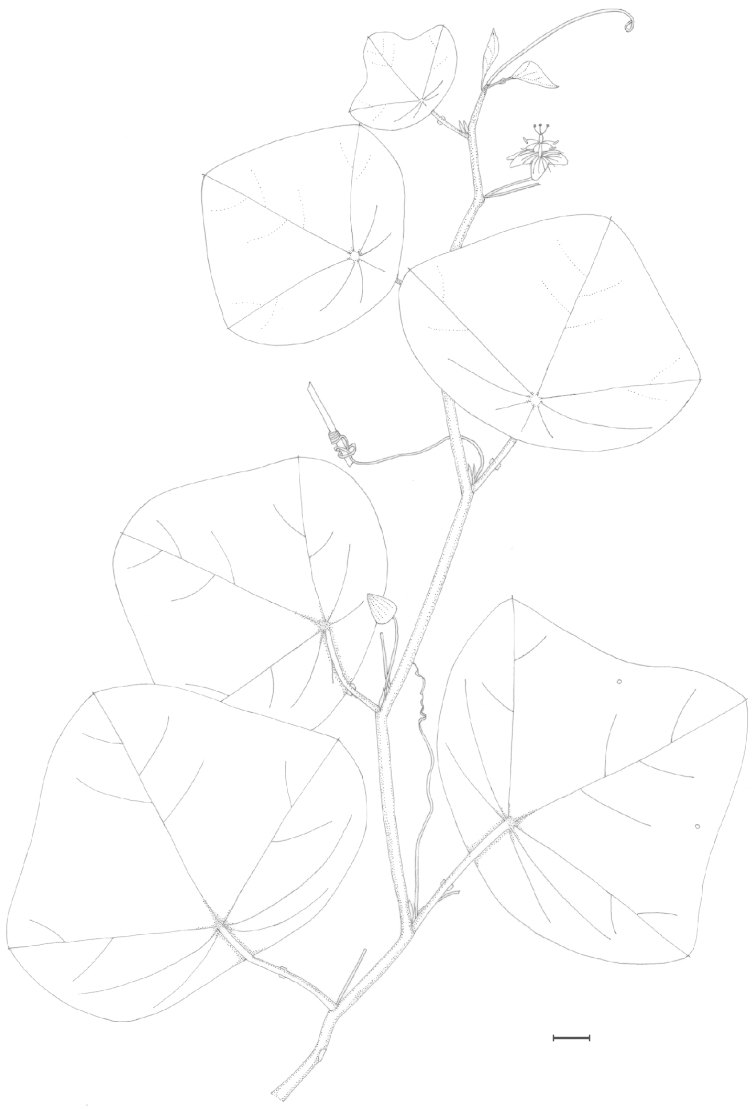
Habit of *Passiflora clypeophylla* (based upon *Smith 1625*) Scale bar = 1.0 cm.

##### Phenology.

The species has been collected in flower in April.

##### Distribution.

Endemic to Guatemala in the department of Alta Verapaz at ca. 762 m altitude. Based upon locality information included on the herbarium specimen and information gathered by J. M. MacDougal (pers. comm.) on a recent trip to the type locality, *Passiflora clypeophylla* is (or was) likely found on slopes of premontane tropical moist forest.

##### Discussion.

*Passiflora clypeophylla* is known only from the type collection from Alta Verapaz, Guatemala. *Passiflora clypeophylla* is distinctive in supersection *Cieca* because of its large, conspicuously peltate leaves that are deltoid in general outline. The flowers are not known to be borne in inflorescences and the pedicels are greater than 16.8 mm long. The floral stipe of *Passiflora clypeophylla* is also one of the longest in the supersection and is greater than 9.4 mm long. In addition, the plant has very shallow leaf lobes (0.03–0.07 of the distance from the leaf outline to the leaf base).

*Passiflora clypeophylla* resembles both *Passiflora trinifolia* and *Passiflora sexocellata*, which are somewhat similar vegetatively and also occur in Guatemala. *Passiflora clypeophylla* is easily distinguished from *Passiflora trinifolia* by its considerably narrower stipules, the obtuse to rounded leaf lobes that are very shallow and the leaves that are coriaceous as opposed to chartaceous in texture. The primary difference between *Passiflora sexocellata* and *Passiflora clypeophylla* is the ratio of the lateral to central lobe length. *Passiflora clypeophylla* has lateral and central leaf lobes that are nearly equal in length, whereas *Passiflora sexocellata* has lateral lobes that are commonly 1.3 to 2.8 times longer than the central lobes. *Passiflora sexocellata* also commonly has a shorter central leaf lobe and more laminar nectaries than *Passiflora clypeophylla*. As with *Passiflora trinifolia*, the lateral leaf lobes in *Passiflora sexocellata* are commonly acute as opposed to obtuse to rounded. The one known flower of *Passiflora clypeophylla* has fewer filaments in the outer coronal row (28 filaments) than either *Passiflora trinifolia* (35–39 filaments) or *Passiflora sexocellata* (40–50). The staminal filaments in *Passiflora clypeophylla* are nearly equal to the androgynophore length, but the filaments in *Passiflora sexocellata* are commonly half the length of the androgynophore.

The seedling leaves of several species in supersection *Cieca* (e.g., *Passiflora sexocellata*, *Passiflora megacoriacea*, *Passiflora juliana*, and *Passiflora viridiflora*) are peltate and very similar in shape to the mature leaves of *Passiflora clypeophylla*, and evolution by neoteny in this taxon seems plausible.

There are only two known specimens of *Passiflora clypeophylla* in the world, one at the Kew Herbarium and the other at the United States National Herbarium. In his description of *Passiflora clypeophylla* Masters did not cite a herbarium, only a collection. The specimen at K is much better than the one at US, so I have designated it the lectotype.

##### Specimens examined.

Only known from the type collection.

#### 10. Passiflora obtusifolia

Taxon classificationPlantaeMalpighialesPassifloraceae

Sessé & Moc., Pl. N. Hispan. ed. 1: 156. 1890.

Figs 39 , 41

##### Type.

Mexico. Michoacán: Apatzingán, Oct 1790, *M. Sessé & J. Mociño s.n.* (lectotype designated by R. McVaugh 2000, pg. 428: original illustration in the Torner Collection of the Hunt Botanical Institute 6331.830 [photograph F!]).

##### Description.

Slender, low-climbing, perennial vine 1.5–3 m long or more, minutely antrorsely appressed-puberulent throughout with unicellular, curved to erect trichomes, 0.1–0.2 mm long, 0.02–0.03 mm wide, also sparsely pubescent with longer, unicellular, curved to erect trichomes on petiole and stem, 0.2–0.4 mm long, 0.02–0.03 mm wide. Flowering stems 0.9–2.1 mm in diameter, terete or somewhat compressed, with the base woody and cork-covered. Stipules 1.4–5.7 mm long, 0.3–1.0 mm wide, very narrowly ovate, acute to attenuate, longitudinally striate-nerved; petioles 0.5–2.8 cm long, 2, round to elliptic, opposite, sessile, discoid nectaries, 1.1–2.2 mm wide, 0.2–1.3 mm high, borne below the distal half of the petiole (0.40–0.83 of the distance from the base toward the apex of the petiole). Laminas 2.4–12.6 cm long, 3.4–18.2 cm wide, subcoriaceous, sometimes peltate, distinctly trilobed 0.36–0.60 the distance from the leaf outline to the leaf base or widely divaricately bilobed to obscurely 3-lobed 0.09–0.28 the distance from the leaf outline to the leaf base, lateral lobes 2.0–10.0 cm long, 0.7–4.4 cm wide, elliptic, acute to obtuse, occasionally attenuate, central lobe elliptic to obovate, or present merely as a widely acute to obtuse tip, rarely emarginate, central vein 2.4–12.1 cm long, angle between the lateral lobes 88–151°, ratio of lateral lobe to central lobe length 0.74–1.64, margins entire, hyaline, primary veins 3, diverging and branching at or above base, laminar nectaries present or rarely absent, 2–4(-11), circular, submarginal, associated with the minor veins of the abaxial surface, 0.6–1.3 mm in diameter, sessile; tendril 0.3–1.1 mm wide, present at flowering node, absent in inflorescence. Flowers borne in leaf axils and terminal inflorescences; inflorescences 5.3–18.3 cm long, associated reduced laminas 2.3–4.9 mm long, 0.5–1.4 mm wide. Pedicels 3.8–6.8(-19.5) mm long, 0.4–0.8 mm wide, 2 per node; bract(s) absent, or with 1–2 narrowly ovate bracts present on the distal half of the pedicel, 1.0–2.0 mm long, ca. 0.1 mm wide; spur(s) absent. Flowers 14.6–21.6 mm in diameter with stipe 3.1–4.6 mm long, 0.4–0.9 mm wide; hypanthium 4.3–6.3 mm in diameter; sepals 4.7–7.8 mm long, 2.1–4.7 mm wide, ovate-triangular, acute, greenish yellow, often flushed with reddish purple abaxially; coronal filaments in 2 series, the outer 28–38, 1.3–3.0(-4.3) mm long, 0.3–0.4 mm wide, linear, often capitellate, strongly curved at the base so that the filaments spread ± horizontally, with the tips often curved toward the sepals, greenish yellow, sometimes flushed with reddish purple at base, ratio of outer coronal row to sepal length 0.22–0.56(-0.85), the inner 38–40, 0.9–3.3 mm long, 0.1–0.3 mm wide, linear, capitate, erect, greenish yellow, ratio of inner coronal row to outer coronal row length 0.55–1.15; operculum 1.0–2.1 mm long, plicate, greenish yellow, sometimes reddish purple at base, the margin whitish with narrow minutely fimbrillate teeth; nectary 0.1–0.5(-0.9) mm high, 0.8–1.5 mm wide; limen erect, 0.1–0.5 mm high, 0.1–0.4 mm wide, greenish yellow, limen floor 1.0–3.5 mm in diameter, greenish yellow; androgynophore 0.7–3.9 mm long, 0.7–1.3 mm wide, greenish yellow, whitish at base; free portions of the staminal filaments 1.6–3.7 mm long, 0.3–0.7 mm wide, linear, greenish yellow; anthers 1.0–3.0 mm long, 0.5–1.7 mm wide, greenish yellow; styles 2.2–4.1 mm long including stigmas, 0.2–0.5 mm wide, greenish yellow; stigmas 0.8–1.6 mm in diameter; ovary 2.0–2.3 mm long, 1.4–2.2 mm wide, widely ellipsoid to globose, greenish yellow. Berry 2.3–2.6 cm long, 2.1–2.4 cm in diameter, widely ellipsoid to globose, very dark purple. Seeds 3.3–3.6(-5) mm long, 2.0–2.3(-3.2) mm wide, 1.5–1.8 mm thick, obovate in outline, acute at both ends, reticulate-foveate with each face marked with 15–17 foveae. Germination unknown.

**Figure 41.**
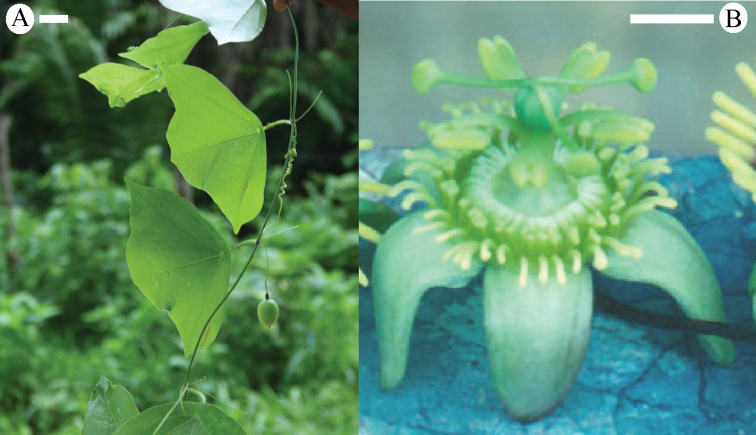
**a** Habit and fruit of *Passiflora obtusifolia* (*Porter-Utley & Ramírez 489*) **b** *Flower* of *Passiflora obtusifolia* (*MacDougal 495GR*) Scale bar = 3.0 mm. Photo by J. M. MacDougal.

##### Phenology.

Flowering and fruiting October to January and May.

##### Distribution.

Costa Rica, El Salvador, and Mexico. Tropical deciduous and subdeciduous forests or disturbed areas in the Pacific lowlands and foothills; near sea level to 300 m in Mexico, 650–1200 m in El Salvador and Costa Rica.

##### Discussion.

As noted by MacDougal and McVaugh (2001), *Passiflora obtusifolia* is quite variable in its vegetative morphology, especially in the depth of the leaf lobes, the shape of the lobe apices, and the number of laminar nectaries. Despite its name, the lobes of *Passiflora obtusifolia* are commonly acute. The type is an illustration of an unusual form that has only been collected again near Cerro de Ortega, Colima, Mexico (*Lott 840*), not far from the type locality. The illustration shows a plant with shallowly trilobed leaves with obtuse lateral lobes, rounded to emarginate central lobes, and six laminar nectaries per leaf, with two glands situated proximal to the lateral leaf veins.

*Passiflora obtusifolia* is similar to *Passiflora mcvaughiana* and both are found in southwestern Mexico. However, these species differ in leaf shape, depth of lobing, number of laminar nectaries, number of petiolar nectaries, pedicel length, sepal length, outer coronal length and shape, seed size, and habitat. *Passiflora obtusifolia* can also be found in locations somewhat near *Passiflora eglandulosa*. At first glance these two species are somewhat similar vegetatively with their distinctly trilobed leaves. However, the stipules of *Passiflora eglandulosa* are much wider and foliose, the leaf bases are cordate, and the leaf apices are acuminate. In addition, *Passiflora eglandulosa* does not possess inflorescences and its flowers are more delicate with narrower sepals and thinner outer coronal filaments. *Passiflora obtusifolia* is also similar to Passiflora suberosa subsp. litoralis. However, Passiflora suberosa subsp. litoralis is never peltate at the reproductive nodes, whereas *Passiflora obtusifolia* is commonly peltate. Passiflora suberosa subsp. litoralis does not produce flowers in long inflorescences. The fruits of *Passiflora obtusifolia* are over 20 mm long and 18 mm wide, but the fruits of Passiflora suberosa subsp. litoralis rarely exceed a length of 12 mm and a width of 10 mm.

##### Specimens examined.

**MEXICO. Colima:** Back of dunes E side of Manzanillo Bay, *Ferris 6208* (US); Isla Socorro, Archipielago de Revillagigedo, 220 m, *Flores & Martínez 851* (MO); Ravine ca. 0.7 km N-NW of summit of Cerro, Socorro Island, 915 m, *Levin 2046* (MO); 1.7 km SE de Cerro de Ortega, Ribera del Río Coahuayana, *Lott & Magallanes 840* (DUKE); Santiago village, near Manzanillo, 5–10 m, *Stork et al. 25409* (UC, US). **Jalisco:** Entre la Manzanilla & el Tamarindo, Mpio. La Huerta, *Guzman & Mejia 180* (IBUG). **Michoacán:** Mpio. Coahuayana, San Telmo, *MacDougal & Miley 495* (MO, US); Apatzingán, *Sessé & Mociño 4462* (AAU, F, G, MO). **Nayarít:** S of Penal Colony, María Madre, Tres Marías Islands, *Ferris 5598* (A, DS); Tres Marías Islands, María Madre, Arroyo Honda, *Mason 1172* (US); Cerro de la Cruz, E of Tepic, 1000 m, *Mexia 666* (UC). **Oaxaca:** Temazcal, Tuxtepec, *MacDougal 4687* (FLAS); Dto. Tuxtepec, cortina de la Presa Miguel Alemán, Temazcal, *Martínez & Ramos* 24029-A (MEXU).

**COSTA RICA. Guanacaste:** Guanacaste, La Cruz, Santa Elena, Parque Nacional Guanacaste, Estación Maritza, 650 m, *Estrada 3028* (MO). **El Salvador Ahuachapán:** alrededores de Ataco, por la calle vieja a Ahuachapán, 1200 m, *Linares 3776* (MEXU); San Benito, al N de la Cumbre, *Sandoval & Chinchilla 495* (MO); Departamento de Ahuachapán, *Padilla 163* (US); Sierra de Apaneca, in the region of Finca Colima, *Standley 20188* (US); Parque Nacional El Imposible, *Villacorta et al. 879* (MO).

**CULTIVATED MATERIAL. United States.** Florida, cultivated at the University of Florida from material collected by J.M. MacDougal and J. Miley (*MacDougal & Miley 495*) in San Telmo, México, *Porter-Utley P-67* (FLAS).

#### 11. Passiflora juliana

Taxon classificationPlantaeMalpighialesPassifloraceae

J.M. MacDougal. Novon 2: 358–361. fig 1. 1992.

Figs 42 , 43

##### Type.

Mexico. Michoacan: Mpio. Coahuayana, high point on coastal road (Hwy. 200) between San Telmo and San Juan de Lima, 70 m, 2 Nov. 1979, *J. M. MacDougal 492* (holotype: DUKE; isotypes: B! [B 10 0249190], CAS, [CAS0000382, photograph seen], CHAPA, DUKE, ENCB, F! [F0044453F], G! [G00441015], GH! [GH00063134], IBUG, MICH [MICH1115897, photograph seen], MO [MO-501793, photograph seen], MEXU! [MEXU00447466], NY [NY00335342, photograph seen], P [P00098890, photograph seen] TEX! [TEX00031092, photograph seen], US! [US00588766], XAL).

##### Description.

Slender, climbing, perennial vine 3 m long or more, minutely antrorsely appressed-puberulent throughout with unicellular, curved trichomes, 0.03–0.13 mm long, 0.02–0.03 mm wide. Flowering stems 1.0–2.7 mm in diameter, terete to somewhat compressed with rounded edges, greenish yellow or reddish purple (5RP 5/6), with the base somewhat woody and cork-covered. Stipules (6.0)8.3–18.9(-23.0) mm long, 2.8–11.3(-15.0) mm wide, asymmetrically ovate to obovate, acute, 5–10 veins departing from the base; petioles 1.1–4.3 cm long, inserted 2.3–15.8 mm from the basal margins of the peltate blades, commonly bearing on the proximal half (0.21–0.52 of the distance from the base toward the apex of the petiole), 2, round or elliptic, opposite to subopposite, sessile or shortly stipitate, saucer-shaped nectaries with flat rims, 0.9–2.7 mm wide, 0.3–2.1 mm high. Laminas 3.4–14.0 cm long, 6.0–20.0 cm wide, coriaceous, occasionally variegated as juveniles, conspicuously peltate, deeply 3-lobed (0.42-)0.50–0.86 the distance from the leaf outline to the leaf base, lateral lobes 2.9–11.1 cm long, 1.2–4.5 cm wide, elliptic to obovate, acute to obtuse, central lobes 3.1–13.2 cm long, 1.3–5.0 cm wide, obovate, acute to obtuse, narrowed at base, angle between the lateral lobes 95–160°, ratio of lateral to central lobe length 0.75–0.99, margins entire, thickened, sometimes purplish red, primary veins 3, diverging and branching above base, 4–11 laminar nectaries present, submarginal, associated with the minor veins of the abaxial surface, 0.3–1.0 mm in diameter, circular to widely elliptic, sessile; tendril 0.4–1.1 mm wide, present at flowering node, absent in inflorescence. Flowers paired in leaf axils or in terminal inflorescences; inflorescences 4.7–9.8 cm long, associated reduced laminas 7.0–14.4 mm long, 0.5–1.3 mm wide. Pedicels 2.9–19.0(-27.0) mm long, 0.5–1.1 mm wide; bract(s) absent; spur(s) absent. Flowers 24.5–31.3 mm in diameter with stipe 0.9–2.0 mm long, 0.6–1.3 mm wide; hypanthium 6.0–8.3 mm in diameter; sepals 9.3–11.5 mm long, 2.6–4.9 mm wide, ovate-triangular, acute to rounded, abaxially and adaxially greenish yellow; coronal filaments in 2 series, the outer 38–46, 4.9–7.2 mm long, 0.3–0.7 mm wide, linear, tapering to a point, spreading flat, greenish yellow becoming gradually lighter in color apically, unmarked or with purple (5P 3/6) spots and streaks near base, ratio of outer coronal row to sepal length 0.47–0.74, the inner 40–47, 3.0–3.8 mm long, 0.2–0.4 mm wide, linear, capitellate, erect to slightly spreading, greenish yellow, unmarked or with a flush of purple at very base, ratio of inner coronal row to outer coronal row length 0.43–0.73; operculum 2.0–2.5 mm long, plicate, greenish yellow, the margin with narrow minutely fimbrillate teeth; nectary 1.3–1.9 mm high, 0.7–1.3 mm wide; limen recurved, 0.8–1.1 mm high, 0.2–1.0 mm wide, greenish yellow, unmarked or with a violet to dark purple tip, limen floor 2.6–3.3 mm in diameter, dark purple (5P 2.5/6); androgynophore 3.9–5.5 mm long, 1.0–1.4 mm wide, whitish with a flush of purple at the base or with the purple coloration nearly reaching the apices of the staminal filaments; free portions of the staminal filaments 2.9–4.0 mm long, 0.5–0.7 mm wide, linear, commonly greenish yellow except as noted above; anthers 3.2–4.8 mm long, 1.0–2.2 mm wide; styles 4.0–6.5 mm long including stigmas, 0.2–0.5 mm wide, greenish yellow; stigmas 1.1–1.7 mm in diameter; ovary 2.6–3.1 mm long, 2.0–2.4 mm wide, globose to ovoid, greenish yellow. Berry (13.0-)17.3–18.1(-25.0) mm long, (13.0-)14.3–14.4(-20.0) mm in diameter, globose, very dark purple with glaucous bloom. Seeds 45–55, 3.7–4.1 mm long, 2.3–2.6 mm wide, 1.5–1.9 mm thick, obovate in outline, acute at both ends, reticulate-foveate with each side marked with ca. 11–18 foveae. Germination epigeal.

**Figure 42.**
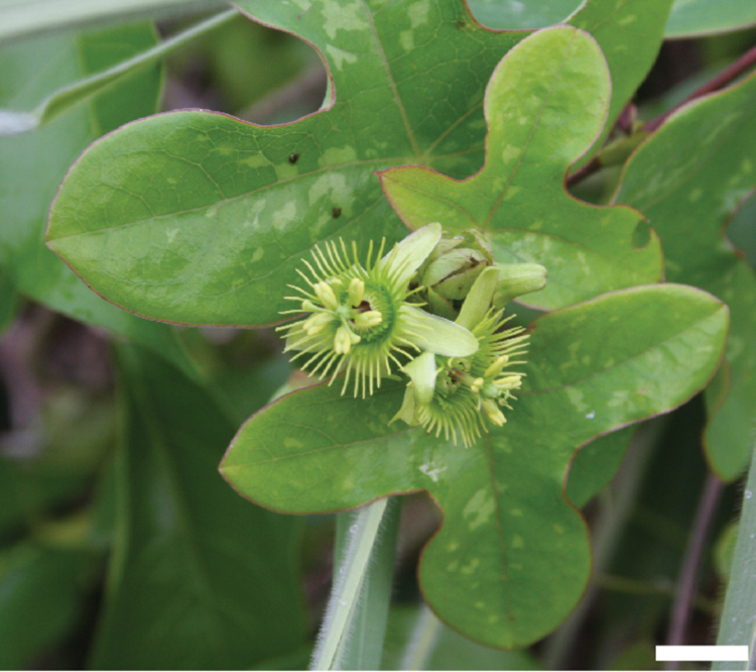
Habit of *Passiflora juliana* (*Porter-Utley &Ramirez 488*) Scale bar = 10.0 mm.

**Figure 43.**
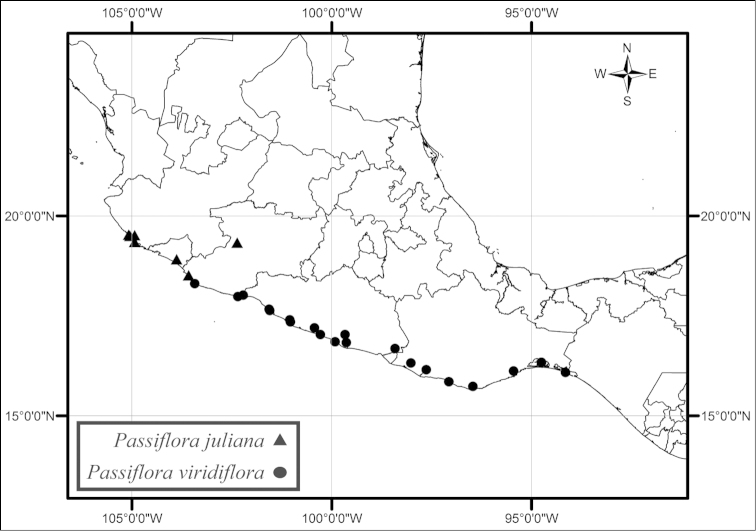
Distribution of *Passiflora juliana* and *Passiflora viridiflora*.

##### Phenology.

Flowering and fruiting August to November.

##### Distribution.

Mexico, in the Pacific lowlands and foothills of Jalisco, Colima, and northern Michoacán. Disturbed tropical deciduous or semideciduous low and medium forests (selva baja caducifolia and selva mediana subcaducifolia); growing on shrubs, trees, boulders, and rocks (sometimes limestone); sea-level to ca. 610 m.

##### Discussion.

*Passiflora juliana* is most closely related to *Passiflora viridiflora* and aside from floral adaptations in *Passiflora viridiflora* resulting from a shift in pollinators, these two species with greenish yellow flowers borne in conspicuous, indeterminate, terminal inflorescences are very similar. Both species possess large, peltate, trilobed leaves that have a central lobe that is distinctly narrowed at the base. They both may possess stems that have some red pigmentation, but those of *Passiflora viridiflora* are generally bright red, while those of *Passiflora juliana* are commonly reddish purple. *Passiflora juliana* can also be separated from *Passiflora viridiflora* vegetatively because that species has small, narrowly ovate stipules, as opposed to the larger, ovate, foliose stipules of *Passiflora juliana*. *Passiflora juliana* bears the shallow cup-shaped flowers typical of most of the members of the supersection and subgenus, whereas *Passiflora viridiflora* possesses long, tubular flowers with a greatly elongated androgynophore. *Passiflora juliana* is a very distinctive taxon possessing the shortest floral stipe in supersection *Cieca*, a limen floor that is distinctly purple and an androgynophore flushed with purple at the base or to just above the middle.

The light green flowers are likely adapted to a small or medium-sized insect pollinator, but J. M. MacDougal (1992) observed a hummingbird visiting the flowers of this plant. In an unpublished manuscript, MacDougal determined the total sugar concentration measured as sucrose equivalents in percent weight per total weight to be 35–38% in *Passiflora juliana*, which is within the range typical for utilization by bees. MacDougal also found that lacebugs and the butterfly *Heliconius charitonia* are important herbivores.

##### Specimens examined.

**MEXICO. Colima:** Mpio. Tecoman, N of Tecoman, 3.9 mi. NE on Hwy. 110 from junction of road to Tecoman (Hwy. 200), *MacDougal & Miley 486* (US); Hwy. 200 between Manzanillo and Tecoman, sea level, 19°00.77N, 104°11.78W, *Porter-Utley & Mondragón 359* (CICY, FLAS). **Jalisco:** Mpio. La Huerta, Rancho Cuixmala, road to Cumbres 1 from Station 45, E of the Puerto Vallarta, B. de Nav. (MEX 200) hwy., 19°31'N, 104°56'W, *Ayala 1212* (CAS, MO, TEX); Chamela, sendero El Tejón, 19°30'N, 105°03'W, 100 m, *Gentry & UNAM Tropical Ecology Class 74432* (MO); Estación de Biología, Chamela, IBUNAM, Chacahalaca Trail, 90 m, 19°29.92N, 105°02.63W, *Porter-Utley & Mondragón 353* (CICY, FLAS); Estacion de Biologia, Chamela, IBUNAM, Chacahalaca Trail, *Porter-Utley & Mondragón 355* (CICY, FLAS); at entrance to the Estación de Biología, Chamela, IBUNAM, 80 m, 19°29.64N, 105°02.81W, *Porter-Utley & Mondragón 357* (CICY). **Michoacán:** Mpio. Apatzingán, Tancitaro Region, Mt. Apatzingán, 2000 ft, *Leavenworth & Hoogstraal 1717* (F); high point on raod between San Telmo and San Juan de Lima (Hwy 200).

##### Cultivated material.

**United States of America:** Missouri, cultivated at the Missouri Botanical Garden, from material collected by J.M. MacDougal & J. Miley (*MacDougal & Miley 492*) in Michoacan, Mexico, *MacDougal 492GR* (MO).

#### 12. Passiflora viridiflora

Taxon classificationPlantaeMalpighialesPassifloraceae

Cav. Icon. Pl. 5: 15, pl. 424. 1799.

Figs 43 , 44

Tacsonia viridiflora (Cav.) Juss. Ann. Mus. Hist. Nat. 6: 389. 1805. Type: Based on *Passiflora viridiflora* Cav.
Murucuia viridiflora (Cav.) Spreng. Syst. Veg. 3: 43. 1826. Type: Based on *Passiflora viridiflora* Cav.
Synactila viridiflora (Cav.) Raf., Fl. Tellur. 4: 104. 1838. Type: Based on *Passiflora viridiflora* Cav.
Psilanthus viridiflorus (Cav.) M. Roem. Fam. Nat. Syn. 2: 198. 1838. Type: Based on *Passiflora viridiflora* Cav.
Passiflora tubiflora Kunth. Nov. Gen. & Sp. 2: 139. 1817. Type: Mexico. Guerrero: Acapulco, *A. Humboldt & A. Bonpland 3886* (holotype: P [P00307398, photograph seen]).

##### Type.

Mexico. Guerrero: Acapulco, *L. Née s.n.* (holotype: MA! [MA603045], photographs DUKE!, F!, MEXU]; isotype: F! [0044450F]).

##### Description.

Slender, climbing or trailing, perennial vine 3 m long or more, minutely antrorsely appressed-puberulent throughout (except on ovary) with unicellular, curved trichomes, 0.05–0.13 mm long, 0.02 mm wide. Flowering stems 1.1–2.8 mm in diameter, somewhat compressed and two-edged, red (4/12) when young, with the base somewhat woody and cork-covered. Stipules 2.5–7.9 mm long, 0.5–1.4 mm wide, asymmetrically narrowly ovate-falcate, slightly attenuate, longitudinally striate-nerved, often red (5R 4/12) at flowering nodes; petioles 1.1–7.6 cm long, inserted 0.4–22.0 mm from the basal margins of the peltate blades, often red (4/12) at flowering nodes, commonly bearing in the proximal third, 0.12–0.33(-0.55) of the distance from the base toward the apex of the petiole, 2, round or elliptic, opposite to subopposite, sessile or shortly stipitate, saucer-shaped nectaries with flat rims, 0.9–2.5 mm wide (on the widest axis), 0.3–1.5 mm high. Laminas 3.7–14.1 cm long, 1.6–19.6 cm wide, coriaceous, occasionally variegated, conspicuously peltate, deeply 3-lobed 0.60–0.82 of the distance to the leaf base, lateral lobes 1.6–10.6 cm long, 1.0–5.8 cm wide, oblong to obovate, acute to rounded, central lobes 1.9–14.1 cm long, 1.0–6.5 cm wide, obovate, acute to rounded, narrowed at base, angle between the lateral lobes 117–180°, ratio of lateral to central lobe length 0.60–1.34, margins entire, thickened, often red (4/12), primary veins 3, diverging and branching above base, laminar nectaries present or absent (rare), (0-)4(-7), submarginal, associated with the minor veins of the abaxial surface, 0.3–1.1 mm in diameter, elliptic, sessile; tendril 0.3–1.1 mm wide, present at flowering node, absent in inflorescence. Flowers borne in leaf axils or terminal inflorescences; inflorescences 11.6–19.2 cm long, associated reduced laminas 7.3–11.3 mm long, 0.8–1.9 mm wide. Pedicels 7.5–25.0 mm long, 0.6–1.3 mm wide, paired in the leaf axils, often red (5R 4/12); bract(s) absent; spur(s) absent. Tubular flowers 5.1–8.6 mm in diameter with stipe 4.5–11.4 mm long, 0.9–1.5 mm wide, greenish yellow (5GY 8/6); hypanthium 5.1–8.6 mm in diameter; sepals 20.5–30.1 mm long, basally connate 5.8–15.4 mm, 1.3–3.9 mm wide, linear to narrowly ovate, acute to rounded, abaxially and adaxially greenish yellow (5GY 8/6), free portions of sepals reflexed at anthesis; coronal filaments in 1 series, adnate to the calyx tube until they become free, 36–50, 2.2–4.0 mm long, basally connate 1.2–2.5 mm, 0.1–0.4 mm wide, linear to narrowly ovate, erect, greenish yellow, ratio of coronal (portion not adnate to sepal) to sepal (free portion) length 0.09–0.35; rarely a trace second coronal row of colorless filaments may be present just outside the operculum; operculum 3.0–4.6 mm long, plicate, greenish yellow, the margin with narrow minutely fimbrillate teeth; nectary 0.3–3.1 mm high, 1.1–2.0 mm wide, sulcate; limen erect, 0.8–1.7 mm high, 0.1–0.5 mm wide, greenish yellow, crenulate-lobed, very close to the base of the androgynophore, limen floor 0.6–2.1 mm in diameter, greenish yellow; androgynophore 17.4–26.1 mm long, 0.6–1.1 mm wide, greenish yellow; free portions of the staminal filaments 2.6–5.3 mm long, 0.3–0.7 mm wide, linear, greenish yellow; anthers 4.0–5.9 mm long, 0.6–2.4 mm wide, pollen presented laterally; styles 3.1–6.2 mm long including stigmas, 0.2–0.5 mm wide, greenish yellow; stigmas 0.9–1.7 mm in diameter; ovary 2.2–5.3 mm long, 1.1–3.5 mm wide, ellipsoid to fusiform, greenish yellow, glabrous. Berry 15.5–24.0 mm long, 12.9–19.0 mm in diameter, fusiform to ovoid, very dark purple. Seeds 39–53, 4.0–5.0 mm long, 2.4–3.6 mm wide, 1.4–2.0 mm thick, flattened, obovate in outline, acute at both ends, reticulate-foveate with each side marked with 15–18 foveae, sometimes pale brown in color at maturity. Germination epigeal.

**Figure 44.**
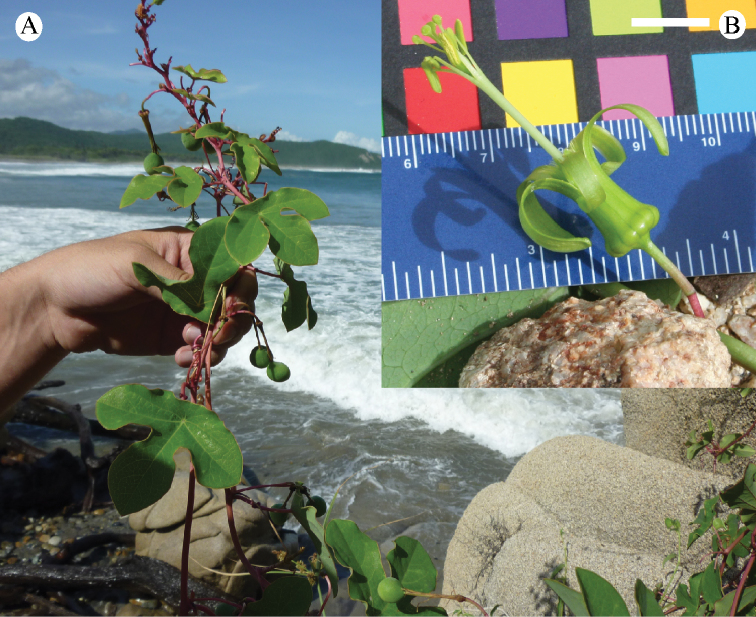
Habit, flowers, and fruits of *Passiflora viridiflora* (*Porter-Utley et al*., *500*) **a** Habit **b** Flower. Scale bar = 10.0 mm.

##### Phenology.

Flowering and fruiting throughout the year.

##### Distribution.

Mexico, in the Pacific lowlands and foothills of southern Michoacán, Guerrero and Oaxaca. Disturbed tropical deciduous or semideciduous low and medium forests (selva baja caducifolia and selva mediana subcaducifolia); growing on shrubs, small trees, boulders and rocks (sometimes limestone) on very limited to moderately developed soils; sea-level to ca. 610 m.

##### Discussion.

Vegetatively, *Passiflora viridiflora* and *Passiflora juliana* are very similar, and the most obvious difference between them is the size and shape of their stipules. However, *Passiflora viridiflora* also differs from *Passiflora juliana* in its adaptations for hummingbird pollination including: vegetative parts that are commonly accentuated with or entirely bright red, a greatly elongated androgynophore that far exceeds the length of the stamen filaments, no inner coronal filaments, a very narrow limen floor, wide floral nectary, long operculum that is not incurved at the margin but erect and lays against the androgynophore, fused sepals that are greatly elongated, pollen that is presented laterally, and a sulcate floral nectary floor.

On an herbarium specimen collected by W.L. Forment (*1125*), he indicated that *Passiflora viridiflora* is utilized by hummingbirds, which is consistent with its floral morphology and lack of floral fragrance.

*Passiflora viridiflora* has been placed at various generic (e.g., *Murucuia*) and infrageneric levels (e.g., subg. *Chloropathanthus*) within the family Passifloraceae. The elongated, tubular flowers of this taxon inspired many previous workers to group it with other taxa that possess tubular flowers or in a group of its own because the flowers are not only tubular but also apetalous. Killip (1938) placed it in the subgenus *Chloropathanthus* with *Passiflora lancifolia*, an apetalous Jamaican endemic. MacDougal (1983) was the first to suggest that *Passiflora viridiflora* be placed within *Cieca* based upon its apetalous flowers and flavonoid chemistry. In 1992, MacDougal resolved the placement of *Passiflora viridiflora* by describing *Passiflora juliana*, a species clearly referable to *Cieca* and morphologically similar to *Passiflora viridiflora*. Both the molecular and morphological data in this study also show that *Passiflora juliana* and *Passiflora viridiflora* are sister species.

##### Specimens examined.

**MEXICO. Guerrero:** above Hotel Papagayo, 1 mi. E of Acapulco, *Barkley 14062* (F, TEX); Mpio. Zihuatanejo, Playa Majahua, W de Bahía de Zihuatanejo, 17 40'N, 101 34'W, 30 m, *Castillo & Zamora 6302* (XAL); Mpio. Zihuatanejo, Cerro el Rialito, base O entre punta Ixtapa & el Rialito, *Castillo et al. 6599* (XAL); 2.5 km W Puerto Marques, *Forment 1125* (UC, XAL); Dist. Galeana, Atoyac, 20 m, *Hinton 10999* (GH, US); Mpio. Acapulco, Cascada de Chorro, 73 km S de Chilpancingo por la carretera a Acapulco, 280 m, *Koch et al. 79191* (DUKE, NY); Mpio. Acapulco, 3 km W de Cuarenta y Dos, 27 km N de Acapulco (Glorieta Diana) sobre la terraceria al la Estación de Microondas 42 & La Providencia, 610 m, *Koch et al. 79221* (CHAPA, DUKE); Mpio. Tecpan, 22 km W de San Luis de La Loma, a 3 km S de Papanoa, carr. Acapulco-Zihuatanejo, *Ladd 212* (CAS, MO); Mpio. Acapulco, Acapulco-Pinotepa Nacional, km 32 E de Acapulco, *Martínez & Tellez 87* (CAS, HUA, MO); along road to El Tamarindo, 6 km from Mex Hwy. 200 between Acapulco and San Marcos, 140 m, *Miller & Tenorio 567* (MO); between Juchitán & Ometepec, 300–1000 ft., *Nelson 2317* (US); Side of Hwy. 200 between Lazaro Cardenas and Zihuatanejo, 50 m, *Porter-Utley & Mondragón 366* (FLAS); Side of Hwy. 200 overlooking the ocean between Petatlán and Atoyac Alvarez, 20 m, *Porter-Utley & Mondragón 371* (FLAS); Side of Hwy. 200 between Acapulco and San Marcos, 50 m, *Porter-Utley & Mondragón 374* (FLAS); 3 km NE de Coyuquilla, Mpio. Petatlán, 90 m, *Soto et al. 12503* (F); Mpio. Zihuatanejo, 15 km NE de Zihuatanejo, por la carretera Zihuatanejo-Ciudad Altamirano, 70 m, *Tenorio et al. 384* (MO). **Michoacán:** Hwy. 200 between El Faro and Maruata, 20 m, 18°18.31N, 103°25.60W, *Porter-Utley & Mondragón 362* (FLAS); 4 km NE de Playa Azul, carr. a Nueva Italia, 150 m, *Soto Nuñez & Boom 2101* (US); 8 km NW de Caleta de Campos, Mpio. de Lazaro Cardenas, 40 m, *Soto Nuñez & de Soto 3756* (CHAPA, MO, XAL). **Oaxaca:** E and below La Soledad, *Ernst 2561* (US); Dist. Jamiltepec, a 6 km NW de Pinotepa Nacional por carretera a Acapulco, 240 m, *Hernández & Torres 431* (MO); Dist. Juquila, Puerto Escondido, 0.1 m N of Rt. 200, *MacDougal 349* (DUKE, US); Dist. Juquila, Puerto Escondido, 150 m SE down coast from town, on rocky peninsula 15 m above ocean, *MacDougal 351* (CHAPA, DUKE, US); between Mixtepec & Colotepec, 250-800 ft., *Nelson 2446* (GH, US); the Pacific coast, just W of Puerto Escondido in the Carrazillo Trailer Park, sea-level, *Taylor 2663* (DUKE); 27 km SW del Morro Mazatlán, carr. Salina Cruz-Pochutla, Dist. de Tehuantepec, *Torres et al. 549* (DUKE).

#### 13. Passiflora mcvaughiana

Taxon classificationPlantaeMalpighialesPassifloraceae

J. M. MacDougal. Novon 11: 69–75. figs 1 & 2. 2001.

Figs 45 , 46

##### Type.

Mexico. México: Mpio. Temascaltepec, N of Temascaltepec on rte. 134, ca. 11 mi. S of road to Tequesquipán, 6200 ft., oak woods, 24 Aug. 1978, *J. M. MacDougal 369* (holotype: DUKE!; isotypes: IBUG, MEXU [MEXU00438950, photograph seen], MICH [MICH1210192, photograph seen]).

##### Description.

Slender, low-climbing or trailing, perennial vine 2–8 m long or more, sparsely pubescent with unicellular curved trichomes on petiole, leaf, stem, and stipule (rare), 0.3–0.6 mm long, 0.02 mm wide, also minutely antrorsely appressed-puberulent throughout (except ovary) with unicellular, curved trichomes, 0.1–0.3 mm long, 0.02–0.03 mm wide. Flowering stems 0.9–2.6 mm in diameter, terete or somewhat compressed, with the base woody and cork-covered. Stipules 3.8–7.5 mm long, 0.3–0.8 mm wide, narrowly ovate, acute to slightly attenuate, longitudinally striate-nerved; petioles 0.8–5.3 cm long, inserted 1.1–6.9 mm from the basal margins of the peltate blades, eglandular or very rarely with 1 or 2, round or elliptic, opposite to subopposite, sessile or shortly stipitate, discoid nectaries with flat rims, 0.8–1.3 mm wide (on the widest axis), 0.4–1.3 mm high, borne just below (rare) or in the distal half of the petiole (0.45–0.86 of the distance from the base toward the apex of the petiole). Laminas 0.9–7.3 cm long, 6.2–22.0 cm wide, coriaceous, occasionally variegated along primary veins, conspicuously peltate, transversely elliptic (widely divaricately bilobed), lateral lobes 3.1–12.0 cm long, 1.0–5.6 cm wide, elliptic, acute to slightly attenuate, central vein 0.7–6.8 cm long (measured from point of petiole insertion), angle between the lateral lobes 101–182(-190)°, ratio of lateral lobe to central vein length 1.15–4.57, margins entire, hyaline, primary veins 3, diverging and branching above base, laminar nectaries absent, associated with the minor veins of the abaxial surface, 0.8–1.1 mm in diameter, sessile; tendril 0.3–1.0 mm wide, present at flowering node, absent in inflorescence. Flowers borne in leaf axils or rarely in inflorescences; inflorescences 2.5–5.8 cm long, associated reduced laminas 1.3–2.3 mm long, 0.5–1.0 mm wide. Pedicels 5.6–25.0 mm long, 0.3–0.6 mm wide, (1-)2 per node; bract(s) absent or with one or two, narrowly ovate, acute bracts, 1.1–1.2 mm long, 0.1 mm wide, the bracts ca. 3.7 mm from base of peduncle; spur(s) absent. Flowers 15.0–25.3 mm in diameter with stipe 3.1–9.0 mm long, 0.5–0.7 mm wide; hypanthium 5.0–8.3 mm in diameter; sepals 6.7–8.5 mm long, 3.3–5.6 mm wide, ovate-triangular, acute, abaxially and adaxially greenish yellow, reflexed at anthesis; coronal filaments in 2 series, the outer 31–36, 3.1–7.1 mm long, 0.3–0.7 mm wide, linear to slightly narrowly obovate, erect, dull purple (5P 4/6) at base, yellow at tips, ratio of outer coronal row to sepal length 0.41–0.89, the inner 40–60, 1.3–2.9 mm long, 0.1–0.3 mm wide, linear, capitate, erect, greenish yellow speckled with dull purple, ratio of inner coronal row to outer coronal row length 0.20–0.56; operculum 1.0–1.8 mm long, plicate, whitish green, the margin with narrow minutely fimbrillate teeth; nectary 0.2–1.1 mm high, 0.6–1.0 mm wide; limen inclined away from androgynophore, 0.2–0.7 mm high, 0.2–0.5 mm wide, whitish green, speckled with purple, limen floor 2.5–4.9 mm in diameter, whitish green, speckled with purple; androgynophore 1.5–4.7 mm long, 0.9–1.3 mm wide; free portions of the staminal filaments 2.1–3.3 mm long, 0.4–0.8 mm wide, linear, greenish yellow anthers 1.7–3.2 mm long, 0.7–1.7 mm wide, greenish yellow with a dark purple edge; styles 2.7–4.9 mm long including stigmas, 0.3–0.5 mm wide, greenish yellow; stigmas 0.6–1.3 mm in diameter; ovary 1.3–3.6 mm long, 1.1–3.0 mm wide, widely ellipsoid to ovoid, greenish yellow. Berry 10.0–14.4 mm long, 12.8–13.8 mm in diameter, globose, very dark purple. Seeds (2-)6–11, 4.8–5.5 mm long, 3.6–4.1 mm wide, 2.1–2.7 mm thick, widely elliptic to widely obovate in outline, obtuse at both ends, reticulate-foveate with each face marked with 15–22 foveae.

**Figure 45.**
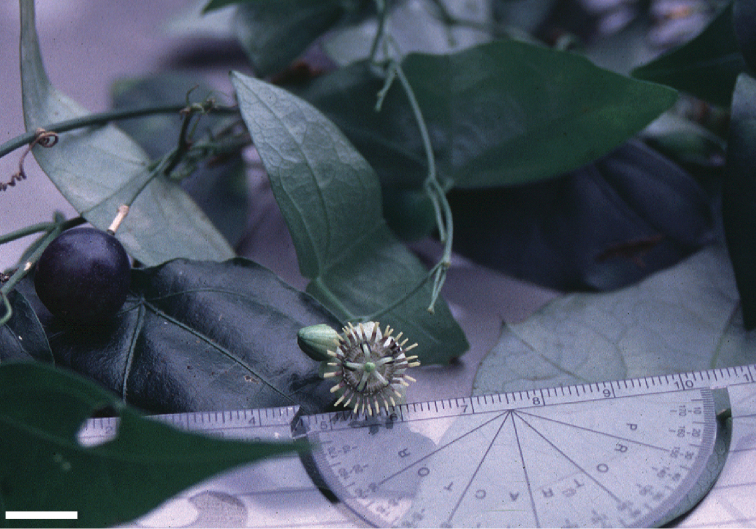
Leaves, flower and fruit of *Passiflora mcvaughiana* (*Porter-Utley & Mondragón 345*) Scale bar = 10.0 mm.

**Figure 46.**
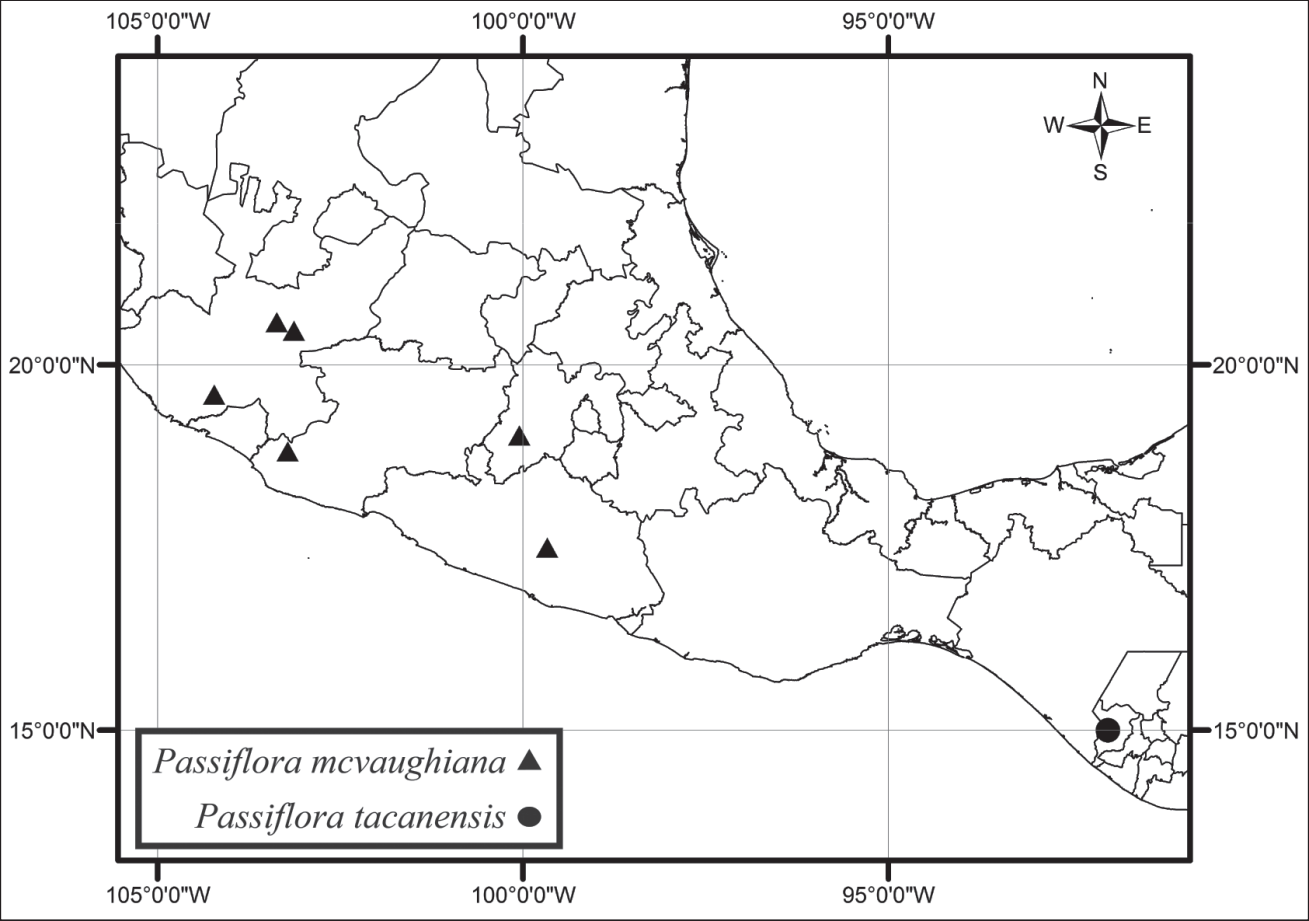
Distribution of *Passiflora mcvaughiana* and *Passiflora tacanensis*.

##### Phenology.

Flowering and fruiting June to December.

##### Distribution.

Mexico, in the states of Jalisco, Mexico, and Guerrero. Pine and oak forests (bosque de pino y encino) or montane mesophytic forests (bosque mesófilo de montaña); growing in trees and on the steep banks of canals (barrancas) or streams, and moist hillsides; 1100–2000 m.

##### Discussion.

*Passiflora mcvaughiana* is one of four species found in Mexico previously known under the name of *Passiflora coriacea* Juss. The other two species are *Passiflora obtusifolia* and *Passiflora tacanensis*, which are both extremely similar vegetatively to *Passiflora mcvaughiana*. *Passiflora mcvaughiana* can usually be separated from *Passiflora obtusifolia* because *Passiflora mcvaughiana* commonly has a central leaf lobe that is nearly as long as the lateral lobes at fertile nodes, as opposed to having lateral lobes that are commonly twice as long as the central lobe in *Passiflora mcvaughiana*. *Passiflora obtusifolia* is commonly 3-lobed more than 0.20 the distance to the base, as opposed to 3-lobed less than 0.20 the distance to the base in *Passiflora mcvaughiana*. *Passiflora mcvaughiana* lacks laminar nectaries, whereas *Passiflora obtusifolia* commonly has 2–6 nectaries present between the primary leaf veins. Flowers are rarely produced in inflorescences in *Passiflora mcvaughiana*, but *Passiflora obtusifolia* commonly has very long inflorescences. The pedicel in *Passiflora mcvaughiana* is longer than 10 mm, but the pedicel in *Passiflora obtusifolia* is commonly less than 10 mm long. The outer coronal filaments of *Passiflora mcvaughiana* are longer than 4.0 mm, linear, and dull purple toward their bases, those of *Passiflora obtusifolia* are commonly less than 4.0 mm long, linear/capitellate and greenish yellow or greenish yellow with a flush of reddish purple at the base. *Passiflora mcvaughiana* possesses the widest seeds in the supersection (over 3.6 mm wide) and *Passiflora obtusifolia* has seeds that are less than 2.3 mm wide. Additionally, *Passiflora mcvaughiana* and *Passiflora obtusifolia* occupy different habitats, with *Passiflora obtusifolia* commonly occurring in lower elevation tropical deciduous or semideciduous forests in Pacific lowlands and foothills and *Passiflora mcvaughiana* in high elevation oak, pine/oak, pine or montane mesophytic forests of Mexico.

*Passiflora tacanensis* is a newly discovered species from Volcán Tacaná, Chiapas, Mexico. Like *Passiflora mcvaughiana*, it occurs in montane mesophytic forests. However, *Passiflora tacanensis* is easily separated from *Passiflora mcvaughiana* by its foliose stipules that are more than 3 mm wide. The fruits of *Passiflora tacanensis* also possess ca. 20 seeds, whereas those of *Passiflora mcvaughiana* produce only 2–10 seeds.

*Passiflora mcvaughiana* is also quite similar vegetatively to *Passiflora sexocellata*, though this species does not occur in southwestern Mexico. The leaves of *Passiflora mcvaughiana* are not as coriaceous as those of *Passiflora sexocellata* and are darker green. In addition, *Passiflora sexocellata* always possesses 4–13 laminar nectaries while *Passiflora mcvaughiana* has none. The petiolar nectaries of *Passiflora sexocellata* are commonly positioned on the proximal half of the petiole, whereas those of *Passiflora mcvaughiana* are positioned on the distal half of the petiole. Flowers are often produced in long inflorescences in *Passiflora sexocellata*, and *Passiflora mcvaughiana* commonly lacks inflorescences. The fruits of *Passiflora sexocellata* are also much larger than those of *Passiflora mcvaughiana* and possess between 40 and 50 seeds per fruit.

##### Specimens examined.

**MEXICO. Jalisco:** cañada que sube al Filo de la Vaca, por la toma de agua, El Zarzamoro, 1980 m, *Cuevas & Guzman 4198* (CHAPA); San Sebastian, trail to El Ranchito, 1500 m, *Mexia 1448* (CAS, F, US). **México:** Temascaltepec, Rincón, 1960 m, *Hinton 3030* (BM,US); Temascaltepec, Rincón, 2000 m, *Hinton 4655* (BM); Hwy. 134 between Temascaltepec and Tejupilco, 1760 m, 19°02.46N, 100°02.95W, *Porter-Utley & Mondragón 345* (CICY, FLAS); Hwy. 134 between Temascaltepec and Tejupilco, seedling, 1760 m, 19°02.46N, 100°02.95W, *Porter-Utley & Mondragón 346* (CICY, FLAS). **Locality Unknown:** *Sessé & Mociño 4457* (AAU, F); *Sessé & Mociño 4458* (F).

#### 14. Passiflora tacanensis

Taxon classificationPlantaeMalpighialesPassifloraceae

K. Porter-Utley. Brittonia 59(1): 25. figs 1 & 2. 2007.

Figs 46 , 47

##### Type.

Mexico. Chiapas: Mpio. Unión Juárez, Volcán Tacaná, entre Talquián & Toniná, 1700–2700 m, 7 May 1987, *E. M. Martínez S. 20782* (holotype: MEXU! [MEXU00665952]).

##### Description.

Vine, pubescent with unicellular curved trichomes on petiole, adaxial leaf surface, and stipules 0.28–0.38 mm long, 0.03 mm wide, also minutely antrorsely appressed-puberulent throughout with unicellular, curved trichomes, 0.06–0.08 mm long, 0.02 mm wide. Flowering stems 1.5–1.8 mm in diameter, terete or somewhat compressed. Stipules 6.3–7.5 mm long, 2.5–3.5 mm wide, ovate, acute to acuminate; petioles 2.3–2.6 cm long, commonly bearing at or just below the middle (0.44–0.50 of the distance from the base toward the apex of the petiole) 2, elliptic, opposite to subopposite, sessile, discoid nectaries with the rims slightly raised, 1.0–1.1 mm wide (on the widest axis), 0.5–0.6 mm high. Laminas 3.8–5.3 cm long, 12.4–14.2 cm wide, membranous, subpeltate or slightly peltate (the distance from leaf base to point of petiole insertion 1.0–1.7 mm), transversely elliptic, 3-lobed 0.02–0.10 of the distance to the leaf base at the deepest sinus, lateral lobes 6.9–7.5 cm long, 2.6–4.2 cm wide, elliptic, acute to attenuate, central lobe elliptic or present as an obtuse to rounded tip, central vein 3.7–5.2 cm long (measured from point of petiole insertion to the leaf apex), angle between the lateral lobes 109–130°, ratio of lateral lobe to central vein length 1.41–1.86, margins entire, hyaline, primary veins 3, diverging and branching above base, laminar nectaries absent; tendril 0.7–0.9 mm wide, present at flowering node. Pedicels 11.9–13.0 mm long in fruit, 0.5 mm wide, paired in the leaf axils; bract(s) absent. Flowers not seen. Stipe 6.9–8.5 mm long in fruit, 0.5–0.6 mm wide. Berry 25.0–26.0 mm long, 24.0–26.0 mm in diameter, ellipsoid to globose, very dark purple. Seeds ca. 20, 4.6–4.9 mm long, 2.9–3.1 mm wide, 2.0–2.1 mm thick, obovate in outline, acute at both ends, reticulate-foveate with each face marked with ca. 22–26 foveae.

**Phenology.** Flowering and fruiting May.

**Distribution.** Mexico, in the state of Chiapas. Bosque mesófilo de moñtana (montane moist forest), 1700–2700 m altitude.

**Discussion.** *Passiflora tacanensis* is known only from the general type locality and though Martínez (*Martínez 20782*) states that the flowers are purple, the specimen does not possess flowers and I have not been able to locate any duplicates. It was found in montane mesophytic forests on Volcán Tacaná in southwestern Mexico and was collected in May during the rainy season.

*Passiflora tacanensis* is very similar to *Passiflora eglandulosa*, which grows on adjacent volcanic cones in San Marcos, Guatemala. The mature leaves of *Passiflora tacanensis* greatly resemble the juvenile leaves of *Passiflora eglandulosa* and are trilobed, with the middle lobe greatly reduced and widely obtuse to truncate. The laminae are also cordate and eglandular, and both of these species possess wide foliose stipules. However, *Passiflora tacanensis* possesses petiolar glands positioned near the middle of the petiole, whereas *Passiflora eglandulosa* does not possess petiolar glands. In addition, the fruits of *Passiflora tacanensis* possess more seeds and the chalazal and micropylar ends of the seed are inclined toward the raphe.

**Specimens examined. MEXICO. Chiapas:** Mpio. Tapachula, Volcán Tacaná. On trail between Talquián and the border of Guatemala, 1901 m, *Porter-Utley et al. 436* (KESC); Mpio. Tapachula, Volcán Tacaná. On trail between Talquián and the border of Guatemala, 1857 m, *Porter-Utley et al. 441* (KESC).

**Figure 47.**
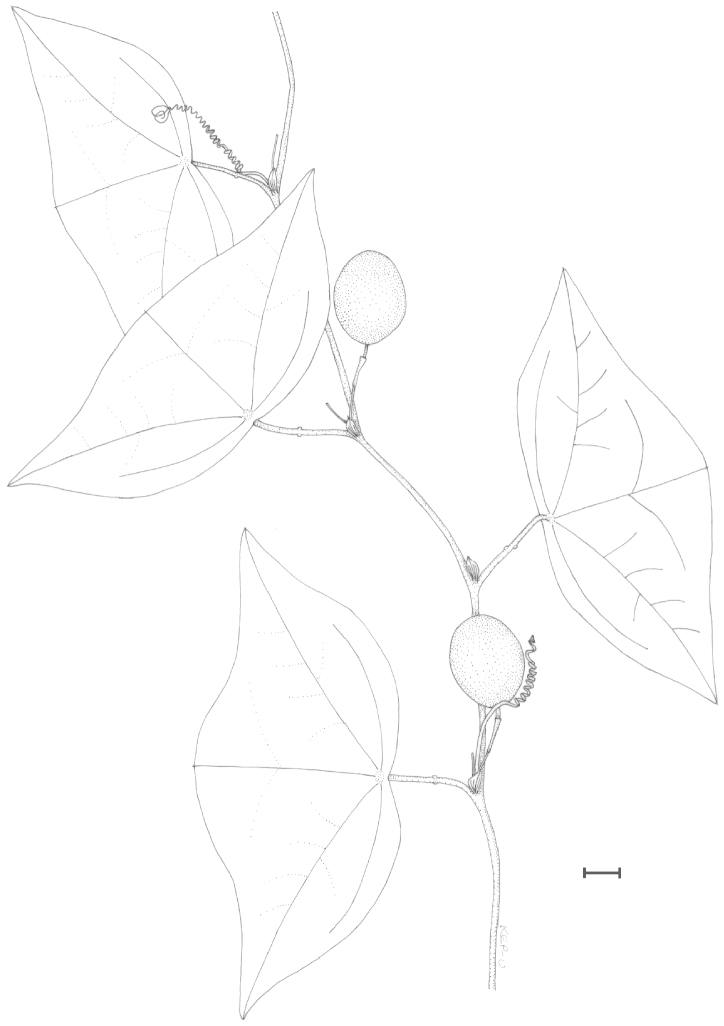
Habit of *Passiflora tacanensis* (based upon *Martínez 20782*) Scale bar = 1.0 cm.

#### 15. Passiflora coriacea

Taxon classificationPlantaeMalpighialesPassifloraceae

Juss., Ann. Mus. Natl. Hist. 6: 109. pl. 39, fig. 2. 1805.

Figs 48 , 49

Monactineirma coriacea (Juss.) Bory, Ann. Gén. Sci. Phys. 2: 138. 1819. Type: Based on *Passiflora coriacea* Juss.
Cieca coriacea (Juss.) M.Roemer, Prospect Fam. Nat. Syn. Monogr. 2: 148. 1846. Type: Based on *Passiflora coriacea* Juss.
Passiflora clypeata Sm., Cycl. [A. Rees] (London ed.) 26: *Passiflora* no. 20. 1814. Type: Colombia. Sin. loc., *J. Mutis s.n.* (lectotype, designated by Killip 1938, pg. 85: LINN 1070.16 [microfiche seen]).
Passiflora difformis Kunth in Humboldt, Bonpland and Kunth. Nov. Gen. Sp. 2: 136. 1817. Type: Colombia. Quindio: “in monte Quindiu juxta El Moral, alt. 1065 hex”, *A. Humboldt & A. Bonpland s.n.* (holotype: P [P00307399, photograph seen], photograph AAU!, isotype: B, destroyed, P [P00307391, photograph seen], photograph AAU!).
Cieca difformis (Kunth) M.Roem., Prospect Fam. Nat. Syn. Monogr. 2: 140. 1846. Type: Based on *Passiflora difformis* Kunth
Passiflora cheiroptera Cortés, Fl. Colomb. ed. 2, fig between pages 112 and 113. 1919. Type: Colombia, (lectotype, designated here: Cortés, Fl. Colomb. ed. 2, fig between pages 112 and 113. 1919).

##### Type.

Colombia. Tolima: Santa Fé, near Honda, *A. Humboldt & A. Bonpland s.n.* (lectotype, designated here: P! [P00307401], photographs AAU!, DUKE!, isolectotype: P! [P00307391], photograph AAU!).

**Description.** Slender, climbing, perennial vine 2–8 m long or more, sparsely pubescent with unicellular curved trichomes on petiole, leaf, stem and stipule, 0.20–0.64 mm long, 0.02–0.03 mm wide, also minutely antrorsely appressed-puberulent throughout with unicellular, curved trichomes, 0.03–0.10 mm long, 0.02–0.03 mm wide. Flowering stems 1.0–2.9 mm in diameter, greenish yellow (5GY 8/4) to reddish purple (5RP 4/6), terete to somewhat compressed, with the base woody and cork-covered. Stipules 2.6–7.5 mm long, 0.4–1.0 mm wide, narrowly ovate-triangular, acute; petioles 1.1–4.3 cm long, with 2 (rarely 3), opposite to subopposite, sessile, discoid nectaries with flat rims, 1.1–2.1 mm wide (on the widest axis), 0.1–1.5 mm high, borne in the proximal two thirds of the petiole (0.21–0.64 of the distance from the base toward the apex of the petiole). Laminas 2.8–5.9 cm long, 6.2–18.8 cm wide, coriaceous, peltate (the distance from leaf base to point of petiole insertion 1.6–11.4 mm), transversely elliptic (widely divaricately bilobed) or sometimes 3-lobed, lateral lobes 3.3–9.5 cm long, 1.8–7.3 cm wide, elliptic, acute to attenuate, central lobe elliptic to obovate or present merely as a widely acute to obtuse tip (rarely retuse), central vein 1.8–6.4 cm long (measured from point of petiole insertion to the leaf apex), angle between the lateral lobes (97-)110–160(-170)°, ratio of lateral lobe to central vein length 1.36–2.61, margins entire, hyaline, primary veins 3, diverging and branching above base, laminar nectaries present, 5–13, submarginal, associated with the minor veins of the abaxial surface, with 2–4 nectaries proximal to the lateral leaf veins, 0.7–1.3 mm in diameter, circular to widely elliptic, sessile; juvenile leaves bilobed and variegated; tendril 0.3–0.9 mm wide, present at flowering node, absent in inflorescence. Flowers borne in leaf axils or inflorescences; inflorescences 2.5–6.5(-12.0) cm long, associated reduced laminas 2.5–5.0 mm long, 1.5–2.8 mm wide. Pedicels 2.2–8.1 mm long, 0.4–1.1 mm wide, 2 per node; bract(s) absent; spur(s) absent. Flowers 18.0–30.0 mm in diameter with stipe 6.3–15.1 mm long, 0.7–1.0 mm wide; hypanthium 4.9–7.4(-8.1) mm in diameter; sepals 5.8–10.9 mm long, 3.3–6.4 mm wide, ovate-triangular, acute to rounded, abaxially and adaxially greenish yellow (5GY 8/4); coronal filaments in 2 series, the outer (36-)49–53, 3.1–5.3(-7.0) mm long, 0.2–0.5 mm wide, linear, spreading, dark reddish purple at base (5RP 3/6–4/6), medium reddish purple just below the middle (5RP 4/4–5/4), light reddish purple (5RP 6/6–6/8) just above middle and white on the distal third, ratio of outer coronal row to sepal length 0.43–0.76, the inner 33–50, 1.4–3.2 mm long, 0.2–0.5(-0.7) mm wide, linear, capitate, erect, dark reddish purple (5RP 3/6), lightening slightly towards tips, ratio of inner coronal row to outer coronal row length (0.29-)0.44–0.63(-0.72); operculum 1.3–2.0(-4.3) mm long, plicate, reddish purple (5RP 3/6–4/6), the margin with narrow minutely fimbrillate teeth; nectary 0.2–0.5(-1.5) mm high, 0.7–1.1 mm wide; limen recurved or sometimes erect, 0.2–0.5(-0.7) mm high, 0.1–0.4 mm wide, reddish purple (5RP 3/6–4/6), limen floor 2.0–3.5(-4.7) mm in diameter, pale greenish yellow with reddish purple (5RP 3/6–4/6) spots and streaks; androgynophore (3.3-)3.8–5.0 mm long, 1.0–1.5 mm wide, pale greenish yellow (5GY 8/2) with reddish purple (5RP 3/6–4/6) spots and streaks; free portions of the staminal filaments 2.4–3.2 mm long, 0.5–1.1 mm wide, linear, greenish yellow; anthers 1.9–3.9 mm long, (0.6-)0.9–2.3 mm wide; styles 3.2–4.6 mm long including stigmas, 0.2–0.5 mm wide, greenish yellow; stigmas 0.6–2.2 mm in diameter; ovary 1.7–2.9 mm long, 1.5–2.5(-4.0) mm wide, widely ellipsoid to globose, greenish yellow. Berry 17.1–21.0 mm long, 12.0–19 mm in diameter, globose, very dark purple (5P 2.5/2). Seeds ca. 44–61, 3.6–4.0(-5.0) mm long, 2.1–2.5 mm wide, 1.5–1.8 mm thick, obovate in outline, acute at both ends, reticulate-foveate with each side marked with ca. 15–17 foveae.

**Figure 48.**
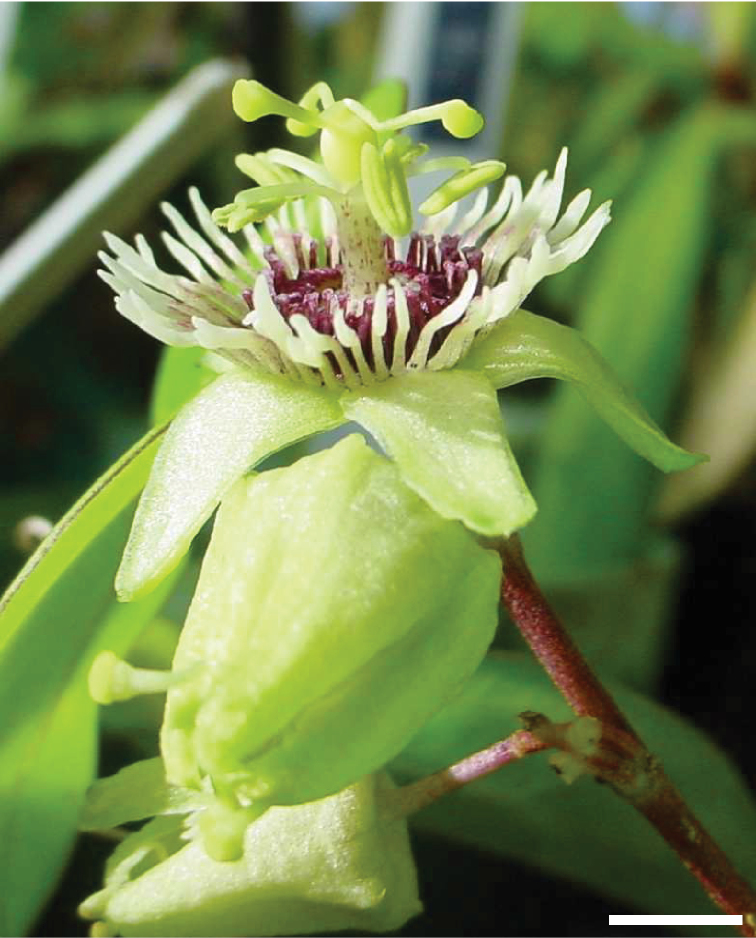
Flower of *Passiflora coriacea* from Colombia. Scale bar = 5.0 mm. Photo by C. Feuillet.

**Figure 49.**
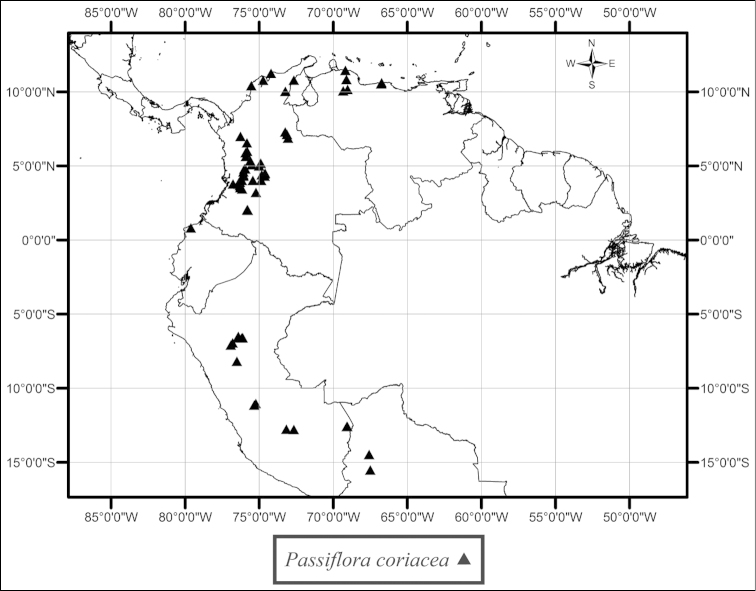
Distribution of *Passiflora coriacea*.

##### Phenology.

Flowering and fruiting throughout the year.

##### Distribution.

Bolivia, Colombia, Ecuador, Perú, and Venezuela; reported once from Guyana (*Lejos 43*, B, destroyed). Growing in shrubs and small trees in secondary successional areas, along the edges of moist tropical forests near rivers and streams, and along the seashore, 0–1500 m.

##### Ethnobotany.

Timothy Plowman in a note on a specimen collected by him in 1976 (*T. Plowman 6029*), noted that in Perú a medicine for the liver is prepared from *Passiflora coriacea* by boiling the whole plant and then drinking the syrup.

##### Discussion.

*Passiflora coriacea* is extremely similar to *Passiflora sexocellata* and *Passiflora megacoriacea* in its vegetative morphology, but is easily distinguished by its flowers. The flowers of *Passiflora coriacea* possess long floral stipes as compared to their pedicels (the stipes are usually two to three times the length of the pedicels) and an operculum that is dark reddish purple. *Passiflora sexocellata* has floral stipes that are commonly shorter than or equal in length to the pedicels and an operculum that is greenish yellow with a flush of dark reddish purple at the base and a white margin. *Passiflora megacoriacea* possesses floral stipes that are commonly less than half the length of the pedicels and an operculum that is greenish yellow with a white margin or greenish yellow with a mere flush of reddish purple at the base and a white margin. *Passiflora coriacea* is also distinguished by outer coronal filaments that may appear banded with light to dark reddish purple. In addition, the outer coronal filaments are more dilated distally, much like *Passiflora megacoriacea* but in contrast to *Passiflora sexocellata*. The limen floor in *Passiflora coriacea* is very light greenish yellow with dark reddish purple spots and streaks, again much like *Passiflora megacoriacea*. *Passiflora sexocellata* usually possesses a very dark red limen floor.

Schlechtendal (1854) attempted to use mostly vegetative characters to distinguish *Passiflora coriacea* from *Passiflora sexocellata*; incidentally, he was the first to notice differences in the stipe and pedicel lengths of the two species. He used the following characters to differentiate them: position of the petiolar nectaries, the number of laminar nectaries, the shape of the stem, leaf venation, the leaf margin, and the leaf texture. However, in my analysis of these species, I did not find any of these vegetative characters to be wholly reliable in distinguishing between these two species. Both have petiolar nectaries that occur in various positions below the middle of the petiole, stems that are terete to somewhat compressed, five distinct leaf veins, thick leaf margins and coriaceous leaves. *Passiflora sexocellata* does tend to have fewer nectaries than *Passiflora coriacea* on average, but there is a significant amount of overlap in the range of variation.

A clone of *Passiflora coriacea* (*MacDougal 3029*) did not produce fruits by autogamy in years in cultivation. This greenhouse accession was given to me by MacDougal, who originally received it as seedlings from J. Zarucchi (*Zarucchi et al. 6102*).

*Heliconius erato* (Lepidoptera: Nymphalidae, Heliconiinae) has been reported to be an herbivore of *Passiflora coriacea* in the central Colombian valleys (Cauca and Magdalena) (Benson et al. 1975).

Fajardo et al. (1998) in a study on the genetic variation analysis of the genus *Passiflora* using RAPD markers, used *Passiflora coriacea* and *Passiflora adenopoda* DC. as representatives of taxa from subgenus *Decaloba*. They found *Passiflora coriacea* to be genetically distant from the other taxa in his study, including *Passiflora adenopoda*, but due to insufficient data, they were not able to discuss the significance of this result (Fajardo et al. 1998).

In Antoine Laurent de Jussieu’s original description of *Passiflora coriacea* (1805) he included a detailed diagnosis and drawing of the species. The lectotype of *Passiflora coriacea* (at P), closely resembles the drawing in Jussieu, but there are no locality data on the specimen. The isolectotype of *Passiflora coriacea* consists of two leaves and a small portion of the stem and does not resemble the type drawing of the species, but written on the specimen are locality and descriptive data in Jussieu’s hand.

##### Selected specimens.

**BOLIVIA. La Paz:** Prov. Alto Beni, Chaco, cerca de Santa Ana de los Mozetenes, 450 m, *Seidel & Schulte 2525* (TEX).

**COLOMBIA. Antioquia:** Mpio. Salgar, along road to Salgar, 4 km from Bolombolo, Bolívar Road, 900 m, *Zarucchi et al. 6102* (HUA, MO). **Bolívar:** vicinity of Cartagena, *Heriberto 392* (US). **Caldas:** Entre Aranca & Manizales 35 km de Manizales, 1500 m, *Escobar & Uribe 483* (HUA, LL). **Chocó:** Mpio. Ríosucio, Parque Nal. Nat. Los Catios, camino Tilupo Peye, Quebrada Peye, 40 m, *Forero et al. 1770* (MO). **Cundinamarca:** Población de Nariño, bosque donde finaliza la carretera de los Mangos, 350–450 m, *Fernández et al. 5480* (MA). **Huila:** about 5 km N of Villavieja; upper basin of Río Magdalena, 500 m, *Mason 13808* (UC). **Magdalena:** about 8 km N of Codazzi, 250 m, *Haught 3752* (S, US); Flanco N de la Sierra Nevada de Santa Marta, *Romero 761* (US). **Santander:** River Suratá valley, between Bucaramanga and El Jaboncillo, 800–1500 m, *Killip & Smith 19062* (GH, US). **Tolima:** Río Cuello, New Quindío Trial, Cordillera Central, 1000–1500 m, *Hazen 9652* (GH, US). **Valle:** Mpio. Tuluá, Corr. Mateguada, Jardín Botánico, 1100 m, *Escobar 1045* (HUA); Mpio. Yotoco, ingenio La Carmelita, sección San Martín, zona A, dentro de un guadual que esta en medio de la cana, mas o menos una hora de Mediacanoa, 950 m, *Ramos et al. 2811* (MO).

**ECUADOR. Esmeraldas:** El Timbre, towards Esmeraldas, *Asplund 16532* (AAU, S).

**PERÚ. Cuzco:** La Convencion, N bank of Río Alto Urubamba, across from village of Kiteni, 500 m, *Knapp & Mallet 6356* (US). **Madre De Dios:** Prov. Tambopata, Cuzco Amazónico, across Río Madre de Dios on road to Lago Sandoval, 200 m, *Gentry & Curso de Posgrado de la Universidad de San Marcos 68962* (F, MO). **San Martín:** Prov. Mariscal Caceres, Dtto. Tocache Nuevo, *Schunke 3823* (F, GH, MO, US).

**VENEZUELA. Distrito Federal.** Cerro Naiguatá, slopes near the sea to the N, above the town of Naiguatá, Lomas de Las Delicias, between Quebrada Basenilla and Quebrada Guayoyo, 9–12 km SE of Hacienda Cocuizal, 1000–1300 m, *Steyermark 92078* (US). **Falcón:** Parque Nacional Quebrada de la Cueva El Toro, trail going to La Piedra, 600–900 m, *Liesner et al. 7831* (MO, VEN). **Lara.** E border near state of Yaracuy, Guaremal River, NE of Barquisimeto, *Meijer & Smith 56* (MO). **Yaracuy.** Dist. Urachiche, Quebrada Higueronal, afluente del Río Urachiche, W de Urachiche, cerca de la Sabana de Mendez, 50 m, *Steyermark et al. 124671* (VEN). **Zulia.** Dist. Mara, alrededores del Puesto “El Bosque” de la Guardia Nacional, 1450–1600 m, *Bunting et al. 12264* (MO).

**CULTIVATED MATERIAL. United States.** Missouri, cultivated at the Missouri Botanical Garden, from material collected by J.L. Zarucchi (*Zarucchi 6102*) in Antioquia, Colombia, *MacDougal 3029* (FLAS, MO); Florida, cultivated at the University of Florida from material collected by J.L. Zarucci (*Zarucchi 6102*) in Antioquia, Colombia, *Porter-Utley P-66* (FLAS).

#### 16. Passiflora megacoriacea

Taxon classificationPlantaeMalpighialesPassifloraceae

K. Porter-Utley. sp. nov.

urn:lsid:ipni.org:names:77143305-1

Figs 50 , 51

##### Diagnosis.

Passiflora scandens; stipulae 0.4–0.7 mm latae; petioli in parte proximali biglandulosi; folia peltata glandulosa bilobata vel obscure ad distincte trilobata, lobis centralibus obtusis ad acutis, lobis lateralibus acutis vel raro acuminatis, marginibus integris; pedunculi ebracteatis vel raro unibracteatis, stipites florum 1.7–5.7 mm longi; petala nulla; sepala 10.0–20.5 mm longa, viridiflava; filamenta coronae 2-seriata, filamentis exterioribus linearibus, 6.8–14.0 mm longis, pro parte maxima viridiflavis, ad apicem flavidis, interdum ad basim purpureis, filamentis interioribus capitatis, 2.3–5.6 mm longis, pro parte maxima viridiflavis, ad apicem flavidis, interdum ad basim purpureis; operculum plicatum; androgynophorum 4.1–10.0 mm longum; ovarium glabrum; fructus ellipsoidei; semina 4.9–5.1 mm longi, 3.0–3.1 mm lata, retifoveata.

Type: Costa Rica. Limón: bluff above mouth of river at Moín, about 7 km N of Limon, sunny clay bank along road, 9 Aug. 1980, *J. M. MacDougal 1204* (holotype: DUKE! [DUKE00274532]; isotypes: C!, CAS! [CAS00767084]).

##### Description.

Slender, climbing, perennial vine 2–4 m long or more, sometimes trailing on ground, sparsely pubescent with unicellular curved trichomes on petiole, leaf and stem, 0.2–0.4 mm long, 0.02–0.03 mm wide, also minutely antrorsely appressed-puberulent throughout with unicellular, curved trichomes, ca. 0.1 mm long, 0.02–0.03 mm wide. Flowering stems 1.0–2.4 mm in diameter, terete or somewhat compressed, with the base woody and cork-covered. Stipules 1.7–5.7 mm long, 0.4–0.7 mm wide, narrowly ovate-triangular, acute, longitudinally striate-nerved; petioles 1.1–3.8 cm long, inserted 1.4–9.6 mm from the basal margins of the peltate blades, with two, round or elliptic, opposite to subopposite, sessile or shortly stipitate (rare), saucer-shaped nectaries with flat rims, 1.3–2.1 mm wide (on the widest axis), 0.4–1.6 mm high, commonly borne in the distal half of the petiole (0.30-)0.53–0.77 of the distance from the base toward the apex of the petiole. Laminas (2.7-)3.3–7.1(-8.2) cm long, 6.6–17.3 cm wide, sometimes glaucous beneath, coriaceous, peltate, transversely elliptic (widely divaricately bilobed) or 3-lobed, lateral lobes 3.5–9.1 cm long, 3.7–25 cm wide, elliptic, acute to slightly attenuate, central lobe elliptic to ovate or present merely as an acute to obtuse tip, central vein 2.4–7.7 cm long (measured from point of petiole insertion), angle between the lateral lobes 104–176°, ratio of lateral lobe to central vein length 0.85–2.47, margins entire, hyaline, primary veins 3, diverging and branching above base, laminar nectaries present, 6–10, submarginal, associated with the minor veins of the abaxial surface, 0.3–1.5 mm in diameter, circular to widely elliptic, sessile; tendril 0.3–1.1 mm wide, present at flowering node, absent in inflorescence. Flowers borne in leaf axils or inflorescences; inflorescences 5.6–11.7 cm long, associated reduced laminas 2.6–9.0 mm long, 1.0–2.8 mm wide. Pedicels 4.4–17.5 mm long, 0.4–1.1 mm wide, 2 per node; bract(s) 1 (rare) or absent; spur(s) absent. Flowers 29.5–56.7 mm in diameter with stipe 2.6–6.1 mm long, 0.9–1.4 mm wide; hypanthium (7.8-)8.1–16.1 mm in diameter; sepals 10.0–20.5 mm long, 4.3–12.1 mm wide, ovate-triangular, acute to rounded, abaxially and adaxially very pale greenish yellow; coronal filaments in 2 series, the outer 31–40, 6.8–14.0 mm long, 0.4–1.1 mm wide, linear, dilated toward tips, erect, greenish yellow (5GY 8/4) with very light yellow tips (5Y 8/6), ratio of outer coronal row to sepal length 0.48–0.85, the inner (12-)30–45, 2.3–5.6 mm long, 0.1–0.4 mm wide, linear, capitate, erect, greenish yellow with whitish apices or greenish yellow with a mere flush of reddish purple (5RP 4/8–4/10) at the very base and whitish tips, ratio of inner coronal row to outer coronal row length 0.30–0.52; operculum 2.1–4.2 mm long, plicate, flushed with reddish purple toward the base and whitish toward the tips, the margin with narrow minutely fimbrillate teeth; nectary 0.4–0.8(-2.3) mm high, 0.8–2.5 mm wide; limen recurved or rarely inclined slightly away from androgynophore, 0.4–1.3 mm high, 0.2–0.9 mm wide, whitish, limen floor 3.3–8.4 mm in diameter, whitish with reddish purple spots and streaks toward base; androgynophore 4.1–10.0 mm long, 0.9–1.9 mm wide, whitish at base with reddish purple spots and streaks becoming light greenish yellow toward apex; free portions of the staminal filaments 2.8–3.9 mm long, 0.5–1.3 mm wide, linear, greenish yellow; anthers 2.8–4.4 mm long, 0.8–2.6(-5.1) mm wide; styles 3.2–6.7 mm long including stigmas, 0.3–0.7 mm wide, greenish yellow; stigmas 1.2–2.5 mm in diameter; ovary 2.1–4.0 mm long, 1.4–3.6 mm wide, widely ellipsoid to globose, greenish yellow. Berry 24.0–27.0 mm long, 19.0–25.0 mm in diameter, ellipsoid, very dark purple (5P 2.5/2) with a glaucous bloom at maturity, immature fruit greenish yellow, sometimes mottled with white or yellow. Seeds (27-)45–50(-60), 4.9–5.1 mm long, 3.0–3.1 mm wide, 1.9–2.0 mm thick, obovate in outline, acute at both ends, reticulate-foveate with each face marked with ca. 15–20 foveae.

**Figure 50.**
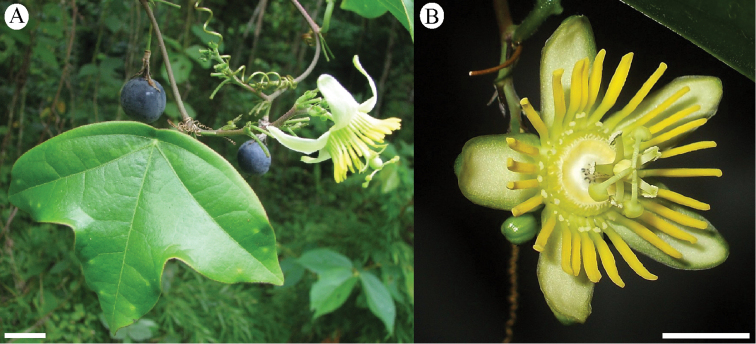
Habit and flower of *Passiflora megacoriacea*. **a** Habit (*Stapf 652*) Scale bar = 8 mm. Photo taken in Panama by M. Stapf **b** Close up of flower from plant in Costa Rica. Scale bar = 8 mm. Photo by R. Ziller.

**Figure 51.**
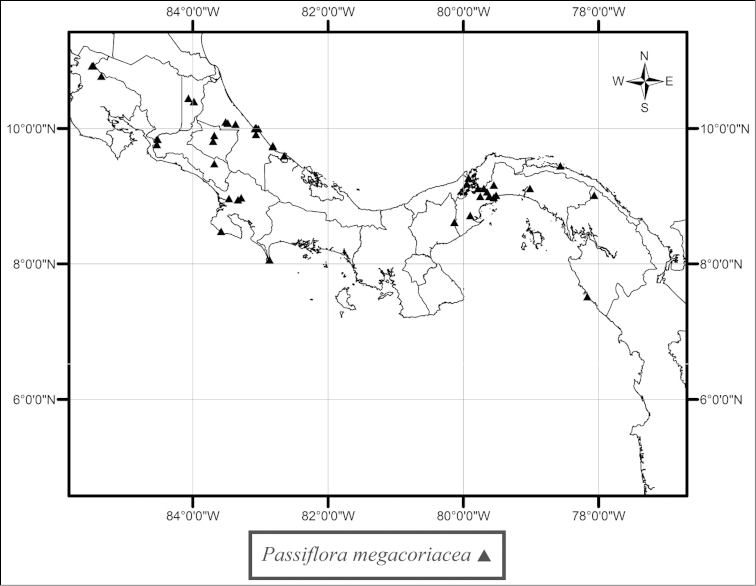
Distribution of *Passiflora megacoriacea*.

##### Phenology.

Flowering and fruiting throughout the year.

##### Distribution.

Colombia, Costa Rica, and Panama. Growing in shrubs or trees in secondary successional areas, along the edges of tropical moist to premontane wet forests, and near the seashore, 0–1100 m altitude.

##### Discussion.

*Passiflora megacoriacea* is relatively common in Costa Rica and Panama. John MacDougal brought my attention to the variation of vegetative and floral characters of some of the Costa Rican and Panamanian specimens then identified as *Passiflora coriacea*.

*Passiflora megacoriacea*, as noted above in the discussion of *Passiflora coriacea*, is very similar to *Passiflora coriacea* and *Passiflora sexocellata*, and although not sympatric, without reproductive material it can be difficult to separate them. *Passiflora megacoriacea* may be recognized by commonly having petiolar nectaries found on the distal half of the petiole, (0.30-) 0.50–0.77 of the distance from the base toward the apex of the petiole, and although that overlaps the 0.21–0.54(-0.64) range of *Passiflora coriacea* and *Passiflora sexocellata*, the character is easily seen in herbarium specimens. *Passiflora megacoriacea* can also possess deeply trilobed leaves (commonly 0.11–0.61 the distance to the base), especially in populations along the Pacific coast of Costa Rica and in the Panamá Canal Zone, whereas *Passiflora coriacea* and *Passiflora sexocellata* do not possess deeply trilobed leaves (commonly less than 0.11 the distance to the base). The reproductive structures of these three species provide a number of distinguishing characters. *Passiflora megacoriacea* possesses floral stipes that are commonly less than half the length of the pedicels, whereas *Passiflora coriacea* possess stipes that are usually two to three times the length of the pedicels and *Passiflora sexocellata* has floral stipes that are commonly just shorter than or rarely up to two times the length of the pedicels. The overall size of the flower of *Passiflora megacoriacea* exceeds that of both *Passiflora coriacea* or *Passiflora sexocellata*, with *Passiflora megacoriacea* commonly having a wider hypanthium, longer sepals, larger and fewer outer coronal filaments, a longer androgynophore, longer staminal filaments, longer anthers, and a longer operculum. The most informative of these is the length of the androgynophore, with *Passiflora megacoriacea* having an androgynophore that is 6.9–8.8 mm long and the androgynophores of both *Passiflora coriacea* and *Passiflora sexocellata* not exceeding a length of 5.9 mm. In addition, the nectary floor is raised in *Passiflora megacoriacea*, never raised in *Passiflora sexocellata*, and only rarely raised in *Passiflora coriacea*. The outer coronal filaments of both *Passiflora megacoriacea* and *Passiflora sexocellata* are erect, while those of *Passiflora coriacea* spread to ca. 140–160°. The flowers of *Passiflora megacoriacea* are commonly referred to as white, greenish white, or cream on herbarium labels and this is due to it having no (or relatively little) reddish purple coloration in the mature flowers; the flowers of *Passiflora coriacea* and *Passiflora sexocellata* both commonly have a significant amount of reddish purple coloration.

According to Benson et al. (1975), *Passiflora megacoriacea* (based on geography) has a different passionflower butterfly herbivore than *Passiflora coriacea* and *Passiflora sexocellata*. *Heliconius cydno* has been reported to be the primary herbivore of *Passiflora megacoriacea* in Panamá and southeastern Costa Rica, though *Heliconius erato* is also known to utilize this species. *Heliconius erato* is the primary herbivore of *Passiflora coriacea* and *Passiflora sexocellata*. *Dryas julia* is also an herbivore of *Passiflora sexocellata* (Benson et al. 1975).

In an unpublished manuscript, MacDougal determined the total sugar concentration measured as sucrose equivalents in percent weight per total weight to be 29–44% in *Passiflora megacoriacea* (*MacDougal 409*). He found the flower odor to be sweet, waxy, and strong. These data indicate that the flowers are likely utilized by bees.

##### Specimens examined (paratypes).

**COSTA RICA. Cartago:** Pasture beside Río Pejibaye, 2 km SW of Taus, 750 m, *Lent 2960* (F); Las Vueltas (de Tucurrique), 635 m, *Tonduz 12808* (US). **Guanacaste:** Parque Nacional Guanacaste Estación Biología Volcán Cacao, 1100 m, *Alvarado 28* (CR, MO); Parque Nacional Rincón de la Vieja Liberia, Cordillera de Guanacaste, Estación Las Pailas, 800 m, *Espinoza 708* (CR, MO). **Herédia:** Los pastizales de la Finca de Napoleon Murillo, *Chacon 778* (DUKE); Finca La Selva, the OTS Field Station on the Río Puerto Viejo, just E of its junction with the Río Sarapiquí, 100 m, *Grayum 2782* (DUKE); N base of hills to the S of the Río Sarapiquí, opposite Chilamate, 60–100 m, *Grayum et al. 5316* (MO). **Limón:** Between Siquerres and the Río Pacuare, and remnant forest on steep hills S of the railroad bridge over Río Pacuare, 50–100 m, *Burger & Liesner 6868* (F, MO); Canton de Siquirres, llanura de Santa Clara, puente sobre Río Barbilla, 50 m, *Chavarria & Solis 955* (MO); Talamanca, Sixaola, en la fila entre Gandoca & Manzanillo frente a Punta Mona, 50–100 m, *Herrera & Bloemen 7632* (F, MO, US); along beach between Port Limón and Moin, *Pittier 3630* (BM, US); Parque Puerto, Vargas, *Poveda & de Ramury 3270* (CR, F). **Puntarenas:** Carara National Park, near Río Carara, near guard post, 120 m, *Gentry et al. 79273* (CR, MO); Canton de Buenos Aires, cañon del Río Grande de Terraba, cerca del Proyecto Boruca-ICE, 100 m, *Hammel et al. 17870* (CR, MO); Parque Nacional Corcovado, Sirena, Río Claro Trail-Río Claro, 0–150 m, *Kernan 131* (MO); Canton de Osa, R.B. Isla del Caño, Península de Osa, 1 m, *Lepiz 462* (MO); Burica Península, unnamed quebrada opposite Quebrada Macho of Panamá, 11 mi. S of Puerto Armuelles, 20–200 m, *Liesner 184* (MO); Reserva Biología Carara, 200 m, *Morales 1267* (MO); Bords du Río Platanar, Hacum, pres Buenos Aires, 250 m, *H. Pittier 6584* (MO); Canton de Buenos Aires Reserva Indígena Boruca, 200 m, *Rojas & Zuniga 158* (CR, MO).

**PANAMÁ. Canal Zone** (currently separated into the provinces of Colón and Panama): Shoreline of E side of Peña Blanca Point across from front no. 8 light, Barro Colorado Island, *Croat 6732* (MO); Río Majé, along river from waterfalls near Bayano Lake to Finca of Chocó Indian Eduardo Maycha, ca. 2 mi. upstream, 30–60 m, *Croat 34557* (MO); vicinity of Panamá Railroad crossing at Guillard Hwy., across road from former Summit Hills golf course, *Croat & Zhu 76290* (MO); between Chilibre & Madden Dam on Transisthmian Hwy., *Dwyer & Correa 9397* (MO); Forest preserve, near Green Park, *Folsom 228* (MO); Barro Colorado Island, SE of Gross Point, *Foster 2285* (DUKE); Barro Colorado Island, tower clearing, *Foster 769* (DUKE); Gaillard hwy., mi. 12–13, *Garwood 1861A* (F); Gatún Locks, *Gilbert 409* (FLAS); on brush along railroad, Summit Gardens, *Hammel 1787* (MO); ca. de Represa Madden, Campo de Exploradores, *Kant 21* (DUKE); junction of Chiva-Chiva and Gaillard Hwy., 50 m, *Knapp & Schmalzel 4870* (MO); Pipeline Road, ca. 5 km from beginning, just NE of crossing of Río Siristes, 128 m, 9°10N, 79°45 W, *MacDougal et al. 6315* (MO); Around Alahajuela, Chagres Valley, 30–100 m, *Pittier 3456* (US); Boy Scout Road, Madden Dam area, *Porter et al. 4014* (MO, UC); Las Cascadas Plantation, near Summit, *Standley 29594* (US); Darien Station, *Standley 31617* (US); near Survival School, Curundú, *Tyson 1054* (MO); Boy Scout Camp on Madden Lake, *Tyson 5454* (MO); Fort Clayton, no. 519, the old hospital building, *Tyson & Blum 3901* (MO, US); Shore N of end of Chapman Trail, *Woodworth & Vestal 501* (A, F, MO); Río Vigue Beach, *Zetek 5564* (MO). **Chiriquí:** near San Juan, *Seemann, s. n.*, 1844 (K). **Coclé:** N rim of El Valle de Antón, 600–1000 m, *Allen 1667* (MO); Forest behind Club Campestre, 700 m, *Duke 13270* (MO); Behind Hotel Turístico, El Valle, 2200 ft., *Hammel 1778* (MO); NE of El Valle de Antón, 2000 ft., *Lewis et al. 1703* (MO); 2.4 km (air) N of the church at El Valle, 725 m, 08°37 N, 80°08 W, *MacDougal & Lezcano 6274* (MO); Above and N of El Copé, road to the old saw mill that used to be called “Whiskey” near the continental divide, now a national park reserve, 484 m, 08°39 N, 80°35 W, *MacDougal et al. 6299* (MO). **Darién:** 3 km S of Jaqué, 0–100 ft., *D’Arcy & Sytsma 14553* (MO); Hill ca. 1 mi. NE of Nura, 200 m, *Duke 10084* (*3*) (ECON, MO). **Panamá:** 1 km E of Chorrera City limits, *Folsom 3466* (MO); SE slope of Cerro Campana, *Lewis et al. 3130* (MO). **San Blas:** on mainland in front of Ustupo, *D’Arcy 9527* (MO).

**COLOMBIA. Bolívar:** Torrecilla, near Turbaco, 150–300 m, *Killip & Smith 14415* (GH, US).

#### 17. Passiflora sexocellata

Taxon classificationPlantaeMalpighialesPassifloraceae

Schltdl., Linnaea 27: 521. 1854.

Figs 52 , 53

##### Type.

Mexico. Veracruz: along Hwy. 180 between Tampico and Pozarica, 12 mi N of Ozuluama, 38 km N of Naranjos, 110 m, 5 June 1987, *T. B. Croat 66095* (neotype, designated here: MO! [MO-312537]).

##### Description.

Slender, climbing, perennial vine 2–6 m long or more, sparsely pubescent with unicellular curved trichomes on petiole, leaf, stem, sepal, and stipule, 0.20–0.64 mm long, 0.02–0.03 mm wide, also minutely antrorsely appressed-puberulent throughout with unicellular, curved trichomes, 0.03–0.12 mm long, 0.02–0.03 mm wide. Flowering stems 1.0–2.4 mm in diameter, terete to somewhat compressed, with the base woody and cork-covered. Stipules 2.5–6.0 mm long, 0.4–1.3 mm wide, narrowly ovate-triangular, acute; petioles 1.2–5.7 cm long, with 2 (rarely 3), opposite to subopposite, sessile, discoid nectaries with flat rims, 1.0–2.1 mm wide (on the widest axis), 0.3–1.3 mm high, borne in the proximal half of the petiole (0.34–0.54 of the distance from the base toward the apex of the petiole). Laminas 2.6–8.5 cm long, 6.5–23.5 cm wide, coriaceous, peltate (the distance from leaf base to point of petiole insertion 3.0–18.9 mm), transversely elliptic (widely divaricately bilobed) or sometimes 3-lobed, lateral lobes 3.7–12.9 cm long, 1.9–7.5 cm wide, elliptic, acute to attenuate, central lobe elliptic to obovate or present merely as a widely acute to obtuse tip (rarely retuse), central vein 1.8–7.0 cm long (measured from point of petiole insertion to the leaf apex), angle between the lateral lobes 132–188°, ratio of lateral lobe to central vein length 1.33–2.77, margins entire, hyaline, primary veins 3, diverging and branching above base, laminar nectaries present, 4–13, submarginal, associated with the minor veins of the abaxial surface, with 2–4 nectaries proximal to the lateral leaf veins, 0.5–1.4 mm in diameter, circular to widely elliptic, sessile; juvenile leaves bilobed and variegated, the variegation seen in some clones at maturity; tendril 0.3–1.0 mm wide, present at flowering node, absent in inflorescence. Flowers borne in leaf axils or inflorescences; inflorescences 2.0–18.5(-25.1) cm long, associated reduced laminas 2.0–4.3 mm long, 1.5–3.1 mm wide. Pedicels 1.9–15.8 mm long, 0.4–0.9 mm wide, 2 per node; bract(s) absent; spur(s) absent. Flowers 18.4–33.4 mm in diameter with stipe 3.1–8.6(-9.4) mm long, 0.5–1.3 mm wide; hypanthium 5.4–8.2 mm in diameter; sepals 6.5–13.3 mm long, 2.9–6.3 mm wide, ovate-triangular, acute to rounded, abaxially and adaxially greenish yellow; coronal filaments in 2 series, the outer 40–51, 5.5–8.4 mm long, 0.3–0.7(-0.8)mm wide, linear, more or less erect, very dark reddish purple (5RP 3/2) on proximal third, greenish yellow (5GY 8/4) on middle third, yellow on distal third (5Y 8/10), ratio of outer coronal row to sepal length 0.59–0.94, the inner 27–40, 2.3–3.8 mm long, 0.2–0.5(-0.6) mm wide, linear, capitate, erect, greenish yellow with a flush of very dark reddish purple at base, ratio of inner coronal row to outer coronal row length 0.35–0.52; operculum 1.2–2.0 mm long, plicate, greenish yellow with a flush of very dark reddish purple at base, the margin white with narrow minutely fimbrillate teeth; nectary 0.1–0.5(-0.6) mm high, 0.5–1.2(-2.9) mm wide; limen not recurved but inclined toward the operculum, 0.1–0.5(-0.7) mm high, 0.1–0.4(-0.5) mm wide, very dark red (5R 2.5/2), limen floor 2.5–5.1 mm in diameter, very dark red; androgynophore (2.1-)3.5–6.0 mm long, 0.4–1.3(-1.5) mm wide, the distal half dark red then greenish yellow with dark red spots and streaks; free portions of the staminal filaments 1.5–2.8 mm long, 0.5–0.9 mm wide, linear, greenish yellow; anthers 1.9–2.9 mm long, 0.6–1.5(-1.9) mm wide; styles 1.8–4.9(-5.4) mm long including stigmas, 0.2–0.5 mm wide, greenish yellow; stigmas 0.8–1.7 mm in diameter; ovary 1.6–2.7 mm long, 1.1–2.1 mm wide, widely ellipsoid to globose, greenish yellow. Berry (12.4-)18–23.1 mm long, (13.4-)18.4–33.0 mm in diameter, globose, very dark purple. Seeds ca. 40–50, 1.8–4.8 mm long, 2.1–2.9 mm wide, 1.5–2.1 mm thick, obovate in outline, acute at both ends, reticulate-foveate with each face marked with ca. 12–17(-19) foveae. Germination epigeal.

**Figure 52.**
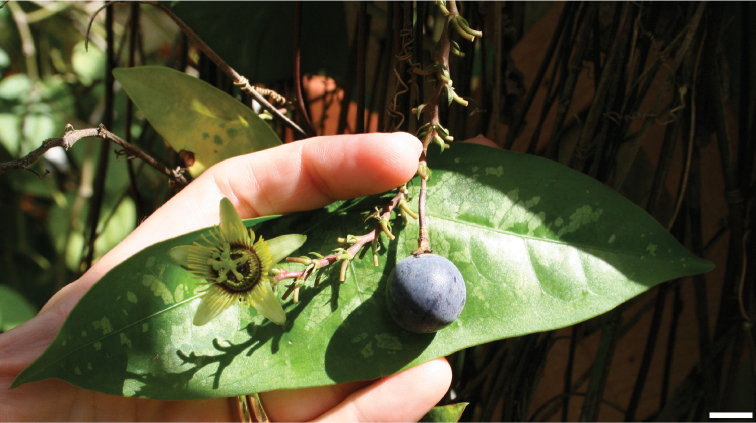
Leaf, flower, inflorescence, and fruit of *Passiflora sexocellata* from plant growing in greenhouse at Butterfly World, Coconut Creek, Florida. Scale bar = 10 mm.

**Figure 53.**
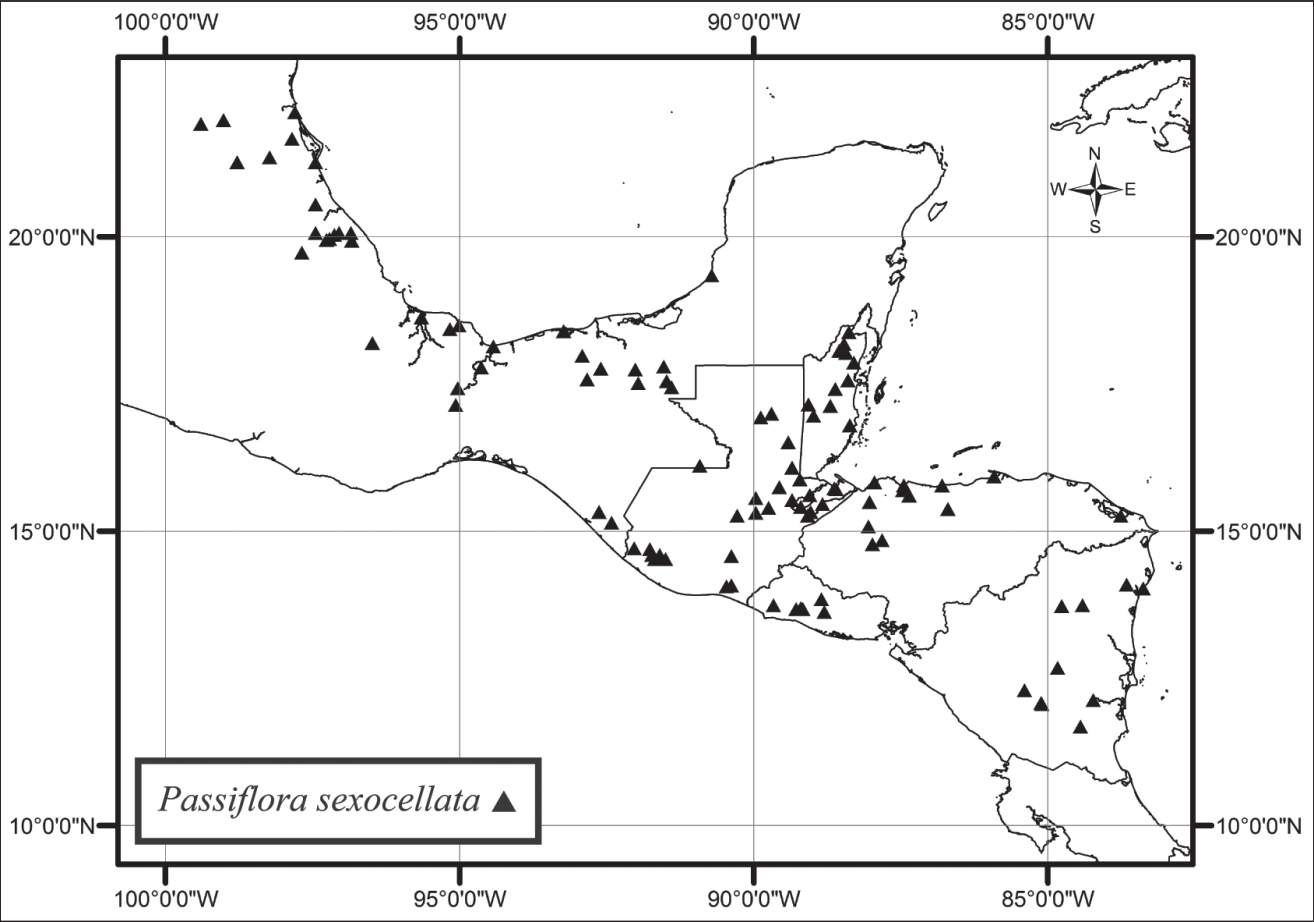
Distribution of *Passiflora sexocellata*.

##### Phenology.

Flowering and fruiting throughout the year.

##### Distribution.

Mexico and Central America (except Costa Rica and Panama). Growing in shrubs, trees or trailing on the ground in secondary successional areas, along the edges of semideciduous to deciduous, dry to wet tropical forests, both inland and near the seashore, 0–1171 m.

##### Ethnobotany.

The vine is sold in Guatemalan herb markets and is sold dried where the plant does not grow naturally (Morton 1981). A decoction of the leaves is commonly taken as a diuretic, especially in the treatment of kidney infections (Morton 1981). In El Salvador and Honduras the leaves are combined with lard and used as a poultice on wounds and swellings (Morton 1981).

##### Discussion.

*Passiflora sexocellata* is very similar to *Passiflora coriacea* and *Passiflora megacoriacea*, and some of their similarities and differences are discussed under their respective descriptions. According to Jan Meerman (pers. comm.), *Passiflora sexocellata* and *Passiflora xiikzodz* grow side by side in Belize, with *Passiflora sexocellata* growing in the sun and *Passiflora xiikzodz* growing in the shade. Where these two species are found in the Yucatán Peninsula of Mexico, I found that *Passiflora sexocellata* occurs in wetter forests along rivers and lakes to the west and *Passiflora xiikzodz* and the related *Passiflora itzensis* are found in drier forests to the east. However, MacDougal concluded that these plants grow together at some sites in the Yucatán (MacDougal 1992). These two species are easily separated because *Passiflora xiikzodz* and *Passiflora itzensis* possess petiolar nectaries at or near the apex of the petiole whereas *Passiflora sexocellata* has petiolar nectaries on the proximal half of the petiole. In addition, numerous floral characters can be used to distinguish between them. The most obvious difference is the number of coronal rows, with *Passiflora xiikzodz* and *Passiflora itzensis* possessing seven series and *Passiflora sexocellata* possessing only two.

Klucking (1992) classified the leaf venation of *Passiflora sexocellata*, identified as *Passiflora coriacea*, as actinodromous and pinnate secondary venation with irregular to regular intercostal venation consisting of lineate to transverse veins. The peltate, trilobed leaf illustrated is the typical form for *Passiflora sexocellata*. There are three primary veins and two acrodromal veins which extend two-thirds the length of the lateral lobes, the lateral lobes are acute, the angle between the lateral veins is 150°, and there are six laminar nectaries apparent on the abaxial surface (Klucking 1992).

In Belize, Meerman (2001) found that *Heliconius erato* is an herbivore of *Passiflora sexocellata* (which he identified as *Passiflora coriacea*). Benson et al. (1975) found that *Dryas julia* and *Heliconius erato* were herbivores of *Passiflora sexocellata* (again, identified as *Passiflora coriacea*).

In 1990, Joanna Turner collected Plaster bees, *Colletes* sp., that regularly visited flowers of *Passiflora sexocellata* in Belize. The bees are approximately 10 mm long, 3–4 mm high, including some off-the-ground leg clearance, and have a thorax that is 2.0–2.5 mm high, and were identified by Rick Clinebell at MO (pers. comm.).

*Passiflora sexocellata* was originally described by Schlechtendal in 1854. He cited “*Passiflora marmorea* hort.”, as a synonym, but this horticultural name was not validly published. It is interesting that the specific epithet “marmorea” means marbled, as the leaves of this species are often variegated. Holm-Nielson et al. (1988), in the synonomy of *Passiflora coriacea*, stated that *Passiflora sexocellata* is an illegitimate name that was based upon material of *Passiflora coriacea* Juss. and *Passiflora difformis* Kunth. However, I do not see any reason why Schlechtendal’s species has to be considered illegitimate. He carefully describes the plant from cultivated material that he had at hand in the Botanical Gardens in Halle, Germany and spends a paragraph differentiating his species from both *Passiflora coriacea* and *Passiflora difformis*. I was unable to locate the type of *Passiflora sexocellata*, and U. Braun (curator of the herbarium at the Herbarium at the Institut fur Geobotanik und Botanischer Garten, Halle) was unable to find any material under the name *Passiflora sexocellata*. Braun was also unable to locate appropriate material under *Passiflora coriacea* or *Passiflora difformis*. Other species of *Passiflora* from Mexico and Central America were in cultivation in Europe by 1830 (Loudon 1830), and it is plausible that Schlechtendal had such material at hand when he described *Passiflora sexocellata*. Schlechtendal’s *Passiflora sexocellata* seems to fit the description of the Mesoamerican entity that I am recognizing as a species distinct from *Passiflora coriacea* and other similar taxa from supersection *Cieca*. However, some of the vegetative characters that he uses to distinguish *Passiflora sexocellata* are actually quite variable, but he only had one live specimen available to him when he described the species. He describes the flower as having five green sepals, outer coronal filaments that are “lilac” at the base but “greenish yellow” otherwise, inner coronal filaments that are dilated at the apex and “lilac” in color at the tips and lighter toward the base, an operculum that is dull “lilac” at the base and becoming “greenish yellow” toward the apex, and a “greenish yellow” androgynophore. The use of the term “lilac” is somewhat misleading, but the description of how the colors vary on the various parts of the flower is diagnostic. For example, *Passiflora coriacea* possesses outer coronal filaments that are reddish purple at the base but obviously white toward the tips with a band of reddish purple and not “greenish yellow.” In addition, the operculum of *Passiflora coriacea* is wholly reddish purple. However, *Passiflora sexocellata* possesses outer coronal filaments that are reddish purple at their bases, greenish yellow at their middles and yellow at their apices and an operculum that is dark reddish purple at the base and greenish yellow otherwise (often with a white margin). Based upon Schlechtendal’s detailed description, I apply the name *Passiflora sexocellata* to this species and have designated a neotype that perfectly illustrates the diagnostic characters of the taxon, with the colors of the corona and limen floor still very vibrant.

##### Selected specimens examined.

**MEXICO. Campeche:** road between Ulumal and Canosayab, *Porter-Utley & Mondragón 311* (CICY); road (MEX15) between El Estado de Mexico and Monclova, close to El Estado de Mexico, *Porter-Utley & Mondragón 314* (FLAS, CICY); Champoton, *Steere 1888* (US). **Chiapas:** Mpio. Ocosingo, el ejido Chajul a la orilla del Río Lacantun, 150 m, *Martínez et al. 26047* (XAL). **Oaxaca:** 5 mi. E of Temascal (10 mi. W of Veracruz border), 45 ft., *Janzen s.n.*, 13 November 1963 (UC). PUEBLA: Mpio. Tenampulco, Tenampulco, *Chavez & Kerbel 327* (CICY). **San Luis Potosi:** Mpio. Ciudad Valles, ca. 1 km upstream from Rancho Pago Pago on Río Mesillas, 120 m, *Fryxell & Anderson 3449* (CHAPA, MO). **Tabasco:** cerca de la parcela de Don Justo Hernández, Ejido Fernández Manero, km 12.1 del camino hacia cacaos de la desviación KM 32 de la carretera Villahermosa hacia Escarcega, *Cowan 2815* (CAS, NY); San Isidro, near Balancan, *Matuda 6045* (LL); road (MEX 180) between Minatitlan and Villahermosa, *Porter-Utley & Mondragón 384* (CICY, FLAS). **Veracruz:** Mpio. Coatzacoalcos, Coatzacoalcos, entro las dos lenguas de la laguna del Ostion, *Castillo-Campos & Acosta 16155* (XAL); Mpio. Tlacotalpan, along the hwy. following the Río Papaloapan towards the coast, 2 km NE of Tlacotalpan, 2 m, *Nee & Taylor 26567* (F, MO); Playa Escondida, *Porter-Utley & Mondragón 326* (CICY, FLAS); La Palmilla, Mpio. de Tlapacoyan, *Ventura 1270* (CHAPA, MEXU); El Encanto, Mpio. de Tlapacoyan, *Ventura 19595* (CAS, XAL).

**BELIZE. Belize:** along Belize River near Burrel Boom, near sea level, *Gentry 8046* (MO); Caves Branch Base Camp, *Whitefoord 1327* (BM). COROZAL: Alfonsoville, *Gentle 821* (MO, NY, US). **Orange Walk:** Mi. 54, N Hwy., *Dwyer & Liesner 12214* (MO). **Stann Creek:** Swasey Branch, Monkey River, *Gentle 3931* (GH, NY).

**EL SALVADOR. Cabañas:** Ilobasco, *Villacorta & Rivas 2117* (MO). **La Libertad:** Santa Tecla, *Garcia 151* (UC). **San Salvador:** Cerro de San Jacinto, near San Salvador, 800–1171 m, *Standley 20602* (GH, NY, US). **San Vicente:** vicinity of San Vicente, 400–500 m, *Standley & Padilla 3444* (F). **Sonsonate:** vicinity of Izalco, 400–600 m, *Pittier 1949* (US).

**GUATEMALA. Alta Verapaz:** 1 km N de finca Mercedes, Telemán, Panzós, 32 m, *Martínez et al. 22859* (MEXU); Pantín, below Tamahú, 600 m, *Standley 70882* (F). **Chiquimula:** Chocón Plantation, *Watson s.n.*, 20 March 1885 (GH). **Esquintla:** San Luis, N of Escuintla, 450 m, *Standley 60135* (F). **Izabal:** 27 km from junction of Atlantic Rt. with road to Tikal, *McDade 210* (DUKE). **Petén:** SE part of Cerro Cauhui, *Walker 1172* (MO). **Retalhuleu:** above Asintal, on road toward Colomba, 750–800 m, *Standley 87879* (F). **San Marcos:** vicinity of Pajapita, 120 m, *Molina & Molina 27104* (F). **Santa Rosa:** Region of La Morenita, NE of Chiquimulilla, 400 m, *Standley 78869* (F). **Suchitepéquez:** Las Ánimas, 650 m, *Shannon 274* (US). **Unknown State:** *Tejada 248* (US).

**HONDURAS. Atlántida:** Valle Río Lean near El Mazapán N of Mezapa, 20 m, *MacDougal et al. 3298* (BM, CHAPA, MO, TEFH). **Comayagua:** 1 km SW Palmitia, 840 m, *Lentz 996* (TEFH); Pitosolo Yojoa, 500 m, *Valerio & Rodriguez 2895* (F). **Cortes:** Mountains E of Lake Yojoa, 600–800 m, *Morton 7760* (US). **Gracias A Dios:** Leymus, orilla del Río Segovia o Wanki, 100 km SO de Puerto Lempira, 30 m, *Nelson & Cruz 8707* (TEFH). **Santa Barbara:** San Pedro Sula, 1200 m, *Thieme 5242* (US).

**NICARAGUA. Chontales:** Cerro Oluma, 750 m, *Gentry et al. 43989* (MO). **Matagalpa:** Carretera al Tuma 6 km NW de Cuatro Esquinas, 700–800 m, *Guzman et al. 812* (MO). **Region Autonomista Atlántico Norte:** matorrales de la Playa S de Puerto Cabezas, 0 m, *Molina 14759* (F).

#### 18. Passiflora itzensis

Taxon classificationPlantaeMalpighialesPassifloraceae

(J. M. MacDougal) K. Porter-Utley. comb. nov.

urn:lsid:ipni.org:names:77143306-1

Figs 54 , 55

Passiflora xiikzodz J.M. MacDougal subsp. itzensis J.M. MacDougal, Novon 2: 363. figs 2–4. 1992. Type: Mexico. Yucatán: Chichén Itzá, *C. L. Lundell & A. A. Lundell 7470* (holotype: LL! [LL00031117]; isotypes: LL, photograph seen [LL00372050], MEXU, MICH, photograph seen [MICH1125812], US! [US00479062]).

##### Type.

Based on Passiflora xiikzodz J.M. MacDougal subsp. itzensis J.M. MacDougal

##### Description.

Slender, low-climbing or trailing, perennial vine 1–3 m or more, minutely antrorsely appressed-puberulent throughout with unicellular, curved trichomes, 0.06–0.11 mm long, 0.02 mm wide. Flowering stems 1.4–2.3 mm in diameter, terete or somewhat compressed, greenish yellow (5GY 8/4) to very dark reddish purple (5RP 2.5/2). Stipules 2.5–5.6 mm long, 0.4–0.6 mm wide, narrowly ovate, acute to slightly attenuate, longitudinally striate-nerved; petioles 0.9–1.8(-3.0) cm long, inserted 2.4–6.1(-7.0) mm from the basal margins of the peltate blades, with 2, round or elliptic, opposite, sessile, discoid nectaries with flat rims, 1.3–1.9 mm wide (on the widest axis), 0.5–0.9 mm high, borne in the distal third of the petiole (0.62–0.83 of the distance from the base toward the apex of the petiole). Laminas 2.3–4.6 cm long, 5.0–12.4(-13.1) cm wide, coriaceous, often variegated along primary veins and major secondary veins, ratio of leaf width to central vein length measured from point of petiole insertion 1.9–5.1, depressed obovate to transversely elliptic (widely divaricately bilobed), lateral lobes (3.5-)4.3–7.4 cm long, 1.7–4.1 cm wide, elliptic, acute to slightly attenuate, central lobe commonly obsolete or present as an obtuse tip, central vein 1.8–3.1(-4.1) cm long (measured from point of petiole insertion), angle between the lateral lobes (85-)103–140°, ratio of lateral lobe to central vein length 1.4–2.8, margins entire, hyaline, primary veins 3, diverging and branching above base, laminar nectaries present, 6–19, submarginal, associated with the minor veins of the abaxial surface, 0.6–1.8 mm in diameter, widely elliptic to circular, sessile; tendril 0.4–0.9 mm wide, present at flowering node, absent in inflorescence. Flowers borne in leaf axils or inflorescences; inflorescences 5.3–9.6 cm long, associated reduced laminas 1.9–2.5 mm long, 1.3–2.7 mm wide. Pedicels 1.3–3.4(-5.8) mm long, 0.6–1.1 mm wide, (1-)2 per node; bract(s) absent; spur(s) absent. Flowers 20.3–25.5 mm in diameter with stipe 9.1–14.3 mm long, 0.6–1.0 mm wide; hypanthium 4.0–6.2 mm in diameter; sepals 7.5–9.8 mm long, 2.6–4.3 mm wide, ovate-triangular, acute, abaxially and adaxially greenish yellow or sometimes greenish yellow with very dark reddish purple streaks abaxially; coronal filaments in 7 series, the outer 22–31, 6.3–8.1 mm long, 0.2–0.3 mm wide, linear, spreading flat, the tips often slightly incurved, very dark reddish purple (5RP 2.5/2–3/2) with yellow (5Y 8/4–8/6) at tips, ratio of outer coronal row to sepal length 0.67–0.97, the second 20–30, 2.5–5.0 mm long, 0.1–0.2 mm wide, linear, spreading flat, very dark reddish purple with yellow tips, ratio of second coronal row to outer coronal row length 0.33–0.64(-0.75), the third ca. 50, 0.7–2.1 mm long, 0.05–0.13 mm wide, linear, spreading flat, very dark reddish purple with yellow tips, ratio of third coronal row to second coronal row length 0.22–0.59, the fourth through seventh ca. 100 per series, 0.7–1.1 mm long, 0.05–0.11 mm wide, linear, capitate, erect, very dark reddish purple, ratio of coronal rows 4–7 to third coronal row length 0.51–0.62(-0.90); operculum 0.3–0.4 mm long, denticulate, very dark reddish purple, nectary absent; limen absent, limen floor 2.8–4.1(-5.7) mm in diameter, very dark reddish purple; androgynophore appearing absent, or 0.3–1.7 mm long, 0.9–1.8 mm wide; free portions of the staminal filaments 1.9–3.4 mm long, 0.5–0.8 mm wide, linear, very dark reddish purple; anthers 1.3–2.0 mm long, 0.7–1.4 mm wide, introrse at anthesis with their axes maintained more or less parallel to the filament, anthers dehiscing distally; styles 1.8–3.1 mm long including stigmas, 0.3–0.5 mm wide, very dark reddish purple or greenish yellow with very dark reddish purple tinge toward base; stigmas 0.9–1.4 mm in diameter; ovary 1.7–2.4 mm long, 1.2–1.3 mm wide, widely ellipsoid to globose, greenish yellow. Berry 26.0 mm long, 14.0 mm in diameter, ovoid to obovoid, greenish yellow with white spots, becoming soft at the base at maturity. Seeds 30–40, 5.0–5.5 mm long, 2.0–2.2 mm wide, 1.3–1.8 mm thick, elliptic to slightly obovate in outline, acute at both ends, reticulate-foveate with each face marked with 20–22 foveae. Germination type epigeal.

**Figure 54.**
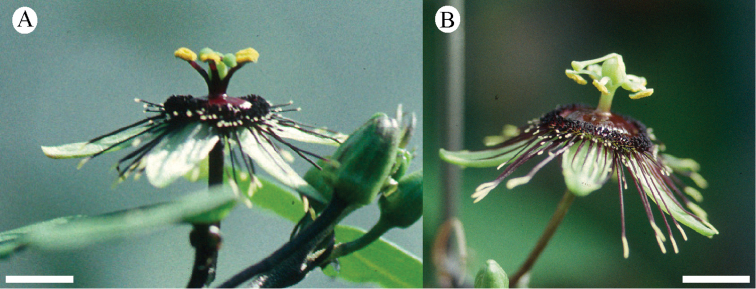
**a** Flower of *Passiflora itzensis* (*MacDougal 4633*) Scale bar = 5.0 mm. Photo by J. M. MacDougal **b** Flower of *Passiflora xiikzodz* (*MacDougal 4677*) Scale bar = 5.0 mm. Photo by J. M. MacDougal.

**Figure 55.**
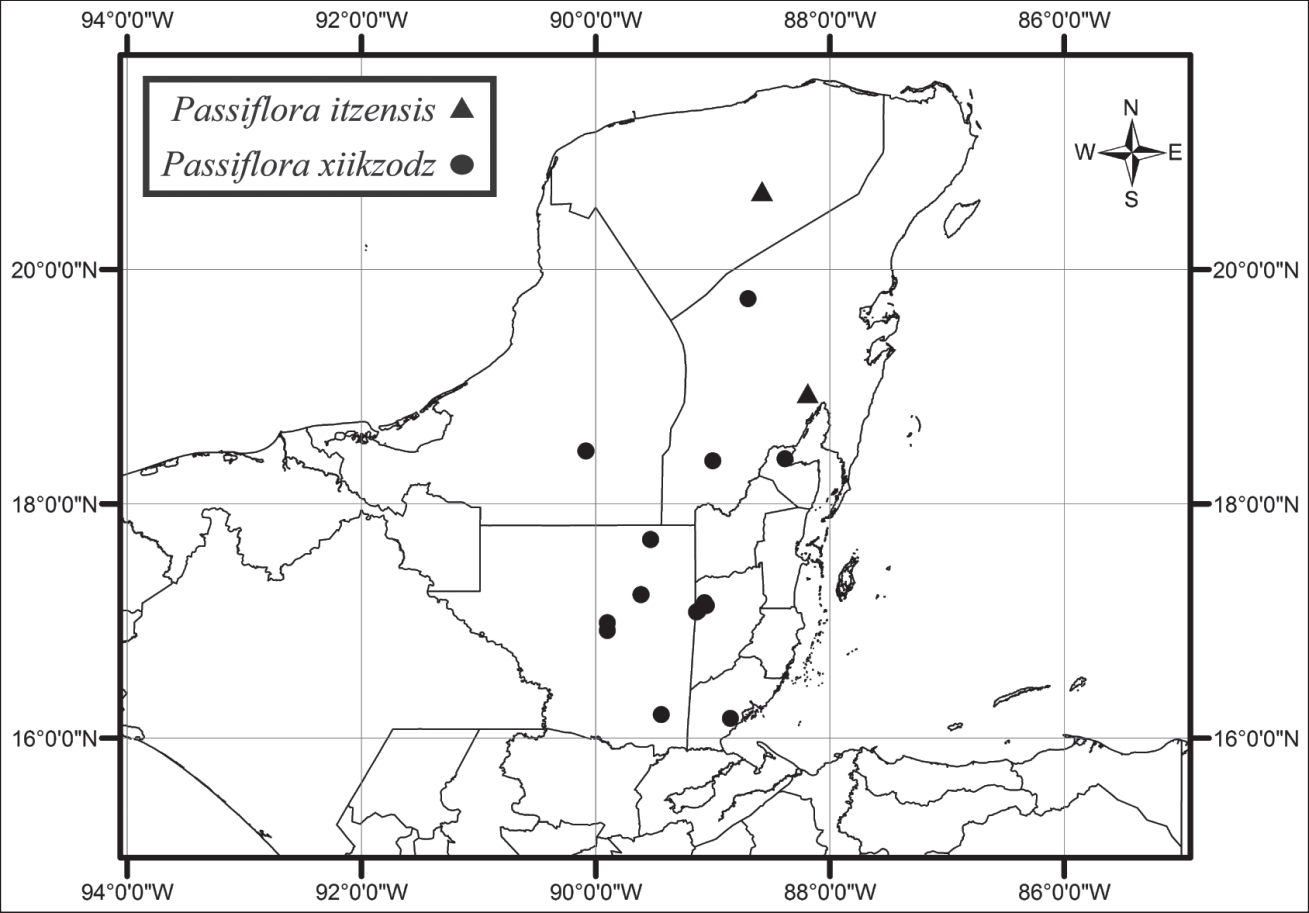
Distribution of *Passiflora itzensis* and *Passiflora xiikzodz*.

##### Phenology.

Flowering and fruiting September to June.

##### Distribution.

Mexico, in the states of Campeche, Quintana Roo, and Yucatán. Tropical semideciduous forests (selva mediana subcaducifolia and selva mediana subperennifolia); growing in shrubs or trailing along the ground on soil of little depth, lying directly on top of limestone; 0–23 m.

##### Discussion.

In 1992, MacDougal described *Passiflora xiikzodz* from herbarium specimens circulated as *Passiflora coriacea* from Belize, Guatemala, and the Yucatán Peninsula. He found the floral corona of this new species to be fundamentally different from *Passiflora coriacea* and the other members of supersection *Cieca*, as it is 5–7-seriate as opposed to 2-seriate. He noted the absence of the floral nectary and the very reduced, denticulate operculum of this species. The seeds are also longer than all of the other species in the supersection. The petiolar nectaries are positioned on the distal third of the petiole in *Passiflora xiikzodz* and the floral stipe is diagnostically long. MacDougal further separated *Passiflora xiikzodz* into two subspecies, Passiflora xiikzodz subsp. xiikzodz and Passiflora xiikzodz subsp. itzensis. Though he found numerous differences in the flowers of the two subspecies and artificial cross-pollinations between them proved unsuccessful, he felt that more information was needed to support the recognition of two separate species. I recognize the two species, *Passiflora xiikzodz* and *Passiflora itzensis*, which is supported by my morphological and molecular analyses of the taxa (see chapters 4 and 6).

*Passiflora itzensis* and *Passiflora xiikzodz* are identical vegetatively, but the flowers are quite different. The flowers of *Passiflora itzensis* lack or have a greatly reduced dark reddish purple androgynophore, are smaller, possess fewer filaments in the outer and second coronal rows, an androecium and gynoecium with reddish purple pigmentation, very short styles, stigmas with their receptive surfaces presented distally, and anthers that do not flip over to an extrorse position after the flower buds open but move only slightly from the original introrse position to present their pollen distally. In the herbarium, it is not necessary to have perfectly preserved flowers to differentiate between *Passiflora itzensis* and *Passiflora xiikzodz*, as the floral stipe of *Passiflora itzensis* is commonly shorter than that of *Passiflora xiikzodz*. Incidentally, in the dried flowers of both *Passiflora itzensis* and *Passiflora xiikzodz*, the coronal filaments appear nearly black.

The occasional appearance of one or two small but well-formed petals in cultivated material of *Passiflora itzensis* has been noted (*MacDougal 4633*) (MacDougal 1992). I also noticed this in the same clone (*MacDougal 4633*) and in another clone given to me by T. Skimina (*Porter-Utley P-69*). Tim Skimina (pers. comm.) successfully crossed *MacDougal 4633* and *Porter-Utley P-69*. The fruits from this cross were greenish yellow with white spots at maturity and possessed 30–40 light brown seeds. After approximately 35–40 days, the mature fruits began to soften at the apex and, at that time, became very attractive to animals in and around his garden. It is thanks to Tim Skimina’s efforts that we now have such detailed information about the fruits of this species.

##### Specimens examined.

**MEXICO. Quintana Roo:** Puerto Morelos, Jardín Botánico Benito Juárez, 3–8 m, *Escalante 127* (CICY); along MEXICO 307 between Chetumal and Cancún, 18°56.71N, 88°11.34W, 20 m, *Porter-Utley & Mondragón 395* (CICY). **Yucatán:** Chichén Itzá, near Pisté, *Lundell & Lundell 7375* (MICH); Mpio. Tinum, A 3 km de Tinum rumbo a San Francisco, 23 m, *Ucan 2303* (CICY).

**CULTIVATED MATERIAL.** cultivated at Missouri Botanical Garden 1989–1992 from a cutting collected in 1989 by Sr. Dzib and E. Leiter at Chichén Itzá, *MacDougal 4633* (MO); cultivated at the University of Florida from a plant collected by Tim Skimina15 September 1990 at Chichen Itza in Yucatan, Mexico, *Porter-Utley P-69* (FLAS).

#### 19. Passiflora xiikzodz

Taxon classificationPlantaeMalpighialesPassifloraceae

J.M. MacDougal, Novon 2: 361, figs 2–4. 1992.

Figs 54 –55

##### Type.

Mexico, Campeche, Tuxpeña, [18°26'N, 90°06'W], 19 Jan. 1932, *C. L. Lundell 1210* (holotype: MICH, [MICH1125811, photograph seen]; isotypes: ARIZ [79504], CAS [CAS0000380, photograph seen], DS [DS 235425, photograph seen], F! [F0044451F], GH! [GH00065789], MO! [MO-312536], NY [NY00232351, photograph seen], US! [US00479061], WIS [v0248704WIS, photograph seen]).

##### Description.

Slender, low-climbing or trailing, perennial vine 1–3 m or more, minutely antrorsely appressed-puberulent throughout with unicellular, curved trichomes, 0.1–0.2 mm long, 0.02–0.03 mm wide. Flowering stems 1.3–2.3 mm in diameter, terete or somewhat compressed. Stipules 1.0–4.9 mm long, 0.3–0.7 mm wide, narrowly ovate, acute to slightly attenuate, longitudinally striate-nerved; petioles (0.1-)0.5–3.0 cm long, inserted 1.4–7.3(-8.9) mm from the basal margins of the peltate blades, with 2, round or elliptic, opposite to subopposite, sessile, discoid nectaries with flat rims, 1.1–1.9 mm wide (on the widest axis), 0.3–1.0 mm high, borne in the distal third of the petiole (0.63–0.87 of the distance from the base toward the apex of the petiole). Laminas 1.1–5.8 cm long, 3.4–13.7 cm wide, coriaceous, commonly variegated along primary veins and major secondary veins, conspicuously peltate, ratio of leaf width to central vein length measured from point of petiole insertion 2.0–5.9, depressed obovate to transversely elliptic (widely divaricately bilobed), lateral lobes 2.6–7.3(-8.1) cm long, 0.8–4.9 cm wide, elliptic, obtuse or acute to slightly attenuate, central lobe commonly obsolete or present as an obtuse to retuse tip, central vein 0.9–4.4(-5.4) cm long (measured from point of petiole insertion), angle between the lateral lobes 53–162°, ratio of lateral lobe to central vein length 1.3–3.0(-4.8), margins entire, hyaline, primary veins 3, diverging and branching above base, laminar nectaries present, 6–17, submarginal, associated with the minor veins of the abaxial surface, 0.8–1.3 mm in diameter, widely elliptic to circular, sessile; tendril 0.4–0.8 mm wide, present at flowering node, absent in inflorescence. Flowers borne in leaf axils or inflorescences; inflorescences 4.5–22.3 cm long, associated reduced laminas 2.1–5.3 mm long, 1.3–2.5 mm wide. Pedicels 1.3–3.1(-9.9) mm long, 0.4–1.1 mm wide, (1-)2 per node; bract(s) absent; spur(s) absent. Flowers 18.8–31.9 mm in diameter with stipe 12.3–19.0(-23.3) mm long, 0.4–0.8 mm wide; hypanthium 4.8–8.1 mm in diameter; sepals 6.5–12.3 mm long, 3.1–6.3 mm wide, ovate-triangular, acute, abaxially and adaxially greenish yellow; coronal filaments in 7 series, the outer 40–50, 6.3–10.4 mm long, 0.1–0.3 mm wide, linear, spreading flat, reflexed above middle and the tips often slightly incurved, very dark reddish purple with yellow at tips, ratio of outer coronal row to sepal length 0.73–1.38, the second 35–50, 2.3–4.8(-5.1) mm long, 0.1–0.2 mm wide, linear, spreading flat, very dark reddish purple with yellow tips, ratio of second coronal row to outer coronal row length 0.23–0.60, the third 40–50, 0.8–3.0 mm long, 0.06–0.13 mm wide, linear, spreading flat, very dark reddish purple with yellow tips, ratio of third coronal row to second coronal row length 0.20–0.65, the fourth through seventh ca. 100 per series, 0.6–1.3 mm long, 0.1–0.2 mm wide, linear, capitate, erect, very dark reddish purple, ratio of coronal rows 4–7 to third coronal row length 0.30–0.72(-0.91); operculum 0.3–0.7 mm long, denticulate, very dark reddish purple, nectary absent; limen absent, limen floor 4.7–7.1 mm in diameter, very dark reddish purple; androgynophore 2.7–4.1 mm long, 0.7–1.3 mm wide; free portions of the staminal filaments 2.3–3.6 mm long, 0.4–0.7 mm wide, linear, very dark reddish purple; anthers 1.6–3.1 mm long, 0.7–1.7 mm wide, extrorse at anthesis with their axes maintained parallel to the filament; styles 4.1–6.3 mm long including stigmas, 0.3–0.5 mm wide, greenish yellow with very dark reddish purple tinge; stigmas 0.7–1.6 mm in diameter; ovary 1.3–3.7 mm long, 1.4–2.7 mm wide, widely ellipsoid to globose, greenish yellow. Berry 14.4–26.0 mm long, 12.5–19.00 mm in diameter, widely ellipsoid to ovoid, greenish yellow with white spots, becoming soft at the base at maturity. Seeds ca. 10, 5.0–6.1 mm long, 2.1–2.7 mm wide, 1.3–1.9 mm thick, widely elliptic in outline, acute at both ends, reticulate-foveate with each face marked with 12–24 foveae. Germination type epigeal.

##### Phenology.

Flowering and fruiting September to June.

##### Distribution.

Belize, Guatemala, and Mexico. Tropical semideciduous forests (selva mediana subcaducifolia and selva mediana subperennifolia); growing in shrubs or trailing along the ground on soil of little depth, lying directly on top of limestone; 20–500 m.

##### Discussion.

As discussed under the description of *Passiflora itzensis*, I have chosen to recognize MacDougal’s two subspecies of *Passiflora xiikzodz* at the species level. The work of MacDougal and molecular and morphological analyses presented here support the specific recognition of this very distinct taxon. *Passiflora xiikzodz* is vegetatively identical to *Passiflora itzensis*, but numerous floral characters may be used to separate them (see description of *Passiflora itzensis*). The most obvious difference between these species is the extreme reduction or lack of an androgynophore in *Passiflora itzensis*. *Passiflora sexocellata* is also vegetatively similar to *Passiflora xiikzodz*. However, these species differ in the position of the petiolar nectaries, with *Passiflora xiikzodz* having nectaries positioned toward the apex of the petiole and *Passiflora sexocellata* possessing nectaries at the middle or on the proximal half of the petiole.

In Belize, Meerman (2001) found that *Heliconius erato* is an herbivore of *Passiflora xiikzodz*.

##### Specimens examined.

**MEXICO. Campeche:** Hwy. 186 between Catamul and Xpujil, km 105, 210 m, 16°31.27N, 89°49.58W, *Porter-Utley & Mondragón 387* (CICY, FLAS); 1 km S de Zoh Laguna, Hopelchén, 18°35.00N, 89°25.00W, *Simá 1382* (CICY). **Quintana Roo:** 16 km S de San José de la Montaña, sobre el camino a Tomás Garrido, *Cabrera & Cabrera 5565* (F, MO); Calica, 7.5 km S de Playa del Carmen, Cozumel, 20°34.25N, 87°08.00W, *Duran et al. 2272* (CICY); Jardín Botánico, Benito Juárez, 20°50.30N, 86°54.00W, *Duran & Cruz 2369* (CICY); road off of Hwy. 186 between Xpujil and San Francisco Villa, 260 m, 18°29.94N, 89°21.40W, *Porter-Utley & Mondragón 391* (CICY, FLAS); Hwy. 307 between Chetumal and Cancún, 30 m, 18°50.88N, 88°16.46W, *Porter-Utley & Mondragón 394* (CICY, FLAS); road between of Hwy. 307 and 180 between Punta Nizuc and Alfredo V. Bonfil, 20 m, 21°02.40N, 86°53.30W, *Porter-Utley & Mondragón 401* (CICY, FLAS); 3.4 km hacia Nuevo Becal, partiendo de la carretera de Zoh-Laguna a Chunchintok, Hopelchén, 18°37.00N, 89°22.35W, *Trejo et al. 582* (CICY). **Yucatán:** camino de Temozán a Xluch, 10 m, *Chan 3540* (CICY); Chichén Itzá, *Lundell & Lundell 7470* (TEX, US); road off main hwy. (no number) between Vallodolid and Tinum, 20 m, 20°44.32N, 88°21.48W, *Porter-Utley & Mondragón 408* (FLAS); 3 km de Tinum rumbo a San Francisco, 23 m, *Ucan 2303* (CICY).

**BELIZE. Cayo:** near Camp 6, *Gentle 2377* (GH); along Macal River, *Hodges & Klassi 20* (MO); Ruins of Xunantunich, *MacDougal 4677* (MO); Crist O Rey, 350 m, *Monro 1101* (BM); Xunantunich (Maya ruins), 600–700 ft., *Proctor 29617* (BM); 1 mi. NE of Benque Viejo on road to Xunantunich, near the ferry, *Turner s.n.*, 25 March 1990 (MO). **Corozal:** Cerro maya Ruins, Lowry’s Bight, *Crane 513* (TEX); *Gentle 255* (US). **No Specific Locality In Belize Given:** *Gaumer 24. 415* (G); Jacinto Hills, 400 ft., *Schipp S-603* (F).

**GUATEMALA. Petén:** Tikal National Park, in ramonal, on Pinar Road about 6 km N, *Contreras 3825* (TEX); Santa Elena, on La Libertad Road, km 5, *Contreras 6083* (TEX); Dos Lagunas, 5 km W on Carmelita Road, *Contreras 8478* (CAS, F, NY, TEX); 8 km N del poblado Melchor de Mencos, frontera con Belice, 17°21'02"N, 89°13'05", *Durán et al. 3281* (CICY); Lake Petén Itza, on cliff along shre E of San José, *Lundell 17235* (MO, TEX); La Cumbre, San Luis area, *Lundell & Contreras 20711* (TEX).

#### Excluded names

*Passiflora regalis* Ramírez Goyena, Fl. Nicarag. 1: 434. 1909-1911. Ramírez Goyena (1909) published a description of *Passiflora regalis*, which he attributed to Macfadyen, but the species that he described was also *Passiflora lancifolia* and a later homonym of *Passiflora regalis* Macf. ex Griseb. Incidentally, Ramírez Goyena’s description of *Passiflora regalis*, other than being in Spanish and not in English, is virtually identical to that of Grisebach.

The following names pubished in *Cieca* (Passifloraceae) are excluded from Passiflora supersection Cieca.

*Cieca appendiculata* (G. Mey.) M. Roemer, Fam. Nat. Syn. Monogr. 2: 145. 1846 = *Passiflora auriculata* Kunth.

*Cieca auriculata* (Kunth) M. Roemer, Fam. Nat. Syn. Monogr. 2: 143. 1846 = *Passiflora auriculata* Kunth.

*Cieca bauhiniifolia* (Kunth) M. Roemer, Fam. Nat. Syn. Monogr. 2: 145. 1846 = *Passiflora alnifolia* Kunth.

*Cieca berteroana* (Balb. ex DC.) M. Roemer, Fam. Nat. Syn. Monogr. 2: 145. 1846 = *Passiflora berteroana* Balb. ex DC. 1828.

*Cieca bilobata* (Juss.) M. Roemer, Fam. Nat. Syn. Monogr. 2: 146. 1846 = *Passiflora bilobata* Juss.

*Cieca cavanillesii* (DC.) M. Roemer, Fam. Nat. Syn. Monogr. 2: 140. 1846 = *Passiflora cupraea* L.

*Cieca cinerea* (Poepp. & Endl.) M. Roemer, Fam. Nat. Syn. Monogr. 2: 148. 1846 = *Passiflora auriculata* Kunth.

*Cieca colubrina* (Poepp. & Endl.) M. Roemer, Fam. Nat. Syn. Monogr. 2: 143. 1846 = *Passiflora triloba* Ruiz & Pav. ex DC.

*Cieca cupraea* (L.) M. Roemer, Fam. Nat. Syn. Monogr. 2: 139. 1846 = *Passiflora cupraea* L.

*Cieca dictamo* (DC.) M. Roemer, Fam. Nat. Syn. Monogr. 2: 146. 1846 = *Passiflora dictamo* DC.

*Cieca discolor* (Link & Otto) M. Roemer, Fam. Nat. Syn. Monogr. 2: 140. 1846 = *Passiflora misera* Kunth.

*Cieca glabrata* (Kunth) M. Roemer, Fam. Nat. Syn. Monogr. 2: 143. 1846 = *Passiflora biflora* Lam.

*Cieca gracilis* (J. Jacq. ex Link) M. Roemer, Fam. Nat. Syn. Monogr. 2: 141. 1846 = *Passiflora gracilis* J. Jacq. ex Link.

*Cieca guyanensis* Klotzsch, Reis. Br.-Guiana 986. Name not validly published.

*Cieca hederacea* Klotzsch, Reis. Br.-Guiana 1090. Name not validly published.

*Cieca maculata* (Scan. ex Colla) M. Roemer, Fam. Nat. Syn. Monogr. 2: 145. 1846 = *Passiflora maculata* Scan. ex Colla.

*Cieca membranacea* (Benth.) M. Roemer, Fam. Nat. Syn. Monogr. 2: 140. 1846 = *Passiflora membranacea* Benth.

*Cieca mexicana* (Juss.) M. Roemer, Fam. Nat. Syn. Monogr. 2: 146. 1846 = *Passiflora mexicana* Juss.

*Cieca misera* (Kunth) M. Roemer, Fam. Nat. Syn. Monogr. 2: 140. 1846 = *Passiflora misera* Kunth.

*Cieca multiflora* (L.) M. Roemer, Fam. Nat. Syn. Monogr. 2: 148. 1846 = *Passiflora multiflora* L.

*Cieca normalis* (L.) M. Roemer, Fam. Nat. Syn. Monogr. 2: 144. 1846 = Passiflora perfoliata var. normalis Fawc. & Rendle.

*Cieca pannosa* (Sm.) M. Roemer, Fam. Nat. Syn. Monogr. 2: 148. 1846 = *Passiflora sexflora* Juss.

*Cieca porophylla* (Vell.) M. Roemer, Fam. Nat. Syn. Monogr. 2: 147. 1846 = *Passiflora organensis* Gardner.

*Cieca pubescens* (Kunth) M. Roemer, Fam. Nat. Syn. Monogr. 2: 141. 1846 = *Passiflora capsularis* L.

*Cieca sururuca* (Vell.) M. Roemer, Fam. Nat. Syn. Monogr. 2: 141. 1846 = *Passiflora setacea* DC.

*Cieca trisetosa* (DC.) M. Roemer, Fam. Nat. Syn. Monogr. 2: 147. 1846 = *Passiflora jorullensis* Kunth.

*Cieca variolata* (Poepp. & Endl.) M. Roemer, Fam. Nat. Syn. Monogr. 2: 140. 1846 = *Passiflora variolata* Poepp. & Endl.

*Cieca vellozii* (Gardner) M. Roemer, Fam. Nat. Syn. Monogr. 2: 142. 1846 = *Passiflora vellozii* Gardner.

## Supplementary Material

XML Treatment for Passiflora

XML Treatment for Passiflora subgenus Decaloba (DC.) Reichenbach supersection Cieca

XML Treatment for Passiflora pallida

XML Treatment for Passiflora suberosa

XML Treatment for Passiflora suberosa subsp. suberosa

XML Treatment for Passiflora suberosa L. subsp. litoralis

XML Treatment for Passiflora tridactylites

XML Treatment for Passiflora lancifolia

XML Treatment for Passiflora macfadyenii

XML Treatment for Passiflora tenuiloba

XML Treatment for Passiflora eglandulosa

XML Treatment for Passiflora trinifolia

XML Treatment for Passiflora clypeophylla

XML Treatment for Passiflora obtusifolia

XML Treatment for Passiflora juliana

XML Treatment for Passiflora viridiflora

XML Treatment for Passiflora mcvaughiana

XML Treatment for Passiflora tacanensis

XML Treatment for Passiflora coriacea

XML Treatment for Passiflora megacoriacea

XML Treatment for Passiflora sexocellata

XML Treatment for Passiflora itzensis

XML Treatment for Passiflora xiikzodz

## Appendix 1

**List of taxa:** List of taxa used in the ITS-1 and ITS-2 analysis, with voucher information and GenBank accession numbers.

Taxon	Voucher	GenBank #
*Passiflora citrina*	Porter-Utley P-60	JX463165
*Passiflora coriacea*	Porter-Utley P-66	JX463147
*Passiflora inca*	Weigend & Weigend 425	JX463163
*Passiflora itzensis*	Porter-Utley P-69	JX463101
*Passiflora juliana*	MacDougal 492GR	JX463152
*Passiflora juliana*	Porter-Utley & Mondragon 355	JX463153
*Passiflora juliana*	Porter-Utley & Mondragon 357	JX463154
*Passiflora lancifolia*	Porter-Utley P-51	JX463158
*Passiflora lobata*	MacDougal 414	JX463164
*Passiflora lobbii*	Weigend 385	JX463162
*Passiflora mcvaughiana*	Cuevas & Guzman 4198	JX463148
*Passiflora mcvaughiana*	Porter-Utley & Mondragon 345	JX463149
*Passiflora obtusifolia*	Porter-Utley P-67	JX463150
*Passiflora obtusifolia*	Porter-Utley s.n.	JX463151
*Passiflora pallida*	Hammel 1778	JX463131
*Passiflora pallida*	Porter-Utley P-65	JX463132
*Passiflora pallida*	Porter-Utley & Mondragon 393	JX463135
*Passiflora pallida*	Porter-Utley & Mondragon 398	JX463136
*Passiflora pallida*	Porter-Utley & Mondragon 403	JX463137
*Passiflora pallida*	Porter-Utley & Mondragon 405	JX463138
*Passiflora pallida*	Porter-Utley & Mondragon 407	JX463139
*Passiflora pallida*	Porter-Utley & Mondragon 412	JX463140
*Passiflora pallida*	Kay 208	JX463141
*Passiflora pallida*	Porter-Utley & Paul P-57	JX463142
*Passiflora penduliflora*	Porter-Utley & Paul P-53	JX463166
*Passiflora perfoliata*	Porter-Utley & Paul P-55	JX463167
*Passiflora podlechii*	Weigend & Weigend 369	JX463161
*Passiflora sexflora*	Porter-Utley & Paul P-48	JX463168
*Passiflora sexocellata*	Porter-Utley & Mondragon 311	JX463143
*Passiflora sexocellata*	Porter-Utley & Mondragon 314	JX463144
*Passiflora sexocellata*	Porter-Utley & Mondragon 326	JX463145
*Passiflora sexocellata*	Porter-Utley & Mondragon 384	JX463146
Passiflora suberosa subsp. litoralis	Porter-Utley P-64	JX463107
Passiflora suberosa subsp. litoralis	Porter-Utley & Mondragon 333	JX463113
Passiflora suberosa subsp. litoralis	Porter-Utley & Mondragon 343	JX463114
Passiflora suberosa subsp. litoralis	Porter-Utley & Mondragon 344 clone a	JX463115
Passiflora suberosa subsp. litoralis	Porter-Utley & Mondragon 344 clone b	JX463116
Passiflora suberosa subsp. litoralis	Porter-Utley & Mondragon 344 clone c	JX463117
Passiflora suberosa subsp. litoralis	Porter-Utley & Mondragon 344 clone d	JX463118
Passiflora suberosa subsp. litoralis	Porter-Utley & Mondragon 332 clone a	JX463112
Passiflora suberosa subsp. litoralis	Porter-Utley & Mondragon 332 clone b	JX463123
Passiflora suberosa subsp. litoralis	Porter-Utley & Mondragon 332 clone c	JX463124
Passiflora suberosa subsp. litoralis	Porter-Utley & Mondragon 332 clone d	JX463125
Passiflora suberosa subsp. litoralis	Porter-Utley & Mondragon 332 clone e	JX463126
Passiflora suberosa subsp. litoralis	Porter-Utley & Mondragon 413	JX463133
Passiflora suberosa subsp. litoralis	Porter-Utley & Mondragon 414	JX463134
Passiflora suberosa subsp. suberosa	Porter-Utley P-4	JX463108
Passiflora suberosa subsp. suberosa	Kay 204	JX463111
Passiflora suberosa subsp. suberosa	Paul & Porter-Utley AP504 clone a	JX463119
Passiflora suberosa subsp. suberosa	Paul & Porter-Utley AP504 clone b	JX463120
Passiflora suberosa subsp. suberosa	Paul & Porter-Utley AP504 clone c	JX463121
Passiflora suberosa subsp. suberosa	Paul & Porter-Utley AP504 clone d	JX463122
Passiflora subersoa subsp. litoralis	Porter-Utley P-58	JX463109
Passiflora subersoa subsp. suberosa	Porter-Utley P-63	JX463110
*Passiflora subersoa* with *pallida* aff.	Abbott 14284 clone a	JX463127
*Passiflora subersoa* with *pallida* aff.	Abbott 14284 clone b	JX463128
*Passiflora subersoa* with *pallida* aff.	Abbott 14284 clone c	JX463129
*Passiflora subersoa* with *pallida* aff.	Abbott 14284 clone d	JX463130
*Passiflora tenuiloba*	MacDougal 227	JX463159
*Passiflora tenuiloba*	Ward 5683	JX463160
*Passiflora viridiflora*	Porter-Utley & Mondragon 362	JX463155
*Passiflora viridiflora*	Porter-Utley & Mondragon 374	JX463156
*Passiflora viridiflora*	Porter-Utley & Mondragon 366	JX463157
*Passiflora xiikzodz*	Porter-Utley & Mondragon 387	JX463102
*Passiflora xiikzodz*	Porter-Utley & Mondragon 391	JX463103
*Passiflora xiikzodz*	Porter-Utley & Mondragon 401	JX463104
*Passiflora xiikzodz*	Porter-Utley & Mondragon 408	JX463105
*Passiflora xiikzodz*	Contreras 8478	JX463106

## Appendix 2

Index to Numbered Collections

Abbott, J. & K. Williams 8461 (pallida)

Abbott, R. 335 (suberosa subsp. litoralis); 14284 (pallida)

Abbott, W. 1469 (suberosa subsp. suberosa); 1724 (pallida); 2507, 2834 (suberosa subsp. suberosa); 1715a (pallida)

Abruzzi, M. 814 (suberosa subsp. litoralis)

Acevedo, P. 405 (megacoriacea); 2434, 3133 (suberosa subsp. suberosa))

Acevedo, P. & D. Chinea 3006 (suberosa subsp. suberosa)

Acevedo, P. & A. Siaca 4075, 4302 (pallida)

Acevedo, P. et al. 639 (pallida); 2063 (suberosa subsp. suberosa); 2330 (pallida); 5196 (suberosa subsp. suberosa); 6503, 8480 (pallida)

Acosta, R. & F. Vazquez 634 (pallida)

Adams, C. 5496 (pallida); 5723, 8152 (lancifolia); 8327 (pallida); 10232 (macfadyenii); 12258 (pallida)

Aguilar, I. 89 (eglandulosa); 211, 390 (suberosa subsp. litoralis)

Aguilar, J. 1773 (suberosa subsp. litoralis)

Alcorn, J.3316 (suberosa subsp. litoralis)

Allard, H. 16368 (pallida); 16370 (suberosa subsp. suberosa); 17783a (pallida)

Allemao, F. & M. Cysneiros 726 (suberosa subsp. litoralis)

Allen, P. 1667, 4473, 5526 (megacoriacea); 6570 (sexocellata)

Almeda, F. 367 (pallida)

Alston, A. 5457 (suberosa subsp. litoralis)

Alvarado 28 (megacoriacea)

Alvarez, D. 466 (pallida)

Alvarez, S. et al. 7627 (tenuiloba)

Alvaroado, F. & IV Curso de Parataxonomos 59c (megacoriacea)

Anderson, E. 2480 (suberosa subsp. litoralis)

Anderson, W. & Sternberg 3238 (pallida)

Andrews, L. 251 (pallida); 541 (suberosa subsp. litoralis)

Apolinar-Maria, B. 309 (suberosa subsp. litoralis)

Araquistain, M. 3171-b (sexocellata)

Arbelaez, G. et al. 970 (suberosa subsp. litoralis)

Archer, W. 152, 370, 759, 1009, 1041 (suberosa subsp. litoralis)

Arguelles, E. 909 (suberosa subsp. litoralis)

Asplund, E. 7686, 13903, 15217 (suberosa subsp. litoralis); 16532 (coriacea)

Atherton, N. 1 (pallida)

Atwater, W. 684 (pallida)

Austin, D. & E. Conroy 4724 (pallida)

Axelrod, F. & A. Axelrod 2346, 4430 (pallida); 8546 (suberosa subsp. suberosa)

Axelrod, F. & M. Nir 8329, 8331 (suberosa subsp. suberosa)

Axelrod, F. & D. Roubik 3854 (suberosa subsp. suberosa)

Axelrod, F. et al. 4158, 8382 (suberosa subsp. suberosa)

Ayala, G. 1212 (juliana)

Baber 4947 (pallida)

Bacigalupo, N. & N. Deginani 16 (suberosa subsp. litoralis)

Bailey, L. 15109 (pallida)

Baker, C. 2617 (suberosa subsp. litoralis)

Balakrishnan, N. & M. Jayasuriaya NBK911 (pallida)

Baldwin, A. 45 (pallida)

Baltzell, L. 6619 (pallida)

Barbeau, G. 31838 (pallida)

Barclay, G. 1966 (viridiflora); 764EC (suberosa subsp. litoralis)

Barkley, F. 14062 (viridiflora)

Barkley, F. et al. 17M751 (viridiflora)

Bartholomew, B. & D. Boufford 6176 (suberosa subsp. litoralis)

Bartlett, H. 11010 (tenuiloba); 12011 (sexocellata)

Bartlett, H. & T. Lasser 16887 (megacoriacea)

Bartrana, A. 4645 (suberosa subsp. suberosa)

Basurto, F. & R. Patron 186 (sexocellata)

Bates, D. et al. 1479 (tenuiloba)

Baumann, M. 5126 (pallida); 6059 (suberosa subsp. litoralis); 13826 (pallida)

Baumann-Bodenheim, G. 5085 (pallida); 5184 (suberosa subsp. litoralis)

Baur, G. 160 (tridactylites)

Beck, G. 6350 (suberosa subsp. litoralis)

Beckner, J. 769, 2235 (pallida)

Beetle, A. 1394 (suberosa subsp. litoralis)

Benzon, A. 199-5098 (pallida)

Berkman, A. 3616 (tenuiloba)

Betancur, J. et al. 727 (coriacea)

Bigelow, J. 393d (tenuiloba)

Bigelow, J. & C. Parry 393b (tenuiloba)

Billberg, J. 121, 122 (pallida)

Bishop, R. P-10192 (pallida)

Blair, B. 4767 (pallida)

Blair, E. 4672 (pallida)

Blake, S. 7595 (sexocellata)

Blanchet, J. 117, 193, 1171 (suberosa subsp. litoralis)

Blanco, M. 2260 (suberosa subsp. litoralis)

Blooding, E. 128 (suberosa subsp. suberosa)

Boege, W. 804 (suberosa subsp. litoralis)

Boldingh, I. 2734 (suberosa subsp. suberosa); 2757, 2764 (pallida); 2834, 2871 (suberosa subsp. suberosa); 3498, 4726, 4926, 5107, 6515, 7042, 7203, 7396 (pallida); 1668B, 192B, 2721B, 313B, 342B, 490B, 690B, 713B, 837B (suberosa subsp. suberosa)

Borgeneu, F. 39 (suberosa subsp. suberosa)

Bouché, P. 324 (pallida)

Box, H. 894, 895 (pallida); 895 (suberosa subsp. suberosa); 1258 (pallida); 1290, 1294, 1881 (suberosa subsp. suberosa)

Brace, J. 224 (pallida)

Brace, L. 1955, 4110, 4414, 4774 (pallida); 4810 (suberosa subsp. suberosa); 5292 (pallida)

Brade, A. & F. Allemao 24384 (suberosa subsp. litoralis)

Brandbyge, J. & F. Astholm 156 (suberosa subsp. litoralis)

Branzer, B. 702412, 703501 (suberosa subsp. litoralis)

Brass, L. 14912, 18081, 21153 (pallida)

Bray, W. 164 (tenuiloba)

Breckon, G. & M. Breckon 764 (viridiflora)

Breedlove, D. 7706 (suberosa subsp. litoralis); 38111, 47251 (sexocellata); 51972 (suberosa subsp. litoralis); 52183 (obtusifolia)

Breedlove, D. & F. Almeda 45168, 65224 (suberosa subsp. litoralis)

Breedlove, D. & G. Davidse 54010, 54392 (suberosa subsp. litoralis)

Breedlove, D. & P. Raven 13212 (suberosa subsp. litoralis)

Brenes, A. 12289 (megacoriacea)

Breteler, F. 4287 (suberosa subsp. litoralis)

Bridges, E. & L. Woodruff 13121 (tenuiloba)

Britton, E. 407 (pallida); 6415 (suberosa subsp. suberosa)

Britton, E. & D. Marble 1228 (suberosa subsp. suberosa)

Britton, N. 127, 143, 158, 446, 463, 2019 (pallida)

Britton, N. & W. Fishlock 1005 (pallida)

Britton, N. & C. Millspaugh 2189 (suberosa subsp. suberosa); 2463, 2474, 6174 (pallida)

Britton, N. & J. Shafer 587 (suberosa subsp. suberosa); 2975 (pallida)

Britton, N. & W. Wheeler 38, 80 (suberosa subsp. suberosa)

Britton, N. & P. Wilson 130, 193, 483 (pallida)

Britton, N. et al. 28, 179, 584 (pallida); 1763 (suberosa subsp. suberosa); 1764, 4637 (pallida); 4857 (suberosa subsp. suberosa); 5030, 5899 (pallida)

Bro. Alain 2278 (pallida); 13235 (suberosa subsp. suberosa)

Bro. Alain & P. Liogier 24531 (suberosa subsp. suberosa); 26347 (pallida)

Bro. Alain et al. 28005 (suberosa subsp. suberosa)

Bro. Augusto 425 (pallida)

Bro. Clemente 4304, 5139, 5187, 5309, 5779, 5781, 5784, 5829, 6238, 6250 (pallida); 6298 (suberosa subsp. suberosa); 6369, 6395, 6396, 7085 (pallida)

Bro. Clemente & Bro. Chrysogone 7250 (suberosa subsp. suberosa)

Bro. Daniel 1466, 3966 (suberosa subsp. litoralis); 5650 (pallida)

Broadway, H. 4236 (pallida)

Broadway, W. 285 (pallida); 291 (suberosa subsp. litoralis); 340, 374, 1720 (pallida)

Bros. Clemente & Bro. Alain 3940, 3941 (pallida)

Brown, E. 1448, W/206 (pallida)

Brown, L. 4884 (tenuiloba)

Brown, S. 95, 115, 718 (pallida)

Brown, S. & N. Britton 23 (pallida)

Brumbach, W. 6055, 7749, 7770 (pallida)

Brunt, M. 2144, 2164 (pallida)

Bryant, O. 4 (pallida)

Bueno, O. 2584 (suberosa subsp. litoralis)

Bunting, G. 2645 (suberosa subsp. litoralis)

Bunting, G. et al. 12264 (coriacea)

Burger, W. & M. Burger 8480 (megacoriacea)

Burger, W. & R. Liesner 6868 (megacoriacea)

Butterwick, M. & J. Lamb 1975 (tenuiloba)

Butterwick, M. & E. Lott 3645 (tenuiloba)

Butterwick, M. & J. Smith 476 (tenuiloba)

Butterwick, M. et al. 316 (tenuiloba)

Byrne, R. 242, 319 (pallida)

Caballero, L. & Y. Sosa 254 (pallida)

Cabrera, E. 1475 (pallida)

Cabrera, E. & H. Cabrera 3556 (pallida); 5565 (xiikzodz); 7834, 9333, 10526, 10575 (pallida)

Cabrera, E. & S. Larate 1545 (pallida)

Cabrera, E. et al. 2584, 4475 (pallida)

Caceres, S. et al. 309 (suberosa subsp. litoralis)

Calderon, S. 829 (sexocellata)

Calizada, J. et al. 6540 (pallida)

Callejas, R. & A. Bornstein 11031 (pallida)

Callejas, R. & O. Escobar 3270 (suberosa subsp. litoralis)

Calzada, J. 1838, 5862 (pallida)

Calzada, J. et al. 3804 (sexocellata)

Cameron, C. 100 (tenuiloba)

Camp, W. 2945, E-2945, E-3559 (suberosa subsp. litoralis)

Cantif, A. 39 (pallida)

Cardenas, M. 1196 (coriacea); 4707 (suberosa subsp. litoralis)

Carnevali, G. 5120 (pallida)

Carnevali, G. & F. May-Pat 5946 (pallida)

Carnevali, G. & I. Ramirez 4270, 5588 (pallida)

Carnevali, G. et al. 5675 (pallida)

Carr, D. & K. Mullen 1018 (pallida)

Carr, W. 9090, 14560b (tenuiloba)

Carr, W. & D. Hernandez 14374 (pallida)

Carranza, E. 2819 (suberosa subsp. litoralis)

Carsuer, E. & R. Studhalter 4338 (tenuiloba)

Casas, F. & M. Valverde 10741 (pallida)

Castillo, G. & M. Medina 2872 (pallida)

Castillo, G. & L. Tapis 764 (suberosa subsp. litoralis)

Castillo, G. & P. Zamora 6302 (viridiflora); 7296 (pallida); 7860 (suberosa subsp. litoralis)

Castillo, G. et al. 6599 (viridiflora)

Castillo, J. et al. 82.347 (eglandulosa)

Castillo-Campos, G. & I. Acosta 16155 (sexocellata)

Castillo-Campos, G. & P. Zamora 8601, 8660 (pallida)

Cerrate, E. 2724, 3623 (suberosa subsp. litoralis)

Chacon, I. 778 (megacoriacea)

Chan, C. 3540 (xiikzodz); 3790 (sexocellata); 6370 (pallida); 5719a (sexocellata)

Chanek, M. 4 (sexocellata)

Chang, H. 2107 (suberosa subsp. litoralis)

Chase, A. 9262 (suberosa subsp. litoralis)

Chavarria, M. & A. Solis 955 (megacoriacea)

Chavelas, J. et al. 2692 (sexocellata)

Chavez, M. & C. Kerbel 327 (sexocellata)

Chazaro, M. & P. Hernandez 3331 (pallida)

Chazaro, M. & H. Oliva 2319 (suberosa subsp. litoralis)

Cheesman, L. 3319 (pallida)

Chernin, M. 844 (pallida)

Chiang, F. 158 (suberosa subsp. litoralis); 275 (pallida)

Chiang, F. et al. 721 (viridiflora); 2106, 2037b (suberosa subsp. litoralis)

Christenson, E. 185 (pallida)

Chuang, C. & M. Kao 3950 (suberosa subsp. litoralis)

Clark, O. 4115 (tenuiloba)

Clemens, J. & M. Clemens 7981 (pallida)

Clewell, A. & D. Hazlett 3858 (eglandulosa)

Cobin, M. & R. Woodbury MB274 (pallida)

Coker, W. 228 (pallida)

Colella, M. & V. Morales 731 (suberosa subsp. litoralis)

Colella, M. et al. 1304 (pallida)

Colhida 13 (suberosa subsp. litoralis)

Collins, F. 235 (pallida)

Cols, H. 370 (pallida)

Comanor, P. 324 (pallida)

Combs, R. 50 (pallida); 50 (suberosa subsp. suberosa); 304 (pallida)

Conduz 10903 (suberosa subsp. litoralis)

Conrad, J. & R. Conrad 2985 (sexocellata)

Contreras, E. 716, 1005, 2349, 3122 (sexocellata); 3825, 5578, 6083 (xiikzodz); 6795, 7229, 7282, 7815 (sexocellata); 8478 (xiikzodz); 8687 (pallida); 8935 (sexocellata)

Conzatti, C. 2183 (suberosa subsp. litoralis); 4419.5 (obtusifolia); 4501 (viridiflora)

Cook, O. 48 (suberosa subsp. suberosa)

Cooley, G. et al. 9155, 9157, 9434 (pallida)

Cornejo, X. & C. Bonitaz 884 (suberosa subsp. litoralis)

Cornejo, X. et al. 4034 (suberosa subsp. litoralis)

Correll, D. 14852 (pallida); 31586 (tenuiloba); 40996, 43434, 44459, 46295 (pallida)

Correll, D. & H. Correll 26134 (tenuiloba); 41995, 42395, 43669 (pallida); 43707, 45674 (suberosa subsp. suberosa)

Correll, D. & C. Hanson 29886 (tenuiloba)

Correll, D. & I. Johnston 18048, 18269, 21241 (tenuiloba)

Correll, D. & F. Meyer 44600 (pallida)

Correll, D. & R. Rollins 32551 (tenuiloba)

Correll, D. & C. Schweinfurth 25413 (tenuiloba)

Correll, D. & D. Wasshausen 27734 (tenuiloba); 46856, 52077 (pallida)

Correll, D. et al. 40198, 45960 (pallida)

Cortes, M. 482 (pallida)

Cory, V. 17227, 35655 (tenuiloba)

Costa Solis, M. 8009 (suberosa subsp. litoralis)

Coulter, T. 58, 59 (suberosa subsp. litoralis)

Cowan, C. 2314, 2815 (sexocellata)

Cowan, R. 892 (suberosa subsp. suberosa)

Cowles, H. 5-28, 8-18, 8-29, 10-5, 10-15, n10-5, n5-26 (pallida)

Crane, C. 513 (xiikzodz)

Crawford, J. 115, 680, 681, 737, 742, 759, 824 (pallida)

Croat, T. 6732 (megacoriacea); 21515 (suberosa subsp. litoralis); 34557 (megacoriacea); 39610 (sexocellata); 42222 (eglandulosa); 66095 (sexocellata); 5414A (megacoriacea)

Croat, T. & G. Zhu 76290 (megacoriacea)

Croft, M. 64 (tenuiloba)

Crosby, M. & W. Anderson 1233 (pallida)

Cuadros, H. 4576 (pallida)

Cuatrecasas, J. 3245 (coriacea); 15341, 15371, 22864 (suberosa subsp. litoralis)

Cuatrecasas, J. & R. Romero 24402 (suberosa subsp. litoralis)

Cuatrecasas, J. et al. 12081B (suberosa subsp. litoralis)

Cuevas, R. & L. Guzman 4198 (mcvaughiana)

Cumana, L. & H. Ceequea 4229 (suberosa subsp. litoralis)

Cuming, H. 1047 (suberosa subsp. litoralis)

Curtiss, A. 552 (pallida)

Curran, H. & M. Haman 184, 205 (pallida)

Curtiss,A. 176, 552, 973, 974, 5641 (pallida)

Curtiss, A. & P. Lennon 974 (pallida)

da Costa, J. 701 (suberosa subsp. litoralis)

Darcy, W. 462B (pallida)

D’Arcy, W. 113, 253 (suberosa subsp. suberosa); 2248, 2851, 4834, 4941 (pallida); 9527 (megacoriacea); 17538, 462A (pallida)

D’Arcy, W. & K. Sytsma 14553 (megacoriacea)

Dash, J. 518 (suberosa subsp. suberosa)

Davidse, G. et al. 30715 (sexocellata)

Davis, J. 156 (pallida)

de Wilde, P. 44a (pallida)

Deam, C. 10 (sexocellata); 6193 (suberosa subsp. litoralis); 60971, 60991, 61084 (pallida)

Degener, O. 1035 (pallida); 7112 (suberosa subsp. suberosa); 18853, 18917, 18929, 18964 (pallida)

Degener, O. & T. Murashige 20030 (pallida)

Degener, O. & E. Orndonez 13788, 13801 (suberosa subsp. litoralis)

Degener, O. & K. Park 9947 (suberosa subsp. suberosa)

Degener, O. et al. 11866 (suberosa subsp. suberosa)

Deginani, N. & C. Taylor 1394 (suberosa subsp. litoralis)

Deginani, N. et al. 900 (suberosa subsp. litoralis)

Diaz, C. et al. 7426, 7472 (suberosa subsp. litoralis)

Diaz, V. 5 (megacoriacea)

Didrichsen, D. 4398 (suberosa subsp. litoralis)

Dieckman, L. 263A (pallida)

Dillon, M. et al. 3042 (suberosa subsp. litoralis)

Dodson, C. & A. Gentry 12562 (suberosa subsp. litoralis)

Dorantes, J. 5281 (pallida); 5281 (suberosa subsp. litoralis)

Dorr, L. & T. Atkins 2240 (tenuiloba)

Dugand, A. & H. Garcia 2277 (pallida)

Dugand, A. & R. Jaramillo 2844 (pallida); 4670 (coriacea)

Duke, J. 6502, 8874 (pallida); 10084, 13270 (megacoriacea)

Dunbar, H. 198 (pallida)

Dunn, D. et al. 17554 (sexocellata)

Duran, R. 2368 (pallida)

Duran, R. & D. Cruz 2356, 2357 (pallida); 2369 (xiikzodz)

Duran, R. et al. 2606 (pallida)

Dusen, P. 9960, 14972, 15932, 18020 (suberosa subsp. litoralis)

Duss, P. 873, 874, 876 (suberosa subsp. suberosa); 886, 2626 (pallida); 3538 (suberosa subsp. suberosa); 3539 (pallida); 3540, 3563 (suberosa subsp. suberosa); 3616 (pallida); 3909 (suberosa subsp. suberosa); 3929, 4690 (pallida)

Dwyer, J. 11002, 11063 (sexocellata); S-40 (megacoriacea)

Dwyer, J. & M. Correa 9397 (megacoriacea)

Dwyer, J. & R. Liesner 12214 (sexocellata)

Dwyer, J. et al. 73 (sexocellata)

Earle, F. 47, 750 (pallida)

Eaton, A. 216, 275.5, 407, 935 (pallida)

Ebinger, J. 229 (megacoriacea)

Edwards, L. & T. Lawrence 9 (pallida)

Edwards, M. 583 (pallida); 924 (suberosa subsp. litoralis)

Eggers, H. 169 (pallida); 437, 446, 547 (suberosa subsp. suberosa); 765 (pallida); 3013 (suberosa subsp. suberosa); 4051, 4406, 4873 (pallida); 13440, 15237, 15583 (suberosa subsp. litoralis)

Egler, F. 39-211 (suberosa subsp. suberosa)

Ehb. 32 (pallida)

Eiten, G. & L. Eiten 10892 (suberosa subsp. litoralis)

Ekman, E. 822, 950, 1025, 1431 (pallida); 1511 (suberosa subsp. litoralis); 2025 (suberosa subsp. suberosa); 2069 (pallida); 2607, 2696 (suberosa subsp. suberosa); 2708, 3718, 3925, 4737, 4925 (pallida); 5045, 5663, 5665, 6908 (suberosa subsp. suberosa); 7551, 7977 (pallida); 8844 (suberosa subsp. suberosa); 9347, 9837, 12349 (pallida); 12355, 14073 (suberosa subsp. suberosa)

Elder, F. 398 (pallida)

Elias, T. 1692 (pallida)

Eliasson, I. 1017, 1218, 1282 (tridactylites)

Ellenberg, H. 2764 (suberosa subsp. litoralis)

Emygdio, L. & J. Reute 2012 (suberosa subsp. litoralis)

Engle 1176, 1177 (suberosa subsp. litoralis)

Ernst, W. 1265 (suberosa subsp. suberosa); 2561 (viridiflora)

Ervendberg, L. 211 (sexocellata)

Escalante, S. 127 (itzensis)

Escobar, L. 1045 (coriacea)

Escobar, L. & J. Brand 2059, 2059b (suberosa subsp. litoralis)

Escobar, L. & F. Roldan 8675 (coriacea)

Escobar, L. & J. Santa 3466 (coriacea)

Escobar, L. & L. Uribe 416 (suberosa subsp. litoralis); 483 (coriacea)

Escobar, L. et al. 602 (tenuiloba); 2513, 3002 (suberosa subsp. litoralis); 4587 (coriacea); 3115a (suberosa subsp. litoralis)

Espejel, I. 551 (pallida)

Espejel, I. & V. Rico-Gray 119 (pallida)

Espinal, S. 2041 (coriacea)

Espinal, S. & J. Ramos 2636 (coriacea)

Espinosa, R. 1140, 2471 (suberosa subsp. litoralis); 89, 708 (megacoriacea)

Espiritu, A. & J. Martinez 18 (suberosa subsp. litoralis)

Estrada, A. 3028 (obtusifolia)

Evans, L. 78 (suberosa subsp. suberosa)

Fagerlind, F. & G. Wibon 3279 (tridactylites)

Fairchild, D. 2625 (pallida)

Fallen, M. 185 (suberosa subsp. litoralis)

Faris, J. 24, 310, 442 (pallida); 121DR (suberosa subsp. suberosa)

Farrera, O. 605 (pallida)

Feagan, J. 50 (pallida)

Fehling, G. 17 (pallida)

Fendler, A. 376 (pallida); 473 (suberosa subsp. litoralis)

Fennell, J. & P. Jones 890, 906 (pallida)

Fernandez et al. I-56 (pallida)

Fernandez, J. et al. 5480 (coriacea); 6970 (suberosa subsp. litoralis)

Fernandez-Jose, A. et al. 607 (suberosa subsp. litoralis)

Ferreyra, R. 2075, 3513, 11129, 11512, 11911, 12425, 14082, 14412A (suberosa subsp. litoralis)

Ferris, R. 5589, 5598, 6208 (obtusifolia)

Feuillet, C. 281 (suberosa subsp. suberosa)

Figueiras, L. 8094 (coriacea)

Figueiras, M. 198 (pallida)

Fishlock, W. 5 (pallida); 24, 39 (suberosa subsp. suberosa); 136 (pallida); 141, 152, 264 (suberosa subsp. suberosa)

Fleetwood, R. 3174, 3762 (pallida); 11005 (tenuiloba)

Florence, J. 2275, 9735 (suberosa subsp. litoralis)

Flores, A. & J. Martinez 851 (obtusifolia)

Flores, R. 2 (xiikzodz)

Flyr, D. 5 (tenuiloba)

Folsom, J. 228, 2288, 3466 (megacoriacea)

Fonnegra, R. et al. 2387, 2405 (suberosa subsp. litoralis)

Forero, E. et al. 3580, 10031 (coriacea)

Forero, F. et al. 1770 (coriacea)

Forment, W. 775 (viridiflora); 1125 (viridiflora)

Fosberg, F. 12286 (suberosa subsp. suberosa); 45002, 45064 (tridactylites); 46212, 48730, 48750, 48778, 49652, 49714, 50550, 52169 (pallida); 61056, 61320, 62924, 63809 (suberosa subsp. litoralis)

Fosberg, F. & W. MacNae 49515 (pallida)

Fosberg, F. & K. McKenzie 49451 (pallida)

Fosberg, F. & M. Sachet 53819, 53886 (pallida)

Fosberg, F. & D. Spellman 54461 (pallida)

Fosberg, F. & D. Stoddart 53866 (pallida)

Fosberg, F. et al. 28232 (suberosa subsp. litoralis)

Foster, R. 769, 1020, 1347, 2285 (megacoriacea)

Fr. Arnoldo 187 (pallida); 641, 796, 3229, 3387 (suberosa subsp. suberosa)

Francis MacBride, J. 2855 (suberosa subsp. litoralis)

Frazier, J. 745 (pallida)

Fredholm, A. 3076, 5545, 5608 (pallida)

Fryxell, P. & W. Anderson 3449 (sexocellata)

Fuertes, M. 160 (suberosa subsp. suberosa)

Furgensen 154 (viridiflora)

Gaillard, D. 1909 (megacoriacea)

Galeotti, H. 3657, 3659, 3661, 3663, 3665, 3667 (suberosa subsp. litoralis)

Gallardo, C. et al. 2253 (pallida)

Garcia, A. et al. 1464 (suberosa subsp. litoralis)

Garcia, C. 151 (sexocellata)

Garcia, J. & Palma 127 (sexocellata)

Garcia, R. & Jimenez 4203 (suberosa subsp. suberosa)

Garcia, R. & Pimentel 781 (pallida)

Garcia, R. et al. 4451 (pallida)

Garcia-Barriga, H. 18186, 21224 (coriacea)

Gardner, G. 50 (suberosa subsp. litoralis)

Garwood, N. 1861 A (megacoriacea)

Gaumer, G. 24.415 (xiikzodz); 1082, 1304, 2168, 2169, 23971, 24417 (pallida)

Gaumer, G. & sons 23606, 23669, 23692 (pallida); 23714 (itzensis)

Gensel, W. 57 (sexocellata)

Gentle, P. 23 (pallida); 215, 544, 821 (sexocellata); 2377 (xiikzodz); 3931 (sexocellata)

Gentry, A. 8046 (sexocellata); 12307, 16381, 16475 (suberosa subsp. litoralis)

Gentry, A. & curso prosgrado de la Universidad de San Marcos 68962 (coriacea)

Gentry, A. & V. Kapos 28403 (pallida)

Gentry, A. & D. Smith 44941 (coriacea)

Gentry, A. & UNAM Tropical Ecology Class 74432 (juliana)

Gentry, A. & T. Zanoni 50539 (suberosa subsp. suberosa)

Gentry, A. & E. Zardini 50449 (suberosa subsp. suberosa)

Gentry, A. et al. 32607 (sexocellata); 37669, 37842 (coriacea); 43989 (sexocellata); 79273 (megacoriacea)

Gentry, H. 2910 (suberosa subsp. litoralis)

German, M. et al. 1096 (sexocellata)

Gerwak, R. 361 (suberosa subsp. litoralis)

Gillis, W. 7010, 7166, 11503, 11785 (pallida)

Gines, H. 2822 (suberosa subsp. litoralis)

Giron, M. 35 (coriacea)

Glaziou, A. 5875, 21461, 5875a (suberosa subsp. litoralis)

Glocker, C. 526 (suberosa subsp. litoralis)

Godfrey, R. 65588 (pallida)

Goldberg, D. & S. McLaughlin 77-181 (suberosa subsp. litoralis)

Goldman, D. 1770, 1771, 1782, 2224 (pallida)

Goldman, D. & R. Hammer 1654 (pallida)

Goldman, D. et al. 1659 (pallida)

Goll 27, 43 (sexocellata)

Goll, G. et al. 312 (suberosa subsp. suberosa)

Gomez, L. & R. Hampshire 20137 (megacoriacea)

Gongora, E. 483 (sexocellata)

Goodwin, G. & B. Goodwin 5 (pallida)

Gradstein, S. et al. V62 (tridactylites)

Graham, J. & M. Johnston 4376 (tenuiloba); 4568 (suberosa subsp. litoralis)

Granskog, L.11 (pallida)

Grant, L. & F. Barkley 22J065 (pallida)

Grayum, M. 2782 (megacoriacea)

Grayum, M. et al. 5316 (megacoriacea)

Griffis & Broky 41A-28 (pallida)

Grimes, J. et al. 2638 (viridiflora)

Groll-Meyer, M. 271s (suberosa subsp. suberosa)

Guerrero, S. et al. 283 (suberosa subsp. litoralis)

Guillaumin, A. & A. Hurlimann 727 (pallida)

Gutierrez, C. 4265, 4442 (pallida)

Guzman, M. et al. 812 (sexocellata)

Guzman, M. 180 (obtusifolia)

Haenke, T. 870 (viridiflora); 873 (viridiflora); 1882 (megacoriacea); 873/A (viridiflora)

Hahn, L. 586 (suberosa subsp. suberosa)

Hahn, W. 1951 (suberosa subsp. litoralis)

Hallier, L. 115 (pallida)

Hammel, B. 1778, 1787, 9189, 12518 (megacoriacea)

Hammel, B. & parataxonomos del 1mer curso, INBIO 17632 (megacoriacea)

Hammel, B. et al. 17870 (megacoriacea)

Hansen, B. & M. Nee 7513 (pallida)

Hanz 32721 (suberosa subsp. litoralis)

Harling, G. 236 (suberosa subsp. litoralis)

Harling, G. & L. Andersson 21071 (suberosa subsp. litoralis)

Harris, J. 17410, 23136, 23214, C17410 (pallida)

Harris, W. 5920 (suberosa subsp. suberosa); 6536 (lancifolia); 6649, 6877, 12747 (pallida)

Harris, W. 12747 (pallida)

Harrison, J. & L. Harrison 722 (pallida)

Harrola, F. 931 (suberosa subsp. litoralis)

Hart, J. 641 (pallida); 1440 (lancifolia); 5017 (pallida)

Harvey, D. 7753 (pallida)

Hatschbach, G. 23163, 52826 (suberosa subsp. litoralis)

Hatschbach, G. & J. Silva 51299, 52483 (suberosa subsp. litoralis)

Haught, O. 3752 (coriacea); 5151 (suberosa subsp. litoralis); 13808 (coriacea); 2436CO (suberosa subsp. litoralis)

Haun 413 (pallida); 343&436 (suberosa subsp. litoralis)

Havard, V. 33, 35, 165 (pallida)

Hawkes, J. et al. 2282 (pallida)

Hawkins, T. 1657 (xiikzodz)

Hazen, T. 9652 (coriacea)

Heller, A. 6068 (pallida); 6068, 6324 (suberosa subsp. suberosa)

Heller, A. & Heller 1391 (suberosa subsp. suberosa)

Henrickson, J. 6428, 6488 (suberosa subsp. litoralis)

Henrickson, J. et al. 16078 (tenuiloba)

Henschen, L. & A. Regnelli III640 (suberosa subsp. litoralis)

Henz, E. 33.435 (suberosa subsp. litoralis)

Herat, T. & N. Wirawan 167, 188 (suberosa subsp. suberosa); 229 (pallida)

Herbst, D. 6114 (suberosa subsp. suberosa)

Herbst, D. & M. Falanruw 6791 (pallida)

Heriberto, B. 392 (coriacea)

Hernandez, H. & R. Torres 429 (viridiflora); 431 (viridiflora)

Hernandez, L. 621 (suberosa subsp. litoralis)

Hernandez, R. 3695, 3769 (suberosa subsp. litoralis)

Hernandez, R. & D. Rodriguez 4975 (suberosa subsp. litoralis)

Herndon, A. 3135 (pallida)

Herrera, G. & S. Bloemen 7632 (megacoriacea)

Herrera, J. 783a (coriacea)

Heyde, H. & E. Lux 3777 (suberosa subsp. litoralis)

Hildebrandt, J. 2963, 3264 (pallida)

Hill, S. 823 (pallida)

Hinckley, L. & L. Hinckley 347 (tenuiloba)

Hinton, G. 3030 (mcvaughiana); 4519 (suberosa subsp. litoralis); 4655 (mcvaughiana); 4700, 6533, 8216, 8607 (suberosa subsp. litoralis); 10999 (viridiflora); 13492 (suberosa subsp. litoralis)

Hitchcock, A. 105 (pallida)

Hjerting, J. et al. 38 (suberosa subsp. litoralis)

Hodge, W. 3082 (suberosa subsp. suberosa)

Hoehne, F. 6376 (suberosa subsp. litoralis)

Holdridge, L. 1128 (pallida); 1190, 1212, 1840 (suberosa subsp. suberosa)

Holmgren, I. 55 (suberosa subsp. litoralis)

Holst, B. et al. 2298 (suberosa subsp. litoralis)

Holton, I. 703 (coriacea)

Hosaka, E. 2683 (pallida)

Houghton, W. et al. 1234 (suberosa subsp. suberosa)

Howard, R. 5037, 5254 (pallida); 6507, 10777, 10790, 10859, 11111, 11266, 11435 (suberosa subsp. suberosa); 12449, 18112 (pallida)

Howard, R. & E. Howard 8509, 8688, 8689, 9265 (suberosa subsp. suberosa); 9498, 9633, 9754, 9828, 10072 (pallida); 15111 (suberosa subsp. suberosa)

Howard, R. & G. Proctor 13477, 13763, 13866 (pallida)

Howard, R. et al. 30, 441 (pallida)

Howell, G. 8747 (suberosa subsp. litoralis)

Howell, J. 9665 (tridactylites); 10492 (obtusifolia)

Hoyos, S. & J. Santa 169 (coriacea); 213, 226 (suberosa subsp. litoralis)

Hudson, J. 1117 (suberosa subsp. litoralis)

Hunnewell, F. 5866, 11040, 11583, 15320 (pallida)

Hunziker, A. & E. Cocucci 17284 (suberosa subsp. litoralis)

Hutchison, P. & D. Bennett 4514 (suberosa subsp. litoralis)

Hutchison, P. & J. Idrobo 3075 (suberosa subsp. litoralis)

Hutson, A. 27 (pallida)

Idrobo, J. & H. Weber 1368 (suberosa subsp. litoralis)

Iltis, H. 30271 (suberosa subsp. suberosa); H-610 (pallida); H-610 (suberosa subsp. litoralis)

Iltis, H. & M. Iltis 378 (suberosa subsp. suberosa)

Innes, R. & B. Moon 1285 (tenuiloba)

Jack, J. 4183, 4196, 4669 (pallida); 4807 (suberosa subsp. suberosa); 4994, 5296 (pallida); 5485, 5770 (suberosa subsp. suberosa); 6011, 6057 (pallida); 7049 (suberosa subsp. suberosa)

Jacobsen, N. 13-6 (pallida)

Jaramillo, J. et al. 748 (suberosa subsp. litoralis)

Jativa, C. & C. Epling 13 (suberosa subsp. litoralis); 426 (coriacea); 969 (suberosa subsp. litoralis)

Javier, F. & O. Marulanda 106 (suberosa subsp. litoralis)

Jayasuriya, M. 287 (pallida)

Jennings, O. 525, 528 (pallida)

Jeremie, J. 131 (suberosa subsp. suberosa); 295 (pallida); 305, 908 (suberosa subsp. suberosa)

Jimenez 492 (suberosa subsp. litoralis); 1646, 1732 (pallida)

Jimenez, J. 1419 (pallida); 1541, 2209 (suberosa subsp. suberosa); 3584 (pallida)

Jimenez, J. & E. Marcano 3389, 3391 (pallida)

Johnson, C. 725-79 (viridiflora)

Johnson, H. 273, 1054 (sexocellata)

Johnston, M. 6409, 6485 (tenuiloba); 20303 (pallida)

Johnston, M. et al. 10916, 12370 (tenuiloba)

Jones, G. et al. 3475 (sexocellata)

Josse, C. 688 (suberosa subsp. litoralis)

Juan, A. 144 (sexocellata)

Judd, W. et al. 3245 (pallida)

Judziewicz, E. & R. Guzman 5080 (mcvaughiana)

Kant, A. 21 (megacoriacea)

Kay, E. SQFR1 (pallida), 204 (suberosa subsp. litoralis), 208 (pallida)

Keil, D. & J. Canne 8837 (suberosa subsp. litoralis)

Kenoyer, L. 449 (suberosa subsp. litoralis); A515 (sexocellata)

Kenoyer, L. & H. Crum 3921 (sexocellata)

Kernan, C. 131 (megacoriacea)

Killip, E. 9726 (coriacea); 10165, 11262 (suberosa subsp. litoralis); 11442 (coriacea); 11673 (suberosa subsp. litoralis); 12039, 13523, 13828, 13941, 31667 (pallida); 34897 (suberosa subsp. litoralis); 35520 (coriacea); 40681, 40875, 40996, 41554, 41876, 43430, 43458, 44110, 44478, 44886, 45109 (pallida)

Killip, E. & T. Hazen 11023 (coriacea)

Killip, E. & A. Smith 14164, 14168, 14329 (pallida); 14415 (megacoriacea); 14479 (pallida); 14970 (coriacea); 15029 (suberosa subsp. litoralis); 16343 (coriacea); 16834, 18398 (suberosa subsp. litoralis); 19062 (coriacea); 20887 (suberosa subsp. litoralis); 21044 (pallida); 21156 (coriacea); 21524 (suberosa subsp. litoralis); 24907 (coriacea)

Killip, E. et al. 38234 (coriacea)

Kings, W. G.C.238 (pallida)

Kjellmark, E. 24 (pallida)

Klug, G. 3963 (coriacea)

Knapp, S. 8361 (coriacea)

Knapp, S. & J. Mallet 2828, 2835 (suberosa subsp. litoralis); 6356, 6877 (coriacea)

Knapp, S. & R. Schmalzel 4870 (megacoriacea)

Knobloch, I. 6035 (suberosa subsp. litoralis)

Koch, S. et al. 79191 (viridiflora); 79221 (viridiflora)

Koyama, T. et al. 16035, 16036 (suberosa subsp. litoralis)

Kral, R. 49163, 51906 (pallida)

Kramer, K. 1752 (pallida)

Krapovickas, A. & L. Mroginski 22226 (suberosa subsp. litoralis)

Krapovickas, A. & A. Schinini 30408 (suberosa subsp. litoralis)

Krapovickas, A. et al. 25533 (suberosa subsp. litoralis)

Krause, E. 15718 (suberosa subsp. suberosa)

Krukoff, B. 15923 (tenuiloba)

Kuntze, O. 218 (suberosa subsp. suberosa); 961, 6385 (pallida)

Kupper, W. 20 (megacoriacea)

Labus, Z. 86 (tenuiloba)

Ladd, M. et al. 212 (viridiflora)

Lakela, O. 27295, 28416, 31283, 31716 (pallida)

Lakela, O. et al. 305897 (pallida)

Lammers, T. 8488 (suberosa subsp. litoralis)

Lankester, C. 1298 (megacoriacea)

Larke, J. 1 (tenuiloba)

Larsen, K. & S. Larsen 35348 (suberosa subsp. suberosa)

Latorre, D. 12 (tenuiloba)

Lau, J. 1503 (suberosa subsp. suberosa); 4687F (obtusifolia)

Laudeman, C. 5288 (suberosa subsp. litoralis)

Lawesson, J. 2587, 2620, 3126 (tridactylites)

Le Gallo, C. 534B (pallida)

Leal, J. & I. Espejel 199 (pallida)

Leavenworth, W. & H. Hoogstraal 1717 (juliana)

Ledingham, G. 4666 (tenuiloba)

Lehmann, F. 1314 (trinifolia); 1849 (pallida); 2512 (suberosa subsp. litoralis); 2777 (coriacea); 3332, 3387 (suberosa subsp. litoralis); 3800 (pallida); 3852, 4579, 14579 (suberosa subsp. litoralis)

Leiter, e. 4633 (itzensis)

Lent, R. 2960 (megacoriacea)

Lentz, D. 996 (sexocellata)

Lentz, D. et al. 2344 (pallida)

Leonard, E. 3314, 3462, 3463, 3562 (pallida); 3610, 4818, 4852 (suberosa subsp. suberosa); 4997, 5135, 7140, 7662 (pallida); 7952, 8116, 8521 (suberosa subsp. suberosa); 8811, 9631, 9726, 9788 (pallida); 3038a, 7394a (suberosa subsp. suberosa)

Leonard, E. & G. Leonard 11181, 11294, 11323, 11901 (pallida); 12713 (suberosa subsp. suberosa); 13952, 13962 (pallida)

Lepiz, E. 462 (megacoriacea)

LeSueur, H. 307 (pallida); 308 (sexocellata)

Levin, G. 2046 (obtusifolia)

Lewis, W. et al. 1703, 3130 (megacoriacea)

Lewton, F. 315 (sexocellata)

Liebmann, F. 59 (pallida); 4083 (sexocellata)

Liesner, R. 184c (megacoriacea)

Liesner, R. & J. Dwyer 1531 (sexocellata)

Liesner, R. et al. 7831, 7916 (coriacea)

Lillo, M. 2400 (suberosa subsp. litoralis)

Linares, J. 3776 (obtusifolia)

Linares, J. & C. Martinez 2178 (obtusifolia); 2396 (eglandulosa)

Linden, J. 751, 755 (suberosa subsp. litoralis)

Lindman, C. 247 (suberosa subsp. litoralis)

Liogier, A. & P. Liogier 25836 (suberosa subsp. suberosa)

Lionarom 314L (suberosa subsp. suberosa)

Lloyd 1074 (pallida)

Loigier, A. 18447 (suberosa subsp. suberosa)

Long, R. et al. 2800 (pallida)

Lopez, A. et al. 8434 (suberosa subsp. litoralis)

Lopez, L. 458 (suberosa subsp. litoralis)

Lopez-Palacios, S. 2129 (suberosa subsp. litoralis)

Lorence, D. DL1559 (pallida)

Lorence, D. et al. 3722 (suberosa subsp. litoralis); 3830 (viridiflora)

Lott, E. & J. Magallanes 840 (obtusifolia)

Lott, E. et al. 2118 (suberosa subsp. litoralis); 4179 (juliana)

Lott, T. & D. Lott DT1085 (pallida)

Luna, A. 395 (suberosa subsp. suberosa)

Lundell, C. 5, 139, 636 (sexocellata); 1210 (xiikzodz); 3836, 3839, 3841 (sexocellata); 16786, 17235 (xiikzodz)

Lundell, C. & E. Contreras 19364 (sexocellata); 20711 (xiikzodz)

Lundell, C. & A. Lundell 7149 (sexocellata); 7375, 7439 (itzensis); 7446 (pallida); 7470 (itzensis); 7546, 7653, 8030 (pallida)

Lutz, A. 1788 (suberosa subsp. litoralis)

Lyonmet, P. & R. Chavez 3279 (viridiflora)

Lyonnel, P. 303 (suberosa subsp. litoralis)

Lyonnet, E. 1425 (suberosa subsp. litoralis)

Maas, P. et al. 8391 (pallida)

MacBride, J. 2855 (suberosa subsp. litoralis)

MacDougal, J. 227 (tenuiloba); 259 (pallida); 316 (eglandulosa); 349 (viridiflora); 350 (viridiflora); 351 (viridiflora); 369 (mcvaughiana); 409, 419 (megacoriacea); 421 (suberosa subsp. suberosa); 438 (suberosa subsp. litoralis); 452 (macfadyenii); 478 (suberosa subsp. litoralis); 492 (juliana); 662 (pallida); 906 (suberosa subsp. litoralis); 1204 (megacoriacea); 1486 (suberosa subsp. litoralis); 2028 (viridiflora); 3007 (sexocellata); 3025 (pallida); 3026 (suberosa subsp. suberosa); 4633 (itzensis); 4677 (xiikzodz); 6023 (suberosa subsp. suberosa); 6210 (eglandulosa); 6223, 6225, 6226, 6227, 6228 (trinifolia); 6234, 6237, 6249 (eglandulosa); 6274, 6299, 6315 (megacoriacea); 259GR (pallida); 351GR (viridiflora); 369GR (mcvaughiana); 492GR (juliana); 495GR (obtusifolia); 637GR (trinifolia)

MacDougal, J. & J. Miley 486 (juliana); 492 (juliana); 495 (obtusifolia); 637 (trinifolia)

MacDougal, J. et al. 3184, 3298 (sexocellata)

MacKee, H. 30708 (pallida)

Magallanes, J. 364 (juliana)

Magana, M. 46 (sexocellata)

Maly 1466, 1467 (pallida)

Mandon, G. 612 (suberosa subsp. litoralis)

Manuel, E. 244 (pallida); 1141 (sexocellata)

Marsh, E. 1660 (tenuiloba)

Martínez, E. 13362 (sexocellata); 20782 (tacanensis)

Martínez, E. & C. Ramos 24029-A (obtusifolia)

Martínez, E. & R. Riviere 1626 (pallida)

Martínez, E. & O. Tellez 87 (viridiflora)

Martínez, E. et al. 22859, 23602, 26047 (sexocellata); 27676, 28871 (pallida); 29363, 29711 (xiikzodz)

Mason, H. 1172, 1772 (obtusifolia); 13808, 13918 (coriacea)

Matuda, E. 6045, 17497 (sexocellata); 31332 (suberosa subsp. litoralis)

Matuda, E. et al. 31600 (suberosa subsp. litoralis)

Maxon, W. 1679 (pallida); 4002 (suberosa subsp. suberosa); 10398 (pallida)

Maxon, W. & E. Killip 338, 343, 389, 1657, 1701, 1426a, 1580a, 1655a (pallida)

Maxwell, J. 76-735 (suberosa subsp. litoralis)

Mayer, L. 72 (pallida)

Mayfield, M. et al. 861 (pallida); 871, 977 (suberosa subsp. litoralis)

McCart, W. 5790 (tenuiloba); 11155, 11226 (pallida)

McCart, W. et al. 50 (tenuiloba)

McClark, A. 4115 (tenuiloba)

McDade, L. 210 (sexocellata)

McDaniel, S. 27160 (pallida)

McFarlin, J. 3958, 9799 (pallida)

McKee, H. 1974 (pallida)

McPherson, G. 4593 (suberosa subsp. litoralis); 4591A, 4591B (pallida)

McVaugh, R. 14008 (mcvaughiana)

Mears, J. & H. Mears 1492 (tenuiloba)

Meave, J. & A. Howe 1288 (sexocellata)

Medina, M. et al. 736 (pallida)

Mehrhoff, L. 11809, 11819 (pallida); 13364 (suberosa subsp. suberosa)

Meier, W. et al. 200 (suberosa subsp. litoralis)

Meijer, W. & R. Smith 56 (coriacea)

Mejia, M. & M. Cabral 1767 (pallida)

Mejia, M. & C. Ramirez 14786 (pallida)

Mejia, M. & T. Zanoni 6080, 6193, 6283, 6545, 6566, 7139 (pallida); 8042, 8106, 8250 (suberosa subsp. suberosa); 8595, 9178, 9381, 9744 (pallida)

Mejia, M. et al. 10130, 10379 (suberosa subsp. suberosa)

Mennega, A. 4287 (suberosa subsp. litoralis)

Merit 175 (suberosa subsp. litoralis)

Mexia, Y. 666 (suberosa subsp. litoralis); 1448 (mcvaughiana); 8101 (suberosa subsp. litoralis)

Meyer, T. 2634, 3721, 8321, 9843, 13313, 35664 (suberosa subsp. litoralis)

Miers, J. 75 (suberosa subsp. litoralis)

Mille, L. 157, 42a, 42b (suberosa subsp. litoralis)

Miller 6 (coriacea)

Miller, G. 222, 225, 1116, 1117 (pallida); 316s (suberosa subsp. suberosa)

Miller, J. & M. Merello 8871 (suberosa subsp. suberosa)

Miller, J. & P. Tenorio 567 (viridiflora)

Miller, J. et al. 5670, 5686 (tenuiloba)

Miller, P. 5198 (suberosa subsp. suberosa)

Millspaugh, C. 2318 (suberosa subsp. suberosa); 2351 (pallida)

Millspaugh, C. & C. Millspaugh 9073 (pallida)

Minador 4134 (viridiflora); 4134(32) (viridiflora)

Missouri Botanical Garden 8/20/07, 113/06/56 (pallida); 13/1910 (coriacea); 146/06/14 (pallida); 15/00 (suberosa subsp. litoralis); 155/05 (pallida); 20/10/00, 283/05/13, 283/05/15 (coriacea)

Mitchell, E. 92 (sexocellata)

Mogensen, B. 1226 (pallida)

Moldenke, H. 323, 340, 516, 517, 526, 3764, 24043, 550a (pallida)

Molina, A. 14759 (sexocellata); 15243, 23340 (suberosa subsp. litoralis)

Molina, A. & A. Molina 26507 (suberosa subsp. litoralis); 27104 (sexocellata); 27543 (eglandulosa); 27552 (suberosa subsp. litoralis)

Molina, A. & E. Montalvo 21514 (eglandulosa)

Molina, A. et al. 15989 (suberosa subsp. litoralis)

Molina, J. & F. Barkley 18 N.S.101 (suberosa subsp. litoralis)

Mondragón, D. et al. 38 (sexocellata)

Monsalve, M. 3209 (suberosa subsp. litoralis)

Montaldo, J. 3672 (suberosa subsp. litoralis)

Montes, J. 14811 (suberosa subsp. litoralis)

Montes, R. 317 (suberosa subsp. litoralis)

Moore, A. 3060 (pallida)

Morales, J. 1267 (megacoriacea)

Morel, I. 1316 (suberosa subsp. litoralis)

Moreno, P. 2949, 2981 (suberosa subsp. litoralis); 16084, 16223 (sexocellata); 19449 (suberosa subsp. litoralis)

Moreno, P. et al. BD1124 (suberosa subsp. litoralis); BD-662 (pallida)

Mori, S. & C. Gracie 24052 (pallida)

Morillo, G. 2820 (suberosa subsp. litoralis)

Morrone, O. et al. 629 (suberosa subsp. litoralis)

Morton, C. 3314, 3315 (pallida); 4008, 4079, 5147 (suberosa subsp. suberosa); 7760 (sexocellata); 10492, 10518 (pallida); 10536, 10790 (suberosa subsp. suberosa)

Morton, C. & J. Acuna 2936, 3000, 3091, 3171, 4522 (pallida)

Morton, C. & Bro. Alain 8813, 8857, 9159 (pallida)

Morton, C. et al. 8727 (pallida)

Mosen, H. 1855 (suberosa subsp. litoralis)

Mosier, C. 268 (pallida)

Mroginski, L. et al. 632 (suberosa subsp. litoralis)

Muenscher, W. 12586 (sexocellata)

Muller, C. 3046 (tenuiloba)

Muller, F. 217 (pallida)

Munro, A. 1101 (xiikzodz)

Murphy, H. 638 (coriacea)

Musselman, L. et al. 5360 (pallida)

Narvaez, M. & A. Puch 354, 530 (pallida)

Narvaez, M. & E. Ucan 892 (pallida)

Nash, G. 155, 424 (pallida); 773 (suberosa subsp. suberosa); 785, 948 (pallida)

Nash, G. & N. Taylor 883, 1097, 1167, 1368, 3779 (pallida)

Nealley, G. 204 (tenuiloba)

Nee, M. 40425 (suberosa subsp. litoralis)

Nee, M. & K. Taylor 26567 (sexocellata)

Neill, D. 4281 (sexocellata)

Nelson, E. 106, 107, 2317 (viridiflora); 2446 (viridiflora)

Nelson, C. & G. Cruz 8707 (sexocellata)

Nelson, C. et al. 2832 (sexocellata); 9728 (pallida)

Nevling, L. et al. 2217 (suberosa subsp. litoralis); 2352 (pallida)

Nickerson, N. & R. Gross 3017 (pallida)

Nicolson, D. 1889, 2095 (suberosa subsp. suberosa)

Nitta, A. 15054 (suberosa subsp. litoralis)

Northrop, J. & A. Northrop 216, 242, 427, 428 (pallida)

Noumea, F. 1364 (suberosa subsp. litoralis)

Novara, L. 1625 (suberosa subsp. litoralis)

Nunez, P. 12363 (coriacea)

Nunez, P. & F. Motocanchi 8782 (suberosa subsp. litoralis)

Nunez, P. et al. 10110 (coriacea)

O’Neill, H. 8244 (pallida)

Orcutt, C. 294 (pallida); 3437 (lancifolia); 3510 (suberosa subsp. suberosa); 3808 (pallida); 3841 (lancifolia); 5838 (suberosa subsp. suberosa); 6009 (tenuiloba); 6884 (lancifolia)

Ortega, R. 49 (suberosa subsp. suberosa)

Ortiz, R. 271 (xiikzodz); 2166 (sexocellata)

Ortiz-Zuazaga, E. et al. 5PR (suberosa subsp. suberosa)

Osborne-Day, C. 124 (pallida)

Ostenfeld, C. 374 (suberosa subsp. suberosa)

Otero, J. 108 (suberosa subsp. suberosa)

Ouch, A. & M. Narvaez 239 (pallida)

Pabst, G. 5728 (suberosa subsp. litoralis)

Padilla, S. 163, 475 (obtusifolia)

Padre Fuertes 160 (pallida); 160, 1338 (suberosa subsp. suberosa)

Palaez, S. 1819 (suberosa subsp. suberosa)

Palmer, E. 185 (pallida); 207 (viridiflora); 237 (viridiflora); 10192, 12966, 13614, 33487 (tenuiloba); 185/2423 (pallida); 409b (obtusifolia)

Palmer, W. & J. Riley 194 (pallida)

Panero, J. & I. Calzada 4024 (suberosa subsp. litoralis)

Parry, C. 393c (tenuiloba)

Parry, C. et al. 393 (tenuiloba)

Paul, A. & K. Porter-Utley AP504 (suberosa subsp. suberosa); AP516 (pallida).

Paxson, J. et al. 17M633 (pallida); 17M633 (suberosa subsp. litoralis)

Pedersen, T. 1721, 4516, 11676 (suberosa subsp. litoralis)

Peguero, B. & A. Veloz 94 (suberosa subsp. suberosa)

Peguero, B. et al. 1591 (pallida)

Peirauo 9526 (suberosa subsp. litoralis)

Pennell, F. 10182 (suberosa subsp. litoralis); 10190 (coriacea); 10641, 10919 (suberosa subsp. litoralis); 12074 (pallida); 12207, 14772 (suberosa subsp. litoralis); 17985 (sexocellata); 19896 (suberosa subsp. litoralis)

Pennell, F. & E. Killip 6180 (coriacea)

Pennell, F. et al. 8500, 8581 (coriacea)

Perez, S. 71 (suberosa subsp. litoralis)

Peviano 13268 (suberosa subsp. litoralis)

Pfeifer, H. & class 2703, 2704, 2886 (suberosa subsp. suberosa)

Pflanz, K. 4005 (suberosa subsp. litoralis)

Philipson, W. 971 (lancifolia)

Pickel, D. 465 (suberosa subsp. litoralis)

Pierre, L. et al. 261 (pallida)

Pimentel, J. et al. 368 (suberosa subsp. suberosa)

Pinkava, D. et al. p5114 (tenuiloba)

Piper, C. 5479 (megacoriacea)

Pittier, H. 160 (trinifolia); 1949 (sexocellata); 3456, 3630 (megacoriacea); 4402 (pallida); 6584 (megacoriacea); 7151, 7871, 9567 (suberosa subsp. litoralis); 9783 (pallida); 11970, 12259, 13598 (suberosa subsp. litoralis)

Plowman, T. 6029 (coriacea)

Plowman, T. & E. Davis 4620 (suberosa subsp. litoralis)

Pollard, C. et al. 79, 158 (pallida); 249 (suberosa subsp. suberosa)

Popenoe, W. 818 (viridiflora)

Porrhansthy 31 (coriacea)

Porter, D. et al. 4014 (megacoriacea)

Porter-Utley, K. P-4 (suberosa subsp. suberosa); P-57 (pallida); P-58, P-63, P-64 (suberosa subsp. litoralis); P-65 (pallida); P-66 (coriacea); P-67 (obtusifolia); P-69 (itzensis)

Porter-Utley, K. & A. Paul P-51 (lancifolia)

Porter-Utley, K. & D. Mondragón 311, 314, 326 (sexocellata); 332, 333, 343, 344 (suberosa subsp. litoralis); 345 (mcvaughiana); 355 (juliana); 357 (juliana); 362 (viridiflora); 384 (sexocellata); 387, 391 (xiikzodz); 393, 398 (pallida); 401 (xiikzodz); 403, 405, 407 (pallida); 408 (xiikzodz); 412, 413 (pallida)

Posada, J. 27 (sexocellata)

Potgieter, K. 24, 26 (suberosa subsp. litoralis); 37 (pallida); 40, 41 (suberosa subsp. litoralis)

Posla, M. s.n. (obtusifolia)

Pott, V. 1812 (suberosa subsp. litoralis)

Poveda, L. et al. 3270 (megacoriacea)

Prenleloup, L. 217 (suberosa subsp. suberosa)

Prichard, E. 1156 (pallida)

Pring, G. 289/05/13 (sexocellata)

Pringle, C. 2966, 3520 (suberosa subsp. litoralis)

Pringle, J. 1924 (pallida)

Proctor, G. 7518, 9025 (pallida); 9659 (lancifolia); 10658 (suberosa subsp. suberosa); 11994 (pallida); 15884, 16172 (macfadyenii); 16888, 17151 (suberosa subsp. suberosa); 18538 (pallida); 18884 (suberosa subsp. suberosa); 22948, 23573, 23725 (lancifolia); 24913 (macfadyenii); 28057 (pallida); 29617 (xiikzodz); 33513 (lancifolia); 42509 (suberosa subsp. suberosa)

Proctor, G. & W. Gillis 33948 (pallida)

Puch, A. 777 (pallida)

Puch, A. & M. Narvaez 488 (pallida)

Puch, A. et al. 561, 572 (pallida)

Purpus, C. 203, 1272, 2067, 3543, 3544, 3545, 3547, 4072, 4073, 5048, 5049 (suberosa subsp. litoralis); 5880 (sexocellata); 7128 (suberosa subsp. litoralis); 9039 (pallida); 9259 (sexocellata); 14228, 15740, 16228 (suberosa subsp. litoralis)

Questel, A. 109 (suberosa subsp. suberosa); 323, 370 (pallida); 583 (suberosa subsp. suberosa); 585 (pallida); 586 (suberosa subsp. suberosa); 666 (pallida); 816 (suberosa subsp. suberosa); 862, 4033 (pallida); 4488 (suberosa subsp. suberosa)

Quiroz, M. 473, 507 (megacoriacea); 2349 (suberosa subsp. litoralis)

Ramamoorthy, T. et al. 2056 (xiikzodz)

Rambo, B. 29405 (suberosa subsp. litoralis)

Rambo, H. 247 (suberosa subsp. litoralis)

Ramirez, N. & M. Lopez 3273 (suberosa subsp. litoralis)

Ramos, J. 419, 621 (suberosa subsp. litoralis)

Ramos, J. & L. Ramos 2936 (coriacea)

Ramos, J. et al. 200 (tenuiloba); 2811 (coriacea); 3596 (suberosa subsp. litoralis)

Randon 6369, 6372 (suberosa subsp. litoralis)

Raunkiaer, C. 2408, 2691, 2851, 1356 (suberosa subsp. suberosa)

Ravier, R. 15 (pallida)

Ray, J. et al. 10786 (pallida)

Read, R. 232 (pallida)

Rehder, A. 909 (pallida)

Reina, A. et al. 2000-494, 98-1226, 98-1409 (suberosa subsp. litoralis)

Reitz, R. & Klein 9639 (suberosa subsp. litoralis)

Reko, B. 3753 (viridiflora); 4574 (suberosa subsp. litoralis)

Rendle, A. 520 (pallida)

Renteria, E. & curso de fitogeografia 3664, 3679 (coriacea)

Renvoize, S. 838, 829 (pallida)

Reugen, P. 199 (pallida)

Reverchou 726 (tenuiloba)

Revirosa, J. 212 (sexocellata)

Reyes, A. & G. Urquijo 769, 812, 846 (suberosa subsp. litoralis)

Reyna, O. 437 (suberosa subsp. litoralis)

Rhyne, C. 862, 439a (pallida)

Ricksecker, A. 186 (suberosa subsp. suberosa)

Ricksecker, A. & J. Ricksecker 322 (suberosa subsp. suberosa)

Rico-Gray, V. & M. Burgos 509 (pallida)

Ridgway, T. 11 (pallida)

Ridnutt, C. 12039 (suberosa subsp. litoralis)

Rimbach, A. 255 (coriacea)

Rivera, R. 25, 157 (pallida)

Robertson, S. 2484 (pallida)

Robinson, B. 134 (pallida)

Robles, R. 136, 844 (sexocellata)

Robleto, W. 667 (sexocellata)

Rodriguez, A. & J. Suarez 1451, 1456 (suberosa subsp. litoralis)

Rodriguez, D. 1123 (suberosa subsp. litoralis)

Rojas, R. 80 (suberosa subsp. litoralis)

Rojas, S. & L. Rojas 69 (suberosa subsp. litoralis)

Rojas, S. & R. Zuniga 158 (megacoriacea)

Roldan, F. & O. Marulanda 107 (suberosa subsp. litoralis)

Roldan, F. et al. 573 (pallida)

Roldan, S. R-5917 (sexocellata)

Romansky et al. 13 (pallida)

Romero, R. 761 (coriacea)

Romero-Castaneda, R. 7607 (coriacea)

Rosa, M. 43879 (suberosa subsp. litoralis)

Rosas, M. 637 (suberosa subsp. litoralis)

Rose, G. & J. Rose 18773 (suberosa subsp. litoralis)

Rose, J. 2946, 3504 (suberosa subsp. litoralis); 18110 (pallida)

Rose, J. & R. Hay 5838 (suberosa subsp. litoralis)

Rose, J. & W. Hough 4748 (suberosa subsp. litoralis)

Rose, J. & J. Painter 7395 (suberosa subsp. litoralis)

Rose, J. & G. Rose 22294, 23854 (suberosa subsp. litoralis)

Rose, J. & P. Russell 24283 (pallida)

Rose, J. et al. 3460 (suberosa subsp. suberosa); 3796 (pallida); 4164 (suberosa subsp. suberosa); 9957 (suberosa subsp. litoralis)

Rose-Innes, R. & B. Moon 1285 (tenuiloba)

Ross, R. & D. Meletische SAN308 (suberosa subsp. suberosa)

Rossbach, G. 1810 (pallida)

Rubio, D. & G. Tipaz 2334 (suberosa subsp. litoralis)

Rudatis, H. 1225 (suberosa subsp. litoralis)

Rugel, F. 169, 254, 255, 256, 257, 258 (pallida)

Ruiz, T. & Equipo de Ecologia 1974 (coriacea)

Runyon, R. 433, 512, 3094, 4906 (pallida)

Rutten-Pekelharing, C. 389 (pallida)

Rwaburindore, P. 1735 (suberosa subsp. litoralis)

Rzedowski, J. 6673, 44847, 47664 (suberosa subsp. litoralis)

Sacco, J. 2086 (suberosa subsp. litoralis)

Sachet, M. & D. Stoddart 1618 (pallida)

Safford, W. & C. Mosier 32, 227, 298, 299 (pallida)

Sagastegui, A. 388, 7330, 10950 (suberosa subsp. litoralis)

Sagastegui, A. et al. 9721 (suberosa subsp. litoralis)

Sagna 312 (pallida)

Saito, S. 7557, 8685 (suberosa subsp. litoralis)

Salas, S. & J. Castrejon 2646 (viridiflora)

Salinas, A. & A. Campos F-3670 (suberosa subsp. litoralis)

Salinas, A. et al. 5443, F-3735 (suberosa subsp. litoralis)

Saltis, T. 385/5 (pallida)

Salzmann, M. 288 (suberosa subsp. litoralis)

Sampaio 3907 (suberosa subsp. litoralis)

Sanabria, O. & P. Sima 159, 194 (pallida)

Sanchez, R. et al. 8345 (tenuiloba)

Sanchez, I. 2820, 4223 (suberosa subsp. litoralis)

Sandoval, E. & Chinchilla 495 (obtusifolia)

Sandoval, E. & A. Roman 1361 (obtusifolia)

Santa, J. & J. Brand 776 (coriacea)

Santa, J. & F. Buitrago 869 (coriacea); 898 (suberosa subsp. litoralis)

Santa, J. & M. Escobar 1089, 1091 (suberosa subsp. litoralis)

Santos, E. 1824 (suberosa subsp. litoralis)

Santos, E. & E. Trinta 2602 (suberosa subsp. litoralis)

Sarant et al. 57 (suberosa subsp. suberosa)

Saravia, E. 522 (suberosa subsp. litoralis)

Sargent, C. 36 (suberosa subsp. litoralis)

Sastre, C. & F. Sastre 2064 (suberosa subsp. suberosa)

Sastre, C. et al. 2566 (suberosa subsp. suberosa)

Saugster 540 (pallida)

Sauleda, R. et al. 3732 (pallida)

Saunders, J. 315, 515 (sexocellata)

Schimpff, H. 52 (tridactylites); 52 (suberosa subsp. litoralis)

Schinini, H. 4429, 10742, 14037, 14077, 14648 (suberosa subsp. litoralis)

Schinini, H. et al. 11361 (suberosa subsp. litoralis)

Schipp, W. 803 (sexocellata); S-603 (xiikzodz)

Schlieben, H. 11161, 11248 (pallida)

Schlmeyer 42, 331, 458 (pallida)

Schmalzel, R. X19 (megacoriacea)

Schneider, M. 749 (coriacea)

Schoenwetter, J. JSOX-9 (suberosa subsp. litoralis)

Schott, A. 2, 157, 898, 983 (pallida)

Schreberianum 1791, 1792, 1793 (pallida)

Schreiter, R. 1220, 5208, 5407, 9450 (suberosa subsp. litoralis)

Schultz 585 (pallida); 533, 534, 7770 (suberosa subsp. litoralis)

Schunke, J. 3823 (sexocellata); 6226 (coriacea); 10959 (sexocellata)

Schwacke, C. 2859, II-361 (suberosa subsp. litoralis)

Sect, A. 138A (suberosa subsp. litoralis)

Seemann 325 (pallida)

Sehnem, P. 3468 (suberosa subsp. litoralis)

Seidel, R. & M. Schulte 2525 (coriacea)

Semple, K. 1, 14 (megacoriacea)

Servin, B. 1412 (suberosa subsp. litoralis)

Sesse, M. & J. Mocino 4449, 4451 (pallida); 4457, 4458 (mcvaughiana); 4462 (obtusifolia); 4465 (suberosa subsp. litoralis); 4466 (pallida); 4474 (viridiflora); 30461 (obtusifolia)

Shafer, J. 36, 121, 380 (pallida); 424, 442, 620 (suberosa subsp. suberosa); 1013 (pallida); 2506 (suberosa subsp. suberosa); 2529, 2564, 2768 (pallida); 2788 (suberosa subsp. suberosa); 3071, 3073, 3208, 3597 (pallida); 3705 (suberosa subsp. suberosa); 10378 (pallida); 10474 (suberosa subsp. suberosa); 10564, 11677, 12145, 13509 (pallida)

Shannon, W. 274 (sexocellata)

Shapiro, G. 95 (sexocellata)

Sharp, A. 441532 (mcvaughiana)

Shattuck, O. 57 (megacoriacea)

Shreve, F. & E. Tinkham 9679 (pallida)

Sidwell, K. et al. 443 (eglandulosa)

Sigee, D. 55 (pallida)

Silseina, E. 815 (suberosa subsp. litoralis)

Silverstone-Sopkin, P. & J. Giraldo-Gensini 6180 (coriacea)

Silverstone-Sopkin, P. & N. Paz 3728 (coriacea)

Silverstone-Sopkin, P. et al. 3075, 3399, 3661, 5376, 6113 (coriacea)

Sima, P. 421 (pallida); 1382 (xiikzodz)

Simpson, J. 260 (pallida)

Sinclair, J. 6467 (pallida)

Sintenis, P. 644 (pallida)

Skovsted, A. 25 (pallida)

Slane, V. & C. Boatman 251SL (suberosa subsp. suberosa)

Small, J. 3928, 4087 (pallida)

Small, J. & J. Carter 63, 194, 202, 731, 790, 812, 2617, 2618, 2619, 3074, 8713, 8726, 8815, 8822, 8963 (pallida)

Small, J. & C. Mosier 5511, 5733, 5923, 6336 (pallida)

Small, J. & B. Nash 196 (pallida)

Small, J. & E. Small 5416 (pallida)

Small, J. & E. Wherry 11947 (tenuiloba)

Small, J. & P. Wilson 1815 (pallida)

Small, J. et al. 3399, 3586, 5787, 6586, 11088 (pallida)

Smith, A. 7288, 7923 (suberosa subsp. litoralis); 8087 (suberosa subsp. litoralis); 10323 (suberosa subsp. suberosa)

Smith, C. 1388 (pallida)

Smith, D. & J. Cabanillas 7120 (suberosa subsp. litoralis)

Smith, G. 10027 (suberosa subsp. suberosa); 10452 (pallida)

Smith, H. 1531 (pallida); 2624 (suberosa subsp. litoralis)

Smith, H. & G. Smith 615, 1314, 1315, 1616 (suberosa subsp. suberosa)

Smith, J. 1625 (clypeophylla)

Smith, J. & M. Butterwick 55, 211 (tenuiloba)

Smith, L. & P. Reitz 12264, 12264BR (suberosa subsp. litoralis)

Smith, L. et al. 3075 (pallida)

Smith, R. & class 4767 (pallida)

Smith, R. & T. Myint 757 (pallida)

Smith, R. et al. 890 (pallida)

Snedaker, S. D-63, E-169 (sexocellata)

Snodgrass, R. & E. Heller 321, 625 (tridactylites)

Snow 470 (tridactylites)

Solomon, J. & M. Nee 17942 (suberosa subsp. litoralis)

Soto, C. 298 (sexocellata)

Soto, J. et al. 3219 (suberosa subsp. litoralis); 12 503 (viridiflora)

Soto, J. & B. Boom 2101 (viridiflora)

Soto, J. & A. de Soto 3756 (viridiflora)

Soukup, J. 3753 (suberosa subsp. litoralis)

Sousa, M. 1348 (sexocellata)

Sousa, M. & C. Ramos 4872 (suberosa subsp. litoralis)

Sousa, M. et al. 5489 (obtusifolia)

Sparre, B. 15493 (sexocellata); 17922 (suberosa subsp. litoralis)

Specht, R. 160 (suberosa subsp. litoralis)

Spegarrini, C. 15544A (suberosa subsp. litoralis)

Spellman, D. 2120 (sexocellata)

Spellman, D. & Stoddart 2142, 2322, 2337, 2366, 2404, 2436 (pallida)

Sperry, O. T1295 (tenuiloba)

Spruce 4532 (coriacea)

Stahl, A. 608 (suberosa subsp. suberosa)

Standley, P. 9717, 10407 (suberosa subsp. litoralis); 18987 (pallida); 20188 (obtusifolia); 20602, 21306, 22723, 24014, 24607, 24959 (sexocellata); 25357 (pallida); 29594 (megacoriacea); 30733 (pallida); 31617 (megacoriacea); 33363, 41490 (suberosa subsp. litoralis); 52783, 54712, 56658 (sexocellata); 57852, 57884, 59341, 59534 (suberosa subsp. litoralis); 60135 (sexocellata); 61188, 61719, 63200, 63859 (suberosa subsp. litoralis); 65404 (eglandulosa); 66995, 70642, 70882, 72332, 73080 (sexocellata); 76851, 77083 (suberosa subsp. litoralis); 78869, 79022 (sexocellata); 80378, 80887, 80928 (suberosa subsp. litoralis); 83336, 84846, 84997, 85571, 86368, 86379 (eglandulosa); 87879, 88205, 88637, 88693, 91913 (sexocellata)

Standley, P. & E. Padilla 3444 (sexocellata)

Steere W. 1888 (sexocellata)

Steggerda, M. 240, 45a (pallida)

Stehle, H. 190, 253, 317, 1493 (pallida); 1586, 2600, 2727, 2879, 4335, 4511, 4845, 5629, 6219, 6411, 428a (suberosa subsp. suberosa)

Stehle, M. 6543 (suberosa subsp. suberosa)

Steinbach 2521, 2706 (suberosa subsp. litoralis)

Stern, W. et al. 2707 (pallida)

Stevens, B. & D. Yamamoto 3-13 (pallida)

Stevens, F. 322, 324 (suberosa subsp. litoralis); 1827, 6343, 6394 (pallida)

Stevens, F. & W. Hess 6135 (pallida)

Stevens, W. 1216 (suberosa subsp. litoralis)

Stevens, W. & B. Krukoff 7313 (sexocellata); 4162-b (suberosa subsp. litoralis); 8614 (sexocellata); 20764 (pallida)

Stevens, W. et al. 15609, 18533 (suberosa subsp. litoralis)

Stewart, A. 2074 (suberosa subsp. litoralis); 2075 (tridactylites); 2076, 2077, 2078 (suberosa subsp. litoralis); 2079 (tridactylites); 2080 (suberosa subsp. litoralis); 2081 (tridactylites); 2082 (suberosa subsp. litoralis)

Steyermark 29859 (eglandulosa); 30703 (suberosa subsp. litoralis); 32352 (eglandulosa); 32401 (suberosa subsp. litoralis); 34360, 35094, 36663 (eglandulosa); 39174 (sexocellata); 43598 (suberosa subsp. litoralis); 43787 (eglandulosa); 44120 (sexocellata); 46628, 46692 (eglandulosa); 50460, 50611, 50693, 51040, 55007, 56249, 88187 (suberosa subsp. litoralis); 92078 (coriacea)

Steyermark, J. & A. Braun 94621 (suberosa subsp. litoralis)

Steyermark, J. & B. Manara 110380 (pallida); 110380 (suberosa subsp. litoralis)

Steyermark, J. et al. 110061, 120139, 120174, 123006 (suberosa subsp. litoralis); 124671 (coriacea)

Stimson, W. 175, 1101 (pallida); 4031 (suberosa subsp. suberosa)

Stoddart, D. 66, 768, 847, 1067, 7026, 7090, 7231, 7263, 8146 (pallida)

Stoddart, D. & M. Poore 1366, 1403 (pallida)

Stoffers, A. 209, 545, 1304, 1693, 2036 (pallida); 3781, 3996, 4140 (suberosa subsp. suberosa)

Stone, B. 4235 (pallida)

Stork, H. et al. 25409 (obtusifolia)

Strandkrat, I. 1907 (pallida)

Strey, R. 5215, 8312 (suberosa subsp. litoralis)

Strieder, A. 33057 (suberosa subsp. litoralis)

Strother, J. 863 (tenuiloba)

Sturrock 192 (pallida); 506 (suberosa subsp. suberosa)

Suitenis 812 (suberosa subsp. suberosa)

Sumithraarachchi, D. & S.Waas DBS254 (suberosa subsp. litoralis)

Tamayo, F. 282 (suberosa subsp. litoralis)

Tan, B. & N. Raymond CR42 (pallida)

Tatnall, R. 744 (pallida)

Taylor, C. 2091 (sexocellata); 2663 (viridiflora); 2667 (viridiflora); 7835 (suberosa subsp. suberosa)

Taylor, C. & R. Gereau 10487 (pallida)

Taylor, C. & J. Miller 10409 (pallida); 10410 (suberosa subsp. suberosa)

Taylor, C. & B. Molano 8680, 8681 (pallida)

Taylor, C. & R. Ross 8179 (suberosa subsp. suberosa)

Taylor, C. et al. 10050 (pallida); 11489 (suberosa subsp. litoralis)

Taylor, M. 440 (tenuiloba)

Taylor, N. 79 (suberosa subsp. suberosa)

Taylor, T. TT126 (tridactylites)

Taylor, W. 49-1076 (pallida)

Tejada, R. 248 (sexocellata)

Tellez, O. 2054 (pallida)

Tellez, O. & E. Cabrera 1547, 2071, 2513 (pallida)

Tellez, O. & L. Rico 3357 (pallida)

Tenorio, P. et al. 384 (viridiflora); 2225 (suberosa subsp. litoralis); 3112 (viridiflora); 4018 (suberosa subsp. litoralis); 5576 (sexocellata); 6640 (suberosa subsp. litoralis); 8539 (sexocellata); 9383, 9444, 14178, 14839, 9262MX (suberosa subsp. litoralis)

Terborgh, J. & J. Brockmann 148 (pallida)

Teyer 251 (pallida)

Tharp, B. 1538, 2850, 3615, 3616, 43-720, 43-721 (tenuiloba)

Thieme, C. 5242 (sexocellata)

Thomas, W. 452, 2032, 2034 (macfadyenii)

Thompson, C. 237 (coriacea)

Thompson, J. 362, 367 (suberosa subsp. suberosa)

Thompson, S. & J. Nishida 2761 (pallida)

Thorne, R. 10186 (pallida)

Thorne, R. & G. Benny 44209 (pallida)

Till, W. 10248 (suberosa subsp. litoralis)

Tillich, J. 3558 (pallida)

Todd, S. 129 (pallida)

Togashi, M. 6211611 (suberosa subsp. litoralis)

Tonduz, A. 12808 (megacoriacea)

Toro, R. 29 (pallida)

Torres, R. & A. Garcia 6752, 6765 (obtusifolia)

Torres, R. et al. 549 (viridiflora); 9707 (sexocellata)

Totwall, E. 2 (tenuiloba)

Tracy, S. 7518, 7655, 9168, 9425 (pallida)

Tressens, S. et al. 4014 (suberosa subsp. litoralis)

Trinta, Z. et al. 1280 (suberosa subsp. litoralis)

Troncoso, N. & N. Bacigalupo 3003 (suberosa subsp. litoralis)

Tucker, G. 304 (pallida); 2577 (sexocellata)

Turner, B. 80-68M, 80-87M (tenuiloba)

Turner, B. & J. Crutchfield 80-48A (tenuiloba)

Turner, B. & G. Turner 15119 (tenuiloba)

Turner, B. & Z. Zhao 16016, 16025 (tenuiloba)

Tyson, E. 1054, 5454 (megacoriacea)

Tyson, E. & K. Blum 3901 (megacoriacea)

Ucan, E. 130 (pallida); 2303 (itzensis); 3947 (pallida)

Ucan, E. & M. Poot 5074 (pallida)

Ucan, E. & M. Ucan 2382 (pallida)

Underwood, L. & F. Earle 933 (pallida)

Underwood, L. & R. Griggs 472 (suberosa subsp. suberosa)

United Fruit Company 344 (megacoriacea)

Urban, I. 644 (pallida); 811 (suberosa subsp. suberosa); 3487, 3488, 3642 (pallida); 5114 (suberosa subsp. suberosa); 5667 (pallida); 5668 (suberosa subsp. suberosa)

Valerio, J. 270 (pallida); 2895 (sexocellata)

Valerio, M. A3 (coriacea)

Valiente, A. et al. 953 (suberosa subsp. litoralis)

van der Werff, H. 1095 (tridactylites); 1420, 1673 (suberosa subsp. litoralis); 1951 (tridactylites); 1951 (suberosa subsp. litoralis)

van Devender et al. 93-1500A, 93-66, 95-23A, 97-1043, 98-1420 (suberosa subsp. litoralis)

van Eenwyk, B. & C. Holway 4-126b (pallida)

van Hermann, H. 331, 781, 863, 914 (pallida)

van Wyk, A. 4256 (suberosa subsp. litoralis)

Vargas, C. & P. Sima 370, 450, 595 (pallida)

Vaughan, J. et al. 211 (sexocellata)

Velez, P. 2055 (suberosa subsp. litoralis)

Ventura, A. 4018 (sexocellata)

Ventura, E. & E. Lopez 338 (sexocellata); 7514 (suberosa subsp. litoralis)

Ventura, F. 1270 (sexocellata); 2379 (suberosa subsp. litoralis); 3324 (sexocellata); 3717, 5394 (suberosa subsp. litoralis); 5788 (pallida); 5788 (suberosa subsp. litoralis); 8538 (pallida); 10084, 10116, 11285 (suberosa subsp. litoralis); 14195, 16044, 19595, 19700, 20111, 20273 (sexocellata)

Venturi, S. 357, 1607, 4404, 5175, 7916 (suberosa subsp. litoralis)

Vergara, G. et al. 8570 (tenuiloba)

Viegas, G. & A.Viegas 2891 (suberosa subsp. litoralis)

Vigo, J. 10959 (coriacea)

Villacorta, R. 588 (sexocellata); 750 (eglandulosa)

Villacorta, R. & A. Gonzalez 683 (eglandulosa)

Villacorta, R. & L. Lara RV-02639 (eglandulosa)

Villacorta, R. & R. Rivas 2117 (sexocellata)

Villacorta, R. et al. 879 (obtusifolia)

Vincent, M. et al. 6026 (sexocellata)

Vogl, C. 1203 (suberosa subsp. litoralis)

von Tuerckheim, H. 1207 (trinifolia); 8215 (sexocellata); II2368 (trinifolia)

Wagner, M. 7 (megacoriacea)

Wagner, R. 1817 (suberosa subsp. suberosa)

Walker, R. 1172, 1264 (sexocellata); 1344 (suberosa subsp. litoralis)

Wallich 72 (pallida); 2008 (pallida)

Wallnofer, B. & F. Tut-Tesucun 5916, 6006 (pallida); 6066 (xiikzodz)

Ward, D. 5683 (tenuiloba); 5856 (pallida)

Ward, D. & D. Burch 3309, 3334, 3983 (pallida)

Ward, D. & G. Crosby 4839 (pallida)

Ward, D. & S. Ward 1777, 2333 (pallida)

Ward, D. et al. 5396 (pallida)

Warnock, B. 171, 485, 11251, 11707, 13494, 15857, 20083, T485, W171 (tenuiloba)

Warnock, B. & L. Hinckley 1875, 3749 (tenuiloba)

Warnock, B. & W. McBryde 15010 (tenuiloba)

Warshall, P. 106 (pallida)

Weaver, R. 2165 (suberosa subsp. litoralis)

Webber, H. 225 (pallida)

Webebauer, A. 7638 (suberosa subsp. litoralis)

Weberling, F. 2610 (eglandulosa)

Webster, G. 163, 177 (tenuiloba); 1072 (suberosa subsp. litoralis); 10914, 24186 (pallida)

Webster, G. & S. Lynch 17623 (pallida)

Wee-lek, C. 1448 (suberosa subsp. litoralis)

Weigend, M. 2190 (suberosa subsp. litoralis)

Weigend & N. Dostert 97/116 (suberosa subsp. litoralis)

Weigend & H. Forther 97/536, 97/922 (suberosa subsp. litoralis)

Weigend, M. et al. 97/318, 97/456 (suberosa subsp. litoralis)

Welch, W. 1547 (pallida)

Wendt, T. & E. Lott 1232 (tenuiloba)

West, E. & L. Arnold 153 (pallida); 240 (suberosa subsp. suberosa)

West, E. et al. 790 (pallida)

Weston, A. & J. Weston 5542 (pallida)

Whistler, A. 8169 (suberosa subsp. litoralis); 9837 (pallida); W4789, W5368 (suberosa subsp. litoralis)

White, G. 133 (megacoriacea)

White, P. 86 (megacoriacea)

Whitefoord, C. 1327 (sexocellata); 1439, 3758, 8223 (pallida)

Whitehouse, E. 25135 (tenuiloba)

Wiggins, I. 18513 (suberosa subsp. litoralis)

Wiggins, I. & D. Porter 403 (tridactylites)

Wight, A. 23, 156 (pallida)

Wilbur, R. & B. Jacobs 34677 (megacoriacea)

Wilkinson, E. 1900 (tenuiloba)

Willdenow 12380 (suberosa subsp. suberosa)

Williams, L. 5517, 5751 (coriacea)

Williams, L. & A. Molina 11822 (eglandulosa); 17799 (sexocellata)

Williams, L. et al. 25309 (suberosa subsp. litoralis); 25997, 26864 (eglandulosa)

Williams, R. 12568 (pallida)

Williges, G. 419 (tenuiloba)

Willschlaegel 1376 (pallida)

Wilson, P. 256, 533 (sexocellata); 7312, 8262, 9348 (pallida)

Wing, E. 38 (sexocellata)

Wirawan, N. 609 (pallida)

Wood, A. 683, 694 (tenuiloba)

Wood, D. 1681 (pallida)

Woodworth, R. & P. Vestal 501 (megacoriacea)

Worthington, R. 12000 (tenuiloba)

Woytkowski, F. 5421, 5491 (coriacea); 7101, 7101 (sexocellata)

Wright, C. 197 (pallida); 197 (suberosa subsp. suberosa); 216, 1083 (tenuiloba); 1245 (pallida); 1245 (suberosa subsp. suberosa); 1383 (tenuiloba); 2597 (pallida)

Wright, C. et al. 27 (suberosa subsp. suberosa); 28 (pallida); 30 (suberosa subsp. suberosa); 27&28, 2× (pallida)

Wullschlagel 238 (suberosa subsp. suberosa); 240, 235 (pallida); 239 (suberosa subsp. suberosa)

Wunderlin, R. 5135 (pallida)

Wunderlin, R. & J. Beckner 6490 (pallida)

Wurzburg Garden 3/7/92 (suberosa subsp. suberosa)

Wydler, H. 88 (suberosa subsp. suberosa)

Yanez, M. 826 (pallida)

Yuncker, T. 4775 (sexocellata); 17191, 17325, 18026, 18184 (pallida); 18425 (suberosa subsp. suberosa); 18631 (pallida)

Yuncker, T. et al. 8280 (sexocellata)

Zamora, P. 4644 (pallida)

Zanoni, T. 27976 (pallida)

Zanoni, T. & R. Garcia 41471 (suberosa subsp. suberosa)

Zanoni, T. & M. Mejia 16485 (suberosa subsp. suberosa); 17728 (pallida); 17780 (suberosa subsp. suberosa)

Zanoni, T. & J. Pimentel 25441, 25943 (pallida)

Zanoni, T. et al. 10740, 11433, 11670 (pallida); 24320, 24681, 28635, 34633 (suberosa subsp. suberosa); 37212, 37263, 39059 (pallida)

Zardini, E. 7744 (suberosa subsp. litoralis)

Zardini, E. & A. Aguayo 10551 (suberosa subsp. litoralis)

Zardini, E. & T. Tilleria 32349, 38858 (suberosa subsp. litoralis)

Zarucchi, J. & H. Cuadros 3934 (pallida)

Zarucchi, J. et al. 6102 (coriacea); 7248 (suberosa subsp. litoralis); 6102F (coriacea)

Zetek 5564, 5571 (megacoriacea)

Zuloaga, F. & N. Deginani 3492 (suberosa subsp. litoralis)

Zuloaga, F. et al. 6320 (suberosa subsp. litoralis)
